# Effects of Genetics and Sex on Acute Gene Expression Changes in the Hippocampus Following Neonatal Ethanol Exposure in BXD Recombinant Inbred Mouse Strains

**DOI:** 10.3390/brainsci12121634

**Published:** 2022-11-29

**Authors:** Jessica A. Baker, Jacob T. Brettin, Megan K. Mulligan, Kristin M. Hamre

**Affiliations:** 1 Department of Anatomy and Neurobiology, University of Tennessee Health Science Center, Memphis, TN 38163, USA; 2 Center for Behavioral Teratology, San Diego State University, San Diego, CA 92120, USA; 3 Department of Genetics, Genomics, and Informatics, University of Tennessee Health Science Center, Memphis, TN 38163, USA

**Keywords:** FASD, alcohol, development, brain, hippocampus, apoptosis, cell death, genetics, microarray, BXD recombinant inbred mouse strains

## Abstract

Fetal alcohol spectrum disorders (FASD) are prevalent neurodevelopmental disorders. Genetics have been shown to have a role in the severity of alcohol’s teratogenic effects on the developing brain. We previously identified recombinant inbred BXD mouse strains that show high (HCD) or low cell death (LCD) in the hippocampus following ethanol exposure. The present study aimed to identify gene networks that influence this susceptibility. On postnatal day 7 (3rd-trimester-equivalent), male and female neonates were treated with ethanol (5.0 g/kg) or saline, and hippocampi were collected 7hrs later. Using the Affymetrix microarray platform, ethanol-induced gene expression changes were identified in all strains with divergent expression sets found between sexes. Genes, such as *Bcl2l11*, *Jun*, and *Tgfb3*, showed significant strain-by-treatment interactions and were involved in many apoptosis pathways. Comparison of HCD versus LCD showed twice as many ethanol-induced genes changes in the HCD. Interestingly, these changes were regulated in the same direction suggesting (1) more perturbed effects in HCD compared to LCD and (2) limited gene expression changes that confer resistance to ethanol-induced cell death in LCD. These results demonstrate that genetic background and sex are important factors that affect differential cell death pathways after alcohol exposure during development that could have long-term consequences.

## 1. Introduction

Alcohol was identified as a teratogen almost 50 years ago, yet exposure to alcohol during pregnancy is still a leading cause of abnormal development throughout the world [1,2,3,4]. The umbrella term, fetal alcohol spectrum disorders (FASD), refers to the range of symptoms and effects due to exposure to alcohol during development [5]. Alterations associated with prenatal alcohol exposure includes alterations in cognitive, emotional, motor, and behavioral functions that present in childhood and can persist throughout life [6]. The central nervous system is extremely vulnerable to alcohol exposure including brain regions involved in learning and memory, such as the hippocampus [7,8]. In rodent models, the hippocampus is particularly vulnerable to alcohol-induced insults, including cell death, during the early postnatal period (equivalent to the 3rd trimester in humans) [7,9].

Genetics has been shown to be an important factor in both the presence and severity of FASD. In humans, there is a higher concordance of deficits seen in monozygotic twins compared to dizygotic twins [10,11,12]. Consistent with twin studies, the severity of alcohol-induced deficits in children varies even among mothers who consume approximately equivalent amounts of alcohol and at approximately the same time periods during their pregnancies [13]. Numerous studies in animal models also support the strong role of genetics by showing differential vulnerability to ethanol’s teratogenic effects across differing genetic backgrounds. A large range of phenotypes have shown differential sensitivity to developmental alcohol exposure including craniofacial dysmorphology [14,15], brain growth delays [16,17,18], cell death [16,19,20,21], epigenetic regulation [20,22], and gene expression [14,23,24].

Development of the nervous system is a highly regulated and organized molecular process controlled by gene expression in response to environmental cues. This well controlled system is extremely vulnerable to alcohol during development which has been shown to alter expression of genes involved in a range of critical processes [25,26,27,28,29,30,31]. Developmental alcohol exposure has been shown to have both short-term [28,30]; and long-term consequences to the transcriptome [25,27,31]. These ethanol-induced gene expression changes have also been found to be dependent on tissue [23,29], developmental time of exposure [27], and sex [28]. Additionally, genetic variation has been shown to affect gene expression changes after exposure to developmental alcohol [14,23,24].

Differences in ethanol-induced gene expression have been identified in two well studied strains of mice, the C57BL/6J (B6) and DBA/2J (D2) strains which have demonstrated differential susceptibility to the teratogenic effects of ethanol [23,24]. In fact, a valuable tool for studying genetic variation are mouse strains that differ in phenotypic responses such as the BXD recombinant inbred (RI) strains of mice which are generated by crossing B6 and D2 strains and inbreeding for over 20 generations [32,33]. The BXD strains have shown differential vulnerability to several developmental phenotypes and malformations after exposure to developmental alcohol [20,34]. Previously, our lab observed differential vulnerability to ethanol-induced apoptosis in the hippocampus using BXD and parental strains [20]. Apoptosis is a critical phenotype in ethanol’s effects on the nervous system because (1) it is ubiquitously observed after ethanol exposure throughout the life-span, and (2) it is one of the earliest responses of the nervous system to ethanol exposure. By identifying mean levels of caspase-3 positive cells, we identified four BXD strains that showed high susceptibility to ethanol-induced cell death (termed high cell death or HCD strains) and three BXD strains that showed low vulnerability (termed low cell death or LCD strains) after exposure to neonatal ethanol exposure [20].

Although the aforementioned studies support the role of genetic background in susceptibility to neurodevelopmental abnormalities after exposure to ethanol during development, there have been limited studies evaluating gene expression changes across these strains or other large populations [23,24]. Comparison of ethanol-induced gene expression between these BXD strains could help to better understand genetic susceptibility to FASD and identify mechanisms involved in ethanol-induced cell death. Further, gene expression analyses could assist in identifying potential candidate genes that mediate this differential response and underlie the previously identified quantitative trait locus (QTL) [35]. In addition, examination of both males and females will help identify sex-specific differences within or between strains that show differential vulnerability to ethanol-induced cell death in the neonatal hippocampus. Recent research has shown that males and females have different molecular pathways underlying cell death and we hypothesize that this is also true for ethanol-induced cell death [36,37,38,39].

In the present study, hippocampal gene expression was examined in parental B6 and D2 strains and six BXD RI strains that display differential vulnerability to cell death in the hippocampus seven hours after exposure to ethanol in neonates. Males and females were examined separately to address the effect of sex on ethanol-induced gene expression changes. Enrichment analysis was used to identify biological and molecular pathways associated with differentially expressed genes. We hypothesize that the previously identified high cell death strains (HCD) will show greater differential gene expression after exposure to developmental alcohol compared to the low cell death strains (LCD). We also theorize that there will be some sex-specific differences in hippocampal gene expression in these strains as sex-dependent gene expression changes after gestational alcohol exposure has recently been reported as well as some sex-by-genotype interactions [28]. Further, we propose that evaluation across strains will identify potential genetic pathways that mediate differential susceptibility in ethanol-induced cell death.

## 2. Materials and Methods

### 2.1. Animals and Numbers

All treatments and experiments were approved by the Institutional Animal Care and Use Committee at the University of Tennessee Health Science Center (UTHSC). Mice were maintained on a 12:12 hour light:dark cycle, given food and water ad libitum, and environmental enrichments were present. Based on previously identified phenotypes, the following strains were examined: B6 (C57BL/6J), D2 (DBA/2J), BXD2, BXD48a, BXD60, BXD71, BXD73, and BXD100 [20]. Mice were obtained from either the Center for Integrative and Translational Genomics (CITG) at UTHSC (Memphis, TN, USA) or Jackson Laboratory (Bar Harbor, ME, USA). Breeding was conducted at UTHSC. Pregnant dams were singly housed and the date of birth was recorded and defined as postnatal day (P) 0. To control for differences in maternal care, the first litter from each dam was not used. Only litters of 4 or more pups were used and large litters were culled to 8 pups. Pups were then left undisturbed until treatment on P7. Only one sex pair per treatment group was used from each litter. From each litter only 1 ethanol-exposed male, 1 ethanol-exposed female, 1 control male, and 1 control female were used (a minimum of 4 litters per treatment group, per sex). From these litters, a total of 128 samples were used—4 samples per treatment (control, ethanol), per sex (male, female), and per strain (B6, D2, BXD2, BXD48a, BXD60, BXD71, BXD73, BXD100) (Supplementary Table S1). Because all animals in a litter do not respond equally to the ethanol exposure and to avoid selection bias, animals from each litter were randomly chosen and assigned to treatment groups.

### 2.2. Ethanol Exposure

**Figure 1:**
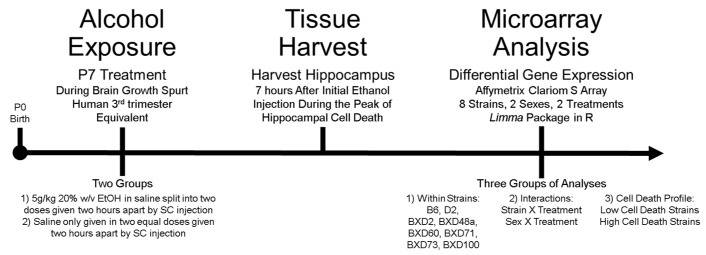
Overview of experimental design. Alcohol exposure paradigm (**left**), tissue harvest (**middle**), and microarray analysis (**right**) are shown.

Neonatal mice were treated on P7 during the brain growth spurt, which is a developmental time point where brain development is equivalent to the mid-3rd-trimester in humans [40]. Pups were split into either ethanol or control groups (Figure 1). As in our initial study [20], ethanol treated animals received 20% ethanol in sterile saline through subcutaneous injection. The total dose of ethanol was 5.0 g/kg split in two 2.5 g/kg doses, given two hours apart (starting between 9:00 A.M. and 10:00 A.M.) while controls received isovolumetric injections of saline [20]. This ethanol exposure represents an acute neonatal binge which has been shown to produce blood alcohol concentrations (BAC) of approximately 350 mg/dL in P7 neonatal mice [20,41]. Early prenatal and postnatal rodent studies of BACs found limited differences in BACs levels across multiple strains, though not enough to explain the phenotypic differences [17,42]. Littermates were used when possible and a maximum of one male and one female per group were used per litter.

### 2.3. Tissue Harvest and RNA Extraction

Animals were sacrificed 7 h after the first injection, which is the peak of ethanol-induced cell death in the hippocampus [7]. Pups were briefly exposed to isoflurane and quickly decapitated. The hippocampus was dissected, flash frozen in liquid nitrogen, and stored at −80 °C until processing. Purification of total RNA, including DNASE, was accomplished with the RNeasy Mini Kit (Qiagen Sciences Inc., Germantown, MD, USA) using the Qiagen QIAcube (Qiagen Sciences Inc., Germantown, MD, USA) following the manufactures protocol for purification of total RNA from easy-to-lyse animal tissues and cells. RNA concentration and purity were measured using NanoDrop 1000 Spectrophotometer (NanoDrop Technologies, Wilmington, DE, USA). RNA integrity was evaluated using Eukaryote Total RNA Nano Chip and measured using the Agilent 2100 Bioanalyzer (Agilent, Santa Clara, CA, USA). The average RIN was 9.87 + 0.17, indicating excellent sample quality.

### 2.4. Gene Expression Microarray & Data Processing

The Affymetrix Genechip Mouse Clariom S was used to examine gene expression (Affymetrix, Santa Clara, CA, USA). Sample processing and generation of Microarray data was completed by experienced technicians in the Molecular Resource Center at UTHSC. In brief, two hundred nanograms of DNase-treated total RNA was amplified, labeled, and fragmented using Ambion Whole Transcript (WT) Expression Kit according to the manufacturer’s protocol (Thermo Fisher Scientific, Santa Clara, CA USA). Samples were hybridized overnight according to manufacturer’s protocols; samples were then washed and stained on Affymetrix GeneChip Fluidics Station 450 (Affymetrix, Santa Clara, CA, USA) followed by scanning on the GeneChip Scanner 3000 (Applied Biosystems, Waltham, MA, USA). Data was normalized and analyzed for quality control in Affymetrix Expression Console Software using RMA-sketch normalization (Affymetrix, Santa Clara, CA, USA). After normalization and quality control, a total number of 22,203 probe sets were used for subsequent data analysis. A total of 128 samples were used—4 samples per treatment (control, ethanol), per sex (male, female), and per strain (B6, D2, BXD2, BXD48a, BXD60, BXD71, BXD73, BXD100) (Supplemental Table S1).

### 2.5. Differential Expression and Statistical Analyses

Differential expression was determined for each strain and sex by empirical Bayes-moderated *t*-statistics using the Bioconductor *limma* package (version 3.13) in the R (version 4.1) software environment [43,44]. The Benjamini-Hochberg post hoc test was used to correct for multiple testing or False Discovery Rate (FDR) [45,46].

#### 2.5.1. Analysis 1: Ethanol-Induced Differential Expression within Strains

Ethanol-induced gene expression changes initially were identified for each strain separately. In addition, males and females within each strain (e.g., BXD2 Male, BXD2 Female, etc.) were analyzed separately in order to assess possible sex differences within each strain. Significant differential expression within strain and sex was defined as an adjusted (*adj*) *p*-value < 0.05.

#### 2.5.2. Analysis 2: Treatment Interactions

In order to investigate differential strain and differential sex responses of ethanol-induced gene expression, the following interactions were examined: strain × treatment and sex × treatment. These analyzes were also conducted using the Bioconductor *limma* package and involved moderated *F*-statistics that combined the *t*-statistics for strain (strain × treatment) and sex (sex × treatment) for an overall test of significance for each gene [44]. Average fold change across strains or sex was used in these analyses. Significant interactions were defined as *adjp* < 0.05 and FC > 1.5.

#### 2.5.3. Analysis 3: Comparison of Strains with Differential Cell Death Phenotype

BXD strains were grouped based on previously identified cell death profile, i.e., high cell death or low cell death in order to analyze the relationship between (1) hippocampal cell death phenotype, and (2) ethanol-induced gene expression changes (Figure 1) [20]. Due to significant sex differences, males and females were analyzed separately though any overlap between the sexes was reported. Significantly expressed genes across all high cell death strains (HCD) (BXD2, BXD48a, BXD100) and all low cell death strains (LCD) (BXD60, BXD71, BXD73) were identified. Of specific interest were genes that were (1) only significantly differentially expressed in the LCD but not in the HCD, (2) only significantly differentially expressed in the HCD but not the LCD, and (3) significantly differentially expressed genes across all BXD strains regardless of previously identified cell death profile.

### 2.6. Gene Enrichment Analysis

Enrichment analysis was performed using tools available at WebGestalt (www.webgestalt.org) [47,48,49], STRING (www.string-db.org) [50], and gProfiler (www.biit.cs.ut.ee/gprofiler/gost) [51]. For expanded descriptive analyses, differentially expressed genes were extended to include those significant at nominal *p*-values (*p* < 0.05) [52]. Gene Ontology (GO) analysis was performed to determine over-representation by functional categories. Kyoto Encyclopedia of Genes and Genomes (KEGG) Pathway analysis was used to identify significant pathways and molecular interactions based on published literature [53,54]. The Mammalian Phenotype Ontology (MP) [55] and Human Phenotype Ontology (HP) [56] were used to determine over-represented mammalian phenotypes and phenotypic abnormities found in human diseases, respectively. STRING was used to examine functional interactions and connectivity networks between genes [50]. Gene symbols were used as inputs for all lists and suggested parameters were used—at least 5 genes per category and significance of *adjp* < 0.05 (FDR) based on Benjamini-Hochberg adjustment for multiple testing [45]. Because the strains used in the current microarray study were chosen based on previously identified cell death phenotype, and because our primary interest is to identify genes and genetic networks involved in strain-specific ethanol-induced cell death in the postnatal hippocampus, we closely analyzed these functional databases for pathways relating to apoptosis or cell death. Other functional categories were additionally included in our analysis.

### 2.7. GeneNetwork Analysis

The microarray data is publicly available on GeneNetwork (www.genenetwork.org) and can be accessed using the following: Species: Mouse (mm10) > Group: BXD Family > Type: Hippocampus mRNA > Datasets: *Hippocampus Postnatal Day 7- Control, Male*, *Hippocampus Postnatal Day 7- Control, Female*, *Hippocampus Postnatal Day 7- Control, Male & Female Combined*, *Hippocampus Postnatal Day 7- Ethanol, Male*, *Hippocampus Postnatal Day 7- Ethanol, Female*, and *Hippocampus Postnatal Day 7- Ethanol, Male & Female Combined*.

In our previous study, we performed genetic analysis of ethanol-induced cell death phenotype in the hippocampus using BXD strains (GeneNetwork Trait ID: 16177) and identified a significant (LOD = 5.67, LRS = 26.13) quantitative trait locus (QTL) on distal chromosome (Chr) 12 and a suggestive (LOD = 3.59, LRS = 16.57) QTL on proximal Chr3 [20]. In the present study, we used the tools available on GeneNetwork.org to perform additional analyses, including identifying differential ethanol-induced gene expression changes that (1) correlate with our ethanol-induced cell death phenotype and (2) are located within the previously identified QTLs.

## 3. Results

The following study was designed to examine the effects of genetics and/or sex on ethanol-induced gene expression changes in the hippocampus of mice exposed to alcohol postnatally by examining gene expression in the hippocampus of multiple BXD strains that showed either high susceptibility to ethanol-induced cell death (BXD2, BXD48a, BXD100) or low vulnerability to ethanol-induced cell death (BXD60, BXD71, BXD73). We also examined the parental stains (B6 and D2) which show moderate levels of cell death in the hippocampus after ethanol exposure. Males and females were examined separately to address the effect of sex on ethanol-induced gene expression changes in the neonatal hippocampus. We focused our analyses on (1) differential ethanol-induced gene expression changes among strains and sexes, and (2) relationships between ethanol-induced gene expression changes in high cell death strains compared to low cell death strains.

### 3.1. Ethanol-Induced Differential Expression

**Figure 2:**
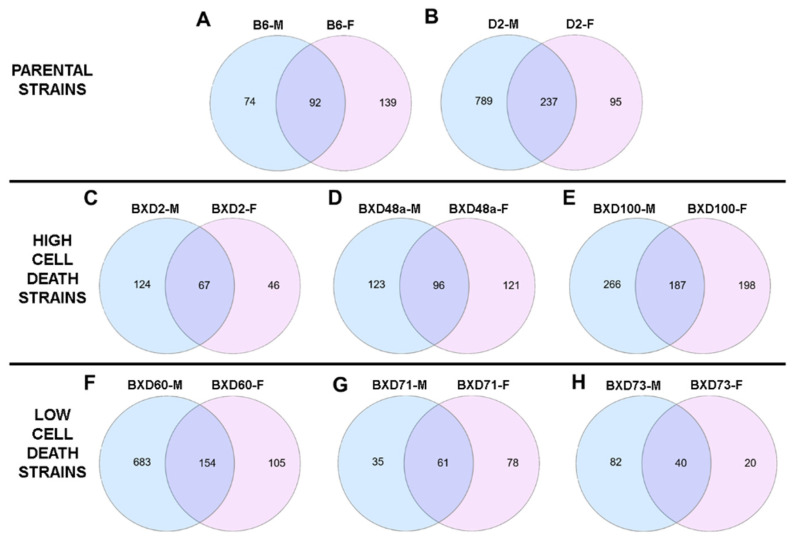
Sex-specific ethanol-induced gene expression for each strain. Differential effects of sex in the parental strains: (**A**) B6 and (**B**) D2 (top); the high cell death strains (middle): (**C**) BXD2, (**D**) BXD48a, and (**E**) BXD100; and low cell death strains (bottom): (**F**) BXD60, (**G**) BXD71, and (**H**) BXD73. For each strain, the number of significant (*adjp* < 0.05) ethanol-induced gene expression changes in males (blue circles, left) and females (pink circles, right). The purple overlap between the two circles (middle) represent number of significant (*adjp* < 0.05) ethanol-induced gene expression changes that were present in both males and females in each strain.

All strains showed specific genes whose expression was altered by exposure to neonatal ethanol (Supplemental Table S2). As hypothesized, ethanol-induced gene expression changes were differentially expressed among parental and BXD strains. Surprisingly, there was limited overlap in significant ethanol-induced gene expression changes between males and females of the same strain (Figure 2). Males exhibited more differentially expressed genes after ethanol exposure, with the exception of the B6 and BXD71 strains in which females showed more ethanol-induced expression changes than males. The largest number of ethanol-induced gene expression changes were found in D2 (Figure 2B) and BXD60 males (Figure 2F).

### 3.2. Interaction Effects and Pathway Analysis of Differentially Exprssed Genes

Ethanol-induced gene expression changes were examined for strain × treatment interactions using all strains and both sexes. There were 6863 genes that were significantly differentially expressed (*adjp* < 0.05) across the BXD and parental strains after exposure to neonatal ethanol. These genes were assessed using functional enrichment analysis on Webgestalt.org using multiple databases that analyzed gene functions. Because (1) the strains used in the current microarray study were chosen based on previously identified cell death phenotype and (2) our primary interest is to identify genes and genetic networks involved in strain-specific ethanol-induced cell death in the postnatal hippocampus, we closely analyzed these functional databases for pathways relating to apoptosis or cell death although other categories were analyzed as well.

**Figure 3:**
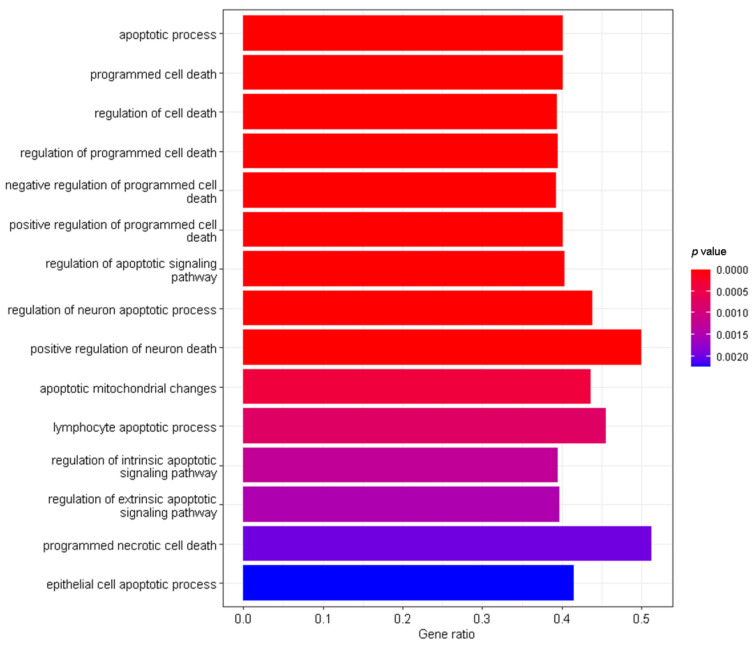
Cell death and apoptosis pathways significantly enriched among genes with significant strain × treatment interactions. Enrichment of Gene Ontology terms was quantified in order to identify over-represented categories of biological processes for our genes that showed a significant strain × treatment interaction. Numerous gene ontology pathways related to cell death and apoptosis were identified and representative pathways are shown. Enrichment analysis was performed using tools available at WebGestalt (www.webgestalt.org), with FDR *adjp* < 0.05. The y-axis lists significant biological processes related to cell death and apoptosis. The x-axis shows the ratio of the number of genes that were detected in our analysis to the total number of genes in each category.

**Figure 4:**
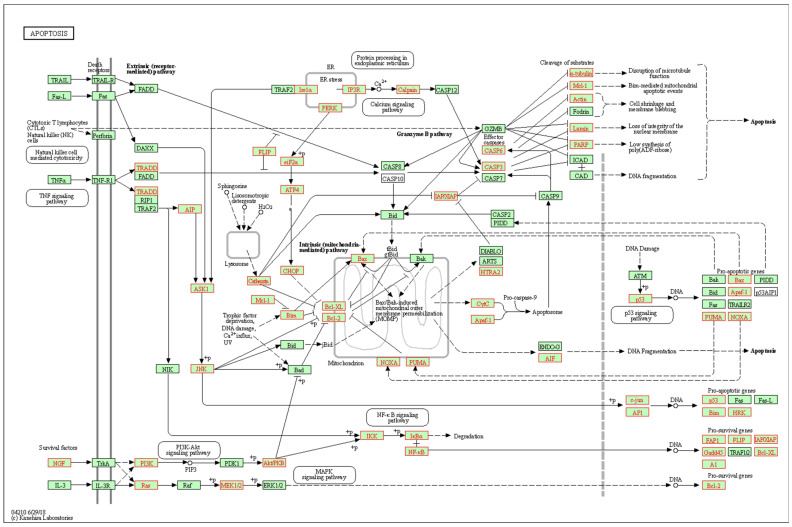
Differential ethanol-induced gene expression changes related to Apoptosis signaling. Over 50% of the genes in the KEGG Apoptosis Signaling pathway (mmu04210) showed a significant (*adjp* < 0.05) strain × treatment interaction (n = 73, total in pathway = 136). Differential ethanol-induced gene expression changes are shown in red font. Enrichment analysis was performed using tools available at WebGestalt (www.webgestalt.org), with FDR *adjp* < 0.05.

Gene Ontology (GO) was performed to identify over-represented categories of biological processes within the list of genes by parameters listed in the Materials and Methods. As shown in Figure 3, numerous cell death and apoptotic pathways were identified in genes that showed a significant strain × treatment interaction. Kyoto Encyclopedia of Genes and Genomes (KEGG) pathway analysis was performed to identify significant pathways and molecular interactions within the list of genes. Similarly, KEGG analysis also identified several significant signaling pathways that are related to apoptosis including p53, transforming growth factor-beta (TGF-β), neurotrophin, phosphatidylinositol 3′-kinase (PI3K)-AKT, mitogen-activated protein kinase (MAPK), and apoptosis signaling pathways. Over 50% of the genes in the KEGG Apoptosis Signaling pathway (Figure 4) showed a significant (*adjp* < 0.05) strain × treatment interaction (n = 73, total in pathway = 136). Other types of functional categories that were present in GO and KEGG analyses included, GO: alcohol and lipid metabolic processes, regulation of cell communication, signaling, immune system processes, cell differentiation, response to nutrient levels and growth factor, tissue development, and cell proliferation; KEGG: metabolic pathways, choline metabolism in cancer, RNA transport, axon guidance, and longevity regulating pathways.

Finally, Mammalian Phenotype (MP) Ontology and Human Phenotype (HP) Ontology were performed to identify over-represented phenotypes. MP analysis identified multiple phenotypes involved in cell death and abnormal brain morphology as well as behavioral phenotypes such as abnormal cognition, abnormal associative learning, and abnormal emotion/affect behavior (Supplemental Figure S1). In addition, HP analysis identified several phenotypes associated with FASD such as thin corpus callosum, abnormality of the philtrum, intellectual disability, and neurodevelopmental delay/abnormality [57].

**Figure 5:**
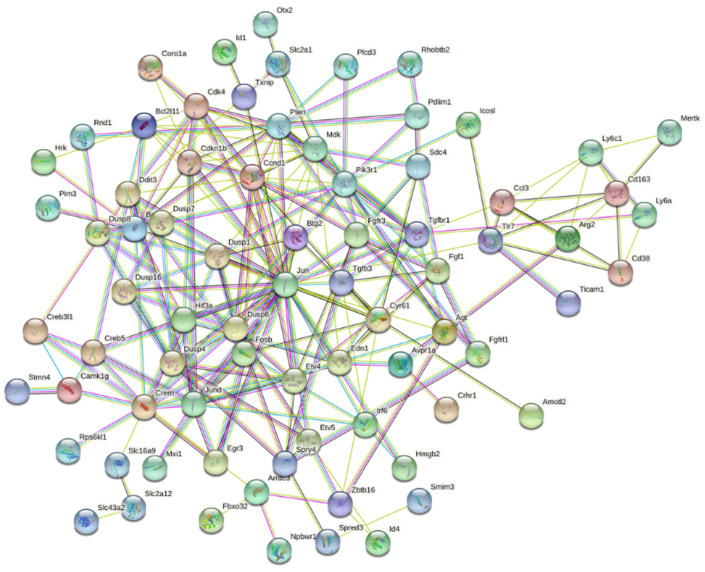
Network of inter-related genes that show differential gene expression changes after postnatal ethanol exposure. Many genes that showed a significant strain × treatment interaction (*adjp* < 0.05) and fold change greater than 1.5 were highly inter-related. The edges between nodes represent predicted functional associations: green line- neighborhood evidence, blue line- co-occurrence evidence, purple line- experimental evidence, yellow line- textmining evidence, light blue line- database evidence, and black line- co-expression evidence. Node colors are randomly assigned and known protein structures are depicted. Network analysis was performed using tools available at STRING (www.string-db.org), with FDR *adjp* < 0.05.

**Figure 6:**
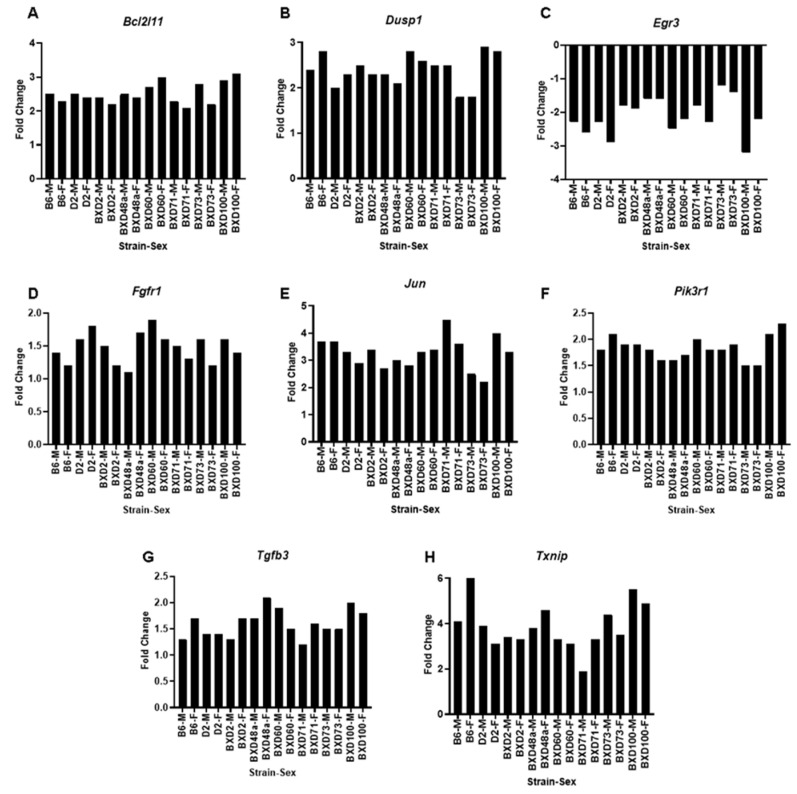
Apoptosis-related genes that show strain × treatment interaction and fold-change greater than 1.5. BXD and parental strains showed significant (*adjp* < 0.05; FC > 1.5) differential expression of many apoptosis-related genes including: (**A**) *Bcl2l11*, (**B**) *Dusp1*, (**C**) *Egr3*, (**D**) *Fgf1*, (**E**) *Jun*, (**F**) *Pik3r1*, (**G**) *Tgfb3*, and (**H**) *Txnip*. For each graph, the x-axis lists the strain and sex (M = male, F = female) and the y-axis represent the fold change relative to saline controls.

We also evaluated the set of genes demonstrating the largest differential expression changes based on the interaction between strain and alcohol treatment. The expression of 210 genes demonstrated large within-strain expression differences (FC > 1.5) between control and ethanol treated animals and the expression of each gene across the study cohort was significantly (*adjp* < 0.05) modulated by a strain × treatment interaction (Supplemental Table S3). Clustering analysis of these highly differentially expressed genes revealed a large network of inter-related genes (Figure 5). Many of these genes are involved in apoptotic and cell death related pathways, including *Bcl2l11* (BCL2 like 11), *Dusp1* (dual specificity phosphatase 1), *Egr3* (early growth response 3), *Fgf1* (fibroblast growth factor), *Jun* (jun proto-oncogene, AP-1 transcription factor subunit), *Pik3r1* (Phosphoinositide-3-kinase regulatory subunit 1), *Tgfb3* (transforming growth factor, beta 3), and *Txnip* (thioredoxin-interacting protein) (Figure 6). Next, ethanol-induced gene expression changes were examined for sex × treatment interactions using all strains and both sexes. There were no ethanol-induced gene expression changes that were significantly (*adjp* < 0.05) differentially expressed between males and females across the eight strains.

### 3.3. Genes of Interest within the Previously Identified Quantitative Trait Locus (QTL)

Previously, we performed genetic analysis of ethanol-induced cell death in the hippocampus using BXD strains [20]. We identified a significant (LOD = 5.67, LRS = 26.13) quantitative trait locus (QTL) on distal chromosome (Chr) 12 and a suggestive (LOD = 3.59, LRS = 16.57) QTL on proximal Chr3 [20]. In the present study, we analyzed genes that are within 1 LOD interval of the previously identified QTLs. Within the significant QTL on Chr12, we identified 42 genes that were also significant for a strain × treatment interaction. Two of these genes *Rps6kl1* (ribosomal protein S6 kinase like 1) and *Tgfb3* (Figure 6G) also showed an FC > 1.5. For the suggestive QTL on Chr3, we identified 17 genes that were significant for a strain × treatment interaction, though none of these genes demonstrated large ethanol-induced within strain differential expression (FC > 1.5). 

Using the tools available at GeneNetwork.org, we also correlated the hippocampal cell death phenotype (GeneNetwork Trait ID: 16177) with the ethanol-exposed hippocampal gene expression dataset. There were 296 genes that were significantly correlated (*p* < 0.05) with the hippocampal cell death phenotype. There were a number of genes related to apoptosis including *Bcl2* (B-cell CLL/lymphoma), *Cflar* (CASP8 and FADD-like apoptosis regulator), and *Nkg7* (natural killer cell group 7 sequence). A number of growth factor-related genes were also significantly correlated with the hippocampal cell death phenotype including *Igf2* (insulin-like growth factor 2), *Ngfr* (nerve growth factor receptor), and *Tgfbr3* (transforming growth factor beta receptor 3). Of the significant genes correlated with the hippocampal cell death phenotype, there were 107 genes that were significant for a strain × treatment interaction. Four of these genes also showed an FC > 1.5: *Gadd45g* (growth arrest and DNA-damage-inducible, gamma), *Kcnj13* (potassium inwardly rectifying channel, subfamily J, member 13), *Plekhg1* (pleckstrin homology domain containing, family G (with RhoGef domain) member 1), and *Sgms2* (sphingomyelin synthase 2).

### 3.4. Comparison between High Cell Death (HCD) Strains and Low Cell Death (LCD) Strains

Previously, BXD strains showed differential response to cell death in the CA1 region of the hippocampus after an acute binge-like alcohol exposure on P7 [20]. BXD2, BXD48a, and BXD100 were found to be more susceptible to ethanol-induced cell death in the hippocampus while BXD60, BXD71, and BXD73 were found to be less sensitive to ethanol-induced hippocampal cell death. For this second analysis, we only focused on these BXD strains and did not include the parental strains that showed moderate levels of ethanol-induced cell death. As discussed above, marked sex differences were found with little overlap within strains thus, males and females were analyzed separately. Of specific interest were genes that were (1) only significantly differentially expressed in the LCD strains but not in the HCD strains or (2) only significantly differentially expressed in the HCD strains but not the LCD strains. We also examined significantly differentially expressed genes across all BXD strains regardless of previously identified cell death profile.

**Figure 7:**
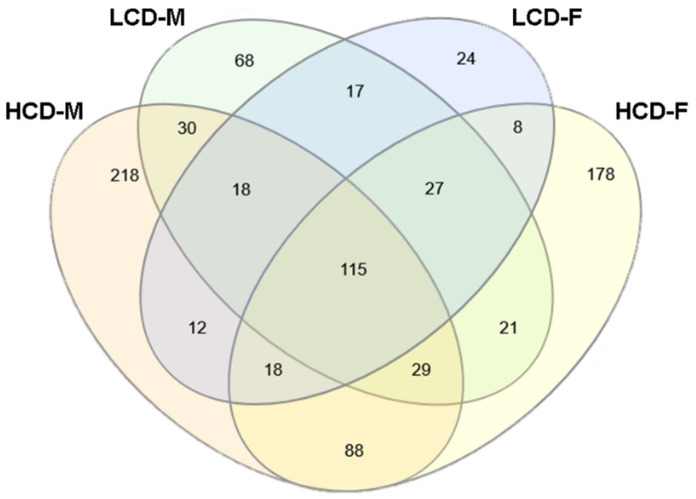
Venn diagram of differential gene expression changes in each group. Similarities and differences in significant ethanol-induced gene expression changes in all four groups. From left to right: high cell death males (HCD-M), low cell death males (LCD-M), low cell death females (LCD-F), and high cell death females (HCD-F).

**Figure 8:**
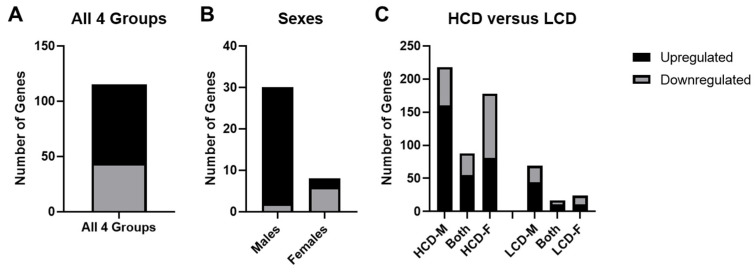
Upregulated and downregulated genes after ethanol exposure among different groups. (**A**) There were only 155 significant ethanol-induced gene expression changes that were similar between all four groups—71 upregulated, 44 downregulated. (**B**) Sex specific changes regardless of cell death profile were identified (males: 28 upregulated, 2 downregulated; females: 2 upregulated, 6 downregulated). (**C**) There were 484 unique genes that in the HCD strains (HCD-M only: 160 upregulated, 58; Both HCD-M & HCD-F: 55 upregulated, 33 downregulated; HCD-F only: 81 upregulated, 97 downregulated). There were 109 genes specific to LCD strains (LCD-M only: 44 upregulated, 25 downregulated; Both LCD-M & LCD-F: 11 upregulated, 6 downregulated; LCD-F only: 11 upregulated, 13 downregulated).

The number of overlapping significant (*p* < 0.05) genes across each group were as follows: 528 genes for high cell death strain males (HCD-M), 325 genes for low cell death strain males (LCD-M), 484 genes for high cell death strain females (HCD-F), and 239 genes for low cell death strain females (LCD-F). Comparison between all four groups revealed there were unique differences between the gene lists regardless of cell death profile or sex (Figure 7), with only 115 genes that were found in all four groups (Figure 8A). All of the genes that were significant in both the HCD and LCD strains showed a significant strain-by-treatment interaction including *Crhr1* (corticotrophin releasing hormone receptor 1), *Slc2a1* (solute carrier family 2 member 1), and *Dtna* (dystrobrevin alpha). There were some genes that were limited to one sex regardless of cell death profile—30 genes in males and 8 genes in females (Figure 8B).

There were 484 unique genes in HCD strains that were not present in LCD strains. Of these, 88 genes were significant for both HCD-M and HCD-F, while 218 were specific for HCD-M and 178 were specific for HCD-F (Figure 8C). Enrichment analysis identified several GO pathways related to cell death and apoptosis in both the HCD-M and HCD-F. In contrast, there were only 109 genes in LCD strains that were not present in HCD strains—17 genes were significant in both LCD-M and LCD-F, 68 were specific for LCD-M, and 24 specific for LCD-F (Figure 8C). While there were no cell death/apoptosis pathways identified in the LCD-M, there were a few identified in LCD-F. However, the HCD-M and HCD-F show a much larger number of ethanol-induced genes in cell death/apoptosis-related GO pathways compared to the LCD-F. Genes related to apoptosis pathways that were found in HCD strains but not LCD strains included *Ciapin1* (cytokine induced apoptosis inhibitor), *Gadd45g* (growth arrest and DNA damage inducible gamma), Id1 (inhibitor of DNA binding 1), *Igf2r* (insulin like growth factor 2 receptor), *Lifr* (leukemia inhibitor factor receptor), Mdk (midkine), and *Noc2l* (NOC2 like nucleolar associated transcriptional repressor).

## 4. Discussion

Genetics has been shown to be an important factor in both the presence and severity of FASD, however little is known about the molecular mechanisms underlying genetic variation in susceptibility to the teratogenic effects of alcohol. Previously, our lab observed differential vulnerability to ethanol-induced apoptosis in the neonatal hippocampus [20]—a brain region that is highly involved in many of the cognitive and neurobehavioral deficits present in FASD. In the current study, we examined the acute effects of ethanol by determining gene expression changes 7 h after the initial ethanol exposure. A number of studies in a variety of ages and neuronal cell populations have shown that developing neurons begin to die rapidly after ethanol exposure and that 7 to 8 h is the typical peak of cell death, including in the postnatal hippocampus [7,58,59,60,61]. Using a different age, we have examined strain differences in the timing of cell death in the BXD strains and found no evidence for a differential time course of cell death across strains [21], suggesting this is the most relevant time to evaluate gene expression changes. Therefore, in order to better understand the molecular mechanisms behind this differential sensitivity to ethanol-induced cell death, the current study examined ethanol-induced gene expression changes in the hippocampus of multiple BXD RI and parental mouse strains that exhibit differential vulnerability to cell death in the hippocampus after postnatal ethanol exposure.

From these analyses, we show the following. First, in addition to corroborating previous work showing genetic differences in responses to developmental ethanol exposure, we show that males and females have differential expression changes that are strain-specific [20]. While there were no overall sex × treatment interactions between males and females across all strains, there were sex-specific ethanol-induced gene expression changes within each strain. Previously we identified a genetic locus that underlies strain differences in response to developmental ethanol exposure [20]. In the present study, we extend this analysis through identification of potential candidate genes that could be mediating this differential response. Finally, we began to evaluate the molecular pathways and functional categories of genes that showed ethanol-induced changes in gene expression and show that there is an over-representation of changes related to cell death suggesting that there may be direct cause and effect between the phenotype of interest and the molecular pathways that mediate these differences.

### 4.1. Effects of Sex on Ethanol-Induced Gene Expression Changes

We observed significant effects of sex on gene expression changes after exposure to postnatal ethanol. Specifically, we identified ethanol-induced gene expression changes that were highly sex-specific with little overlap in ethanol-induced gene expression changes between males and females in the same strain. In six out of the eight strains examined, males showed significantly more ethanol-induced gene expression changes. This is consistent with previous literature that showed male fetuses are more vulnerable than female fetuses to at least some types of in utero perturbations and suggests that this issue should be further evaluated following developmental exposure to ethanol [36,38]. However, examination of the interaction between sex and treatment across all the BXD and parental strains revealed no significant effect of sex on ethanol-induced gene expression changes. The lack of significant interaction between sex and treatment is likely due to the fact that sex-specific ethanol-induced gene expression changes were specific within each strain and thus were not carried over across strains. This suggests that both strain and sex are important variables and may work in a synergistic fashion.

### 4.2. Effects of Strain on Ethanol-Induced Gene Expression Changes

From these analyses we were able to identify significant molecular pathways and genes both in terms of identifying potential candidate genes in relation to the previously identified QTL as well as identifying molecular pathways relevant to the cell death phenotype. We identified many ethanol-induced gene expression changes that show differential variation among the BXD RI and parental strains after exposure to ethanol during development. Enrichment analysis of these differential gene expression changes in response to postnatal alcohol exposure revealed numerous biological categories that were involved in apoptosis, either directly or indirectly, including p53, PI3K-Akt, MAPK, TGF-β, and Neurotrophin signaling pathways. Many genes that were involved in these apoptosis-related pathways were also involved in significantly enriched phenotypes that are related to FASD such as abnormality of the philtrum, intellectual disability, and neurodevelopmental delay/abnormality [57].

There are a number of possible mechanisms for gene expression changes. For example, we found several lines of evidence of transcription factor expression changes that could modulate further downstream gene expression such as *Jun*. Additionally, in a separate analysis, we examined B6 mice using the same ethanol paradigm as in the current study and showed epigenetic changes including histone modification in the hippocampus [41]. This suggests that these epigenetic alterations could play a role in the gene expression changes across the BXD strains.

Interestingly, multiple genes that were significant for a strain × treatment interaction and showed a fold-change greater than 1.5 are involved in apoptosis-related pathways and have previously been linked to FASD including *Bcl2l11* [62], *Dusp1* [62], *Egr3* [62,63], *Fgf1* [64,65] *Jun* [62,66], *Pik3r1* [62] *Tgfb3* [62,67], and *Txnip* [62,66]. Moreover, many genes that showed differential ethanol-induced gene expression changes have been previously linked to gene expression changes in the brain of B6 mice using the same ethanol exposure paradigm as used in the current study [62]. Specifically, top genes identified in the present study, *Bcl2l11*, *Jun*, *Tgfb3*, and *Txnip,* have been previously identified as central genes in significant networks affected by third-trimester-equivalent ethanol exposure [62].

Genes that were up-regulated after ethanol exposure and involved in cell death pathways included *Bcl211*, *Dusp1*, *Jun*, *Pik3r1*, and *Txnip*. *Bcl2l11* is a member of the BCL-2 family that has been shown to act as an apoptotic activator [68]. *Dusp1* is member of MAPK pathway that has been shown to promote apoptosis and alter neuroinflammation [69,70,71]. *Jun* is a transcription factor that has been shown to induce apoptosis, among its many other functions [72]. *Pik3r1* is a member of PI3K/AKT pathway that is involved in growth factor responses and cell survival [73,74]. *Txnip* is a member of the alpha arrestin family whose upregulation has been linked to inflammasome activation and apoptosis [75]. There were also a number of growth factors or growth factor-related genes that have been linked to either apoptosis or neuroprotection that were significantly differentially expressed among the strains after ethanol exposure. *Egr3*, an immediate-early growth response gene induced by mitogenic stimulation [76], was down-regulated after ethanol exposure. In contrast, the growth factors *Fgf1* and *Tgfb3* were up-regulated after ethanol exposure. Fgf1 is a member of the fibroblast growth factor family and is involved in cell survival [77]. *Tgfb3* is a member of the transforming growth factor-beta superfamily involved in recruitment and activation of transcription factors [78,79]. In addition, *Tgfb3* has been shown to dramatically increase potency of other growth factors [80] and promote and accelerate apoptosis [81].

Interestingly, *Tgfb3* was identified within the significant QTL for our ethanol induced-hippocampal cell death from our previous study [20]. Another gene that was within the previously identified QTL and was differentially expressed in BXD strains after exposure to postnatal ethanol was *Rps6kl1*, a member of the ribosomal S6 kinase family involved in protein synthesis, cell growth, and cell proliferation. In addition, many genes that were correlated with our hippocampal cell death phenotype were related to growth factors including *Igf2*, *Ngfr*, and *Tgfbr3*. Thus, there are several differentially expressed genes within the previously identified QTL that are good candidate genes and deserve further investigation, starting with *Tgfb3*.

### 4.3. Comparison of Ethanol-Inuced Gene Expression Changes between HCD & LCD Strains

One of the goals of the present study was to evaluate multiple strains that showed the high cell death phenotype and compare the results to multiple strains with a low cell death phenotype. The expectation is that molecular changes found across multiple genetic backgrounds will have greater translational relevance and this type of strategy has been used for other phenotypes [82,83,84,85]. We identified marked differences in ethanol-induced gene expression changes between the HCD strains and LCD strains. Since there were such robust sex differences within each strain, we analyzed males and females separately for our analysis of ethanol-induced gene expression changes in strains. When comparing the number of genes that were significantly expressed in all three HCD strains or all three LCD strains across the two sexes, there were more ethanol-induced gene expression changes in the males compared to the females.

The number of ethanol-induced gene expression changes were much higher in the HCD strains compared to the LCD strains in both sexes; HCD-M showed 1.6× more ethanol-induced gene changes than LCD-M while HCD-F had double the number of genes changes after ethanol exposure compared to LCD-F. This suggests that resistance or susceptibility may be due to different levels of expression rather than due to something that is only expressed in either the susceptible or resistant strains. Moreover, an interesting finding was that significant ethanol-induced gene expression changes in the HCD and LCD strains were always regulated in the same direction, i.e., there were no significant gene expression changes that were regulated in opposing directions. These results suggest more perturbed effects of ethanol in the HCD strains compared to the LCD strains, and this could be why we see more significant cell death in the hippocampus in the HCD strains than the LCD strains. The results also suggest there may be limited gene expression changes that confer resistance to ethanol-induced cell death in the hippocampus in the LCD strains. This notion that vulnerable strains show more perturbed gene expression changes compared to resistant strains while resistant strains show limited gene expression changes that could account for protection against ethanol’s teratogenic effects has been previously proposed in a study that compared ethanol-induced gene expression changes in two strains that showed differential vulnerability to ethanol exposure during embryonic development [23].

Enrichment analysis found several apoptosis and cell death pathways that were unique to HCD-M compared to LCD-M. While females in both HCD and LCD strains showed cell death pathways, the HCD-F showed a much larger number of genes in these pathways compared to the LCD-F. These results demonstrate that there are substantial variations in differential ethanol-induced gene expression and subsequent biological pathways between the HCD and LCD strains. Some apoptosis-related genes that were found in HCD strains but not LCD strains included *Ciapin1*, *Gadd45g*, *Id1*, *Igf2r*, *Lifr*, Mdk, and *Noc2l*. *Ciapin1* and *Noc2l* both have been found to suppress p53-induced apoptotic signaling and both were significantly decreased in the HCD strains [86,87]. *Ciapin1* is regulated by cytokines and growth factors to inhibit apoptosis [88] and *Noc2l* modifies transcription by inhibiting histone acetyltransferase activity [89]. Other apoptosis-related genes were upregulated in HCD strains, including *Gadd45g*, *ILg2r*, *Lifr*, and *Mdk*. The stress-responsive gene that contributes to p53 activation [90,91], *Gadd45g*, is involved in brain development, cognition, and has been linked to neurodevelopmental diseases [92,93,94]. The transcriptional repressor, *Id1*, has been shown to interact with sonic-hedgehog signaling to trigger neuronal death through caspase-dependent pathways [95]. Other apoptosis-related genes that were upregulated in HCD strains were linked to neuroinflammation including the growth factor *Mdk* [96] and growth factor receptors, *Igf2r* [97] and *Lifr* [98]. Interestingly, many of these apoptosis-related genes that were only found in HCD strains have been linked to TGFβ signaling including *Gadd45g* [93], *Id1* [99], and *Igf2r* [100].

All of the genes that were significant in both the HCD and LCD strains showed a significant strain-by-treatment interaction and many have been previously linked to FASD. In both the HCD and LCD strains, *Crhr1* was downregulated after ethanol-exposure, a finding similar to that from a study using a prenatal model [101]. *Crhr1* is a stress-related receptor that is a major regulator of hippocampus development and function [101,102]. Both *Slc2a1*, and *Dtna* were upregulated in HCD and LCD strains. *Slc2a1* is a glucose transporter involved in angiogenesis and has been linked to abnormal vasculature development in FASD [103]. *Dtna* is a dystrophin-associated protein involved in the formation and stability of synapses and has recently been identified as a potential biomarker for FASD in humans [104,105].

Several genes unique in the HCD strains have been previously linked to FASD and apoptosis including *Abca1* (ATP binding cassette subfamily A member 1), *Eng* (endoglin), *Elavl2* (ELAV like RNA binding protein), *Igf2r* (insulin like growth factor 2 receptor), *Napepld* (N-acyl phosphatidylethanolamine phospholipase D) and *Vegfa* (vascular endothelial growth factor A). Males and females in the HCD strains showed ethanol-induced downregulation of *Elavl2*, a neuron-specific gene associated with development, and upregulation of *Igf2r*, a growth factor receptor linked to activation of TGFβ. Previous studies on prenatal alcohol exposure have reported alterations in *Elavl2* [106] and *Igf2r* [107] including methylation changes of both genes [108].

Males in the HCD strains showed downregulated of *Vegfa*, a member of both the platelet-derived and vascular endothelial growth factor families that has been shown to suppress apoptosis [109]. This ethanol-induced downregulation is consistent with previous FASD literature that also show decreases in *Vegfa* expression after exposure to ethanol during development [110,111]. In contrast, *Napepld*, an enzyme involved in endocannabinoid signaling [112], was upregulated after ethanol exposure in males in the HCD strain. Previous studies have also reported increased levels of *Napepld* after ethanol exposure [113] including in the hippocampus of mice given the same postnatal ethanol exposure used in the current study [114]. Females in the HCD strains showed upregulation of two genes that have been previously linked to FASD [115,116,117,118], *Abca1*, a cholesterol transporter [119], and *Eng*, a glycoprotein and component of TGFβ receptor complex [120,121], which have both been linked to FASD. Moreover, sex-dependent increase of *Abca1* was previously reported in the neocortex of rodent fetuses after prenatal ethanol exposure [115]. Interestingly, both *Abca1* and *Eng* are linked to multiple growth signaling pathways including insulin-like (IGF) [118], platelet-derived growth factor (PDGF) [122,123], TGFβ [124,125,126,127], and vascular endothelial growth factor (VEGF) [128].

A few genes that were unique to LCD strains have been previously linked to FASD including *Atf6* (activating transcription factor 6), *Irs2* (insulin receptor substrate 2), *Fgf1*, and *Uhrf1* (ubiquitin like with PHD and ring finger domains 1). Both *Irs2* and *Fgf1* were up regulated in LCD males after ethanol exposure. Interestingly, studies that previously reported ethanol-induced alterations in *Irs2* and *Fgf1* reported down-regulation after ethanol exposure [64,129,130]. However, sex-specific increases of *Irs2* in males after exposure to prenatal ethanol have also been reported [131]. In LCD females, *Atf6*, a transcription factor involved in unfolded protein response, was up-regulated after ethanol exposure during development which is consistent with previous reports [132,133]. In contrast, *Uhrf1*, a key epigenetic regulator, was down-regulated in LCD females which is inconsistent with other studies that have reported ethanol-induced increases of Uhrf1 [134,135].

### 4.4. Limitations & Future Directions

There are some limitations of this study that are worth mentioning. First, while we show significant differential gene expression among BXD RI and parental strains after exposure to postnatal ethanol, there could be other sources of variation that we did not account for in the current study. Second, we used subcutaneous injections to administer ethanol, and this is not a traditional route of ethanol exposure in humans [136]. In a related limitation, animals were given ethanol only during the third-trimester-equivalent which is not a typical drinking pattern observed in humans [137]. Nevertheless, the ethanol paradigm used in the current study is a common method to examine these important hippocampal cells that are intimately involved in learning and memory, at a time when they are highly sensitive to ethanol-induced cell death [7,59,136,138]. Third, the purpose of the current study was to examine initial gene expression responses to ethanol exposure. Thus, this analysis was conducted while ethanol was presumably still in the system and the expression changes would likely differ if examined at a later timepoint. Fourth, although we tested 128 samples in our microarray study, our sample size per strain, per sex, per treatment was relatively low and further analysis is needed with enhanced sample sizes. Fifth, we used the whole hippocampus in this analysis. It is possible that, because of the mixture of cell types that were present, important changes in gene expression that were restricted to specific cell types were not detected.

In our study, we used microarrays which are a hybridization-based technique used to detect predefined RNA sequences within a sample [139]. In contrast, newer next-generation RNA-sequencing (RNA-seq) do not use hybridization and instead use a sequence-based technique that is not reliant on predefined sequence information and therefore can detect novel sequences and splice variants [139]. RNA-seq studies have been shown to have higher detection of low abundance transcripts and higher resolution of differentially expressed genes compared to microarrays [140]. However, overall microarrays and RNA-seq are comparable techniques to analyze gene expression changes. Microarrays were chosen for the current study due to (1) cost effectiveness for our large study across eight strains, two sexes, and two treatments, (2) turn-around time for results, and (3) numerous other microarray expression data is available for the BXD RI strains on GeneNetwork.org, though future studies on the effects of developmental alcohol exposure should explore the use of RNA-seq. As discussed above, a higher sample size would also allow for data mining of control tissue to assess possible genetic pathway that predict vulnerability to ethanol-induced cell death.

## 5. Conclusions

To our knowledge, this is the first study using the BXD RI strains to examine the effects of genetics and sex on ethanol-induced gene expression changes during development. Overall, our study identified numerous effects of strain on hippocampal gene expression changes after exposure to postnatal ethanol. Within each strain there was little overlap of differential expression between males and females although there were no interactive effects of sex × treatment across all strains. We identified numerous strain differences in gene expression changes after ethanol exposure. Many of our top genes that showed ethanol-induced expression differences among the strains were found to be previously linked to cell death and apoptosis, as well as many which have previously been linked to FASD. In addition, many genes identified were growth factors or in growth factor pathways. Ethanol-induced dysregulation of growth factors during development could contribute to increased cell death after ethanol exposure.

An advantage of the current study was the comparison between ethanol-induced gene expression changes in strains that showed differential vulnerability of cell death in the hippocampus after postnatal ethanol exposure. This allowed us to compare gene expression changes after ethanol exposure in multiple strains that have been shown to be susceptible to hippocampal cell death and multiple strains that have shown to be resistant to ethanol-induced cell death in the hippocampus. We observed more perturbed effects of ethanol in the high cell death strains compared to the low cell death strains. Future studies are needed to understand how these genes directly affect cell death in the hippocampus and to evaluate the long-term consequences of ethanol-induced differential gene expression and hippocampal cell death in these diverse strains. Behavioral studies examining the effects of postnatal ethanol exposure on learning and memory in BXD strains could further our understanding of the genetic variation seen in ethanol teratogenicity and its long-term effects on behavior.

## Data Availability

The original contributions presented in the study are included in the article and Supplemental Materials. The phenotype and microarray data are publicly accessible and available at GeneNetwork (www.genenetwork.org). Further inquiries can be directed to the corresponding author (K.M.H.).

## Supplementary Materials

The following supporting information can be downloaded at: https://www.mdpi.com/article/10.3390/brainsci12121634/s1, Figure S1: Mammalian phenotype ontology categories significantly enriched among genes with a significant strain × treatment interaction; Table S1: Animal numbers used for microarray analyses; Table S2: Significant (*adjp* < 0.05) ethanol-induced gene expression changes within each strain; Table S3: Differentially expressed genes with significant (*adjp* < 0.05) strain × treatment interaction and fold change > 1.5.
