# Supplementary material for: Effects of Genetics and Sex on Acute Gene Expression Changes in the Hippocampus Following Neonatal Ethanol Exposure in BXD Recombinant Inbred Mouse Strains
Source: Brain Sci. 2022 Nov 29;12(12):1634. doi: 10.3390/brainsci12121634 (PMC9776411; doi:10.3390/brainsci12121634)
Supplement: Supplementary file 1 [file brainsci-12-01634-s001.zip › Baker et al 2022 Supplemental Figure S1.pdf]

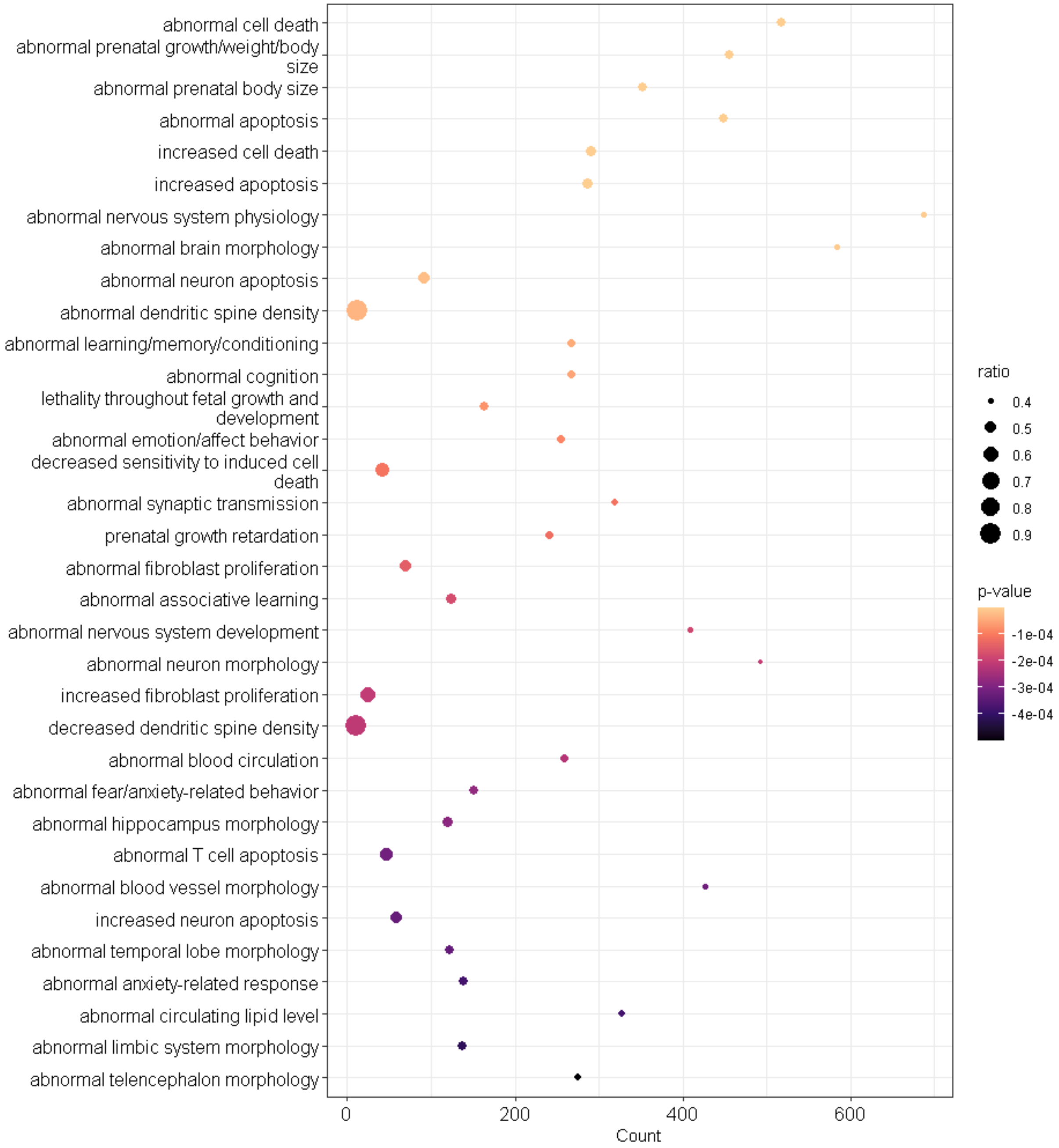

Supplemental Figure S1. Mammalian Phenotype Ontology Categories significantly enriched among genes with a significant strain x treatment interaction. Enrichment of Mammalian Phenotype Ontology (MP) was performed to identify over-represented phenotypes for our genes that showed a significant strain x treatment interaction. Numerous MP phenotypes were involved in cell death, brain morphology, behavior, and cognition. Enrichment analysis was performed using tools available at WebGestalt (www.webgestalt.org), with FDR *adjp* < 0.05. The y-axis lists significant mammalian phenotypes and the x-axis shows the number of genes that were detected in our analysis in each category. The size of the circle represents the ratio of the number of detected genes to the total number of genes in each category.
