# Supplementary material for: Effects of Genetics and Sex on Acute Gene Expression Changes in the Hippocampus Following Neonatal Ethanol Exposure in BXD Recombinant Inbred Mouse Strains
Source: Brain Sci. 2022 Nov 29;12(12):1634. doi: 10.3390/brainsci12121634 (PMC9776411; doi:10.3390/brainsci12121634)
Supplement: Supplementary file 1 [file brainsci-12-01634-s001.zip › Baker et al 2022 Supplemental Table S1.pdf]

Supplemental Table S1. Animal numbers used for microarray analyses. From each litter only 1 ethanol-exposed male, 1 ethanol-exposed female, 1 control male, and 1 control female were used (a minimum of 4 litters per treatment group, per sex). From these litters, a total of 128 samples were used—4 samples per treatment (control, ethanol), per sex (male, female), and per strain (B6, D2, BXD2, BXD48a, BXD60, BXD71, BXD73, BXD100).

Strain	Sex	Treatment	Total Number (Total Litters)
B6	Female	Control	4 (4)
B6	Female	Ethanol	4 (4)
B6	Male	Control	4 (4)
B6	Male	Ethanol	4 (4)
BXD2	Female	Control	4 (4)
BXD2	Female	Ethanol	4 (4)
BXD2	Male	Control	4 (4)
BXD2	Male	Ethanol	4 (4)
BXD48a	Female	Control	4 (4)
BXD48a	Female	Ethanol	4 (4)
BXD48a	Male	Control	4 (4)
BXD48a	Male	Ethanol	4 (4)
BXD60	Female	Control	4 (4)
BXD60	Female	Ethanol	4 (4)
BXD60	Male	Control	4 (4)
BXD60	Male	Ethanol	4 (4)
BXD71	Female	Control	4 (4)
BXD71	Female	Ethanol	4 (4)
BXD71	Male	Control	4 (4)
BXD71	Male	Ethanol	4 (4)
BXD73	Female	Control	4 (4)
BXD73	Female	Ethanol	4 (4)
BXD73	Male	Control	4 (4)
BXD73	Male	Ethanol	4 (4)
BXD100	Female	Control	4 (4)
BXD100	Female	Ethanol	4 (4)
BXD100	Male	Control	4 (4)
BXD100	Male	Ethanol	4 (4)
D2	Female	Control	4 (4)
D2	Female	Ethanol	4 (4)
D2	Male	Control	4 (4)
D2	Male	Ethanol	4 (4)
