# Supplementary material for: Effects of Genetics and Sex on Acute Gene Expression Changes in the Hippocampus Following Neonatal Ethanol Exposure in BXD Recombinant Inbred Mouse Strains
Source: Brain Sci. 2022 Nov 29;12(12):1634. doi: 10.3390/brainsci12121634 (PMC9776411; doi:10.3390/brainsci12121634)
Supplement: Supplementary file 1 [file brainsci-12-01634-s001.zip › Baker et al 2022 Supplemental Table S3.pdf]

Supplemental Table S3. Differentially expressed genes that show significant strain x treatment interaction (*adj. p* < 0.05) and fold change > 1.5.

Accession	Gene Symbol	Gene Name	*Adj p*-value	Fold Change
NR_015488	*A930003A15Rik*	RIKEN cDNA A930003A15 gene	4.29E-26	1.9
NR_033609	*A930017M01Rik*	RIKEN cDNA A930017M01 gene	4.49E-23	1.6
NM_030210	*Aacs*	acetoacetyl-CoA synthetase	3.23E-27	-1.5
NM_001166556	*Abca6*	ATP binding cassette subfamily A member 6	2.43E-15	1.6
NM_013851	*Abca8b*	ATP-binding cassette, sub-family A (ABC1), member 8b	6.79E-26	1.8
NM_178162	*Agfg2*	ArfGAP with FG repeats 2	7.23E-27	-1.7
NM_007428	*Agt*	angiotensinogen (serpin peptidase inhibitor, clade A, member 8)	7.82E-15	2.0
NM_001172146	*Aimp2*	aminoacyl tRNA synthetase complex-interacting multifunctional protein 2	2.43E-23	-1.6
NM_019764	*Amotl2*	angiomotin-like 2	1.94E-17	1.5
NM_020581	*Angptl4*	angiopoietin-like 4	6.11E-27	1.9
NM_001024851	*Ankrd34a*	ankyrin repeat domain 34A	1.84E-19	-1.6
NM_028390	*Anln*	anillin, actin binding protein	5.20E-13	1.5
NM_001109914	*Apold1*	apolipoprotein L domain containing 1	1.16E-18	1.6
NM_009705	*Arg2*	arginase type II	2.83E-16	1.5
NM_001172205	*Arid5a*	AT rich interactive domain 5A (MRF1-like)	6.65E-29	2.0
NM_023598	*Arid5b*	AT rich interactive domain 5B (MRF1-like)	1.80E-33	1.7
NM_001042591	*Arrdc3*	arrestin domain containing 3	1.48E-47	2.7
NM_001042592	*Arrdc4*	arrestin domain containing 4	1.68E-29	1.8
NM_016847	*Avpr1a*	arginine vasopressin receptor 1A	1.21E-29	-1.9
NM_001159407	*B3gnt5*	UDP-GlcNAc:betaGal beta-1,3-N-acetylglucosaminyltransferase 5	1.13E-25	1.6
NM_001284410	*Bcl2l11*	BCL2-like 11 (apoptosis facilitator)	7.31E-40	2.5
NM_007570	*Btg2*	BTG anti-proliferation factor 2	1.05E-33	1.9
NM_009770	*Btg3*	BTG anti-proliferation factor 3	2.19E-27	1.7
NM_016859	*Bysl*	bystin-like	2.35E-18	-1.5
NM_144817	*Camk1g*	calcium/calmodulin-dependent protein kinase I gamma	3.04E-28	-1.5
NM_178396	*Car12*	carbonic anhydrase 12	5.90E-06	1.6
NM_144820	*Ccdc28a*	coiled-coil domain containing 28A	1.43E-22	1.7
NM_011337	*Ccl3*	chemokine (C-C motif) ligand 3	1.47E-14	1.7
NM_007631	*Ccnd1*	cyclin D1	1.59E-24	-1.5

NM_001170395	*Cd163*	CD163 antigen	6.04E-10	1.5
NM_007646	*Cd38*	CD38 antigen	8.27E-18	1.7
NM_001111060	*Cd59a*	CD59a antigen	1.05E-13	1.5
NM_001081345	*Chd2*	chromodomain helicase DNA binding protein 2	1.32E-26	1.5
NM_001271496	*Chka*	choline kinase alpha	2.30E-13	1.6
NM_001033302	*Ciart*	circadian associated repressor of transcription	2.05E-15	-1.7
NM_001243762	*Clcn5*	chloride channel, voltage-sensitive 5	4.55E-28	1.7
NM_016674	*Cldn1*	claudin 1	1.18E-04	1.6
NM_172469	*Clic6*	chloride intracellular channel 6	4.43E-02	1.8
NM_153384	*Clrn1*	clarin 1	3.27E-19	1.5
NM_009898	*Coro1a*	coronin, actin binding protein 1A	1.63E-18	-1.5
NM_001252525	*Cpeb1*	cytoplasmic polyadenylation element binding protein 1 [*Mus musculus*	2.06E-24	1.8
NM_011957	*Creb3l1*	cAMP responsive element binding protein 3-like 1 [*Mus musculus*	6.47E-15	-1.5
NM_172728	*Creb5*	cAMP responsive element binding protein 5	1.00E-26	1.8
NM_001110850	*Crem*	cAMP responsive element modulator	2.33E-33	1.8
NM_007762	*Crhr1*	corticotropin releasing hormone receptor 1	7.82E-32	-1.9
NM_001145799	*Ctla2a*	cytotoxic T lymphocyte-associated protein 2 alpha	9.60E-17	1.5
NM_153775	*Ctu2*	cytosolic thiouridylase subunit 2	7.39E-17	-1.5
NM_009994	*Cyp1b1*	cytochrome P450, family 1, subfamily b, polypeptide 1	2.67E-23	2.0
NM_001177713	*Cyp26b1*	cytochrome P450, family 26, subfamily b, polypeptide 1	6.96E-14	-1.5
NM_028979	*Cyp2j9*	cytochrome P450, family 2, subfamily j, polypeptide 9	3.50E-28	1.8
NM_010516	*Cyr61*	cellular communication network factor 1	2.20E-18	1.5
NM_007837	*Ddit3*	DNA-damage inducible transcript 3	1.24E-15	1.5
NM_053272	*Dhcr24*	24-dehydrocholesterol reductase	3.80E-48	-1.7
NR_002854	*Dlx1as*	distal-less homeobox 1	2.48E-17	-1.6
NM_013642	*Dusp1*	dual specificity phosphatase 1	2.94E-47	2.4
NM_019819	*Dusp14*	dual specificity phosphatase 14	6.09E-16	1.5
NM_001048054	*Dusp16*	dual specificity phosphatase 16	6.68E-35	2.0
NM_176933	*Dusp4*	dual specificity phosphatase 4	9.31E-19	-1.5
NM_026268	*Dusp6*	dual specificity phosphatase 6	7.13E-26	-1.6
NM_153459	*Dusp7*	dual specificity phosphatase 7	1.11E-16	-1.5

NM_008748	*Dusp8*	dual specificity phosphatase 8	5.63E-41	1.5
NM_010104	*Edn1*	endothelin 1	9.30E-23	2.1
NM_001289925	*Egr3*	early growth response 3	6.61E-34	-2.1
NM_018781	*Egr3*	early growth response 3	3.46E-15	-1.5
NM_175522	*Elfn1*	leucine rich repeat and fibronectin type III, extracellular 1	1.53E-16	-1.5
NM_008815	*Etv4*	ets variant 4	6.10E-27	-1.6
NM_023794	*Etv5*	ets variant 5	3.01E-30	-1.7
NM_183187	*Fam107a*	family with sequence similarity 107, member A	6.75E-42	3.3
NM_153574	*Fam13a*	family with sequence similarity 13, member A	5.34E-17	1.5
NM_178908	*Fam26e*	calcium homeostasis modulator family member 5	3.31E-36	2.0
NM_175104	*Fam53c*	family with sequence similarity 53, member C	1.05E-27	1.7
NM_011812	*Fbln5*	fibulin 5	3.39E-02	1.5
NM_026346	*Fbxo32*	F-box protein 32	1.33E-14	1.5
NM_010191	*Fdft1*	farnesyl diphosphate farnesyl transferase 1	2.21E-42	-1.5
NM_001253751	*Fdps*	farnesyl diphosphate synthetase	2.05E-23	-1.5
NM_010197	*Fgf1*	fibroblast growth factor 1	1.61E-13	1.5
NM_001163215	*Fgfr3*	fibroblast growth factor receptor 3	2.78E-24	1.6
NM_001164259	*Fgfrl1*	fibroblast growth factor receptor-like 1	5.65E-24	-1.5
NM_001159706	*Folh1*	folate hydrolase 1	4.16E-26	1.9
NM_011817	*Gadd45g*	growth arrest and DNA-damage-inducible 45 gamma	3.55E-16	1.5
NM_028022	*Gatsl3*	cytosolic arginine sensor for mTORC1 subunit 1	2.97E-19	1.7
NM_001010937	*Gjb6*	gap junction protein, beta 6	1.87E-11	1.9
NM_029102	*Glt8d2*	glycosyltransferase 8 domain containing 2	1.77E-22	1.8
NM_001110809	*Gpatch4*	G patch domain containing 4	1.70E-20	-1.5
NM_027518	*Gpr137c*	G protein-coupled receptor 137C	1.33E-46	2.1
NM_010338	*Gpr37*	G protein-coupled receptor 37	2.49E-27	1.6
NM_145066	*Gpr85*	G protein-coupled receptor 85	6.20E-26	1.5
NM_153419	*Grwd1*	glutamate-rich WD repeat containing 1	1.08E-21	-1.6
NM_008216	*Has2*	hyaluronan synthase 2	6.21E-16	-1.7
NM_198962	*Hcrtr2*	hypocretin (orexin) receptor 2	7.02E-22	-1.6
NM_144835	*Heatr1*	HEAT repeat containing 1	2.73E-26	-1.6
NM_175256	*Heg1*	heart development protein with EGF like domains 1	6.46E-21	1.5

NM_001162950	*Hif3a*	hypoxia inducible factor 3, alpha subunit	2.61E-17	1.7
NM_008252	*Hmgb2*	high mobility group box 2	1.16E-18	1.5
NM_008255	*Hmgcr*	3-hydroxy-3-methylglutaryl-Coenzyme A reductase	1.14E-33	-1.6
NM_008256	*Hmgcs2*	3-hydroxy-3-methylglutaryl-Coenzyme A synthase 2	1.58E-27	2.1
NM_007545	*Hrk*	harakiri, BCL2 interacting protein (contains only BH3 domain)	9.28E-20	1.5
NM_010476	*Hsd17b7*	hydroxysteroid (17-beta) dehydrogenase 7	2.95E-38	-1.7
NM_019564	*Htra1*	HtrA serine peptidase 1	2.19E-30	1.7
NM_015790	*Icosl*	icos ligand	1.00E-17	1.5
NM_010495	*Id1*	inhibitor of DNA binding 1, HLH protein	4.58E-17	1.5
NM_031166	*Id4*	inhibitor of DNA binding 4	1.30E-14	1.6
NM_145360	*Idi1*	isopentenyl-diphosphate delta isomerase	1.85E-48	-2.2
NM_001101605	*Ifit1bl1*	interferon induced protein with tetratricpeptide repeats 1B like 1	6.77E-16	1.6
NM_013562	*Ifrd1*	interferon-related developmental regulator 1	5.04E-24	-1.6
NM_172439	*Inpp5j*	inositol polyphosphate 5-phosphatase J	7.71E-25	1.7
NM_153526	*Insig1*	insulin induced gene 1	1.44E-42	-2.2
NM_016851	*Irf6*	interferon regulatory factor 6	5.41E-19	1.5
NM_010591	*Jun*	Jun proto-oncogene, AP-1 transcription factor subunit	3.20E-60	3.2
NM_001286944	*Jund*	JunD proto-oncogene, AP-1 transcription factor subunit	1.05E-34	1.7
NM_001081134	*Kcng1*	potassium voltage-gated channel, subfamily G, member 1	7.56E-30	-1.8
NM_001110227	*Kcnj13*	potassium inwardly-rectifying channel, subfamily J, member 13	2.69E-02	2.0
NR_045177	*Kctd16*	potassium channel tetramerisation domain containing 16	1.43E-27	1.6
NM_026135	*Kctd16*	potassium channel tetramerisation domain containing 16	9.88E-21	1.5
NM_078477	*Klf16*	Kruppel-like factor 16	6.82E-34	-1.8
NM_001252658	*Ldlr*	low density lipoprotein receptor	2.07E-37	-2.3
NM_001083125	*Lhx6*	LIM homeobox protein 6	3.47E-15	-1.5
NM_028894	*Lonrf3*	LON peptidase N-terminal domain and ring finger 3	1.79E-14	1.5
NM_181470	*Ltv1*	LON peptidase N-terminal domain and ring finger 3	3.80E-18	-1.5
NM_001271416	*Ly6a*	lymphocyte antigen 6 complex, locus A	1.40E-13	1.5

NM_001252055	*Ly6c1*	lymphocyte antigen 6 complex, locus C1	3.71E-24	2.0
NM_001171187	*Mal*	myelin and lymphocyte protein, T cell differentiation protein	3.92E-17	1.5
NM_011737	*Map3k19*	mitogen-activated protein kinase kinase kinase 19	6.66E-09	1.5
NM_008563	*Mcm3*	minichromosome maintenance complex component 3	1.28E-16	-1.5
NM_001012335	*Mdk*	midkine	4.22E-14	1.5
NM_008587	*Mertk*	MER proto-oncogene tyrosine kinase	1.83E-26	1.9
NM_001163833	*Msl3l2*	MSL3 like 2	2.40E-15	1.5
NM_025436	*Msmo1*	methylsterol monoxygenase 1	5.20E-43	-2.3
NM_013602	*Mt1*	metallothionein 1	4.56E-34	1.6
NM_008630	*Mt2*	metallothionein 2	1.98E-43	2.7
NM_138656	*Mvd*	mevalonate diphosphate decarboxylase	6.34E-38	-2.1
NM_001008542	*Mxi1*	MAX interactor 1, dimerization protein	1.63E-46	1.8
NM_178728	*Napepld*	N-acyl phosphatidylethanolamine phospholipase D	2.98E-14	1.6
NM_022565	*Ndst4*	N-deacetylase/N-sulfotransferase (heparin glucosaminyl) 4	2.63E-17	1.5
NM_028995	*Nipal3*	NIPA-like domain containing 3	6.65E-23	1.6
NM_024193	*Nop56*	NOP56 ribonucleoprotein	5.39E-23	-1.5
NM_010342	*Npbwr1*	neuropeptides B/W receptor 1	1.85E-20	1.5
NM_178644	*Oaf*	out at first homolog	5.42E-16	1.6
NM_011859	*Osr1*	odd-skipped related transcription factor 1	1.91E-05	1.5
NM_001286481	*Otx2*	orthodenticle homeobox 2	3.05E-02	1.7
NM_001008497	*P2ry14*	purinergic receptor P2Y, G-protein coupled, 14	1.22E-11	1.5
NM_153594	*Pcmtd2*	protein-L-isoaspartate (D-aspartate) O-methyltransferase domain containing 2	3.50E-25	1.6
NM_016861	*Pdlim1*	PDZ and LIM domain 1 (elfin)	6.76E-17	1.7
NM_001159367	*Per1*	period circadian clock 1	4.91E-24	1.5
NM_178149	*Pik3ip1*	phosphoinositide-3-kinase interacting protein 1	5.02E-37	2.1
NM_001024955	*Pik3r1*	phosphoinositide-3-kinase regulatory subunit 1	2.81E-52	1.8
NM_145478	*Pim3*	proviral integration site 3	1.05E-13	-1.5
NM_008872	*Plat*	plasminogen activator, tissue	1.50E-19	1.5
NM_152813	*Plcd3*	phospholipase C, delta 3	4.03E-28	1.7
NM_001033253	*Plekhg1*	pleckstrin homology domain containing, family G (with RhoGef domain) member 1	1.02E-38	2.0

NM_013807	*Plk3*	polo like kinase 3	1.70E-31	-1.7
NM_001164630	*Pwwp3b*	PWWP domain containing 3B	7.21E-14	1.5
NM_029494	*Rab30*	RAB30, member RAS oncogene family	4.38E-44	1.9
NM_001099624	*Rapgef2*	Rap guanine nucleotide exchange factor 2	4.60E-46	1.7
NM_001252494	*Rapgef6*	Rap guanine nucleotide exchange factor (GEF) 6	8.64E-46	2.1
NM_009062	*Rgs4*	regulator of G-protein signaling 4	1.98E-23	1.5
NM_153514	*Rhobtb2*	Rho related BTB domain containing 2	3.77E-24	-1.5
NM_172612	*Rnd1*	Rho family GTPase 1	1.45E-21	-1.5
NM_001166553	*Rnf145*	ring finger protein 145	1.01E-33	-1.5
NM_133982	*Rpp25*	ribonuclease P/MRP 25 subunit	4.57E-22	-1.5
NM_146244	*Rps6kl1*	ribosomal protein S6 kinase-like 1	1.01E-33	1.8
NM_011521	*Sdc4*	syndecan 4	6.79E-33	2.2
NM_030261	*Sesn3*	sestrin 3	4.25E-32	1.5
NM_016687	*Sfrp4*	secreted frizzled related protein 4	1.00E-15	1.6
NM_001168525	*Sgms1*	sphingomyelin synthase 1	8.19E-14	1.5
NM_028943	*Sgms2*	sphingomyelin synthase 2	3.96E-05	1.5
NM_027921	*Slc16a14*	solute carrier family 16 (monocarboxylic acid transporters), member 14	3.51E-27	1.5
NM_001029842	*Slc16a6*	solute carrier family 16 (monocarboxylic acid transporters), member 6	1.28E-23	1.8
NM_025807	*Slc16a9*	solute carrier family 16 (monocarboxylic acid transporters), member 9	2.35E-07	1.5
NM_011400	*Slc2a1*	solute carrier family 2 (facilitated glucose transporter), member 1	4.41E-28	1.7
NM_178934	*Slc2a12*	solute carrier family 2 (facilitated glucose transporter), member 12	3.84E-08	1.9
NM_001199283	*Slc43a2*	solute carrier family 43, member 2	9.51E-26	1.5
NM_001081263	*Slc44a5*	solute carrier family 44, member 5	4.36E-38	1.8
NM_139142	*Slc6a20a*	solute carrier family 6 (neurotransmitter transporter), member 20A	9.75E-06	1.6
NM_001038643	*Slco3a1*	solute carrier organic anion transporter family, member 3a1	3.95E-27	-1.5
NM_134133	*Smim3*	small integral membrane protein 3	4.01E-16	1.6
NM_182927	*Spred3*	sprouty-related EVH1 domain containing 3	1.66E-31	-2.0
NM_011898	*Spry4*	sprouty RTK signaling antagonist 4	3.04E-31	-1.9
NM_009270	*Sqle*	squalene epoxidase	2.68E-34	-1.6
NM_011358	*Srsf2*	serine and arginine rich splicing factor 2	3.51E-30	-1.5

NM_001195485	*Srsf7*	serine and arginine-rich splicing factor 7	2.69E-27	-1.6
NM_133774	*Stard4*	StAR-related lipid transfer (START) domain containing 4	8.50E-33	-1.8
NM_027399	*Steap1*	six transmembrane epithelial antigen of the prostate 1	1.31E-03	1.5
NM_019675	*Stmn4*	stathmin-like 4	2.23E-22	1.5
NM_013515	*Stom*	stomatin	2.98E-13	1.5
NM_133670	*Sult1a1*	sulfotransferase family 1A, phenol-preferring, member 1	1.42E-13	1.7
NM_017465	*Sult2b1*	sulfotransferase family, cytosolic, 2B, member 1	2.44E-11	1.5
NM_001040085	*Sytl2*	synaptotagmin-like 2	1.16E-28	1.8
NM_001082976	*Tc2n*	tandem C2 domains, nuclear	3.01E-03	1.6
NM_009368	*Tgfb3*	transforming growth factor, beta 3	3.69E-14	1.6
NM_174989	*Ticam1*	toll-like receptor adaptor molecule 1	2.03E-22	1.8
NM_133211	*Tlr7*	toll-like receptor 7	1.72E-17	1.6
NM_001160385	*Tmem196*	transmembrane protein 196	1.01E-18	1.5
NM_177344	*Tmem203*	transmembrane protein 203	5.70E-18	-1.5
NM_133706	*Tmem97*	transmembrane protein 97	1.57E-23	-1.6
NM_001170855	*Trim36*	tripartite motif-containing 36	2.58E-14	-1.5
NM_001170912	*Trim66*	tripartite motif containing 66	1.00E-15	1.6
NM_012035	*Trpc7*	transient receptor potential cation channel subfamily C member 7	1.27E-13	-1.5
NM_001009935	*Txnip*	thioredoxin interacting protein	1.51E-43	3.8
NM_001169576	*Ube2h*	ubiquitin conjugating enzyme E2 H	5.01E-33	1.5
NM_175158	*Utp20*	UTP20 small subunit processome component	3.61E-18	-1.5
NM_001039385	*Vgf*	VGF nerve growth factor inducible	1.37E-26	-1.9
NM_172372	*Wdr45*	WD repeat domain 45	5.48E-20	1.5
NM_020603	*Wdr46*	WD repeat domain 46	8.66E-18	-1.5
NM_001005342	*Ypel4*	yippee like 4	2.16E-34	2.4
NM_027166	*Ypel5*	yippee like 5	3.80E-34	1.5
NM_001033324	*Zbtb16*	zinc finger and BTB domain containing 16	3.70E-16	1.5
NM_001110309	*Zfp426*	zinc finger protein 426	3.11E-26	1.5
NM_001005425	*Zfp663*	zinc finger protein 663	3.60E-12	1.6
NM_001252584	*Zmynd8*	zinc finger MYND-type containing 8	3.03E-23	1.5
