# Supplementary material for: Effects of Genetics and Sex on Acute Gene Expression Changes in the Hippocampus Following Neonatal Ethanol Exposure in BXD Recombinant Inbred Mouse Strains
Source: Brain Sci. 2022 Nov 29;12(12):1634. doi: 10.3390/brainsci12121634 (PMC9776411; doi:10.3390/brainsci12121634)
Supplement: Supplementary file 1 [file brainsci-12-01634-s001.zip › Baker et al Supplemental Table S2.pdf]

Supplemental Table S2. Significant (*adjp* < 0.05) ethanol-induced gene expression changes within each strain. NS = not significant.

Accession	Gene Symbol	Strain	*Adjp*-value Male	Fold Change Male	*Adjp*-value Female	Fold Change Female
NM_021446	*0610007P14Rik*	B6	NS	NS	2.13E-03	-1.4
NM_001081095	*1700009N14Rik*	B6	3.37E-02	1.5	NS	NS
NM_198630	*1810024B03Rik*	B6	NS	NS	1.99E-02	1.7
NM_001167925	*A730017C20Rik*	B6	2.44E-02	1.2	NS	NS
NR_015488	*A930003A15Rik*	B6	3.85E-06	2.9	NS	NS
NR_033609	*A930017M01Rik*	B6	2.68E-02	1.6	NS	NS
NM_030210	*Aacs*	B6	NS	NS	7.86E-03	-1.6
NM_013851	*Abca8b*	B6	5.88E-03	2.1	2.53E-02	1.8
NM_178162	*Agfg2*	B6	6.51E-03	-1.9	3.85E-04	-2.1
NM_001172146	*Aimp2*	B6	NS	NS	4.35E-02	-1.6
NM_001035532	*Akap2*	B6	NS	NS	2.51E-02	1.5
NM_017476	*Akap8l*	B6	2.26E-02	1.4	NS	NS
NM_009658	*Akr1b3*	B6	NS	NS	2.49E-02	-1.4
NM_011786	*Aloxe3*	B6	4.19E-02	2.1	NS	NS
NM_020581	*Angptl4*	B6	NS	NS	6.29E-04	2.3
NM_001024851	*Ankrd34a*	B6	NS	NS	1.26E-02	-1.9
NM_001109914	*Apold1*	B6	NS	NS	3.67E-02	1.8
NM_001172205	*Arid5a*	B6	1.60E-02	2.0	3.23E-03	2.1
NM_023598	*Arid5b*	B6	2.80E-04	1.8	6.10E-05	1.8
NM_207231	*Arl5c*	B6	4.09E-02	1.9	NS	NS
NM_001042591	*Arrdc3*	B6	1.64E-08	3.0	9.51E-08	2.8
NM_001042592	*Arrdc4*	B6	4.40E-04	2.0	8.56E-04	1.9
NM_026855	*Arv1*	B6	NS	NS	2.67E-02	-1.7
NM_026217	*Atg12*	B6	8.41E-03	1.4	NS	NS
NM_001199304	*Atxn1*	B6	5.49E-03	1.5	NS	NS
NM_001080930	*Atxn1l*	B6	NS	NS	2.63E-02	1.5
NM_016847	*Avpr1a*	B6	3.58E-02	-1.7	NS	NS
NM_020025	*B3galt2*	B6	1.40E-02	1.6	3.49E-02	1.5
NM_001159407	*B3gnt5*	B6	NS	NS	4.03E-02	1.6
NM_019835	*B4galt5*	B6	NS	NS	3.59E-02	-1.4
NM_001001182	*Baz2b*	B6	4.01E-02	1.4	4.63E-02	1.4
NM_175325	*Bbs4*	B6	1.00E-05	1.6	5.88E-03	1.4
NM_001001332	*BC117090*	B6	NS	NS	4.63E-02	-1.9

NM_001284410	*Bcl2l11*	B6	6.55E-05	2.5	2.57E-04	2.3
NM_001122683	*Bdh1*	B6	4.83E-02	-1.3	NS	NS
NM_172506	*Boc*	B6	NS	NS	2.67E-02	-1.6
NM_133889	*Bsdc1*	B6	1.04E-03	1.5	6.71E-05	1.5
NM_007570	*Btg2*	B6	7.06E-05	2.1	1.50E-03	1.9
NM_009770	*Btg3*	B6	7.06E-03	1.8	NS	NS
ENSMUST00000180393	*C130032M10Rik*	B6	NS	NS	1.27E-02	-1.6
NM_026192	*Calcoco1*	B6	NS	NS	1.01E-02	1.5
NM_144817	*Camk1g*	B6	3.13E-03	-1.5	2.07E-05	-1.8
NM_025451	*Camk2n1*	B6	1.05E-02	1.5	NS	NS
NM_007625	*Cbx4*	B6	NS	NS	8.01E-03	-1.5
NM_144820	*Ccdc28a*	B6	4.60E-04	2.3	1.01E-02	1.9
NM_007631	*Ccnd1*	B6	8.73E-03	-1.6	4.40E-04	-1.7
NM_009831	*Ccng1*	B6	4.83E-02	1.3	NS	NS
NM_001111060	*Cd59a*	B6	NS	NS	3.12E-02	2.0
NM_001081345	*Chd2*	B6	2.49E-02	1.5	1.50E-03	1.6
NM_139134	*Chodl*	B6	NS	NS	4.83E-02	-1.9
NM_001289429	*Cipc*	B6	NS	NS	1.30E-03	-1.5
NM_025854	*Cir1*	B6	9.68E-03	1.5	NS	NS
NM_001243762	*Clcn5*	B6	4.35E-02	1.6	1.93E-02	1.6
NM_009904	*Clgn*	B6	NS	NS	4.98E-02	1.5
NM_026977	*Cnppd1*	B6	1.78E-02	1.5	NS	NS
NM_008779	*Cntn3*	B6	1.89E-02	1.6	NS	NS
NM_001039710	*Coq10b*	B6	NS	NS	4.97E-02	-1.5
NM_001252525	*Cpeb1*	B6	1.31E-02	2.0	NS	NS
NM_001177379	*Cpeb2*	B6	NS	NS	1.99E-02	1.5
NM_172728	*Creb5*	B6	NS	NS	1.88E-03	2.1
NM_001110850	*Crem*	B6	1.73E-02	1.7	1.62E-02	1.7
NM_007762	*Crhr1*	B6	NS	NS	1.45E-03	-2.0
NM_001113330	*Crx*	B6	1.17E-02	2.4	NS	NS
NM_001082573	*Crygc*	B6	NS	NS	4.98E-02	1.4
NM_001085448	*Ctnnd1*	B6	NS	NS	1.98E-02	1.4
NM_153775	*Ctu2*	B6	NS	NS	1.73E-02	-1.7
NM_007808	*Cycs*	B6	NS	NS	2.45E-02	-1.2
NM_009994	*Cyp1b1*	B6	NS	NS	1.73E-02	2.2
NM_028979	*Cyp2j9*	B6	6.88E-04	2.1	NS	NS
NM_020010	*Cyp51*	B6	2.13E-03	-1.5	6.52E-03	-1.4

NM_010516	*Cyr61*	B6	NS	NS	2.67E-02	1.7
NM_001165980	*Dcaf17*	B6	4.63E-02	1.4	NS	NS
NM_019553	*Ddx21*	B6	NS	NS	3.51E-03	-1.6
NM_001252457	*Ddx39b*	B6	1.31E-02	-1.4	NS	NS
NM_001282055	*Ddx46*	B6	NS	NS	4.11E-02	-1.5
NM_010030	*Defb2*	B6	NS	NS	3.59E-02	2.1
NM_053272	*Dhcr24*	B6	9.19E-07	-1.6	6.67E-08	-1.7
NR_002854	*Dlx1as*	B6	NS	NS	2.24E-03	-2.2
NM_025926	*Dnajb4*	B6	NS	NS	2.72E-03	1.5
NM_001163026	*Dnajc13*	B6	NS	NS	4.11E-02	-1.4
NM_007882	*Dsc3*	B6	3.66E-02	3.7	NS	NS
NM_001285807	*Dtna*	B6	4.65E-02	1.3	9.46E-03	1.3
NM_013642	*Dusp1*	B6	8.59E-08	2.4	4.94E-10	2.8
NM_001048054	*Dusp16*	B6	8.38E-04	2.0	1.13E-04	2.2
NM_176933	*Dusp4*	B6	NS	NS	2.51E-02	-1.7
NM_001085390	*Dusp5*	B6	NS	NS	2.25E-02	-1.7
NM_026268	*Dusp6*	B6	1.54E-02	-1.6	NS	NS
NM_008748	*Dusp8*	B6	4.31E-08	1.7	7.08E-04	1.4
NM_026932	*Ebna1bp2*	B6	NS	NS	4.56E-02	-1.4
NM_001110331	*Eci2*	B6	NS	NS	2.41E-02	1.4
NM_010104	*Edn1*	B6	NS	NS	1.56E-03	2.9
NM_001289925	*Egr3*	B6	6.55E-05	-2.3	2.33E-06	-2.6
NM_001289927	*Egr3*	B6	NS	NS	2.64E-02	-1.8
NM_018781	*Egr3*	B6	NS	NS	4.36E-02	-1.7
NM_010121	*Eif2ak3*	B6	NS	NS	2.67E-02	1.5
NM_001253692	*Elmod3*	B6	3.90E-02	1.5	4.18E-02	1.4
NM_175313	*Eogt*	B6	NS	NS	9.80E-03	1.6
NM_023612	*Esm1*	B6	NS	NS	1.01E-02	2.0
NM_008815	*Etv4*	B6	2.69E-03	-1.7	5.02E-05	-1.9
NM_023794	*Etv5*	B6	4.50E-04	-1.9	2.66E-04	-1.9
NM_007970	*Ezh1*	B6	4.01E-02	1.4	NS	NS
NM_010171	*F3*	B6	NS	NS	1.15E-02	1.6
NM_001163567	*Fam102b*	B6	1.88E-02	1.3	NS	NS
NM_183187	*Fam107a*	B6	7.06E-03	2.3	1.25E-05	3.3
NM_025582	*Fam213b*	B6	NS	NS	3.22E-02	-1.6
NM_178908	*Fam26e*	B6	3.72E-04	2.0	5.13E-05	2.1
NM_175104	*Fam53c*	B6	1.14E-02	1.8	NS	NS
NM_178797	*Far2*	B6	NS	NS	3.12E-02	-1.4

NM_028149	*Fbxl20*	B6	3.55E-02	1.4	NS	NS
NM_010191	*Fdft1*	B6	4.50E-04	-1.4	1.20E-05	-1.5
NM_008003	*Fgf15*	B6	4.25E-02	-1.8	NS	NS
NM_001164259	*Fgfrl1*	B6	4.14E-02	-1.5	NS	NS
NM_022009	*Flii*	B6	NS	NS	2.55E-02	-1.5
NM_001081416	*Fndc1*	B6	1.97E-02	1.5	NS	NS
NM_001159706	*Folh1*	B6	8.41E-03	2.1	6.92E-04	2.4
NM_001163282	*Gdf1*	B6	NS	NS	2.97E-02	-2.0
NM_029102	*Glt8d2*	B6	1.80E-03	2.4	NS	NS
ENSMUST00000092165	*Gm10271*	B6	NS	NS	3.41E-02	2.5
uc029usq.1	*Gm13308*	B6	1.99E-02	1.5	NS	NS
ENSMUST00000112772	*Gm3443*	B6	NS	NS	4.11E-02	2.1
NM_027518	*Gpr137c*	B6	9.01E-09	2.3	5.27E-07	2.0
NM_001167694	*Gpr19*	B6	NS	NS	5.88E-03	1.6
NM_010338	*Gpr37*	B6	1.01E-02	1.7	4.05E-03	1.8
NM_175490	*Gpr75*	B6	4.77E-02	1.4	NS	NS
NM_145066	*Gpr85*	B6	1.05E-02	1.5	NS	NS
NM_001114333	*Grm1*	B6	1.99E-02	1.5	NS	NS
NM_198962	*Hcrtr2*	B6	NS	NS	4.50E-02	-1.6
NM_144835	*Heatr1*	B6	1.68E-02	-1.7	5.62E-04	-1.9
NM_175256	*Heg1*	B6	NS	NS	2.30E-03	1.8
NM_001162950	*Hif3a*	B6	NS	NS	4.18E-02	2.0
NM_025812	*Hmg20a*	B6	NS	NS	2.96E-02	1.4
NM_008252	*Hmgb2*	B6	NS	NS	3.70E-02	1.6
NM_008255	*Hmgcr*	B6	2.32E-02	-1.5	2.10E-03	-1.6
NM_008256	*Hmgcs2*	B6	NS	NS	3.23E-03	2.4
NM_016805	*Hnrnpu*	B6	4.09E-02	-1.3	NS	NS
NM_053262	*Hsd17b11*	B6	2.13E-03	1.6	4.71E-02	1.4
NM_010476	*Hsd17b7*	B6	3.55E-03	-1.6	2.69E-05	-1.8
NM_019564	*Htra1*	B6	4.40E-02	1.6	1.27E-02	1.7
NM_010495	*Id1*	B6	NS	NS	3.41E-02	1.7
NM_145360	*Idi1*	B6	2.41E-07	-2.1	2.32E-09	-2.3
NM_013562	*Ifrd1*	B6	4.06E-02	-1.6	5.88E-03	-1.7
NM_008384	*Inpp1*	B6	3.47E-02	1.7	NS	NS
NM_153526	*Insig1*	B6	7.79E-06	-2.2	9.29E-07	-2.3
NM_010591	*Jun*	B6	2.29E-15	3.7	1.01E-15	3.7
NM_001286944	*Jund*	B6	1.05E-05	1.9	1.30E-03	1.6

NM_001039347	*Kcnd3*	B6	NS	NS	4.03E-02	-1.5
NM_001081134	*Kcng1*	B6	NS	NS	1.73E-02	-1.7
NM_011317	*Khdrbs1*	B6	1.37E-02	-1.5	NS	NS
NM_010636	*Klf12*	B6	NS	NS	4.37E-02	1.3
NM_078477	*Klf16*	B6	8.58E-03	-1.7	5.13E-05	-1.9
NM_001081403	*Klhl14*	B6	NS	NS	1.25E-02	-2.5
NM_029436	*Klhl24*	B6	1.40E-02	1.4	1.52E-02	1.4
NM_010655	*Kpna2*	B6	6.39E-04	-1.6	3.49E-02	-1.4
NM_172787	*L3mbtl3*	B6	4.17E-02	1.4	NS	NS
NM_001252658	*Ldlr*	B6	1.93E-04	-2.3	5.95E-07	-2.9
NM_001199043	*Lgals8*	B6	4.56E-02	1.4	NS	NS
NM_181074	*Lingo1*	B6	NS	NS	2.69E-02	-1.3
NM_001013758	*Lingo3*	B6	NS	NS	1.16E-02	-1.6
NM_001146048	*Lrrc1*	B6	NS	NS	4.23E-02	1.7
NM_181470	*Ltv1*	B6	NS	NS	2.67E-02	-1.7
NM_001252055	*Ly6c1*	B6	4.09E-02	2.0	NS	NS
NM_053201	*Magee1*	B6	NS	NS	3.59E-02	-1.4
NM_011948	*Map3k4*	B6	NS	NS	4.83E-02	1.5
NM_001045483	*Mapk1ip1*	B6	1.95E-02	1.4	NS	NS
NM_011841	*Mapk7*	B6	NS	NS	5.74E-03	-1.5
NM_133350	*Mapre3*	B6	NS	NS	2.70E-02	-1.4
NM_175171	*Mast4*	B6	NS	NS	1.04E-03	1.5
NM_008563	*Mcm3*	B6	NS	NS	3.49E-02	-1.7
NM_026896	*Med27*	B6	NS	NS	1.90E-02	-1.4
NM_001001979	*Megf10*	B6	NS	NS	8.10E-04	1.8
NM_008587	*Mertk*	B6	1.31E-02	2.1	1.45E-02	2.0
NM_026197	*Mettl16*	B6	NS	NS	2.66E-02	-1.6
NM_173870	*Mgat4a*	B6	NS	NS	2.80E-03	1.7
NM_023799	*Mgea5*	B6	1.47E-02	1.3	2.03E-02	1.3
NM_001166635	*Mid1ip1*	B6	NS	NS	4.04E-02	-1.4
NM_021565	*Midn*	B6	3.66E-02	-1.4	4.37E-02	-1.4
NM_053164	*Mrpl43*	B6	NS	NS	3.03E-02	-1.5
NM_025436	*Msmo1*	B6	1.29E-05	-2.2	2.33E-06	-2.3
NM_013602	*Mt1*	B6	NS	NS	2.98E-03	1.6
NM_008630	*Mt2*	B6	1.09E-02	1.9	3.05E-05	2.5
NM_001128601	*Mthfsl*	B6	1.41E-02	1.5	NS	NS
NM_138656	*Mvd*	B6	1.93E-02	-1.7	6.45E-05	-2.1
NM_001008542	*Mxi1*	B6	2.78E-08	1.9	6.67E-08	1.9

NM_001171615	*Myt1*	B6	NS	NS	4.43E-02	1.5
NM_172722	*Naa25*	B6	NS	NS	1.01E-02	-1.6
NM_026742	*Ndufaf4*	B6	NS	NS	1.16E-03	-1.5
NM_001111324	*Nedd9*	B6	3.06E-02	1.5	NS	NS
NM_028995	*Nipal3*	B6	4.45E-02	1.6	6.69E-03	1.7
NM_028024	*Nkiras2*	B6	3.53E-02	1.4	NS	NS
NM_021303	*Noc2l*	B6	NS	NS	4.97E-03	-1.5
NM_009834	*Noct*	B6	2.62E-02	-1.5	NS	NS
NM_001008421	*Nol10*	B6	NS	NS	1.90E-02	-1.6
NM_138747	*Nop2*	B6	3.08E-02	-1.4	2.03E-03	-1.5
NM_024193	*Nop56*	B6	9.68E-03	-1.6	4.18E-02	-1.5
NM_018868	*Nop58*	B6	NS	NS	2.03E-02	-1.4
NM_001163684	*Nosip*	B6	4.32E-02	1.5	NS	NS
NM_010342	*Npbwr1*	B6	7.58E-04	1.9	2.59E-03	1.8
NM_016789	*Nptx2*	B6	NS	NS	1.98E-02	-1.8
NM_010941	*Nsdhl*	B6	NS	NS	1.73E-02	-1.4
NM_016968	*Olig1*	B6	1.93E-02	-1.5	3.12E-02	-1.4
NM_152818	*Osbp2*	B6	NS	NS	2.64E-02	1.4
NM_001134791	*Osbpl9*	B6	1.05E-02	1.3	1.32E-04	1.5
NM_001285839	*Osgepl1*	B6	2.88E-02	1.6	NS	NS
NM_146169	*Paip2b*	B6	NS	NS	3.32E-02	1.5
NM_146129	*Pcif1*	B6	NS	NS	4.48E-02	-1.5
NM_183028	*Pcmtd1*	B6	NS	NS	2.76E-02	1.3
NM_153594	*Pcmtd2*	B6	1.18E-02	1.7	1.29E-02	1.7
NM_001177980	*Pde4b*	B6	1.05E-02	1.4	NS	NS
NM_008808	*Pdgfa*	B6	NS	NS	1.42E-02	-1.5
NM_001159367	*Per1*	B6	2.32E-02	1.6	NS	NS
NM_030064	*Phf23*	B6	NS	NS	3.64E-02	-1.5
NM_178149	*Pik3ip1*	B6	1.07E-06	2.5	6.45E-05	2.2
NM_001024955	*Pik3r1*	B6	9.01E-09	1.8	7.75E-13	2.1
NM_145478	*Pim3*	B6	NS	NS	4.05E-03	-2.1
NM_152813	*Plcd3*	B6	1.10E-02	1.8	2.86E-04	2.0
NM_001033253	*Plekhg1*	B6	9.02E-05	2.0	1.39E-04	1.9
NM_013807	*Plk3*	B6	NS	NS	3.41E-04	-1.9
NM_133249	*Ppargc1b*	B6	NS	NS	4.63E-03	-1.8
NM_177782	*Prex1*	B6	NS	NS	4.56E-02	-1.4
NM_177698	*Psd3*	B6	9.35E-03	1.4	NS	NS
NM_029494	*Rab30*	B6	3.85E-06	1.9	1.14E-06	1.9

NM_001038621	*Rabgap1l*	B6	6.45E-05	1.5	4.58E-03	1.4
NM_001099624	*Rapgef2*	B6	3.62E-05	1.6	1.52E-08	1.8
NM_001252494	*Rapgef6*	B6	9.01E-09	2.4	3.88E-07	2.2
NM_001176528	*Rara*	B6	NS	NS	9.80E-03	-1.6
NM_009025	*Rasa3*	B6	NS	NS	1.56E-03	-1.7
NM_028234	*Rbm33*	B6	6.23E-03	1.5	NS	NS
NM_016846	*Rgl1*	B6	2.20E-02	1.4	NS	NS
NM_153514	*Rhobtb2*	B6	NS	NS	4.84E-06	-2.0
NM_182929	*Rims3*	B6	NS	NS	4.15E-02	-1.6
NM_172612	*Rnd1*	B6	NS	NS	4.84E-04	-1.8
NM_001166553	*Rnf145*	B6	NS	NS	6.92E-04	-1.5
NM_194346	*Rnf31*	B6	NS	NS	2.19E-03	-1.8
NM_183263	*Rnmtl1*	B6	NS	NS	2.34E-02	-1.6
NM_001289921	*Rorb*	B6	2.64E-02	1.6	NS	NS
NM_001043354	*Rorb*	B6	4.56E-02	1.7	NS	NS
NM_001163461	*Rpap2*	B6	3.43E-02	1.5	NS	NS
NM_146244	*Rps6kl1*	B6	6.33E-07	2.4	7.97E-04	1.9
NM_001136227	*Rtkn*	B6	NS	NS	5.88E-03	1.7
NM_172769	*Sc5d*	B6	1.31E-02	-1.3	NS	NS
NM_011521	*Sdc4*	B6	1.71E-02	2.0	6.43E-04	2.3
NM_030261	*Sesn3*	B6	NS	NS	8.01E-03	1.4
NM_001286538	*Sigmar1*	B6	NS	NS	4.47E-04	-1.6
NM_027921	*Slc16a14*	B6	1.88E-02	1.5	9.64E-04	1.7
NM_001029842	*Slc16a6*	B6	NS	NS	4.09E-02	1.9
NM_080853	*Slc17a6*	B6	4.06E-02	1.6	NS	NS
NM_001159593	*Slc20a1*	B6	1.10E-02	-1.4	1.60E-04	-1.6
NM_011400	*Slc2a1*	B6	8.73E-03	1.8	7.88E-03	1.7
NM_172479	*Slc38a5*	B6	NS	NS	9.84E-03	-1.6
NM_001199283	*Slc43a2*	B6	1.80E-03	1.7	4.30E-02	1.5
NM_001081263	*Slc44a5*	B6	2.13E-03	1.6	2.07E-05	1.8
NM_029643	*Slc52a2*	B6	NS	NS	7.26E-03	-1.6
NM_001038643	*Slco3a1*	B6	2.46E-02	-1.4	3.38E-03	-1.5
NM_008539	*Smad1*	B6	3.08E-02	1.4	NS	NS
NM_133697	*Smim14*	B6	NS	NS	3.12E-02	1.3
NM_001177833	*Smox*	B6	2.98E-03	1.6	NS	NS
NM_009224	*Snrnp70*	B6	NS	NS	1.80E-02	-1.4
NM_182927	*Spred3*	B6	1.10E-02	-1.9	1.50E-03	-2.1
NM_011898	*Spry4*	B6	1.95E-02	-1.8	2.80E-03	-1.9

NM_009270	*Sqle*	B6	1.95E-02	-1.5	1.90E-03	-1.6
NM_009272	*Srm*	B6	1.70E-02	-1.4	4.78E-03	-1.5
NM_011358	*Srsf2*	B6	6.74E-03	-1.5	5.74E-03	-1.5
NM_001195485	*Srsf7*	B6	2.03E-02	-1.6	1.01E-02	-1.6
NM_011374	*St8sia1*	B6	NS	NS	5.75E-04	1.6
NM_133774	*Stard4*	B6	2.39E-02	-1.7	2.30E-03	-1.8
NM_019675	*Stmn4*	B6	NS	NS	3.38E-03	1.7
NM_153563	*Strip1*	B6	NS	NS	3.59E-02	-1.3
NM_153399	*Syne1*	B6	NS	NS	1.15E-03	1.7
NM_001040085	*Sytl2*	B6	6.82E-03	1.9	NS	NS
NM_013763	*Tbl2*	B6	2.46E-03	-1.6	NS	NS
NM_145396	*Tbl3*	B6	NS	NS	3.36E-02	-1.6
NM_009381	*Thrsp*	B6	NS	NS	1.73E-02	1.7
NM_174989	*Ticam1*	B6	NS	NS	1.52E-02	2.0
NM_026617	*Tmbim4*	B6	NS	NS	2.41E-02	1.3
NM_172476	*Tmc7*	B6	1.99E-02	1.5	NS	NS
NM_175564	*Tmem169*	B6	3.13E-02	1.6	NS	NS
NM_001160385	*Tmem196*	B6	3.63E-02	1.7	NS	NS
NM_029384	*Tmem238*	B6	NS	NS	2.42E-02	-1.6
NM_133706	*Tmem97*	B6	NS	NS	6.78E-04	-2.0
NM_001161746	*Tnfrsf12a*	B6	NS	NS	2.25E-02	1.8
NM_001048267	*Tnpo1*	B6	NS	NS	7.78E-03	1.4
NM_144812	*Tnrc6b*	B6	3.87E-02	1.5	NS	NS
NM_001109748	*Tomm40*	B6	6.67E-03	-1.4	2.64E-02	-1.4
NM_001039466	*Trim46*	B6	NS	NS	1.73E-02	-1.5
NM_001170912	*Trim66*	B6	1.95E-02	2.2	NS	NS
NM_001127233	*Trp53*	B6	NS	NS	4.23E-02	-1.4
NM_021884	*Tsg101*	B6	3.66E-02	-1.3	NS	NS
NM_001252588	*Tspan4*	B6	NS	NS	2.59E-02	-1.5
NM_001009935	*Txnip*	B6	8.05E-07	4.1	2.55E-10	6.0
NM_001015876	*Tyw1*	B6	4.56E-02	1.6	NS	NS
NM_001163769	*U2af1*	B6	2.64E-02	-1.5	NS	NS
NM_001169576	*Ube2h*	B6	8.73E-03	1.5	2.61E-05	1.7
NM_001145162	*Ube2ql1*	B6	NS	NS	4.18E-02	-1.3
NM_030724	*Uck2*	B6	3.66E-02	-1.3	4.63E-03	-1.4
ENSMUST00000053686	*Uck2*	B6	NS	NS	9.19E-03	-1.7
NM_009484	*Uty*	B6	NS	NS	4.11E-02	-2.0

NM_001039385	*Vgf*	B6	NS	NS	1.01E-02	-2.0
NM_001110015	*Wdr36*	B6	NS	NS	7.89E-03	-1.5
NM_172372	*Wdr45*	B6	2.39E-02	1.7	NS	NS
NM_001005342	*Ypel4*	B6	1.57E-02	2.1	1.22E-04	2.6
NM_027166	*Ypel5*	B6	4.50E-04	1.5	3.43E-05	1.6
NM_028106	*Zbed3*	B6	1.68E-02	1.5	NS	NS
NM_001115130	*Zbtb44*	B6	1.18E-02	1.4	NS	NS
NM_178404	*Zc3h6*	B6	5.61E-04	1.7	NS	NS
NM_001110309	*Zfp426*	B6	6.74E-03	1.6	5.88E-03	1.5
NM_207255	*Zfp532*	B6	NS	NS	4.78E-02	1.4
NM_001267583	*Zfp811*	B6	NS	NS	5.12E-03	1.6
NM_001167944	*Zkscan5*	B6	NS	NS	3.59E-02	1.7
NM_001252584	*Zmynd8*	B6	NS	NS	1.01E-02	1.6
NM_001009818	*-44450*	D2	2.56E-02	-1.2	1.86E-02	-1.3
NM_001113486	*-44448*	D2	1.17E-04	-1.4	NS	NS
NM_001033262	*-44264*	D2	1.56E-05	-2.2	NS	NS
NM_001166372	*-44256*	D2	NS	NS	1.87E-02	1.4
NM_021446	*0610007P14Rik*	D2	3.53E-02	-1.3	NS	NS
NM_027056	*1700012P22Rik*	D2	2.50E-03	2.3	NS	NS
NM_144953	*1700019D03Rik*	D2	1.69E-02	1.6	NS	NS
NM_029693	*1700123K08Rik*	D2	NS	NS	4.03E-02	1.6
NM_001163145	*1810041L15Rik*	D2	1.02E-02	-1.5	NS	NS
NR_045177	*2900055J20Rik*	D2	1.07E-03	1.7	2.47E-03	1.7
NM_029113	*4930447A16Rik*	D2	2.79E-02	1.9	NS	NS
NM_029444	*4930447C04Rik*	D2	5.89E-03	2.3	NS	NS
NM_001166394	*4931428F04Rik*	D2	1.28E-02	-1.5	NS	NS
NM_173771	*4933406M09Rik*	D2	2.76E-02	1.7	NS	NS
NM_001167925	*A730017C20Rik*	D2	9.53E-04	1.3	1.53E-02	1.2
NR_015488	*A930003A15Rik*	D2	1.44E-03	2.1	7.41E-03	2.0
NR_033609	*A930017M01Rik*	D2	5.79E-03	1.7	NS	NS
NM_030210	*Aacs*	D2	2.26E-06	-1.9	6.06E-03	-1.6
NM_001128091	*Aadacl2*	D2	NS	NS	1.94E-02	1.8
NM_013851	*Abca8b*	D2	3.01E-03	2.0	8.50E-03	1.9
NM_015751	*Abce1*	D2	1.14E-02	-1.3	1.88E-02	-1.3
NM_134130	*Abhd3*	D2	3.10E-03	1.8	NS	NS
NM_133360	*Acaca*	D2	6.18E-03	-1.4	NS	NS
NM_178799	*Acad12*	D2	2.33E-02	1.7	NS	NS
NM_144784	*Acat1*	D2	2.33E-02	-1.2	NS	NS

NM_009338	*Acat2*	D2	7.00E-05	-1.7	1.98E-03	-1.6
NM_153151	*Acat3*	D2	3.74E-04	-1.6	1.67E-02	-1.4
NM_133225	*Acbd3*	D2	4.93E-02	-1.3	NS	NS
NM_001199296	*Acly*	D2	2.82E-05	-1.6	2.35E-02	-1.3
NM_025790	*Acot13*	D2	2.86E-02	1.3	NS	NS
NM_001146057	*Acot7*	D2	1.17E-02	-1.3	NS	NS
NM_001205385	*Actr3*	D2	3.26E-02	-1.2	NS	NS
NM_001270996	*Adam9*	D2	1.57E-03	1.4	NS	NS
NM_172845	*Adamts4*	D2	4.88E-02	1.5	NS	NS
NM_001100606	*Adat3*	D2	1.21E-02	-1.6	NS	NS
NM_001163290	*Adck3*	D2	NS	NS	1.26E-02	1.7
NM_009623	*Adcy8*	D2	4.48E-02	-1.6	NS	NS
NM_182928	*Adm2*	D2	3.55E-02	1.6	NS	NS
NM_019822	*Adrm1*	D2	2.62E-02	-1.4	NS	NS
NM_001162939	*Aen*	D2	NS	NS	3.03E-02	-1.6
NM_027373	*Afap1*	D2	4.79E-02	-1.3	NS	NS
NM_178162	*Agfg2*	D2	2.13E-03	-1.9	1.24E-03	-2.0
NM_026644	*Agpat4*	D2	3.36E-02	1.3	NS	NS
NM_007428	*Agt*	D2	3.99E-02	2.5	NS	NS
NM_146155	*Ahdc1*	D2	8.43E-03	-1.8	NS	NS
NM_001199330	*AI987944*	D2	6.92E-04	1.6	NS	NS
NM_001172146	*Aimp2*	D2	5.42E-04	-1.9	5.51E-03	-1.8
NM_001177602	*Ak4*	D2	1.90E-03	-1.7	NS	NS
NM_017476	*Akap8l*	D2	9.61E-04	1.5	NS	NS
NM_009658	*Akr1b3*	D2	7.37E-03	-1.4	1.58E-02	-1.4
NM_001165894	*Akt1*	D2	2.85E-03	-1.4	NS	NS
NM_001276446	*Alad*	D2	7.37E-03	-1.6	NS	NS
NM_009657	*Aldoc*	D2	4.49E-02	1.3	NS	NS
NM_133981	*Alg9*	D2	3.79E-02	-1.3	NS	NS
NM_025538	*Alkbh7*	D2	1.76E-04	-1.7	4.38E-03	-1.6
NM_001081395	*Amotl1*	D2	2.70E-02	1.7	NS	NS
NM_019764	*Amotl2*	D2	NS	NS	1.15E-02	1.8
NM_001276301	*Ampd3*	D2	7.58E-04	1.9	NS	NS
NM_028333	*Angptl1*	D2	1.33E-02	1.9	NS	NS
NM_020581	*Angptl4*	D2	4.10E-02	1.7	2.45E-04	2.4
NM_001024851	*Ankrd34a*	D2	1.39E-04	-2.3	2.62E-02	-1.8
NM_001128103	*Ano3*	D2	1.65E-03	2.2	NS	NS
NM_001164679	*Ano8*	D2	3.83E-02	-1.4	NS	NS

NM_009680	*Ap3b1*	D2	4.42E-03	1.3	NS	NS
NM_007460	*Ap3d1*	D2	1.08E-02	-1.3	NS	NS
NM_007461	*Apba2*	D2	6.18E-03	-1.6	NS	NS
NM_001109914	*Apold1*	D2	8.93E-03	1.8	NS	NS
NM_007481	*Arf6*	D2	6.80E-03	-1.3	1.20E-02	-1.3
NM_021493	*Arhgap23*	D2	3.05E-02	-1.5	NS	NS
NM_001289686	*Arhgef3*	D2	2.12E-03	1.5	NS	NS
NM_001172205	*Arid5a*	D2	6.09E-03	2.0	2.81E-03	2.1
NM_023598	*Arid5b*	D2	1.83E-03	1.6	7.23E-03	1.6
NM_001025102	*Arl14ep*	D2	5.85E-03	1.3	NS	NS
NM_207231	*Arl5c*	D2	4.39E-02	1.7	NS	NS
NM_001042591	*Arrdc3*	D2	4.62E-10	3.2	3.11E-08	2.9
NM_001042592	*Arrdc4*	D2	7.05E-03	1.7	1.62E-03	1.9
NM_026855	*Arv1*	D2	NS	NS	3.07E-02	-1.6
NM_007492	*Arx*	D2	4.15E-04	-2.0	NS	NS
NM_133346	*Asb6*	D2	3.54E-02	-1.5	NS	NS
NM_001287180	*Atf4*	D2	6.89E-04	-1.6	5.51E-03	-1.5
NM_001081304	*Atf6*	D2	1.80E-03	1.5	NS	NS
NM_017406	*Atf6b*	D2	1.37E-02	-1.4	NS	NS
NM_025770	*Atg10*	D2	3.21E-02	1.5	NS	NS
NM_026217	*Atg12*	D2	7.69E-03	1.3	1.90E-02	1.3
NM_001145879	*Atp6ap1l*	D2	4.78E-02	2.0	NS	NS
NM_027855	*Atraid*	D2	6.11E-03	1.4	NS	NS
NM_001199304	*Atxn1*	D2	4.86E-03	1.4	NS	NS
NM_009125	*Atxn2*	D2	4.77E-02	-1.3	NS	NS
NM_016847	*Avpr1a*	D2	3.46E-07	-2.7	2.95E-05	-2.3
NM_194347	*AY358078*	D2	NS	NS	4.15E-02	-1.7
NM_001122993	*B3galt5*	D2	7.84E-03	1.6	1.88E-02	1.6
NM_029792	*B3gat1*	D2	7.60E-03	-1.5	NS	NS
NM_024256	*B3gat3*	D2	5.62E-03	-1.5	NS	NS
NM_001169114	*B3gnt2*	D2	1.54E-02	1.6	NS	NS
NM_001159407	*B3gnt5*	D2	2.13E-03	1.7	1.18E-02	1.6
ENSMUST00000181156	*B430202K04Rik*	D2	4.42E-02	1.5	NS	NS
NM_001244617	*B4galnt1*	D2	1.01E-02	-1.5	NS	NS
NM_177897	*B4galnt4*	D2	1.11E-02	-1.5	NS	NS
NM_019835	*B4galt5*	D2	1.96E-04	-1.6	3.69E-02	-1.4
NM_001045523	*Bahd1*	D2	4.36E-02	-1.5	NS	NS

NM_001038231	*Banf1*	D2	2.31E-02	-1.4	NS	NS
NM_027395	*Basp1*	D2	3.88E-02	-1.4	NS	NS
NM_001001182	*Baz2b*	D2	7.73E-03	1.5	NS	NS
NM_175325	*Bbs4*	D2	1.92E-03	1.4	NS	NS
NM_027444	*Bbx*	D2	1.01E-02	1.7	NS	NS
NM_153161	*BC037034*	D2	NS	NS	3.43E-02	-1.4
NM_001164369	*Bcas1*	D2	2.01E-02	2.3	NS	NS
NM_007534	*Bcl2a1b*	D2	NS	NS	3.57E-02	1.8
NM_001289716	*Bcl2l1*	D2	2.15E-02	-1.4	NS	NS
NM_001284410	*Bcl2l11*	D2	1.86E-05	2.5	8.20E-05	2.4
NM_029933	*Bcl9*	D2	5.00E-03	-1.3	NS	NS
NM_001122683	*Bdh1*	D2	7.56E-05	-1.5	1.51E-03	-1.5
NM_001141922	*Bean1*	D2	2.67E-02	-1.4	NS	NS
NM_009751	*Bfsp1*	D2	2.25E-02	-2.0	NS	NS
NM_021560	*Bhlhe22*	D2	1.29E-02	-1.7	NS	NS
NM_173404	*Bmp3*	D2	1.11E-02	-1.7	NS	NS
NM_013481	*Bop1*	D2	5.40E-03	-1.6	NS	NS
NM_026396	*Brix1*	D2	2.82E-02	-1.4	NS	NS
NM_133889	*Bsdc1*	D2	NS	NS	2.81E-03	1.4
NM_007570	*Btg2*	D2	8.65E-08	2.6	4.88E-06	2.3
NM_009770	*Btg3*	D2	2.76E-02	1.6	3.35E-03	1.9
NM_016859	*Bysl*	D2	9.03E-04	-1.8	NS	NS
NM_007573	*C1qbp*	D2	1.93E-03	-1.4	NS	NS
NM_183175	*C1qtnf9*	D2	3.88E-02	1.5	NS	NS
NM_009779	*C3ar1*	D2	9.79E-03	1.8	NS	NS
NM_001252533	*Cacnb2*	D2	9.79E-03	1.4	NS	NS
NM_019431	*Cacng4*	D2	4.48E-02	-1.3	NS	NS
NM_001025600	*Cadm1*	D2	2.67E-02	-1.5	NS	NS
NM_009788	*Calb1*	D2	NS	NS	7.23E-03	-1.7
NM_026192	*Calcoco1*	D2	4.08E-02	1.4	NS	NS
NM_144817	*Camk1g*	D2	3.57E-04	-1.6	2.95E-05	-1.7
NM_001163749	*Camsap3*	D2	7.21E-04	-1.6	NS	NS
NM_001111289	*Caprin1*	D2	2.82E-02	-1.3	NS	NS
NM_025821	*Carhsp1*	D2	5.31E-03	1.7	NS	NS
NM_021531	*Carm1*	D2	7.37E-03	-1.3	NS	NS
NM_027937	*Caskin1*	D2	2.15E-02	-1.4	NS	NS
NM_001159344	*Casz1*	D2	2.62E-02	-1.6	NS	NS
NM_019626	*Cbln1*	D2	3.48E-02	-2.0	NS	NS

NM_172633	*Cbln2*	D2	1.29E-02	-1.9	NS	NS
NM_173047	*Cbr3*	D2	2.05E-02	-1.5	6.49E-03	-1.6
NM_001271353	*Cbs*	D2	2.46E-02	1.4	NS	NS
NM_007625	*Cbx4*	D2	7.70E-03	-1.4	NS	NS
NM_173763	*Ccbl2*	D2	1.13E-02	2.0	NS	NS
NM_207202	*Ccdc120*	D2	2.94E-02	-1.5	NS	NS
NM_001025576	*Ccdc141*	D2	2.04E-02	1.9	NS	NS
NM_177716	*Ccdc184*	D2	1.72E-06	-2.0	NS	NS
NM_144820	*Ccdc28a*	D2	NS	NS	2.56E-03	2.1
NM_025602	*Ccdc59*	D2	NS	NS	3.03E-02	-1.6
NM_207268	*Ccdc87*	D2	2.12E-03	-2.1	NS	NS
NM_011338	*Ccl9*	D2	3.31E-02	1.9	NS	NS
NM_007631	*Ccnd1*	D2	8.16E-04	-1.6	2.81E-03	-1.6
NM_009831	*Ccng1*	D2	2.30E-03	1.4	NS	NS
NM_001114328	*Ccpg1*	D2	9.00E-03	1.5	NS	NS
NM_153098	*Cd109*	D2	2.34E-02	1.7	NS	NS
NM_026956	*Cd209f*	D2	4.41E-02	-2.0	NS	NS
NM_009846	*Cd24a*	D2	3.84E-02	-1.6	NS	NS
NM_001111058	*Cd33*	D2	3.91E-02	1.8	NS	NS
NM_007646	*Cd38*	D2	1.61E-02	1.9	NS	NS
NM_013488	*Cd4*	D2	1.23E-02	3.1	NS	NS
NM_025584	*Cd99*	D2	2.28E-03	-1.8	NS	NS
NM_026772	*Cdc42ep2*	D2	9.75E-04	-1.8	NS	NS
NM_009865	*Cdh10*	D2	NS	NS	3.06E-02	-1.4
NM_001168304	*Cdk19*	D2	1.14E-02	1.4	NS	NS
NM_009872	*Cdk5r2*	D2	1.35E-04	-1.9	NS	NS
NM_009874	*Cdk7*	D2	NS	NS	2.84E-02	-1.5
NM_183294	*Cdkl1*	D2	4.08E-02	1.4	NS	NS
NM_001080929	*Cdr2l*	D2	2.33E-02	-1.7	NS	NS
NM_026014	*Cdt1*	D2	4.91E-02	-1.5	NS	NS
NM_001134426	*Cdv3*	D2	5.30E-03	-1.3	1.93E-02	-1.3
NM_001039185	*Ceacam1*	D2	2.17E-02	1.9	NS	NS
NM_001159364	*Cep97*	D2	4.25E-02	1.5	NS	NS
NM_009891	*Chat*	D2	5.95E-03	1.5	NS	NS
NM_133928	*Chchd4*	D2	1.49E-02	-1.7	NS	NS
NM_001081345	*Chd2*	D2	1.30E-03	1.6	NS	NS
NM_021350	*Chml*	D2	2.82E-02	-1.5	NS	NS
NM_001114385	*Chrdl1*	D2	5.72E-03	-1.7	NS	NS

NM_029935	*Chst15*	D2	3.43E-02	-1.4	NS	NS
NM_001289429	*Cipc*	D2	1.30E-02	-1.3	NS	NS
NM_016680	*Clasrp*	D2	NS	NS	2.17E-02	-1.5
NM_001177770	*Clcc1*	D2	8.97E-03	1.7	NS	NS
NM_001243762	*Clcn5*	D2	1.20E-03	1.7	4.08E-02	1.5
NM_001160096	*Cldn10*	D2	8.82E-03	1.6	NS	NS
NM_001033175	*Cln6*	D2	9.60E-03	1.5	NS	NS
NM_153384	*Clrn1*	D2	NS	NS	2.62E-03	1.9
NM_025647	*Cmpk1*	D2	4.98E-02	-1.3	NS	NS
NM_026066	*Cmtm5*	D2	4.19E-02	1.3	NS	NS
NM_177751	*Cnksr2*	D2	2.49E-02	1.4	NS	NS
NM_007726	*Cnr1*	D2	1.61E-02	-1.4	NS	NS
NM_130457	*Cntnap4*	D2	4.61E-03	-1.6	NS	NS
NM_001198835	*Coch*	D2	1.16E-03	-2.0	NS	NS
NM_146211	*Colgalt1*	D2	4.61E-02	-1.4	NS	NS
NM_026965	*Comtd1*	D2	3.73E-02	-1.3	NS	NS
NM_001039710	*Coq10b*	D2	2.99E-02	-1.5	NS	NS
NM_009898	*Coro1a*	D2	1.11E-02	-1.7	2.75E-02	-1.6
NM_178379	*Cox10*	D2	1.16E-02	-1.5	NS	NS
NM_001033310	*Cox18*	D2	7.57E-03	-1.4	NS	NS
NM_001252525	*Cpeb1*	D2	5.99E-04	2.2	1.62E-03	2.2
NM_016856	*Cpsf2*	D2	NS	NS	4.85E-02	-1.3
NM_013499	*Cr1l*	D2	2.07E-02	1.4	NS	NS
NM_007758	*Cr2*	D2	4.55E-02	-1.7	NS	NS
NM_011957	*Creb3l1*	D2	8.40E-03	-1.9	2.82E-02	-1.9
NM_172728	*Creb5*	D2	3.98E-03	1.9	1.33E-04	2.3
NM_001110850	*Crem*	D2	2.39E-05	2.1	2.95E-05	2.1
NM_007762	*Crhr1*	D2	9.18E-09	-2.9	3.85E-03	-1.9
NM_024223	*Crip2*	D2	3.14E-02	-1.4	NS	NS
NM_016669	*Crym*	D2	1.19E-04	-1.6	NS	NS
NM_023565	*Cse1l*	D2	3.04E-03	-1.4	4.61E-02	-1.3
NM_001037859	*Csf1r*	D2	1.96E-02	1.5	NS	NS
NM_007783	*Csk*	D2	4.55E-02	-1.5	NS	NS
NM_153407	*Csrnp2*	D2	1.67E-02	-1.5	NS	NS
NM_153409	*Csrnp3*	D2	2.66E-02	1.3	NS	NS
NM_007791	*Csrp1*	D2	1.83E-02	1.6	NS	NS
NM_177655	*Cstl1*	D2	1.63E-02	1.8	NS	NS
NM_001170744	*Ctbp2*	D2	2.94E-02	-1.4	NS	NS

NM_001145799	*Ctla2a*	D2	3.89E-02	1.6	NS	NS
NM_031251	*Ctns*	D2	NS	NS	6.49E-03	1.5
NM_016748	*Ctps*	D2	9.21E-03	-1.5	NS	NS
NM_007799	*Ctse*	D2	1.69E-02	1.7	NS	NS
NM_019861	*Ctsf*	D2	2.28E-02	1.4	NS	NS
NM_001163332	*Cttnbp2nl*	D2	3.16E-02	-1.4	NS	NS
NM_153775	*Ctu2*	D2	1.54E-04	-2.1	NS	NS
NM_029402	*Cul2*	D2	1.42E-02	-1.4	NS	NS
NM_009987	*Cx3cr1*	D2	3.42E-02	1.4	NS	NS
NM_009911	*Cxcr4*	D2	1.40E-02	-1.7	1.51E-02	-1.7
NM_133687	*Cxxc5*	D2	4.18E-02	-1.5	NS	NS
NM_025567	*Cyc1*	D2	3.39E-02	-1.4	NS	NS
NM_010008	*Cyp2j6*	D2	1.20E-03	1.6	NS	NS
NM_028979	*Cyp2j9*	D2	9.98E-05	2.1	4.30E-03	1.9
NM_130882	*Cyp4f13*	D2	2.40E-02	1.6	NS	NS
NM_001204333	*Cyp4f14*	D2	3.55E-02	1.7	NS	NS
NM_020010	*Cyp51*	D2	1.30E-06	-1.7	3.46E-06	-1.7
NM_010516	*Cyr61*	D2	5.50E-04	1.9	NS	NS
NM_001004455	*Cys1*	D2	NS	NS	2.75E-02	1.7
NM_001163548	*Cyth3*	D2	3.14E-02	-1.3	NS	NS
NM_194061	*D630045J12Rik*	D2	1.34E-02	-1.5	NS	NS
NM_001190473	*Dapk3*	D2	2.67E-02	-1.4	NS	NS
NM_001177371	*Dbn1*	D2	2.50E-02	-1.4	NS	NS
NM_001146308	*Dbnl*	D2	7.01E-03	-1.5	NS	NS
NM_153555	*Dcaf8*	D2	2.27E-02	1.3	NS	NS
NM_025600	*Dda1*	D2	2.90E-02	-1.2	NS	NS
NM_007837	*Ddit3*	D2	NS	NS	2.95E-02	1.7
NM_025860	*Ddx18*	D2	4.76E-02	-1.4	NS	NS
NM_019553	*Ddx21*	D2	9.59E-03	-1.5	NS	NS
NM_197982	*Ddx39*	D2	3.99E-02	-1.7	2.90E-02	-1.8
NM_001252457	*Ddx39b*	D2	1.48E-02	-1.3	NS	NS
NM_001282055	*Ddx46*	D2	6.22E-03	-1.5	3.17E-03	-1.6
NM_053183	*Ddx50*	D2	1.24E-02	-1.4	5.42E-03	-1.5
NM_027156	*Ddx51*	D2	4.01E-02	-1.7	NS	NS
NM_028041	*Ddx54*	D2	3.04E-04	-1.7	NS	NS
NM_011933	*Decr2*	D2	2.36E-02	1.4	3.03E-02	1.4
NM_007853	*Degs1*	D2	7.47E-03	1.4	NS	NS
NM_010046	*Dgat1*	D2	4.27E-02	-1.7	NS	NS

NM_053272	*Dhcr24*	D2	2.86E-11	-1.9	9.51E-11	-1.9
NM_026819	*Dhrs1*	D2	9.21E-03	1.4	NS	NS
NM_172594	*Dhx29*	D2	4.36E-02	-1.3	NS	NS
NM_001252682	*Dhx30*	D2	7.37E-03	-1.4	NS	NS
NM_026191	*Dhx40*	D2	3.71E-02	1.4	NS	NS
NM_007842	*Dhx9*	D2	NS	NS	7.86E-05	-1.5
NM_020265	*Dkk2*	D2	NS	NS	1.83E-02	-1.9
NM_001042487	*Dlgap4*	D2	1.34E-02	-1.4	NS	NS
NM_001286013	*Dlk2*	D2	3.52E-02	-1.6	NS	NS
NM_007865	*Dll1*	D2	1.75E-02	-1.6	NS	NS
NR_002854	*Dlx1as*	D2	3.03E-03	-2.1	4.10E-03	-2.1
NM_010058	*Dmwd*	D2	2.10E-02	-1.4	NS	NS
NM_018808	*Dnajb1*	D2	NS	NS	4.47E-02	-1.4
NM_025926	*Dnajb4*	D2	1.89E-04	1.5	3.78E-03	1.5
NM_001099738	*Dnajc28*	D2	8.07E-03	1.8	NS	NS
NM_152915	*Dner*	D2	9.09E-03	-1.3	NS	NS
NM_001199431	*Dnmt1*	D2	1.11E-02	-1.6	NS	NS
NM_053246	*Dok4*	D2	NS	NS	4.67E-02	-1.3
NM_020329	*Dolpp1*	D2	3.21E-02	-1.6	NS	NS
NM_172639	*Dpcd*	D2	3.05E-02	-1.4	NS	NS
NM_013874	*Dpf1*	D2	6.92E-04	-2.0	8.30E-03	-1.8
NM_023047	*Dpysl5*	D2	1.97E-02	-1.3	NS	NS
NM_023842	*Dsp*	D2	2.54E-02	1.8	NS	NS
NM_001285807	*Dtna*	D2	7.83E-06	1.5	2.58E-04	1.4
NM_008052	*Dtx1*	D2	1.11E-02	-1.4	NS	NS
NM_172442	*Dtx4*	D2	NS	NS	9.41E-03	1.4
NM_013642	*Dusp1*	D2	1.82E-05	2.0	6.36E-07	2.3
NM_023173	*Dusp12*	D2	2.19E-04	-1.8	NS	NS
NM_019819	*Dusp14*	D2	2.66E-02	1.6	NS	NS
NM_001048054	*Dusp16*	D2	5.42E-04	1.9	3.59E-03	1.9
NM_176933	*Dusp4*	D2	1.64E-07	-2.5	1.58E-03	-1.9
NM_001085390	*Dusp5*	D2	2.76E-02	-1.6	NS	NS
NM_026268	*Dusp6*	D2	1.01E-04	-1.9	2.57E-04	-1.9
NM_153459	*Dusp7*	D2	4.31E-04	-2.0	NS	NS
NM_008748	*Dusp8*	D2	4.14E-05	1.5	1.67E-06	1.6
NM_019682	*Dynll1*	D2	4.13E-02	-1.3	NS	NS
NM_001037957	*Dyrk1b*	D2	2.53E-02	1.3	NS	NS
NM_177856	*E130311K13Rik*	D2	3.97E-02	1.6	NS	NS

NM_148952	*E2f4*	D2	1.05E-02	-1.6	NS	NS
NM_007893	*E4f1*	D2	NS	NS	2.76E-02	-1.5
NM_025380	*Eef1e1*	D2	6.11E-03	-1.4	3.43E-02	-1.4
NM_026007	*Eef1g*	D2	1.71E-02	-1.3	NS	NS
NM_007909	*Efna2*	D2	7.01E-03	-1.6	NS	NS
NM_010108	*Efna3*	D2	1.13E-04	-1.6	NS	NS
NM_010111	*Efnb2*	D2	4.26E-02	-1.4	NS	NS
NM_007911	*Efnb3*	D2	4.70E-04	-1.6	NS	NS
NM_001082483	*Efr3b*	D2	2.69E-03	-1.4	NS	NS
NM_053208	*Egln2*	D2	1.31E-02	-1.4	NS	NS
NM_001289925	*Egr3*	D2	2.38E-05	-2.3	1.12E-07	-2.9
NM_018781	*Egr3*	D2	NS	NS	5.98E-03	-1.9
NM_026892	*Eif1b*	D2	4.23E-02	-1.3	NS	NS
NM_010121	*Eif2ak3*	D2	3.91E-02	1.4	1.00E-02	1.6
NM_018749	*Eif3d*	D2	3.51E-02	-1.4	2.75E-02	-1.4
NM_001159375	*Eif4a1*	D2	2.94E-02	-1.3	NS	NS
NM_138669	*Eif4a3*	D2	NS	NS	1.64E-02	-1.4
NM_007917	*Eif4e*	D2	NS	NS	2.03E-02	-1.3
NM_145941	*Eif4g1*	D2	1.51E-02	-1.3	4.45E-03	-1.3
NM_177586	*Eif5a2*	D2	NS	NS	3.71E-02	-1.4
NM_023479	*Elac2*	D2	4.74E-02	-1.4	NS	NS
NM_001177883	*Elavl2*	D2	4.49E-04	-1.5	NS	NS
NM_175522	*Elfn1*	D2	7.05E-03	-1.8	1.35E-02	-1.8
NM_001253692	*Elmod3*	D2	2.67E-02	1.4	NS	NS
NM_130450	*Elovl6*	D2	7.73E-03	-1.4	2.47E-03	-1.5
NM_198299	*Enkd1*	D2	2.96E-02	-1.5	NS	NS
NM_001168620	*Enpp5*	D2	5.50E-04	1.5	NS	NS
NM_177304	*Enpp6*	D2	3.97E-02	2.5	NS	NS
NM_175313	*Eogt*	D2	1.21E-02	1.5	NS	NS
NM_013512	*Epb41l4a*	D2	2.28E-03	-1.5	NS	NS
NM_134065	*Epdr1*	D2	3.04E-03	1.6	NS	NS
NM_010140	*Epha3*	D2	2.49E-02	-1.4	NS	NS
NM_010143	*Ephb3*	D2	4.17E-02	-1.6	NS	NS
NM_175266	*Epm2aip1*	D2	4.10E-02	-1.3	NS	NS
NM_001005868	*Erbb2ip*	D2	5.24E-03	1.5	NS	NS
NM_028042	*Ercc8*	D2	1.23E-03	-1.8	NS	NS
NM_133659	*Erg*	D2	NS	NS	4.15E-02	-1.6
NM_028039	*Esco2*	D2	NS	NS	1.90E-02	-2.0

NM_029250	*Etnk1*	D2	3.14E-02	1.3	NS	NS
NM_001038642	*Ets1*	D2	NS	NS	3.72E-02	-1.5
NM_008815	*Etv4*	D2	2.28E-05	-1.9	4.97E-04	-1.8
NM_023794	*Etv5*	D2	1.31E-05	-2.1	5.98E-03	-1.7
NM_145570	*Eva1a*	D2	9.21E-03	1.6	NS	NS
NM_177077	*Exoc6b*	D2	2.25E-02	-1.4	NS	NS
NM_175399	*Exosc4*	D2	1.24E-02	-1.7	NS	NS
NM_028274	*Exosc6*	D2	3.52E-02	-1.2	NS	NS
NM_001163514	*Extl2*	D2	8.09E-03	1.5	NS	NS
NM_027980	*Faap100*	D2	1.75E-02	-1.4	NS	NS
NM_183187	*Fam107a*	D2	2.75E-14	7.8	2.20E-09	4.8
NM_001085513	*Fam131c*	D2	4.46E-06	-2.3	NS	NS
NM_001034851	*Fam134b*	D2	2.39E-05	1.6	3.72E-02	1.3
NM_153574	*Fam13a*	D2	1.24E-02	1.7	NS	NS
NM_001159743	*Fam150b*	D2	NS	NS	4.44E-02	-1.8
NM_177838	*Fam163a*	D2	1.90E-03	-1.6	4.62E-02	-1.5
NM_001081161	*Fam171a1*	D2	1.51E-03	-1.6	NS	NS
NM_182808	*Fam19a1*	D2	1.39E-04	1.7	NS	NS
NM_030565	*Fam20c*	D2	3.31E-04	-1.7	NS	NS
NM_001113283	*Fam214a*	D2	NS	NS	1.88E-02	1.6
NM_178908	*Fam26e*	D2	3.63E-06	2.3	7.61E-06	2.3
NM_175104	*Fam53c*	D2	1.20E-03	1.9	2.05E-02	1.7
NM_001160261	*Fam78b*	D2	3.82E-02	-1.4	NS	NS
NM_029007	*Fam84a*	D2	7.37E-03	-1.5	NS	NS
NM_026635	*Fam96a*	D2	4.31E-02	1.4	NS	NS
NM_178797	*Far2*	D2	3.01E-06	-1.7	2.58E-04	-1.6
NM_007988	*Fasn*	D2	5.68E-03	-1.5	NS	NS
NM_007991	*Fbl*	D2	4.54E-02	-1.3	NS	NS
NM_001161851	*Fbxo44*	D2	NS	NS	2.88E-02	-1.5
NM_176982	*Fbxo48*	D2	4.31E-03	-2.0	NS	NS
NM_172591	*Fcho2*	D2	2.10E-02	1.4	NS	NS
NM_010191	*Fdft1*	D2	9.18E-09	-1.7	1.67E-06	-1.6
NM_001253751	*Fdps*	D2	5.95E-05	-1.9	2.81E-03	-1.7
NM_001039824	*Fdx1l*	D2	1.01E-02	-1.5	NS	NS
NM_010197	*Fgf1*	D2	NS	NS	4.42E-02	1.8
NM_010201	*Fgf14*	D2	1.42E-02	1.5	NS	NS
NM_008005	*Fgf18*	D2	6.22E-03	-1.7	NS	NS
NM_001277268	*Fgf5*	D2	2.52E-02	1.5	NS	NS

NM_001163215	*Fgfr3*	D2	3.31E-04	1.9	1.87E-02	1.6
NM_001164259	*Fgfrl1*	D2	3.83E-03	-1.6	3.21E-03	-1.7
NM_008013	*Fgl2*	D2	1.63E-02	1.5	NS	NS
NM_001081243	*Filip1*	D2	3.18E-02	1.6	NS	NS
NM_008019	*Fkbp1a*	D2	2.21E-02	-1.4	NS	NS
NM_010219	*Fkbp4*	D2	3.22E-02	-1.3	NS	NS
NM_022009	*Flii*	D2	4.97E-03	-1.5	NS	NS
NM_001159706	*Folh1*	D2	4.54E-02	1.7	2.03E-02	1.9
NM_008037	*Fosl2*	D2	1.50E-02	-1.8	NS	NS
NM_001080932	*Foxk2*	D2	4.75E-03	-1.3	NS	NS
NM_019739	*Foxo1*	D2	3.55E-02	-1.7	NS	NS
NM_194060	*Foxo6*	D2	1.97E-03	-1.9	NS	NS
NM_001286607	*Foxp2*	D2	2.19E-02	4.7	NS	NS
NM_001017983	*Foxred2*	D2	7.05E-03	-1.7	NS	NS
NM_007984	*Fscn1*	D2	1.74E-04	-1.4	NS	NS
NM_008046	*Fst*	D2	2.94E-02	-1.5	NS	NS
NM_025799	*Fuca2*	D2	1.74E-02	1.4	NS	NS
NM_011814	*Fxr2*	D2	1.72E-02	-1.3	NS	NS
NM_010246	*Fzd9*	D2	3.64E-02	-1.5	NS	NS
NM_019757	*Fzr1*	D2	6.11E-03	-1.5	NS	NS
NM_001162477	*Gab2*	D2	1.55E-02	-1.4	NS	NS
NM_020590	*Gabarapl1*	D2	2.47E-02	1.3	NS	NS
NM_001271467	*Gabpb1*	D2	1.63E-02	-1.5	1.88E-02	-1.5
NM_011817	*Gadd45g*	D2	2.27E-02	1.8	NS	NS
NM_001177691	*Gal3st1*	D2	4.01E-02	1.6	NS	NS
NM_178389	*Gale*	D2	4.65E-03	-1.7	NS	NS
NM_001081421	*Galnt16*	D2	3.18E-02	1.4	NS	NS
NM_008083	*Gap43*	D2	2.79E-02	-1.2	NS	NS
NM_028022	*Gatsl3*	D2	NS	NS	1.90E-02	2.0
NM_001163282	*Gdf1*	D2	2.18E-02	-1.9	NS	NS
NM_145741	*Gdf10*	D2	9.46E-04	-1.6	NS	NS
NM_144786	*Ggt7*	D2	4.22E-02	-1.3	NS	NS
NM_016867	*Gipc2*	D2	4.97E-02	1.7	NS	NS
NM_010288	*Gja1*	D2	8.56E-03	1.4	NS	NS
NM_080450	*Gjc3*	D2	3.64E-03	2.3	NS	NS
NM_010290	*Gjd2*	D2	1.20E-02	-1.5	NS	NS
NM_001286728	*Glcci1*	D2	4.41E-02	1.4	NS	NS
NM_027450	*Glipr2*	D2	NS	NS	3.75E-02	1.6

NM_029102	*Glt8d2*	D2	1.12E-03	2.3	7.95E-03	2.1
NM_008131	*Glul*	D2	1.15E-03	1.5	NS	NS
NM_001281514	*Gm10354*	D2	NS	NS	3.28E-02	1.9
NM_001037926	*Gm13051*	D2	8.82E-03	1.7	NS	NS
ENSMUST00000105763	*Gm13101*	D2	4.45E-02	1.5	NS	NS
NM_001177392	*Gm13547*	D2	3.31E-02	1.8	NS	NS
ENSMUST00000140106	*Gm14569*	D2	8.44E-03	1.9	NS	NS
NM_001102665	*Gm14920*	D2	2.19E-02	1.6	NS	NS
ENSMUST00000164512	*Gm2974*	D2	3.18E-02	-1.5	NS	NS
ENSMUST00000163525	*Gm3239*	D2	3.24E-02	-1.5	NS	NS
ENSMUST00000181880	*Gm3532*	D2	4.88E-02	-1.6	NS	NS
ENSMUST00000171906	*Gm3667*	D2	3.54E-02	-1.4	NS	NS
NM_001034859	*Gm4841*	D2	1.11E-02	-1.9	NS	NS
NR_045668	*Gm5124*	D2	8.16E-03	-1.5	NS	NS
NM_001033266	*Gm525*	D2	3.26E-02	1.7	NS	NS
XM_485592.5	*Gm5553*	D2	3.39E-02	1.7	NS	NS
NM_001033789	*Gm5820*	D2	1.07E-02	-1.9	NS	NS
NM_198101	*Gmip*	D2	3.01E-02	-1.6	NS	NS
NM_001190414	*Gne*	D2	NS	NS	4.47E-02	-1.4
NM_153547	*Gnl3*	D2	5.35E-03	-1.7	NS	NS
NM_001141983	*Golga7b*	D2	1.08E-02	-1.5	NS	NS
NM_001177669	*Golph3l*	D2	2.80E-02	1.4	NS	NS
NM_008149	*Gpam*	D2	5.03E-04	1.4	1.90E-02	1.3
NM_001110809	*Gpatch4*	D2	1.17E-02	-1.6	1.16E-02	-1.6
NM_016696	*Gpc1*	D2	1.70E-02	-1.5	NS	NS
NM_030084	*Gpr108*	D2	3.65E-02	1.5	NS	NS
NM_027518	*Gpr137c*	D2	1.35E-08	2.2	1.67E-07	2.1
NM_001190297	*Gpr155*	D2	3.07E-03	1.6	NS	NS
NM_029536	*Gpr165*	D2	3.52E-02	1.5	NS	NS
NM_010338	*Gpr37*	D2	1.32E-02	1.6	4.78E-02	1.5
NM_134438	*Gpr37l1*	D2	1.34E-02	1.4	NS	NS
NM_145066	*Gpr85*	D2	3.65E-02	1.4	4.13E-02	1.4
NM_012014	*Gprin1*	D2	1.74E-05	-1.6	NS	NS
NM_001177874	*Gps1*	D2	4.18E-04	-1.4	NS	NS

NM_026240	*Gramd3*	D2	1.24E-02	1.6	NS	NS
NM_001033351	*Grin3a*	D2	2.23E-03	-1.7	NS	NS
NM_175012	*Grp*	D2	4.14E-02	-1.7	NS	NS
NM_153419	*Grwd1*	D2	3.31E-04	-2.1	8.82E-04	-2.1
NM_145147	*Gtpbp6*	D2	4.62E-02	-1.6	NS	NS
NM_010436	*H2afx*	D2	1.28E-03	-1.6	NS	NS
NM_008216	*Has2*	D2	1.44E-02	-2.1	NS	NS
NM_028633	*Haus7*	D2	2.49E-02	-1.6	NS	NS
NM_008227	*Hcn3*	D2	2.11E-03	-1.7	NS	NS
NM_001081192	*Hcn4*	D2	4.19E-04	-1.8	1.87E-02	-1.6
NM_198962	*Hcrtr2*	D2	2.69E-03	-1.8	NS	NS
NM_144835	*Heatr1*	D2	1.21E-02	-1.6	1.08E-02	-1.7
NM_001001883	*Hecw2*	D2	2.53E-02	-1.7	NS	NS
NM_175256	*Heg1*	D2	4.82E-02	1.5	NS	NS
NM_175189	*Hepacam*	D2	4.54E-02	1.4	NS	NS
NM_010419	*Hes5*	D2	3.55E-02	-1.5	NS	NS
NM_010422	*Hexb*	D2	2.03E-05	1.7	NS	NS
NM_001162950	*Hif3a*	D2	2.74E-02	1.9	NS	NS
NM_008248	*Hint1*	D2	3.82E-02	-1.4	NS	NS
NM_178213	*Hist2h2ab*	D2	5.42E-04	-1.9	NS	NS
NM_007772	*Hivep1*	D2	4.87E-04	-1.6	NS	NS
NM_008255	*Hmgcr*	D2	2.40E-06	-1.9	1.39E-04	-1.7
NM_008256	*Hmgcs2*	D2	2.21E-04	2.6	1.25E-02	2.1
NM_016957	*Hmgn2*	D2	9.36E-03	1.4	NS	NS
NM_080462	*Hnmt*	D2	3.28E-02	1.5	NS	NS
NM_007545	*Hrk*	D2	4.98E-03	1.7	NS	NS
NM_010476	*Hsd17b7*	D2	7.59E-08	-2.1	4.34E-08	-2.1
NM_175185	*Hsdl1*	D2	1.11E-02	-1.5	NS	NS
NM_024255	*Hsdl2*	D2	1.91E-03	1.6	NS	NS
NM_010479	*Hspa1a*	D2	1.43E-02	1.5	NS	NS
NM_001002012	*Hspa2*	D2	8.43E-03	1.7	NS	NS
NM_024172	*Hspbp1*	D2	9.90E-04	-1.5	NS	NS
NM_008303	*Hspe1*	D2	6.86E-03	-1.4	NS	NS
NM_019564	*Htra1*	D2	1.55E-07	2.4	1.51E-03	1.8
NM_019752	*Htra2*	D2	3.61E-02	-1.3	NS	NS
NM_008317	*Hyal1*	D2	2.49E-02	1.7	NS	NS
NM_031166	*Id4*	D2	1.62E-02	2.0	NS	NS
NM_145360	*Idi1*	D2	7.08E-07	-2.0	8.75E-08	-2.1

NM_001101605	*Ifit1bl1*	D2	NS	NS	3.77E-02	1.9
NM_013562	*Ifrd1*	D2	2.94E-03	-1.7	4.38E-03	-1.7
NM_198610	*Igsf21*	D2	1.14E-02	-1.6	NS	NS
NM_001033323	*Igsf9b*	D2	NS	NS	1.36E-02	1.7
NM_011772	*Ikzf4*	D2	NS	NS	2.88E-02	-1.5
NM_145837	*Il17d*	D2	1.24E-02	-1.5	NS	NS
NM_001123382	*Il1r1*	D2	7.65E-03	1.9	NS	NS
NM_001159317	*Il1rap*	D2	NS	NS	1.05E-02	-1.7
NM_011919	*Ing1*	D2	2.08E-02	-1.4	1.32E-03	-1.5
NM_172439	*Inpp5j*	D2	1.20E-03	1.9	7.71E-04	2.0
NM_153526	*Insig1*	D2	7.92E-07	-2.3	1.54E-06	-2.3
NM_026748	*Ints1*	D2	2.68E-02	-1.3	NS	NS
NM_029665	*Ipo11*	D2	1.76E-02	-1.4	NS	NS
NM_023579	*Ipo5*	D2	8.63E-03	-1.3	NS	NS
NM_016851	*Irf6*	D2	1.51E-03	1.8	NS	NS
NM_010586	*Itpr2*	D2	2.38E-03	1.6	NS	NS
NM_027391	*Iyd*	D2	4.48E-02	1.6	NS	NS
NM_033398	*Jmjd6*	D2	5.84E-03	-1.5	NS	NS
NM_021310	*Jmy*	D2	3.99E-02	1.4	NS	NS
NM_010591	*Jun*	D2	6.88E-14	3.3	9.39E-11	2.9
NM_001286944	*Jund*	D2	5.24E-03	1.5	1.59E-03	1.6
NM_001038010	*Kat2a*	D2	1.31E-05	-1.6	4.97E-02	-1.3
NM_145983	*Kcna5*	D2	3.00E-02	1.8	NS	NS
NM_001039347	*Kcnd3*	D2	3.82E-02	-1.4	8.92E-03	-1.6
NM_001081134	*Kcng1*	D2	1.21E-06	-2.5	3.25E-05	-2.2
NM_001039056	*Kcnj15*	D2	1.07E-02	-1.7	NS	NS
NM_028231	*Kcnmb2*	D2	3.10E-02	-1.6	NS	NS
NM_032397	*Kcnn1*	D2	1.34E-02	-1.6	NS	NS
NM_026135	*Kctd16*	D2	1.22E-04	2.0	1.24E-04	2.1
NM_027008	*Kctd5*	D2	NS	NS	3.92E-02	1.4
NM_172262	*Kdm1b*	D2	3.52E-02	-1.5	NS	NS
NM_011317	*Khdrbs1*	D2	2.40E-03	-1.5	NS	NS
NM_001145779	*Kif2a*	D2	2.41E-02	-1.3	NS	NS
NM_172898	*Kirrel2*	D2	1.60E-02	1.7	4.48E-05	2.4
NM_001122733	*Kit*	D2	1.32E-05	-1.5	3.03E-02	-1.3
NM_008451	*Klc2*	D2	1.34E-02	-1.4	NS	NS
NM_010636	*Klf12*	D2	3.57E-03	1.4	NS	NS
NM_078477	*Klf16*	D2	7.59E-08	-2.3	7.86E-05	-1.9

NM_009769	*Klf5*	D2	2.94E-02	-1.7	NS	NS
NM_011803	*Klf6*	D2	1.51E-02	-1.5	3.03E-02	-1.5
NM_010638	*Klf9*	D2	1.24E-03	1.6	NS	NS
NM_029436	*Klhl24*	D2	2.54E-04	1.5	1.41E-02	1.4
NM_001033171	*Klrg2*	D2	NS	NS	4.62E-02	1.6
NM_010655	*Kpna2*	D2	4.19E-06	-1.8	2.58E-04	-1.7
NM_021284	*Kras*	D2	1.34E-02	-1.3	NS	NS
NM_201255	*Krt9*	D2	1.61E-02	2.0	NS	NS
NM_029571	*Kti12*	D2	2.73E-02	-1.3	4.74E-02	-1.3
NM_001289711	*L3mbtl2*	D2	1.91E-02	-1.5	NS	NS
NM_001113474	*Lair1*	D2	3.36E-02	1.6	NS	NS
NM_010688	*Lasp1*	D2	5.76E-03	-1.3	NS	NS
NM_027448	*Lca5*	D2	NS	NS	3.48E-02	-1.5
NM_177846	*Lcmt2*	D2	1.63E-02	1.7	NS	NS
NM_001247984	*Lcp1*	D2	NS	NS	4.62E-02	-1.5
NM_001077398	*Ldb2*	D2	2.18E-02	-1.5	NS	NS
NM_001252658	*Ldlr*	D2	1.92E-05	-2.4	3.42E-05	-2.4
NM_001018087	*Ldoc1*	D2	1.11E-02	-2.0	NS	NS
NM_001081193	*Lemd3*	D2	3.73E-02	-1.4	NS	NS
NM_173012	*Letm2*	D2	2.15E-02	1.5	1.20E-02	1.6
NM_008494	*Lfng*	D2	4.03E-04	1.7	2.92E-03	1.6
NM_001199043	*Lgals8*	D2	6.09E-03	1.5	2.78E-02	1.4
NM_001083125	*Lhx6*	D2	5.42E-04	-2.2	1.17E-02	-2.0
NM_010717	*Limk1*	D2	8.84E-04	-1.5	NS	NS
NM_144862	*Lims2*	D2	1.63E-02	1.9	NS	NS
NM_181074	*Lingo1*	D2	7.00E-05	-1.4	NS	NS
NM_001191001	*Lins1*	D2	1.69E-02	-1.3	NS	NS
NM_001163170	*Lix1l*	D2	2.42E-03	-1.5	NS	NS
NM_001002011	*Lmna*	D2	2.23E-04	-1.8	NS	NS
NM_001161769	*Lmo4*	D2	1.21E-02	-1.4	NS	NS
NM_201529	*Lmo7*	D2	1.09E-03	1.5	9.92E-03	1.4
ENSMUST00000096148	*LOC105242423*	D2	3.37E-02	1.9	NS	NS
NM_001081150	*Lonrf1*	D2	2.10E-02	-1.5	NS	NS
NM_028894	*Lonrf3*	D2	3.68E-03	1.9	NS	NS
NM_145376	*Lpcat1*	D2	3.88E-03	-1.3	NS	NS
NM_001141921	*Lrfn1*	D2	5.23E-04	-1.8	NS	NS
NM_027452	*Lrfn2*	D2	1.65E-02	-1.5	NS	NS

NM_001146048	*Lrrc1*	D2	4.67E-02	1.6	NS	NS
NM_001024645	*Lrrc16b*	D2	1.63E-02	-1.6	NS	NS
NM_145152	*Lrrc3*	D2	8.59E-04	-1.8	NS	NS
NM_153521	*Lrrc41*	D2	8.98E-03	-1.5	NS	NS
NM_133807	*Lrrc59*	D2	5.92E-04	-1.5	NS	NS
NM_001111142	*Lrrc73*	D2	7.68E-03	-1.4	NS	NS
NM_010732	*Lrrn2*	D2	5.16E-03	-1.6	NS	NS
NM_146006	*Lss*	D2	4.28E-02	-1.4	NS	NS
NM_181470	*Ltv1*	D2	8.04E-04	-1.9	1.88E-02	-1.7
NM_001252055	*Ly6c1*	D2	3.01E-03	2.2	NS	NS
NM_001135688	*Ly6h*	D2	3.80E-03	-1.5	NS	NS
NM_011942	*Lypla2*	D2	1.03E-03	-1.6	NS	NS
NM_021500	*Maea*	D2	7.67E-03	-1.4	NS	NS
NM_001199246	*Maged2*	D2	3.46E-02	-1.4	3.72E-02	-1.4
NM_053201	*Magee1*	D2	6.81E-03	-1.4	3.26E-02	-1.4
NM_026453	*Mak16*	D2	1.87E-02	-1.5	NS	NS
NM_011840	*Map2k5*	D2	4.70E-03	1.4	4.99E-02	1.3
NM_011948	*Map3k4*	D2	NS	NS	2.74E-02	1.5
NM_001043355	*Map6*	D2	2.33E-02	-1.3	NS	NS
NM_011161	*Mapk11*	D2	6.65E-04	-1.9	NS	NS
NM_001045483	*Mapk1ip1*	D2	1.94E-02	1.3	2.35E-02	1.4
NM_015806	*Mapk6*	D2	2.14E-02	-1.6	NS	NS
NM_011841	*Mapk7*	D2	2.07E-06	-1.7	4.94E-02	-1.3
NM_021921	*Mapk8ip2*	D2	1.83E-02	-1.3	NS	NS
NM_010807	*Marcksl1*	D2	2.87E-02	-1.5	NS	NS
NM_175439	*Mars2*	D2	1.53E-03	-1.7	NS	NS
NM_008555	*Masp1*	D2	2.99E-02	-1.4	NS	NS
NM_010773	*Mbd2*	D2	6.16E-03	1.4	NS	NS
NM_028372	*Mblac2*	D2	7.93E-04	-1.7	NS	NS
NM_175341	*Mbnl2*	D2	2.22E-04	1.6	NS	NS
NM_029934	*Mboat7*	D2	1.83E-02	-1.6	NS	NS
NM_001085373	*Mcc*	D2	1.93E-02	-1.4	NS	NS
NM_008564	*Mcm2*	D2	NS	NS	3.98E-02	-1.6
NM_008567	*Mcm6*	D2	2.98E-02	-1.4	NS	NS
NM_001010833	*Mdc1*	D2	NS	NS	1.50E-02	-1.8
NM_001012335	*Mdk*	D2	3.99E-02	1.7	NS	NS
NM_001277309	*Mea1*	D2	1.57E-02	-1.7	NS	NS
NM_026896	*Med27*	D2	2.92E-02	-1.3	NS	NS

NM_025895	*Med28*	D2	4.10E-02	-1.4	NS	NS
NM_008587	*Mertk*	D2	9.24E-07	3.1	1.65E-03	2.3
NM_030705	*Mesdc1*	D2	1.22E-02	-1.8	NS	NS
NM_008591	*Met*	D2	3.55E-02	-1.6	NS	NS
NM_175224	*Metap1*	D2	1.91E-03	-1.6	NS	NS
NM_172567	*Mettl2*	D2	4.51E-02	-1.4	NS	NS
NM_025964	*Mettl21a*	D2	1.92E-02	1.4	NS	NS
NM_198615	*Mex3d*	D2	1.07E-03	-1.6	NS	NS
NM_028657	*Mfsd12*	D2	3.73E-02	-1.6	NS	NS
NM_029662	*Mfsd2a*	D2	1.54E-02	1.5	NS	NS
NM_023799	*Mgea5*	D2	1.22E-02	1.3	9.95E-06	1.5
NM_177461	*Micall1*	D2	2.21E-02	-1.6	NS	NS
NM_030110	*Micu3*	D2	6.95E-03	1.4	NS	NS
NM_021565	*Midn*	D2	1.67E-06	-1.8	2.04E-03	-1.6
NM_153760	*Mill2*	D2	3.73E-02	1.7	NS	NS
NR_106092	*Mir7233*	D2	NS	NS	1.87E-02	2.0
NM_022328	*Mllt1*	D2	4.54E-02	-1.5	NS	NS
NM_001159384	*Mlx*	D2	1.76E-04	-1.6	NS	NS
NM_008609	*Mmp15*	D2	1.49E-05	-1.9	8.26E-05	-1.9
NM_172457	*Mob3a*	D2	5.77E-04	-1.8	NS	NS
NM_023773	*Mphosph8*	D2	1.11E-02	-1.4	NS	NS
NM_172610	*Mpped1*	D2	3.49E-02	-1.4	NS	NS
NM_027204	*Mrpl12*	D2	8.65E-03	-1.4	NS	NS
NM_026490	*Mrpl19*	D2	2.92E-02	-1.3	NS	NS
NM_026851	*Mrpl52*	D2	1.87E-02	-1.7	NS	NS
NM_025878	*Mrps18b*	D2	2.53E-02	-1.4	1.35E-02	-1.5
NM_026835	*Ms4a6d*	D2	4.61E-02	1.9	NS	NS
NM_001163833	*Msl3l2*	D2	4.88E-02	1.6	NS	NS
NM_025436	*Msmo1*	D2	6.29E-09	-2.7	5.99E-08	-2.6
NM_013602	*Mt1*	D2	7.59E-08	2.1	2.34E-05	1.8
NM_008630	*Mt2*	D2	2.86E-11	4.0	1.81E-10	3.8
NM_008638	*Mthfd2*	D2	1.11E-03	-1.9	NS	NS
NM_020009	*Mtor*	D2	3.42E-02	-1.3	NS	NS
NM_001005863	*Mtus1*	D2	2.32E-03	1.7	1.58E-02	1.6
NM_023431	*Mum1*	D2	1.50E-02	-1.4	NS	NS
NM_001164630	*Mum1l1*	D2	3.85E-02	1.7	NS	NS
NM_138656	*Mvd*	D2	2.34E-10	-3.1	2.95E-05	-2.2
NM_023556	*Mvk*	D2	1.75E-02	-1.7	3.87E-02	-1.7

NM_010753	*Mxd4*	D2	3.44E-03	1.7	2.34E-02	1.6
NM_001008542	*Mxi1*	D2	2.34E-10	2.0	5.73E-09	1.9
NM_001177352	*Myc*	D2	4.23E-04	-1.7	NS	NS
NM_001033481	*Myrf*	D2	3.53E-02	1.8	NS	NS
NM_030153	*Naa35*	D2	NS	NS	4.24E-02	-1.4
NM_001040395	*Nadk2*	D2	3.13E-04	1.5	NS	NS
NM_178421	*Nanos1*	D2	7.49E-03	-1.6	NS	NS
NM_001285489	*Nap1l4*	D2	4.82E-02	-1.3	NS	NS
NM_178728	*Napepld*	D2	1.69E-02	1.9	NS	NS
NM_001081475	*Nasp*	D2	8.88E-03	-1.6	NS	NS
NM_001114085	*Nde1*	D2	3.10E-03	1.7	2.81E-03	1.7
NM_172399	*Ndnf*	D2	4.40E-02	-1.4	NS	NS
NM_001145959	*Ndrg2*	D2	1.91E-03	1.3	NS	NS
NM_027244	*Ndufa11*	D2	1.45E-02	-1.4	NS	NS
NM_026742	*Ndufaf4*	D2	8.23E-03	-1.4	5.70E-04	-1.6
NM_026612	*Ndufb2*	D2	1.57E-02	-1.4	NS	NS
NM_025843	*Ndufb7*	D2	3.29E-02	-1.5	NS	NS
NM_029272	*Ndufs7*	D2	3.28E-02	-1.4	NS	NS
NM_001271443	*Ndufs8*	D2	2.66E-02	-1.3	NS	NS
NM_145138	*Nek9*	D2	2.78E-02	1.4	NS	NS
NM_021393	*Nelfb*	D2	4.47E-02	-1.4	NS	NS
NM_016701	*Nes*	D2	4.23E-02	-1.5	NS	NS
NM_001081324	*Neto2*	D2	3.71E-02	-1.3	NS	NS
NM_001271411	*Nfam1*	D2	4.29E-02	1.6	NS	NS
NM_001170591	*Nfu1*	D2	3.49E-02	-1.4	NS	NS
NM_011482	*Nhp2l1*	D2	2.19E-04	-1.5	NS	NS
NM_001163592	*Nhsl1*	D2	4.10E-02	1.3	NS	NS
NM_026472	*Nifk*	D2	NS	NS	4.85E-02	-1.3
NM_145469	*Nipal2*	D2	1.83E-02	1.5	1.17E-02	1.6
NM_028995	*Nipal3*	D2	2.60E-02	1.5	2.63E-02	1.6
NM_025623	*Nipsnap3b*	D2	3.00E-02	-1.4	NS	NS
NM_008704	*Nme1*	D2	4.08E-02	-1.3	NS	NS
NM_001077529	*Nme2*	D2	9.09E-03	-1.3	NS	NS
NM_019515	*Nmu*	D2	4.20E-02	-1.6	NS	NS
NM_010923	*Nnat*	D2	4.62E-02	-1.3	NS	NS
NM_021303	*Noc2l*	D2	1.35E-04	-1.7	NS	NS
NM_153570	*Noc4l*	D2	6.70E-04	-1.6	NS	NS
NM_009834	*Noct*	D2	2.54E-03	-1.6	2.04E-03	-1.7

NM_133800	*Nol12*	D2	1.97E-03	-1.8	NS	NS
NM_001001986	*Nol4l*	D2	5.42E-04	-1.6	1.35E-02	-1.5
NM_153057	*Nomo1*	D2	2.30E-03	-1.5	NS	NS
NM_138747	*Nop2*	D2	2.97E-07	-1.9	3.86E-03	-1.5
NM_024193	*Nop56*	D2	4.27E-02	-1.4	7.41E-03	-1.6
NM_008718	*Npas1*	D2	8.90E-04	-1.9	NS	NS
NM_008723	*Npm3*	D2	4.41E-02	-1.5	NS	NS
NM_001029836	*Npnt*	D2	4.69E-02	1.5	NS	NS
NM_001139509	*Nr4a2*	D2	1.48E-03	-3.0	NS	NS
NM_015743	*Nr4a3*	D2	6.62E-04	-1.5	NS	NS
NM_173440	*Nrip1*	D2	2.34E-02	1.4	NS	NS
NM_008737	*Nrp1*	D2	1.90E-02	-1.5	NS	NS
NM_001077403	*Nrp2*	D2	2.79E-02	-1.4	NS	NS
NM_010941	*Nsdhl*	D2	3.61E-05	-1.5	7.99E-05	-1.5
NM_027602	*Nsun7*	D2	4.93E-02	1.6	NS	NS
NM_001164363	*Nt5c2*	D2	6.11E-03	1.4	NS	NS
NM_001163348	*Ntng1*	D2	2.21E-02	-1.9	NS	NS
NM_001025074	*Ntrk2*	D2	4.10E-02	1.3	NS	NS
NM_001190179	*Nup35*	D2	NS	NS	3.07E-02	-1.5
NM_008751	*Nxph1*	D2	1.33E-04	-1.7	2.03E-02	-1.5
NM_001161430	*Nxt2*	D2	4.25E-02	1.3	NS	NS
NM_026950	*Ociad2*	D2	4.23E-02	1.4	NS	NS
NM_013614	*Odc1*	D2	3.29E-02	1.5	NS	NS
NM_173777	*Olfm2*	D2	1.90E-03	-1.6	NS	NS
NM_001005524	*Olfr194*	D2	4.12E-02	2.0	NS	NS
NM_001011780	*Olfr287*	D2	1.20E-02	1.8	NS	NS
NR_033638	*Olfr29-ps1*	D2	1.34E-02	-1.8	NS	NS
NM_147115	*Olfr578*	D2	1.38E-02	1.8	NS	NS
NM_147121	*Olfr644*	D2	4.10E-02	1.7	NS	NS
NM_147061	*Olfr691*	D2	1.13E-02	-1.9	NS	NS
NM_019409	*Omg*	D2	7.83E-04	1.5	NS	NS
NM_010098	*Opn3*	D2	3.18E-03	-1.9	NS	NS
NM_001003717	*Osbpl8*	D2	4.05E-02	-1.4	NS	NS
NM_001134791	*Osbpl9*	D2	1.35E-04	1.4	3.25E-04	1.4
NM_001285839	*Osgepl1*	D2	7.58E-03	1.6	NS	NS
NM_001001490	*Oxgr1*	D2	4.92E-02	-1.8	NS	NS
NM_027032	*Pacrg*	D2	3.96E-03	1.6	NS	NS
NM_008776	*Pafah1b3*	D2	1.23E-03	-1.9	NS	NS

NM_026420	*Paip2*	D2	3.35E-04	1.4	NS	NS
NM_146169	*Paip2b*	D2	NS	NS	3.19E-02	1.5
NM_001169131	*Papd7*	D2	NS	NS	3.92E-02	-1.5
NM_198410	*Paqr6*	D2	2.52E-02	1.6	NS	NS
NM_028829	*Paqr8*	D2	7.63E-03	1.4	1.06E-02	1.5
NM_011039	*Pax7*	D2	NS	NS	4.67E-02	1.5
NM_001081251	*Pbrm1*	D2	2.40E-02	-1.3	NS	NS
NM_011865	*Pcbp1*	D2	8.59E-04	-1.5	NS	NS
NM_029357	*Pcdh1*	D2	2.60E-02	-1.2	NS	NS
NM_001042726	*Pcdh8*	D2	4.33E-03	-1.5	NS	NS
NM_146129	*Pcif1*	D2	4.39E-03	-1.6	NS	NS
NM_183028	*Pcmtd1*	D2	2.21E-03	1.4	NS	NS
NM_153594	*Pcmtd2*	D2	4.98E-03	1.7	NS	NS
NM_001163144	*Pcsk5*	D2	3.42E-02	-1.5	NS	NS
NM_024229	*Pcyt2*	D2	1.38E-02	-1.5	7.97E-03	-1.6
NM_001177980	*Pde4b*	D2	1.01E-02	1.4	NS	NS
NM_013875	*Pde7b*	D2	1.24E-02	2.6	NS	NS
NM_008808	*Pdgfa*	D2	2.70E-05	-1.7	3.41E-05	-1.7
NM_011057	*Pdgfb*	D2	1.11E-02	-1.7	NS	NS
NM_001163794	*Pdik1l*	D2	7.85E-03	-1.5	NS	NS
NM_013743	*Pdk4*	D2	2.50E-03	1.8	NS	NS
NM_016861	*Pdlim1*	D2	5.63E-04	2.6	NS	NS
NM_019501	*Pdss1*	D2	1.51E-02	-1.7	NS	NS
NM_008820	*Pepd*	D2	4.08E-02	-1.4	NS	NS
NM_001159367	*Per1*	D2	3.82E-02	1.4	NS	NS
NM_134025	*Pex12*	D2	NS	NS	3.92E-02	1.6
NM_011072	*Pfn1*	D2	4.96E-02	-1.3	NS	NS
NM_001163314	*Pgap1*	D2	2.67E-02	-1.3	NS	NS
NM_025954	*Pgp*	D2	3.27E-02	-1.4	NS	NS
NM_001007154	*Phactr3*	D2	1.94E-02	1.4	NS	NS
NM_008831	*Phb*	D2	7.59E-03	-1.3	1.47E-03	-1.4
NM_172705	*Phf13*	D2	6.09E-03	-1.8	NS	NS
NM_030064	*Phf23*	D2	2.43E-04	-1.8	2.90E-02	-1.5
NM_026737	*Phf5a*	D2	NS	NS	1.51E-02	-1.6
NM_009344	*Phlda1*	D2	1.38E-02	-1.8	1.93E-02	-1.9
NM_001278207	*Pih1d1*	D2	2.33E-02	-1.4	NS	NS
NM_178149	*Pik3ip1*	D2	1.92E-06	2.4	1.12E-07	2.7
NM_001024955	*Pik3r1*	D2	2.23E-10	1.9	1.25E-10	1.9

NM_181585	*Pik3r3*	D2	9.98E-05	1.6	NS	NS
NM_145478	*Pim3*	D2	1.97E-02	-1.8	NS	NS
NM_028228	*Pinx1*	D2	2.69E-03	-1.7	4.83E-03	-1.7
NM_001099779	*Pklr*	D2	3.82E-02	1.5	NS	NS
NM_001029838	*Pknox2*	D2	4.92E-02	-1.5	NS	NS
NM_008872	*Plat*	D2	3.49E-02	1.5	NS	NS
NM_152813	*Plcd3*	D2	2.63E-04	2.0	1.65E-02	1.7
NM_001081456	*Plcd4*	D2	6.68E-03	1.7	NS	NS
NM_019588	*Plce1*	D2	7.24E-03	1.8	NS	NS
NM_001177503	*Plekhd1*	D2	NS	NS	1.65E-02	1.5
NM_024413	*Plekhf1*	D2	1.36E-04	2.0	1.35E-02	1.7
NM_001033253	*Plekhg1*	D2	5.78E-08	2.4	2.28E-05	2.0
NM_013807	*Plk3*	D2	1.51E-06	-2.1	6.16E-04	-1.8
NM_011123	*Plp1*	D2	1.01E-02	4.8	NS	NS
NM_018797	*Plxnc1*	D2	1.83E-02	-1.4	NS	NS
NM_008884	*Pml*	D2	NS	NS	6.81E-03	1.6
NM_026925	*Pnlip*	D2	4.10E-02	-1.5	NS	NS
NM_008891	*Pnn*	D2	3.53E-02	-1.4	NS	NS
NM_025443	*Pno1*	D2	3.22E-02	-1.4	2.75E-02	-1.5
NM_001205075	*Pnoc*	D2	1.31E-02	-1.5	NS	NS
NM_026383	*Pnrc2*	D2	1.26E-03	1.5	3.04E-02	1.4
NM_178627	*Poldip3*	D2	2.92E-02	-1.4	NS	NS
NM_020032	*Poll*	D2	NS	NS	2.71E-02	1.8
NM_009086	*Polr1b*	D2	9.60E-03	-1.7	NS	NS
NM_001164082	*Polr3d*	D2	NS	NS	2.11E-03	-1.7
NM_148932	*Pom121*	D2	2.68E-04	-1.6	NS	NS
NM_133249	*Ppargc1b*	D2	7.01E-03	-1.7	1.10E-02	-1.7
NM_008014	*Ppm1g*	D2	3.31E-04	-1.4	1.01E-02	-1.3
NM_175523	*Ppm1k*	D2	9.61E-04	1.7	NS	NS
NM_008889	*Ppp1r14b*	D2	7.75E-03	-1.6	NS	NS
NM_027242	*Ppp1r35*	D2	1.55E-02	-1.6	2.43E-02	-1.6
NM_009358	*Ppp2r5d*	D2	3.72E-02	-1.3	NS	NS
NM_001081214	*Pprc1*	D2	4.40E-02	-1.8	NS	NS
NM_007548	*Prdm1*	D2	3.22E-02	-1.6	NS	NS
NM_025596	*Prelid1*	D2	4.92E-02	-1.3	NS	NS
NM_177782	*Prex1*	D2	1.05E-02	-1.4	NS	NS
NM_178143	*Prkaa2*	D2	3.27E-02	-1.3	NS	NS
NM_008859	*Prkcq*	D2	1.70E-02	1.5	NS	NS

NM_001252476	*Prmt1*	D2	1.36E-04	-1.7	4.64E-02	-1.4
NM_001077638	*Prmt2*	D2	2.00E-04	-1.5	NS	NS
NM_145404	*Prmt7*	D2	4.86E-03	-1.5	NS	NS
NM_001126338	*Prnd*	D2	4.38E-05	-2.3	NS	NS
NM_011172	*Prodh*	D2	3.21E-02	1.6	NS	NS
NM_018786	*Prpf40b*	D2	1.70E-02	-1.6	NS	NS
NM_175022	*Prr12*	D2	1.38E-02	-1.6	NS	NS
NM_027626	*Psd3*	D2	1.20E-02	1.4	NS	NS
NM_177698	*Psd3*	D2	2.53E-02	1.3	NS	NS
NM_009439	*Psmd3*	D2	4.74E-02	-1.3	NS	NS
NM_001282017	*Psmd4*	D2	1.91E-02	-1.4	NS	NS
NM_011192	*Psme3*	D2	1.89E-02	-1.3	NS	NS
NM_025682	*Pspc1*	D2	1.23E-02	-1.5	NS	NS
NM_028474	*Ptchd4*	D2	1.11E-02	-1.7	NS	NS
NM_133949	*Ptov1*	D2	4.63E-02	-1.3	NS	NS
NM_001166388	*Ptp4a3*	D2	3.82E-02	-1.4	NS	NS
NM_207232	*Ptpdc1*	D2	NS	NS	4.94E-02	1.6
NM_011201	*Ptpn1*	D2	6.18E-03	-1.4	NS	NS
NM_019933	*Ptpn4*	D2	6.11E-03	1.7	NS	NS
NM_001163565	*Ptpn5*	D2	1.63E-02	-1.3	NS	NS
NM_019651	*Ptpn9*	D2	2.94E-04	-1.5	NS	NS
NM_025412	*Pycrl*	D2	3.25E-02	-1.6	NS	NS
NM_133198	*Pygl*	D2	4.25E-02	1.7	NS	NS
NM_001159516	*Qk*	D2	1.08E-02	1.2	NS	NS
NM_026677	*Rab13*	D2	3.19E-02	1.6	NS	NS
NM_029494	*Rab30*	D2	1.71E-07	2.0	3.89E-07	2.0
NM_133685	*Rab31*	D2	1.76E-02	1.3	NS	NS
NM_175122	*Rab39b*	D2	NS	NS	4.51E-03	1.4
NM_029391	*Rab4b*	D2	9.61E-03	-1.5	NS	NS
NM_001038621	*Rabgap1l*	D2	3.25E-05	1.5	3.08E-05	1.5
NM_001199059	*Rabgef1*	D2	4.30E-02	-1.3	NS	NS
NM_133223	*Rac3*	D2	1.47E-02	-1.4	NS	NS
NM_001277938	*Rad51d*	D2	4.23E-02	1.6	NS	NS
NM_019491	*Rala*	D2	1.01E-02	-1.5	NS	NS
NM_001145834	*Ralgds*	D2	1.96E-02	-1.4	NS	NS
NM_009391	*Ran*	D2	4.83E-03	-1.6	NS	NS
NM_001252466	*Ranbp3*	D2	NS	NS	2.22E-02	-1.4
NM_001099624	*Rapgef2*	D2	5.72E-11	2.0	4.97E-05	1.5

NM_175930	*Rapgef5*	D2	3.01E-02	1.4	NS	NS
NM_001252494	*Rapgef6*	D2	5.95E-09	2.4	3.30E-07	2.1
NM_001176528	*Rara*	D2	4.18E-05	-1.8	1.80E-03	-1.7
NM_009025	*Rasa3*	D2	4.31E-03	-1.6	7.51E-03	-1.6
NM_001166493	*Rasgrp3*	D2	NS	NS	3.95E-02	-1.5
NM_028234	*Rbm33*	D2	3.11E-03	1.5	1.41E-02	1.4
NM_001141981	*Rbm43*	D2	4.83E-03	1.5	1.25E-02	1.5
NM_001081549	*Rcan1*	D2	3.59E-02	1.3	NS	NS
NM_021557	*Rdh11*	D2	8.13E-03	-1.6	NS	NS
NM_030017	*Rdh12*	D2	NS	NS	1.88E-02	1.8
NM_001204931	*Reep6*	D2	2.94E-02	-1.5	NS	NS
NM_153793	*Rell2*	D2	1.04E-02	-1.4	NS	NS
NM_011261	*Reln*	D2	1.63E-02	-1.6	NS	NS
NM_024233	*Rexo2*	D2	6.44E-04	-1.7	NS	NS
NM_016846	*Rgl1*	D2	4.17E-02	1.3	NS	NS
NM_178615	*Rgmb*	D2	3.68E-02	-1.3	NS	NS
NM_009061	*Rgs2*	D2	NS	NS	4.15E-02	-1.9
NM_009062	*Rgs4*	D2	5.28E-03	1.6	NS	NS
NM_001165934	*Rgs9*	D2	3.22E-02	1.7	NS	NS
NM_146002	*Rhbdd2*	D2	2.66E-02	-1.5	3.66E-02	-1.5
NM_053075	*Rheb*	D2	3.70E-02	-1.3	NS	NS
NM_153514	*Rhobtb2*	D2	8.03E-04	-1.6	3.78E-03	-1.6
NM_175092	*Rhof*	D2	3.89E-03	-1.5	NS	NS
NM_001004193	*Rhox8*	D2	3.13E-02	1.4	NS	NS
NM_030168	*Rictor*	D2	4.12E-02	1.3	NS	NS
NM_001037915	*Ripply1*	D2	NS	NS	2.75E-02	1.8
NM_172612	*Rnd1*	D2	4.95E-05	-1.9	NS	NS
NM_144528	*Rnf126*	D2	8.50E-03	-1.3	NS	NS
NM_001166553	*Rnf145*	D2	1.17E-06	-1.7	2.19E-04	-1.5
NM_001289917	*Rora*	D2	3.55E-02	1.8	NS	NS
NM_001163461	*Rpap2*	D2	4.23E-02	1.4	NS	NS
NM_011286	*Rph3a*	D2	3.71E-02	-1.4	NS	NS
NM_133982	*Rpp25*	D2	8.01E-05	-2.0	9.31E-06	-2.2
NM_175109	*Rps19bp1*	D2	3.18E-02	-1.5	NS	NS
NM_146244	*Rps6kl1*	D2	2.43E-04	1.9	NS	NS
NM_199447	*Rrp12*	D2	1.08E-02	-1.7	NS	NS
NM_001159671	*Rsph6a*	D2	3.18E-02	1.5	NS	NS
NM_198620	*Rundc3b*	D2	1.31E-02	-1.3	NS	NS

NM_001111026	*Runx1t1*	D2	3.53E-02	-1.4	NS	NS
NM_001083807	*Rusc1*	D2	2.87E-02	-1.5	NS	NS
NM_178717	*Rxfp3*	D2	1.34E-02	-1.7	NS	NS
NM_010101	*S1pr3*	D2	4.48E-02	-1.7	NS	NS
NM_001081415	*Samd1*	D2	1.24E-03	-1.7	NS	NS
NM_172676	*Samd10*	D2	1.39E-02	-1.7	NS	NS
NM_001037221	*Samd4*	D2	1.36E-03	1.8	NS	NS
NM_001168521	*Sarm1*	D2	3.76E-02	-1.6	NS	NS
NM_016926	*Sart3*	D2	3.87E-02	-1.5	NS	NS
NM_145587	*Sbk1*	D2	2.12E-03	-1.7	NS	NS
NM_001008422	*Scaf1*	D2	3.79E-02	-1.5	NS	NS
NM_011886	*Scamp3*	D2	1.75E-02	-1.6	NS	NS
NM_020255	*Scand1*	D2	3.84E-02	-1.6	NS	NS
NM_172604	*Scara3*	D2	1.74E-02	1.5	4.62E-02	1.5
NM_009129	*Scg2*	D2	5.50E-04	1.4	4.13E-02	1.3
NM_009136	*Scrg1*	D2	1.01E-02	1.4	NS	NS
NM_130893	*Scrt1*	D2	7.65E-03	-1.7	NS	NS
NM_023912	*Scyl1*	D2	1.20E-02	-1.5	NS	NS
NM_011521	*Sdc4*	D2	1.30E-06	3.0	3.75E-03	2.1
NM_025468	*Sec11c*	D2	4.52E-02	-1.3	NS	NS
NM_024206	*Sec13*	D2	3.42E-02	-1.5	4.27E-02	-1.5
NM_153055	*Sec63*	D2	2.04E-02	1.4	NS	NS
NM_009151	*Selplg*	D2	1.11E-02	1.9	NS	NS
NM_001126047	*Sema4c*	D2	3.68E-02	-1.4	NS	NS
NM_001042613	*Sepp1*	D2	3.91E-02	1.4	NS	NS
NM_008458	*Serpina3c*	D2	5.25E-04	2.1	NS	NS
NM_001177794	*Sertad4*	D2	NS	NS	1.88E-02	1.5
NM_030261	*Sesn3*	D2	4.24E-08	1.8	2.47E-03	1.5
NM_001040398	*Setd1b*	D2	NS	NS	4.62E-02	-1.5
NM_001035123	*Setd6*	D2	2.13E-02	-1.6	NS	NS
NM_080793	*Setd7*	D2	4.95E-02	1.5	NS	NS
NM_021286	*Sez6*	D2	1.24E-03	-1.6	NS	NS
NM_153053	*Sf3b4*	D2	1.42E-02	-1.5	NS	NS
NM_134114	*Sft2d1*	D2	NS	NS	2.50E-02	-1.4
NM_029339	*Sgf29*	D2	4.19E-02	-1.4	NS	NS
NM_001168525	*Sgms1*	D2	7.67E-03	1.9	NS	NS
NM_001162965	*Sgsm1*	D2	1.34E-02	-1.4	NS	NS
NM_134091	*Sgsm3*	D2	3.08E-02	-1.5	NS	NS

NM_009167	*Shc3*	D2	3.00E-05	-1.5	1.32E-02	-1.3
NM_138676	*Shkbp1*	D2	2.99E-02	-1.6	NS	NS
NM_001252316	*Shmt2*	D2	7.91E-03	-1.5	NS	NS
NM_001168505	*Shoc2*	D2	1.88E-04	-1.3	NS	NS
NM_001286538	*Sigmar1*	D2	1.59E-03	-1.5	NS	NS
NM_010831	*Sik1*	D2	2.86E-04	-1.7	2.24E-02	-1.5
NM_176987	*Simc1*	D2	3.18E-02	-1.5	NS	NS
NM_001110350	*Sin3a*	D2	3.13E-02	-1.3	NS	NS
NM_001039090	*Skil*	D2	4.69E-02	1.3	NS	NS
NM_027921	*Slc16a14*	D2	4.20E-02	1.4	2.46E-02	1.5
NM_001029842	*Slc16a6*	D2	4.23E-06	2.9	NS	NS
NM_018861	*Slc1a4*	D2	2.51E-02	-1.3	NS	NS
NM_001159593	*Slc20a1*	D2	3.00E-03	-1.4	1.95E-02	-1.4
NM_001033167	*Slc22a23*	D2	4.14E-02	-1.3	NS	NS
NM_001110240	*Slc24a2*	D2	3.64E-03	1.7	NS	NS
NM_153150	*Slc25a1*	D2	7.15E-04	-1.6	NS	NS
NM_023596	*Slc29a3*	D2	3.66E-02	1.4	3.15E-02	1.4
NM_011400	*Slc2a1*	D2	3.83E-03	1.7	1.61E-02	1.7
NM_172479	*Slc38a5*	D2	1.57E-02	-1.5	NS	NS
NM_001037717	*Slc38a6*	D2	1.37E-02	1.5	3.28E-02	1.4
NM_001012305	*Slc39a12*	D2	2.68E-06	2.2	NS	NS
NM_001199283	*Slc43a2*	D2	8.16E-04	1.7	1.65E-03	1.7
NM_001081263	*Slc44a5*	D2	6.70E-04	1.6	6.75E-05	1.8
NM_029643	*Slc52a2*	D2	1.70E-02	-1.5	NS	NS
NM_001142809	*Slc6a8*	D2	4.79E-02	1.3	NS	NS
NM_017394	*Slc7a10*	D2	3.18E-02	1.4	NS	NS
NM_012030	*Slc9a3r1*	D2	5.31E-03	-1.5	NS	NS
NM_177909	*Slc9a9*	D2	2.43E-04	1.7	NS	NS
NM_001038643	*Slco3a1*	D2	3.91E-04	-1.6	1.07E-04	-1.7
NM_148933	*Slco4a1*	D2	3.68E-02	-1.6	NS	NS
NM_025690	*Sltm*	D2	4.51E-02	-1.3	2.34E-02	-1.4
NM_008539	*Smad1*	D2	8.41E-04	1.5	NS	NS
NM_001252481	*Smad2*	D2	1.39E-04	-1.7	4.83E-03	-1.6
NM_028534	*Smap1*	D2	9.14E-03	-1.3	NS	NS
NM_008017	*Smc2*	D2	3.64E-02	-1.5	NS	NS
NM_134133	*Smim3*	D2	NS	NS	1.50E-02	2.0
NM_009214	*Sms*	D2	3.30E-02	-1.3	NS	NS
NM_026796	*Smyd2*	D2	4.64E-03	-1.4	NS	NS

NM_144918	*Smyd5*	D2	7.83E-03	-1.6	NS	NS
NM_133968	*Snapc2*	D2	2.92E-02	-1.5	NS	NS
NM_183316	*Snapc5*	D2	3.55E-02	1.4	NS	NS
NM_001190156	*Snx7*	D2	NS	NS	3.47E-02	-1.4
NM_001168655	*Socs2*	D2	7.82E-05	-1.8	NS	NS
NM_001034962	*Sorbs1*	D2	1.91E-02	1.3	NS	NS
NM_146126	*Sord*	D2	4.10E-02	1.5	NS	NS
NM_011447	*Sox8*	D2	1.97E-02	1.8	NS	NS
NM_001025428	*Spag9*	D2	1.34E-02	1.3	NS	NS
NM_001172561	*Sphk2*	D2	2.63E-03	-1.7	NS	NS
NM_182927	*Spred3*	D2	2.42E-06	-2.7	2.95E-05	-2.5
NM_011898	*Spry4*	D2	2.27E-07	-2.6	4.25E-05	-2.2
NM_029035	*Spsb1*	D2	2.95E-02	1.8	NS	NS
NM_011479	*Sptlc2*	D2	NS	NS	2.23E-02	-1.4
NM_009270	*Sqle*	D2	2.64E-08	-2.1	2.35E-06	-1.9
NM_033218	*Srebf2*	D2	4.19E-04	-1.3	5.27E-03	-1.3
NM_020493	*Srf*	D2	4.54E-02	-1.5	2.39E-02	-1.6
NM_001081037	*Srgap1*	D2	3.16E-05	-1.8	1.29E-04	-1.8
NM_001081011	*Srgap2*	D2	2.76E-02	-1.2	NS	NS
NM_009272	*Srm*	D2	1.92E-05	-1.6	1.53E-02	-1.4
NM_016795	*Srpk1*	D2	4.92E-03	-1.5	2.35E-03	-1.6
NM_001109909	*Srrt*	D2	6.23E-04	-1.6	NS	NS
NM_011358	*Srsf2*	D2	4.71E-07	-1.9	1.67E-03	-1.5
NM_001195485	*Srsf7*	D2	6.03E-04	-1.8	1.34E-02	-1.6
NM_178750	*Ss18l1*	D2	7.31E-05	-1.6	NS	NS
NM_026155	*Ssr3*	D2	2.47E-03	1.4	NS	NS
NM_020491	*Sssca1*	D2	4.62E-02	-1.4	NS	NS
NM_009177	*St3gal1*	D2	8.43E-03	-2.0	NS	NS
NM_012028	*St6galnac5*	D2	1.94E-04	-1.5	NS	NS
NM_001159745	*St8sia4*	D2	7.72E-03	-1.6	NS	NS
NM_133774	*Stard4*	D2	2.70E-05	-2.1	7.59E-04	-1.9
NM_016737	*Stip1*	D2	NS	NS	3.99E-03	-1.5
NM_022416	*Stk32b*	D2	1.11E-02	-1.6	NS	NS
NM_009133	*Stmn3*	D2	3.89E-02	-1.3	NS	NS
NM_019675	*Stmn4*	D2	4.28E-02	1.4	1.18E-02	1.6
NM_153563	*Strip1*	D2	5.74E-06	-1.6	3.37E-05	-1.5
NM_019719	*Stub1*	D2	2.47E-03	-1.5	NS	NS
NM_001114611	*Stxbp5l*	D2	2.06E-04	1.6	NS	NS

NM_001168290	*Sugp2*	D2	8.59E-04	-1.6	NS	NS
NM_133670	*Sult1a1*	D2	4.23E-02	2.1	NS	NS
NM_019995	*Supt20*	D2	NS	NS	2.81E-03	-1.7
NM_013678	*Surf2*	D2	9.60E-03	-1.4	NS	NS
NM_009300	*Svs4*	D2	5.06E-03	1.8	NS	NS
NM_153399	*Syne1*	D2	5.62E-03	1.5	NS	NS
NM_018803	*Syt10*	D2	8.46E-03	1.9	NS	NS
NM_018804	*Syt11*	D2	1.65E-02	1.3	NS	NS
NM_001285857	*Syt8*	D2	1.54E-02	1.6	NS	NS
NM_001040085	*Sytl2*	D2	1.09E-03	1.9	1.12E-02	1.8
NM_025609	*Tab1*	D2	2.43E-02	-1.5	NS	NS
NM_030245	*Tada1*	D2	7.37E-03	-1.5	NS	NS
NM_001177798	*Taf6l*	D2	2.82E-02	-1.6	NS	NS
NM_033074	*Tars*	D2	1.11E-02	-1.4	NS	NS
NM_009321	*Tbca*	D2	4.62E-02	-1.4	NS	NS
NM_025548	*Tbcb*	D2	9.84E-03	-1.4	NS	NS
NM_001289689	*Tbpl2*	D2	NS	NS	2.87E-02	2.0
NM_146008	*Tcp11l2*	D2	4.28E-02	1.5	NS	NS
NM_028354	*Tdp1*	D2	1.82E-02	-1.3	NS	NS
NM_001163661	*Telo2*	D2	2.99E-02	-1.5	NS	NS
NM_011571	*Tesk1*	D2	4.75E-04	-1.4	NS	NS
NM_001289603	*Tfcp2*	D2	NS	NS	1.58E-02	-1.5
NM_001289550	*Tgfb1i1*	D2	1.01E-02	-1.7	1.37E-03	-2.0
NM_009381	*Thrsp*	D2	NS	NS	2.63E-02	1.7
NM_145585	*Thumpd1*	D2	3.98E-02	-1.4	NS	NS
NM_174989	*Ticam1*	D2	NS	NS	3.78E-03	2.1
NM_177003	*Tigar*	D2	2.32E-03	-1.8	2.17E-02	-1.7
NM_011591	*Timm17b*	D2	1.14E-02	-1.5	NS	NS
NM_205820	*Tlr13*	D2	2.50E-03	2.6	NS	NS
NM_133211	*Tlr7*	D2	1.27E-04	2.5	NS	NS
NM_026617	*Tmbim4*	D2	2.15E-02	1.3	NS	NS
NM_178638	*Tmem108*	D2	2.15E-02	-1.4	2.62E-02	-1.5
NM_001039723	*Tmem120b*	D2	4.59E-02	-1.5	NS	NS
NM_027495	*Tmem144*	D2	1.40E-02	1.6	NS	NS
NM_011626	*Tmem165*	D2	2.34E-02	-1.3	NS	NS
NM_172049	*Tmem18*	D2	NS	NS	6.01E-03	1.4
NM_001160385	*Tmem196*	D2	4.10E-05	2.1	NS	NS
NM_177344	*Tmem203*	D2	6.58E-03	-1.7	NS	NS

NM_144525	*Tmem214*	D2	3.83E-02	-1.3	2.90E-02	-1.4
NM_001162901	*Tmem217*	D2	3.88E-02	1.6	NS	NS
NM_029748	*Tmem234*	D2	1.34E-02	-1.5	NS	NS
NM_029384	*Tmem238*	D2	1.06E-02	-1.6	NS	NS
NM_199305	*Tmem39b*	D2	2.99E-02	-1.5	NS	NS
NM_028766	*Tmem43*	D2	NS	NS	3.04E-02	1.4
NM_001033394	*Tmem88b*	D2	3.99E-02	2.1	NS	NS
NM_133706	*Tmem97*	D2	5.63E-05	-2.1	2.35E-02	-1.7
NM_029537	*Tmem98*	D2	2.71E-02	1.8	NS	NS
NM_027902	*Tmprss6*	D2	3.55E-02	1.7	NS	NS
NM_011607	*Tnc*	D2	3.70E-05	-1.9	4.05E-03	-1.6
NM_001033535	*Tnfaip8l3*	D2	NS	NS	2.43E-02	-1.6
NM_001161746	*Tnfrsf12a*	D2	4.04E-02	1.6	NS	NS
NM_001048267	*Tnpo1*	D2	1.09E-03	1.5	7.15E-03	1.4
NM_001109748	*Tomm40*	D2	1.76E-04	-1.5	NS	NS
NM_026485	*Trabd*	D2	2.07E-02	-1.5	NS	NS
NM_021502	*Trappc2l*	D2	1.16E-02	-1.5	NS	NS
NM_021789	*Trappc4*	D2	2.40E-03	-1.5	NS	NS
NM_001164641	*Trappc9*	D2	2.39E-05	-1.6	NS	NS
NM_144551	*Trib2*	D2	1.19E-02	-1.5	NS	NS
NM_009054	*Trim27*	D2	4.51E-02	-1.4	NS	NS
NM_029979	*Trim35*	D2	7.59E-03	-1.4	NS	NS
NM_001170855	*Trim36*	D2	6.63E-04	-2.4	NS	NS
NM_001039466	*Trim46*	D2	2.23E-04	-1.7	NS	NS
NM_178110	*Trim62*	D2	1.00E-03	-1.7	1.93E-02	-1.5
NM_198632	*Trim67*	D2	2.12E-03	-1.8	NS	NS
NM_001167994	*Trmt2b*	D2	7.85E-03	1.8	NS	NS
NM_001099792	*Trmt61a*	D2	1.44E-02	-1.6	NS	NS
NM_001127233	*Trp53*	D2	3.10E-03	-1.5	6.06E-03	-1.5
NM_013735	*Trp53bp1*	D2	8.06E-03	-1.5	NS	NS
NM_173378	*Trp53bp2*	D2	1.63E-02	1.5	NS	NS
NM_012035	*Trpc7*	D2	1.21E-02	-2.0	NS	NS
NM_001077364	*Tsc22d3*	D2	4.87E-02	1.5	1.05E-02	1.8
NM_001024619	*Tsku*	D2	1.63E-02	-1.8	NS	NS
NM_145928	*Tspan14*	D2	3.27E-02	-1.3	NS	NS
NM_197996	*Tspan15*	D2	2.85E-03	1.7	NS	NS
NM_025982	*Tspan31*	D2	3.46E-02	1.3	NS	NS
NM_001252588	*Tspan4*	D2	5.34E-03	-1.6	NS	NS

NM_028417	*Ttc9b*	D2	1.21E-02	-1.7	NS	NS
NM_023279	*Tubb3*	D2	3.01E-03	-1.4	NS	NS
NM_146116	*Tubb4b*	D2	1.58E-05	-1.6	1.60E-02	-1.4
NM_011655	*Tubb5*	D2	1.57E-02	-1.2	NS	NS
NM_172597	*Txndc16*	D2	1.19E-02	1.4	NS	NS
NM_001009935	*Txnip*	D2	8.13E-07	3.9	7.99E-05	3.1
NM_001163769	*U2af1*	D2	7.91E-03	-1.5	NS	NS
NM_133806	*Uap1*	D2	1.34E-02	-1.3	NS	NS
NM_176860	*Ubash3b*	D2	1.19E-02	-1.6	NS	NS
NM_026454	*Ube2f*	D2	3.18E-02	-1.3	NS	NS
NM_001169576	*Ube2h*	D2	1.82E-05	1.7	6.49E-03	1.4
NM_001168469	*Ube2m*	D2	3.50E-02	-1.2	NS	NS
NM_001145162	*Ube2ql1*	D2	2.36E-06	-1.5	3.87E-03	-1.4
NM_030724	*Uck2*	D2	NS	NS	2.11E-03	-1.4
NM_031250	*Ucn3*	D2	2.41E-02	1.6	NS	NS
NM_011673	*Ugcg*	D2	NS	NS	1.85E-02	-1.3
NM_011674	*Ugt8a*	D2	2.79E-02	1.8	NS	NS
NM_029770	*Unc5b*	D2	1.70E-02	-1.4	NS	NS
NM_025710	*Uqcrfs1*	D2	3.55E-02	-1.3	NS	NS
NM_009462	*Usp10*	D2	1.57E-03	-1.4	3.87E-03	-1.4
NM_183225	*Usp24*	D2	1.45E-02	-1.4	NS	NS
NM_019461	*Usp27x*	D2	2.41E-02	-1.5	NS	NS
NM_173754	*Usp43*	D2	2.67E-04	-2.1	NS	NS
NM_152825	*Usp45*	D2	2.95E-02	1.6	NS	NS
NM_030180	*Usp54*	D2	4.12E-03	1.7	NS	NS
NM_001013375	*Utp18*	D2	6.03E-04	-1.6	NS	NS
NM_175158	*Utp20*	D2	7.83E-03	-1.7	1.10E-02	-1.7
NM_146216	*Vac14*	D2	2.28E-02	-1.4	3.22E-03	-1.5
NM_013933	*Vapa*	D2	3.23E-02	-1.2	NS	NS
NM_177354	*Vash1*	D2	3.97E-02	-1.6	NS	NS
NM_001282021	*Vasp*	D2	3.51E-02	-1.6	NS	NS
NM_012037	*Vat1*	D2	5.92E-05	-1.8	NS	NS
NM_009500	*Vav2*	D2	1.97E-03	-1.6	NS	NS
NM_001185164	*Vegfb*	D2	3.06E-02	-1.5	NS	NS
NM_001039385	*Vgf*	D2	2.40E-06	-2.9	8.81E-03	-2.0
NM_001104623	*Vmn2r12*	D2	4.56E-02	1.7	NS	NS
NM_029478	*Vmp1*	D2	7.75E-03	1.4	NS	NS
NM_145967	*Vstm2a*	D2	NS	NS	8.57E-03	-1.3

NM_027906	*Vwa8*	D2	4.97E-02	1.4	NS	NS
NM_175450	*Wdr18*	D2	1.35E-02	-1.5	NS	NS
NM_172372	*Wdr45*	D2	3.56E-02	1.5	NS	NS
NM_020603	*Wdr46*	D2	2.74E-02	-1.6	1.68E-02	-1.7
NM_134139	*Wdr74*	D2	3.18E-02	-1.4	1.90E-02	-1.4
NM_001011874	*Xkr4*	D2	1.94E-02	1.4	3.10E-02	1.4
NM_001011732	*Xkr7*	D2	4.22E-02	-1.6	NS	NS
NM_001081642	*Xlr4a*	D2	3.49E-02	1.7	NS	NS
NM_001035226	*Xpo1*	D2	NS	NS	3.76E-02	1.4
NM_175645	*Xylt1*	D2	4.54E-02	-1.3	NS	NS
NM_026940	*Ydjc*	D2	4.22E-02	-1.6	NS	NS
NM_001005342	*Ypel4*	D2	3.54E-06	3.0	5.26E-05	2.7
NM_027166	*Ypel5*	D2	NS	NS	6.14E-03	1.4
NM_153566	*Yrdc*	D2	7.59E-03	-1.7	NS	NS
NM_001163013	*Ythdc2*	D2	2.97E-03	-1.7	NS	NS
NM_173761	*Ythdf1*	D2	3.18E-02	-1.5	NS	NS
NM_028106	*Zbed3*	D2	2.01E-02	1.4	NS	NS
NM_001033324	*Zbtb16*	D2	1.28E-03	2.0	NS	NS
NM_001033466	*Zbtb2*	D2	1.69E-02	-1.5	NS	NS
NM_001100460	*Zbtb42*	D2	2.69E-02	1.8	NS	NS
NM_001115130	*Zbtb44*	D2	1.13E-02	1.4	NS	NS
NM_173181	*Zc2hc1a*	D2	3.71E-02	-1.3	5.17E-03	-1.4
NM_029888	*Zfp142*	D2	9.60E-03	-1.4	NS	NS
NM_001253694	*Zfp219*	D2	2.82E-02	-1.6	NS	NS
NM_175247	*Zfp28*	D2	1.55E-02	-1.8	NS	NS
NM_013866	*Zfp385a*	D2	3.36E-02	-1.5	NS	NS
NM_001110309	*Zfp426*	D2	1.84E-03	1.6	3.78E-03	1.6
NM_001013777	*Zfp488*	D2	8.06E-03	2.0	NS	NS
NM_207255	*Zfp532*	D2	2.88E-03	1.5	1.64E-02	1.4
NM_172385	*Zfp536*	D2	4.33E-02	-1.5	NS	NS
NM_026741	*Zfp579*	D2	4.25E-02	-1.5	NS	NS
NM_001145999	*Zfp710*	D2	4.64E-02	-1.5	NS	NS
NM_001267583	*Zfp811*	D2	8.13E-03	1.5	2.78E-02	1.5
NM_146231	*Zfp825*	D2	3.84E-02	1.3	NS	NS
NM_173387	*Zfp879*	D2	1.01E-02	1.6	NS	NS
NM_001105557	*Zfp938*	D2	2.12E-02	1.4	NS	NS
NM_001177985	*Zmym3*	D2	NS	NS	2.90E-02	-1.5
NM_001252584	*Zmynd8*	D2	2.04E-04	1.8	2.36E-03	1.7

NM_028028	*Zswim1*	D2	1.08E-02	-1.5	NS	NS
NM_001033634	*Zyg11b*	D2	NS	NS	4.73E-02	-1.2
NM_175149	*2310022B05Rik*	BXD2	3.68E-03	1.7	NS	NS
NR_045177	*2900055J20Rik*	BXD2	NS	NS	3.62E-02	1.6
NM_001167925	*A730017C20Rik*	BXD2	NS	NS	6.37E-03	1.3
NR_015488	*A930003A15Rik*	BXD2	NS	NS	4.78E-02	1.8
NR_033609	*A930017M01Rik*	BXD2	3.79E-03	1.8	NS	NS
NM_030210	*Aacs*	BXD2	2.20E-03	-1.6	1.24E-02	-1.6
NM_013851	*Abca8b*	BXD2	1.76E-03	2.1	NS	NS
NM_153151	*Acat3*	BXD2	NS	NS	3.50E-02	-1.4
NM_001276719	*Ackr2*	BXD2	NS	NS	2.05E-02	-2.0
NM_001199296	*Acly*	BXD2	NS	NS	1.03E-02	-1.4
NM_001205385	*Actr3*	BXD2	NS	NS	4.60E-02	-1.2
NM_178162	*Agfg2*	BXD2	2.39E-02	-1.7	NS	NS
NM_001172146	*Aimp2*	BXD2	4.92E-02	-1.6	NS	NS
NM_025538	*Alkbh7*	BXD2	2.05E-02	-1.5	NS	NS
NM_019764	*Amotl2*	BXD2	NS	NS	4.54E-03	1.9
NM_001276301	*Ampd3*	BXD2	1.46E-02	1.7	NS	NS
NM_020581	*Angptl4*	BXD2	7.69E-03	2.0	NS	NS
NM_026674	*Aph1c*	BXD2	2.93E-02	1.5	NS	NS
NM_023598	*Arid5b*	BXD2	1.57E-04	1.8	NS	NS
NM_001042591	*Arrdc3*	BXD2	3.11E-06	2.5	1.87E-03	2.0
NM_001042592	*Arrdc4*	BXD2	2.17E-02	1.7	NS	NS
NM_026217	*Atg12*	BXD2	1.90E-03	1.4	NS	NS
NM_001199304	*Atxn1*	BXD2	4.90E-02	1.4	NS	NS
NM_016847	*Avpr1a*	BXD2	NS	NS	4.34E-03	-2.0
NM_001122993	*B3galt5*	BXD2	5.39E-03	1.7	NS	NS
NM_001169114	*B3gnt2*	BXD2	1.07E-02	1.7	NS	NS
NM_001159407	*B3gnt5*	BXD2	7.63E-03	1.7	NS	NS
NM_019835	*B4galt5*	BXD2	NS	NS	4.06E-03	-1.6
NM_175325	*Bbs4*	BXD2	1.65E-02	1.3	NS	NS
NM_001284410	*Bcl2l11*	BXD2	7.99E-05	2.4	1.88E-03	2.2
NM_029933	*Bcl9*	BXD2	2.73E-02	-1.3	NS	NS
NM_001141922	*Bean1*	BXD2	NS	NS	3.79E-02	-1.4
NM_173404	*Bmp3*	BXD2	1.85E-02	-1.8	NS	NS
NM_007570	*Btg2*	BXD2	6.13E-06	2.3	1.30E-02	1.8
NM_009779	*C3ar1*	BXD2	NS	NS	2.78E-02	1.8
NM_144817	*Camk1g*	BXD2	2.70E-02	-1.4	NS	NS

NM_177716	*Ccdc184*	BXD2	2.76E-02	-1.5	NS	NS
NM_144820	*Ccdc28a*	BXD2	4.92E-02	1.8	NS	NS
NM_007681	*Cenpa*	BXD2	2.80E-02	1.7	NS	NS
NM_001159364	*Cep97*	BXD2	3.44E-02	1.6	NS	NS
NM_001081345	*Chd2*	BXD2	3.73E-02	1.4	NS	NS
NM_025854	*Cir1*	BXD2	1.51E-03	1.5	NS	NS
NM_001243762	*Clcn5*	BXD2	2.76E-02	1.6	2.05E-02	1.7
NM_024217	*Cmtm3*	BXD2	NS	NS	2.82E-02	1.4
NM_001163126	*Cog5*	BXD2	4.21E-02	1.4	NS	NS
NM_009898	*Coro1a*	BXD2	4.15E-02	-1.6	NS	NS
NM_001252525	*Cpeb1*	BXD2	1.06E-02	2.0	NS	NS
NM_172728	*Creb5*	BXD2	3.79E-03	2.0	NS	NS
NM_001110850	*Crem*	BXD2	2.89E-04	2.0	NS	NS
NM_007762	*Crhr1*	BXD2	1.07E-04	-2.2	2.82E-02	-1.7
NM_016748	*Ctps*	BXD2	NS	NS	4.57E-02	-1.5
NM_009994	*Cyp1b1*	BXD2	NS	NS	2.78E-02	2.2
NM_028979	*Cyp2j9*	BXD2	3.38E-03	1.9	4.57E-02	1.7
NM_001285947	*Cyp39a1*	BXD2	3.99E-02	1.8	NS	NS
NM_020010	*Cyp51*	BXD2	2.07E-03	-1.5	1.23E-03	-1.5
NM_010516	*Cyr61*	BXD2	NS	NS	1.86E-02	1.8
NM_001252457	*Ddx39b*	BXD2	4.92E-02	-1.3	NS	NS
NM_172733	*Dera*	BXD2	4.48E-03	1.9	NS	NS
NM_053272	*Dhcr24*	BXD2	1.83E-10	-1.9	5.57E-03	-1.4
NM_001163026	*Dnajc13*	BXD2	1.43E-02	-1.5	NS	NS
NM_009584	*Dnajc2*	BXD2	2.70E-02	-1.4	2.67E-02	-1.5
NM_001285807	*Dtna*	BXD2	4.11E-04	1.4	NS	NS
NM_013642	*Dusp1*	BXD2	3.74E-08	2.5	1.46E-06	2.3
NM_001048054	*Dusp16*	BXD2	1.62E-03	2.0	1.36E-02	1.8
NM_008748	*Dusp8*	BXD2	3.77E-06	1.5	6.93E-03	1.4
NM_010104	*Edn1*	BXD2	2.70E-02	2.4	NS	NS
NM_001289925	*Egr3*	BXD2	1.27E-02	-1.8	1.41E-02	-1.9
NM_198303	*Eif5b*	BXD2	NS	NS	3.24E-02	-1.4
NM_001253692	*Elmod3*	BXD2	3.72E-03	1.6	2.05E-02	1.5
NM_130450	*Elovl6*	BXD2	NS	NS	2.05E-02	-1.4
NM_023794	*Etv5*	BXD2	1.86E-02	-1.6	NS	NS
NM_183187	*Fam107a*	BXD2	3.40E-07	3.9	1.46E-03	2.6
NM_178908	*Fam26e*	BXD2	4.92E-02	1.6	5.70E-03	1.8
NM_175104	*Fam53c*	BXD2	4.73E-04	2.0	2.66E-02	1.7

NM_178797	*Far2*	BXD2	NS	NS	1.70E-03	-1.5
NM_026346	*Fbxo32*	BXD2	8.64E-03	2.1	NS	NS
NM_010191	*Fdft1*	BXD2	1.35E-07	-1.6	1.34E-06	-1.6
NM_001253751	*Fdps*	BXD2	2.85E-02	-1.5	NS	NS
NM_001163215	*Fgfr3*	BXD2	6.85E-04	1.9	1.41E-02	1.7
NM_001164259	*Fgfrl1*	BXD2	3.32E-02	-1.5	NS	NS
NM_022009	*Flii*	BXD2	NS	NS	1.26E-02	-1.6
NM_001159706	*Folh1*	BXD2	1.71E-02	2.0	NS	NS
NM_028058	*Fundc1*	BXD2	3.73E-02	1.4	NS	NS
NM_028022	*Gatsl3*	BXD2	2.80E-02	2.0	4.57E-02	2.0
ENSMUST0000 0178468	*Gm21907*	BXD2	1.68E-02	2.6	NS	NS
NM_001035122	*Golm1*	BXD2	3.98E-02	1.5	NS	NS
NM_027518	*Gpr137c*	BXD2	9.10E-08	2.1	1.14E-08	2.3
NM_010338	*Gpr37*	BXD2	7.38E-03	1.7	NS	NS
NM_010287	*Gpr83*	BXD2	2.90E-02	2.1	NS	NS
NM_145066	*Gpr85*	BXD2	4.21E-02	1.4	NS	NS
NM_153419	*Grwd1*	BXD2	3.09E-02	-1.8	NS	NS
NM_008185	*Gstt1*	BXD2	NS	NS	2.20E-02	1.9
NM_198962	*Hcrtr2*	BXD2	3.69E-02	-1.7	NS	NS
NM_010422	*Hexb*	BXD2	NS	NS	4.57E-02	1.4
NM_008252	*Hmgb2*	BXD2	8.40E-04	1.9	NS	NS
NM_008255	*Hmgcr*	BXD2	3.40E-07	-2.0	5.33E-03	-1.6
NM_013751	*Hrasls*	BXD2	4.68E-02	1.8	NS	NS
NM_053262	*Hsd17b11*	BXD2	2.49E-02	1.5	NS	NS
NM_010476	*Hsd17b7*	BXD2	6.62E-07	-2.0	1.13E-05	-1.9
NM_001002012	*Hspa2*	BXD2	3.79E-03	1.8	NS	NS
NM_008300	*Hspa4*	BXD2	2.70E-02	-1.3	NS	NS
NM_019564	*Htra1*	BXD2	7.52E-03	1.7	NS	NS
NM_031166	*Id4*	BXD2	1.65E-02	2.1	NS	NS
NM_145360	*Idi1*	BXD2	1.20E-07	-2.1	4.36E-07	-2.1
NM_001162884	*Igsf10*	BXD2	NS	NS	2.82E-02	1.7
NM_008360	*Il18*	BXD2	1.02E-02	1.4	NS	NS
NM_016671	*Il27ra*	BXD2	1.85E-03	-1.9	NS	NS
NM_023626	*Ing3*	BXD2	1.49E-04	1.6	NS	NS
NM_153526	*Insig1*	BXD2	1.66E-07	-2.4	1.46E-06	-2.3
NM_010591	*Jun*	BXD2	8.98E-14	3.4	1.93E-09	2.7
NM_001286944	*Jund*	BXD2	2.58E-04	1.7	5.16E-03	1.6

NM_001008785	*Kbtbd8*	BXD2	3.19E-02	-1.9	NS	NS
NM_001081134	*Kcng1*	BXD2	4.73E-04	-2.1	4.87E-02	-1.7
NM_172898	*Kirrel2*	BXD2	1.13E-02	1.9	NS	NS
NM_078477	*Klf16*	BXD2	1.76E-03	-1.7	2.14E-02	-1.6
NM_008457	*Klk1b8*	BXD2	NS	NS	1.95E-02	1.7
NM_010655	*Kpna2*	BXD2	NS	NS	3.18E-02	-1.5
NM_001252658	*Ldlr*	BXD2	2.07E-05	-2.5	3.02E-05	-2.5
NM_008494	*Lfng*	BXD2	3.72E-03	1.6	NS	NS
NM_001113545	*Lima1*	BXD2	3.32E-02	1.6	NS	NS
NM_144862	*Lims2*	BXD2	4.49E-02	1.9	NS	NS
NM_177250	*Lingo4*	BXD2	1.73E-02	1.5	NS	NS
NM_201529	*Lmo7*	BXD2	4.61E-02	1.4	NS	NS
NM_181470	*Ltv1*	BXD2	1.73E-02	-1.7	NS	NS
NM_001252055	*Ly6c1*	BXD2	4.96E-02	2.0	NS	NS
NM_001045483	*Mapk1ip1*	BXD2	5.39E-03	1.4	3.29E-02	1.4
NM_016977	*Mc4r*	BXD2	1.10E-02	2.4	NS	NS
NM_008587	*Mertk*	BXD2	4.23E-02	1.9	2.82E-02	2.0
NM_026197	*Mettl16*	BXD2	NS	NS	2.82E-02	-1.6
NM_001252094	*Mettl20*	BXD2	2.76E-02	2.0	NS	NS
NM_029662	*Mfsd2a*	BXD2	3.44E-02	1.6	NS	NS
NM_001243584	*Mif4gd*	BXD2	1.96E-03	1.9	NS	NS
NM_025436	*Msmo1*	BXD2	6.62E-07	-2.4	4.36E-07	-2.5
NM_013602	*Mt1*	BXD2	3.62E-04	1.7	2.47E-04	1.7
NM_008630	*Mt2*	BXD2	2.35E-06	2.7	1.48E-05	2.6
NM_178051	*Mterf4*	BXD2	1.88E-02	1.6	NS	NS
NM_138656	*Mvd*	BXD2	1.84E-05	-2.2	2.45E-06	-2.4
NM_001008542	*Mxi1*	BXD2	8.64E-07	1.8	4.89E-05	1.6
NM_001162417	*Myef2*	BXD2	NS	NS	4.32E-02	-1.3
NM_001040395	*Nadk2*	BXD2	5.03E-05	1.6	NS	NS
NM_023239	*Ndnl2*	BXD2	7.47E-03	1.8	NS	NS
NM_001145959	*Ndrg2*	BXD2	NS	NS	2.78E-02	1.3
NM_026742	*Ndufaf4*	BXD2	9.35E-03	-1.5	NS	NS
NM_028757	*Nebl*	BXD2	2.93E-02	1.7	NS	NS
NM_145469	*Nipal2*	BXD2	3.67E-02	1.5	NS	NS
NM_001033367	*Nlrc4*	BXD2	4.68E-02	1.7	NS	NS
NM_138314	*Nme7*	BXD2	1.03E-02	1.5	NS	NS
NM_010941	*Nsdhl*	BXD2	NS	NS	3.62E-04	-1.5
NM_001161430	*Nxt2*	BXD2	2.80E-02	1.4	NS	NS

NM_178644	*Oaf*	BXD2	6.09E-03	2.2	1.59E-02	2.1
NM_146921	*Olfr1*	BXD2	4.11E-02	-1.7	NS	NS
NM_146742	*Olfr1316*	BXD2	NS	NS	6.77E-03	-2.1
NM_146583	*Olfr52*	BXD2	1.43E-03	-2.1	NS	NS
NM_146955	*Olfr60*	BXD2	NS	NS	3.56E-02	-1.8
NM_019409	*Omg*	BXD2	1.43E-02	1.4	NS	NS
NM_001134791	*Osbpl9*	BXD2	1.07E-04	1.5	7.38E-05	1.5
NM_028829	*Paqr8*	BXD2	2.76E-02	1.4	4.87E-02	1.4
NM_001177980	*Pde4b*	BXD2	1.51E-02	1.4	NS	NS
NM_025954	*Pgp*	BXD2	NS	NS	4.81E-02	-1.5
NM_178149	*Pik3ip1*	BXD2	2.63E-06	2.4	NS	NS
NM_001024955	*Pik3r1*	BXD2	1.38E-08	1.8	2.12E-06	1.6
NM_181585	*Pik3r3*	BXD2	4.79E-03	1.5	NS	NS
NM_001033253	*Plekhg1*	BXD2	1.30E-04	1.9	9.22E-05	2.0
NM_013807	*Plk3*	BXD2	3.64E-03	-1.7	6.44E-03	-1.7
NM_133249	*Ppargc1b*	BXD2	NS	NS	4.57E-02	-1.7
NM_145404	*Prmt7*	BXD2	4.80E-02	-1.4	NS	NS
NM_201371	*Prmt8*	BXD2	NS	NS	1.26E-02	-1.3
NM_011201	*Ptpn1*	BXD2	7.63E-03	-1.4	NS	NS
NM_133198	*Pygl*	BXD2	NS	NS	2.82E-02	1.9
NM_029494	*Rab30*	BXD2	2.93E-06	1.9	6.77E-03	1.6
NM_001038621	*Rabgap1l*	BXD2	1.05E-03	1.4	1.88E-02	1.3
NM_001099624	*Rapgef2*	BXD2	2.50E-06	1.6	1.67E-06	1.7
NM_001252494	*Rapgef6*	BXD2	1.19E-06	2.1	1.47E-03	1.7
NM_009025	*Rasa3*	BXD2	2.60E-02	-1.5	NS	NS
NM_178045	*Rassf4*	BXD2	NS	NS	1.91E-02	1.8
NM_001081549	*Rcan1*	BXD2	2.76E-02	1.4	NS	NS
NM_153514	*Rhobtb2*	BXD2	NS	NS	2.81E-02	-1.5
NM_030259	*Rilpl2*	BXD2	3.73E-02	1.6	NS	NS
NM_001081977	*Rnf144a*	BXD2	3.64E-02	1.4	NS	NS
NM_001166553	*Rnf145*	BXD2	2.07E-03	-1.5	NS	NS
NM_001163461	*Rpap2*	BXD2	1.72E-02	1.5	NS	NS
NM_133982	*Rpp25*	BXD2	7.61E-03	-1.7	1.30E-03	-1.9
NM_146244	*Rps6kl1*	BXD2	6.78E-07	2.4	5.16E-03	1.8
NM_007901	*S1pr1*	BXD2	4.23E-02	1.6	NS	NS
NM_172769	*Sc5d*	BXD2	NS	NS	2.05E-02	-1.3
NM_172604	*Scara3*	BXD2	NS	NS	4.32E-02	1.5
NM_009127	*Scd1*	BXD2	4.23E-02	-1.5	NS	NS

NM_009129	*Scg2*	BXD2	6.30E-03	1.4	NS	NS
NM_011521	*Sdc4*	BXD2	1.16E-02	2.0	1.36E-02	2.0
NM_011342	*Sec22b*	BXD2	4.90E-02	1.4	NS	NS
NM_030261	*Sesn3*	BXD2	4.79E-03	1.5	1.25E-02	1.4
NM_009167	*Shc3*	BXD2	5.35E-04	-1.5	NS	NS
NM_013612	*Slc11a1*	BXD2	NS	NS	8.62E-03	2.0
NM_027921	*Slc16a14*	BXD2	3.79E-03	1.6	NS	NS
NM_001159593	*Slc20a1*	BXD2	9.74E-04	-1.5	NS	NS
NM_011400	*Slc2a1*	BXD2	7.38E-03	1.8	4.07E-02	1.6
NM_001012305	*Slc39a12*	BXD2	NS	NS	1.42E-02	1.7
NM_001199283	*Slc43a2*	BXD2	NS	NS	3.83E-02	1.5
NM_001081263	*Slc44a5*	BXD2	1.38E-05	1.9	1.30E-02	1.5
NM_001038643	*Slco3a1*	BXD2	7.69E-03	-1.5	NS	NS
NM_001177833	*Smox*	BXD2	2.52E-02	1.5	NS	NS
NM_183316	*Snapc5*	BXD2	1.10E-02	1.6	NS	NS
NM_009238	*Sox4*	BXD2	3.17E-02	1.5	3.62E-02	1.5
NM_182927	*Spred3*	BXD2	7.42E-04	-2.2	4.47E-03	-2.1
NM_011898	*Spry4*	BXD2	1.39E-02	-1.8	2.82E-02	-1.7
NM_009270	*Sqle*	BXD2	1.29E-04	-1.7	8.50E-06	-1.8
NM_011358	*Srsf2*	BXD2	2.27E-02	-1.4	1.95E-02	-1.5
NM_001195485	*Srsf7*	BXD2	3.17E-02	-1.6	2.19E-02	-1.6
NM_133774	*Stard4*	BXD2	9.74E-04	-1.9	4.89E-05	-2.1
NM_001102423	*Stx16*	BXD2	4.93E-02	1.4	NS	NS
NM_001040085	*Sytl2*	BXD2	3.67E-02	1.7	NS	NS
NM_001184706	*Tfdp2*	BXD2	4.43E-02	1.6	NS	NS
NM_026436	*Tmem86a*	BXD2	NS	NS	4.57E-02	1.6
NM_001048267	*Tnpo1*	BXD2	4.23E-02	1.4	2.05E-02	1.4
NM_001109748	*Tomm40*	BXD2	4.19E-02	-1.4	1.71E-03	-1.5
NM_001170488	*Tprkb*	BXD2	4.92E-02	1.5	NS	NS
NM_001243916	*Trim34b*	BXD2	3.06E-02	1.7	NS	NS
NM_001170912	*Trim66*	BXD2	4.31E-02	2.1	NS	NS
NM_001099792	*Trmt61a*	BXD2	NS	NS	3.31E-02	-1.6
NM_172597	*Txndc16*	BXD2	7.63E-03	1.5	NS	NS
NM_001009935	*Txnip*	BXD2	2.05E-05	3.4	6.43E-05	3.3
NM_133806	*Uap1*	BXD2	4.80E-02	-1.3	NS	NS
NM_001169576	*Ube2h*	BXD2	2.30E-03	1.5	5.16E-03	1.5
NM_001145162	*Ube2ql1*	BXD2	NS	NS	2.09E-02	-1.3
NM_030724	*Uck2*	BXD2	2.32E-03	-1.4	NS	NS

NM_175158	*Utp20*	BXD2	NS	NS	2.82E-02	-1.7
NM_001039385	*Vgf*	BXD2	9.50E-03	-2.1	3.83E-02	-1.9
NM_001005342	*Ypel4*	BXD2	6.22E-04	2.5	4.45E-03	2.3
NM_027166	*Ypel5*	BXD2	7.14E-03	1.4	NS	NS
NM_028106	*Zbed3*	BXD2	4.31E-02	1.5	NS	NS
NM_001115130	*Zbtb44*	BXD2	9.55E-03	1.4	NS	NS
NM_178404	*Zc3h6*	BXD2	7.99E-03	1.5	NS	NS
NM_144546	*Zfp119a*	BXD2	4.20E-02	1.5	NS	NS
NM_001110309	*Zfp426*	BXD2	2.76E-02	1.5	NS	NS
NM_001267583	*Zfp811*	BXD2	1.51E-02	1.6	NS	NS
NM_001105557	*Zfp938*	BXD2	3.44E-02	1.4	NS	NS
NM_001252584	*Zmynd8*	BXD2	1.77E-02	1.6	NS	NS
NM_029598	*1700019B03Rik*	BXD48a	4.63E-02	1.6	NS	NS
NM_001033630	*2310061I04Rik*	BXD48a	4.42E-02	-1.4	NS	NS
NR_045177	*2900055J20Rik*	BXD48a	3.77E-02	1.5	9.99E-03	1.6
NM_026077	*3110040N11Rik*	BXD48a	NS	NS	5.00E-02	1.5
NR_015591	*5430405H02Rik*	BXD48a	1.59E-03	2.2	NS	NS
NR_015488	*A930003A15Rik*	BXD48a	2.46E-02	1.9	NS	NS
NM_030210	*Aacs*	BXD48a	NS	NS	7.83E-03	-1.6
NM_001166556	*Abca6*	BXD48a	1.61E-03	2.6	NS	NS
NM_013851	*Abca8b*	BXD48a	NS	NS	6.12E-03	2.0
NM_011076	*Abcb1a*	BXD48a	NS	NS	3.06E-02	1.7
NM_001276719	*Ackr2*	BXD48a	NS	NS	2.91E-02	-1.9
NM_001199296	*Acly*	BXD48a	NS	NS	3.29E-02	-1.3
NM_001102404	*Acp5*	BXD48a	NS	NS	1.06E-02	-3.4
NM_007981	*Acsl1*	BXD48a	5.57E-03	1.7	NS	NS
NM_020330	*Adam21*	BXD48a	NS	NS	8.74E-03	-2.1
NM_001270996	*Adam9*	BXD48a	NS	NS	4.64E-02	1.3
NM_172133	*Adap2*	BXD48a	4.91E-03	1.7	NS	NS
NM_133222	*Adgrl4*	BXD48a	NS	NS	1.63E-02	1.4
NM_026644	*Agpat4*	BXD48a	2.37E-02	1.4	NS	NS
NM_001172146	*Aimp2*	BXD48a	6.82E-03	-1.8	1.17E-03	-1.9
NM_001035532	*Akap2*	BXD48a	3.50E-03	1.6	NS	NS
NM_025538	*Alkbh7*	BXD48a	4.47E-02	-1.5	NS	NS
NM_019764	*Amotl2*	BXD48a	4.65E-04	2.1	NS	NS
NM_020581	*Angptl4*	BXD48a	1.05E-03	2.3	1.23E-03	2.2
NM_027799	*Ankrd40*	BXD48a	4.98E-02	1.4	NS	NS
NM_001198911	*Arhgef2*	BXD48a	3.23E-02	-1.4	NS	NS

NM_175251	*Arid2*	BXD48a	1.24E-02	1.5	NS	NS
NM_001172205	*Arid5a*	BXD48a	2.14E-02	1.9	2.05E-02	1.9
NM_023598	*Arid5b*	BXD48a	1.89E-03	1.6	1.39E-04	1.8
NM_001042591	*Arrdc3*	BXD48a	5.16E-08	2.9	3.63E-07	2.7
NM_001042592	*Arrdc4*	BXD48a	5.48E-05	2.1	8.41E-03	1.7
NM_009713	*Arsa*	BXD48a	1.20E-02	1.8	NS	NS
NM_026217	*Atg12*	BXD48a	6.28E-06	1.6	1.72E-03	1.4
NM_001199304	*Atxn1*	BXD48a	NS	NS	2.47E-02	1.4
NM_009125	*Atxn2*	BXD48a	NS	NS	3.17E-02	-1.3
NM_016847	*Avpr1a*	BXD48a	NS	NS	5.05E-03	-1.9
NM_001159407	*B3gnt5*	BXD48a	5.68E-04	1.9	2.22E-02	1.6
NM_175325	*Bbs4*	BXD48a	1.20E-02	1.4	NS	NS
NM_001284410	*Bcl2l11*	BXD48a	4.15E-05	2.5	9.55E-05	2.4
NM_001122683	*Bdh1*	BXD48a	1.24E-02	-1.4	3.61E-02	-1.3
NM_133889	*Bsdc1*	BXD48a	NS	NS	5.31E-03	1.4
NM_007570	*Btg2*	BXD48a	1.72E-05	2.2	1.96E-02	1.7
NM_009770	*Btg3*	BXD48a	2.96E-02	1.7	NS	NS
NM_001172096	*Cacul1*	BXD48a	NS	NS	2.72E-02	1.4
NM_144817	*Camk1g*	BXD48a	1.44E-02	-1.5	2.05E-02	-1.4
NM_025821	*Carhsp1*	BXD48a	3.77E-02	1.6	NS	NS
NM_178793	*Ccbe1*	BXD48a	3.50E-03	1.9	NS	NS
NM_009831	*Ccng1*	BXD48a	2.17E-02	1.4	4.96E-03	1.4
NM_007635	*Ccng2*	BXD48a	NS	NS	3.72E-02	1.5
NM_025422	*Cd302*	BXD48a	NS	NS	3.92E-04	2.1
NM_001168304	*Cdk19*	BXD48a	4.41E-03	1.5	NS	NS
NM_009875	*Cdkn1b*	BXD48a	1.90E-03	1.5	NS	NS
NM_001081345	*Chd2*	BXD48a	2.04E-02	1.5	7.35E-03	1.5
NM_007692	*Chkb*	BXD48a	NS	NS	4.25E-02	-1.4
NM_053068	*Chrac1*	BXD48a	3.78E-02	1.7	NS	NS
NM_203491	*Chrm2*	BXD48a	1.36E-02	-1.7	NS	NS
NM_176844	*Chrna5*	BXD48a	3.98E-03	1.9	NS	NS
NM_021528	*Chst12*	BXD48a	2.03E-03	-1.6	NS	NS
NM_001243762	*Clcn5*	BXD48a	1.97E-03	1.8	4.59E-02	1.6
NM_001033242	*Cln5*	BXD48a	1.20E-02	2.0	5.05E-03	2.2
NM_001033175	*Cln6*	BXD48a	NS	NS	4.79E-02	1.5
NM_153384	*Clrn1*	BXD48a	2.51E-03	1.9	NS	NS
NM_001252525	*Cpeb1*	BXD48a	NS	NS	1.63E-02	1.9
NM_172728	*Creb5*	BXD48a	NS	NS	3.56E-02	1.8

NM_029870	*Crebrf*	BXD48a	NS	NS	4.88E-02	1.6
NM_001110850	*Crem*	BXD48a	7.61E-04	1.9	2.42E-04	2.0
NM_007762	*Crhr1*	BXD48a	5.17E-03	-1.9	4.87E-06	-2.5
NM_007773	*Crybb2*	BXD48a	2.96E-02	-1.7	NS	NS
NM_031249	*Cstf2t*	BXD48a	4.98E-02	-1.4	NS	NS
NM_001145799	*Ctla2a*	BXD48a	4.31E-02	1.7	8.42E-04	2.1
NM_007808	*Cycs*	BXD48a	2.99E-02	-1.2	NS	NS
NM_009994	*Cyp1b1*	BXD48a	NS	NS	4.07E-03	2.4
NM_028979	*Cyp2j9*	BXD48a	NS	NS	1.93E-02	1.7
NM_001100181	*Cyp4a32*	BXD48a	NS	NS	1.02E-02	-1.9
NM_010516	*Cyr61*	BXD48a	1.05E-02	1.8	NS	NS
NM_001008231	*Daam2*	BXD48a	NS	NS	2.97E-02	1.5
NM_001142570	*Dach2*	BXD48a	3.77E-02	1.9	NS	NS
NM_001161515	*Dctd*	BXD48a	NS	NS	4.79E-02	-1.6
NM_019553	*Ddx21*	BXD48a	NS	NS	4.79E-02	-1.5
NM_001252457	*Ddx39b*	BXD48a	1.22E-02	-1.3	NS	NS
NM_011933	*Decr2*	BXD48a	4.31E-02	1.4	NS	NS
NM_053272	*Dhcr24*	BXD48a	6.89E-07	-1.7	3.70E-07	-1.7
NM_001030307	*Dkc1*	BXD48a	NS	NS	8.25E-03	-2.4
NR_002854	*Dlx1as*	BXD48a	NS	NS	1.33E-02	-2.0
NM_025926	*Dnajb4*	BXD48a	1.07E-02	1.4	6.50E-03	1.4
NM_025274	*Dppa5a*	BXD48a	2.81E-02	-1.8	NS	NS
NM_001285807	*Dtna*	BXD48a	2.20E-03	1.4	1.23E-03	1.4
NM_172442	*Dtx4*	BXD48a	4.66E-03	1.4	NS	NS
NM_013642	*Dusp1*	BXD48a	9.78E-07	2.3	1.78E-05	2.1
NM_001048054	*Dusp16*	BXD48a	1.15E-04	2.2	2.67E-05	2.3
NM_026268	*Dusp6*	BXD48a	NS	NS	1.86E-03	-1.7
NM_008748	*Dusp8*	BXD48a	5.60E-06	1.5	2.49E-04	1.4
NM_010104	*Edn1*	BXD48a	4.28E-03	2.7	9.87E-03	2.5
NM_010118	*Egr2*	BXD48a	1.43E-03	2.4	NS	NS
NM_010121	*Eif2ak3*	BXD48a	3.77E-02	1.5	NS	NS
NM_007917	*Eif4e*	BXD48a	NS	NS	3.06E-02	-1.3
NM_001253692	*Elmod3*	BXD48a	5.14E-03	1.6	NS	NS
NM_001146348	*Eng*	BXD48a	3.77E-02	1.7	NS	NS
NM_007946	*Epx*	BXD48a	4.50E-03	-2.0	NS	NS
NM_029250	*Etnk1*	BXD48a	NS	NS	3.97E-02	1.4
NM_008815	*Etv4*	BXD48a	NS	NS	1.72E-03	-1.7
NM_023794	*Etv5*	BXD48a	1.19E-03	-1.8	2.38E-02	-1.6

NM_183187	*Fam107a*	BXD48a	1.15E-04	2.9	3.63E-07	4.0
NM_001100458	*Fam169a*	BXD48a	3.77E-02	-1.5	NS	NS
NM_178908	*Fam26e*	BXD48a	1.61E-03	1.9	9.40E-05	2.1
NM_175104	*Fam53c*	BXD48a	6.38E-03	1.8	NS	NS
NM_019643	*Fam60a*	BXD48a	3.41E-02	1.7	NS	NS
NM_178797	*Far2*	BXD48a	6.94E-05	-1.7	NS	NS
NM_026346	*Fbxo32*	BXD48a	2.64E-02	2.0	NS	NS
NM_010191	*Fdft1*	BXD48a	9.78E-07	-1.6	9.55E-05	-1.5
NM_001253751	*Fdps*	BXD48a	NS	NS	4.68E-02	-1.5
NM_008013	*Fgl2*	BXD48a	NS	NS	5.85E-03	1.7
NM_010218	*Fjx1*	BXD48a	3.00E-02	1.3	NS	NS
NM_001276408	*Fn1*	BXD48a	NS	NS	4.79E-02	1.6
NM_173753	*Fnip1*	BXD48a	8.17E-03	1.5	NS	NS
NM_001159706	*Folh1*	BXD48a	4.55E-02	1.9	1.31E-02	2.0
NM_010234	*Fos*	BXD48a	4.10E-03	2.2	6.54E-04	2.4
NM_008036	*Fosb*	BXD48a	3.73E-03	2.5	1.06E-02	2.3
NM_019740	*Foxo3*	BXD48a	2.40E-02	1.6	NS	NS
NM_010236	*Fpgs*	BXD48a	1.32E-02	-1.6	NS	NS
NM_001081151	*Gan*	BXD48a	3.41E-02	1.7	NS	NS
NM_028022	*Gatsl3*	BXD48a	3.00E-02	2.0	NS	NS
NM_001252067	*Gga3*	BXD48a	1.37E-02	-1.4	NS	NS
NM_010288	*Gja1*	BXD48a	NS	NS	4.47E-03	1.5
NM_029102	*Glt8d2*	BXD48a	NS	NS	3.13E-02	2.0
ENSMUST00000073126	*Gm11555*	BXD48a	1.82E-02	-1.8	NS	NS
ENSMUST00000179595	*Gm21721*	BXD48a	2.96E-02	1.9	NS	NS
ENSMUST00000179217	*Gm2310*	BXD48a	4.50E-02	-2.0	NS	NS
NM_001035122	*Golm1*	BXD48a	2.96E-02	1.5	NS	NS
NM_008149	*Gpam*	BXD48a	1.31E-02	1.4	1.50E-02	1.4
NM_001110809	*Gpatch4*	BXD48a	3.33E-03	-1.7	1.06E-02	-1.7
NM_001145820	*Gpd2*	BXD48a	2.46E-02	-1.5	NS	NS
NM_027518	*Gpr137c*	BXD48a	6.89E-07	2.0	3.87E-05	1.8
NM_010338	*Gpr37*	BXD48a	4.25E-04	1.9	NS	NS
NM_145066	*Gpr85*	BXD48a	4.99E-03	1.6	NS	NS
NM_001177874	*Gps1*	BXD48a	NS	NS	3.93E-02	-1.3
NM_153419	*Grwd1*	BXD48a	NS	NS	5.05E-03	-2.0
NM_029938	*H2afv*	BXD48a	3.77E-02	1.6	NS	NS

NM_027382	*Hdac8*	BXD48a	NS	NS	3.55E-02	1.5
NM_144835	*Heatr1*	BXD48a	1.24E-02	-1.7	3.51E-02	-1.6
NM_175256	*Heg1*	BXD48a	6.03E-03	1.7	NS	NS
NM_025812	*Hmg20a*	BXD48a	2.74E-02	1.4	NS	NS
NM_008255	*Hmgcr*	BXD48a	7.80E-03	-1.5	6.54E-04	-1.7
NM_008256	*Hmgcs2*	BXD48a	1.32E-02	2.2	4.71E-05	3.0
NM_001177705	*Homez*	BXD48a	NS	NS	7.18E-03	-1.9
NM_010460	*Hoxb7*	BXD48a	NS	NS	1.63E-02	1.8
NM_053262	*Hsd17b11*	BXD48a	NS	NS	6.37E-03	1.6
NM_010476	*Hsd17b7*	BXD48a	5.68E-04	-1.7	3.99E-06	-1.9
NM_028306	*Hspa12b*	BXD48a	4.17E-02	1.7	NS	NS
NM_019564	*Htra1*	BXD48a	1.24E-02	1.7	1.39E-04	2.0
NM_010495	*Id1*	BXD48a	4.16E-03	2.0	NS	NS
NM_145360	*Idi1*	BXD48a	4.12E-07	-2.1	3.02E-06	-2.0
NM_013562	*Ifrd1*	BXD48a	NS	NS	7.83E-03	-1.7
NM_008360	*Il18*	BXD48a	NS	NS	4.22E-02	1.3
NM_010556	*Il3*	BXD48a	3.95E-02	1.8	NS	NS
NM_172439	*Inpp5j*	BXD48a	2.42E-02	1.7	NS	NS
NM_153526	*Insig1*	BXD48a	5.09E-05	-2.1	2.46E-05	-2.1
NM_010591	*Jun*	BXD48a	2.53E-11	3.0	5.95E-10	2.8
NM_001286944	*Jund*	BXD48a	4.12E-07	2.1	6.98E-03	1.6
NM_001289437	*Kansl2*	BXD48a	NS	NS	1.05E-02	-1.4
NM_001081134	*Kcng1*	BXD48a	NS	NS	1.56E-02	-1.8
NM_029911	*Kcnk10*	BXD48a	2.81E-02	1.5	NS	NS
NM_026135	*Kctd16*	BXD48a	NS	NS	4.69E-03	1.8
NM_078477	*Klf16*	BXD48a	1.39E-03	-1.8	1.59E-03	-1.7
NM_011803	*Klf6*	BXD48a	NS	NS	1.01E-02	-1.6
NM_001252658	*Ldlr*	BXD48a	2.34E-03	-2.1	3.80E-05	-2.5
NM_008494	*Lfng*	BXD48a	1.61E-03	1.6	NS	NS
NM_181074	*Lingo1*	BXD48a	3.77E-02	-1.3	NS	NS
NM_201529	*Lmo7*	BXD48a	2.67E-02	1.4	3.35E-02	1.4
NM_207206	*Lpcat4*	BXD48a	4.33E-02	-1.6	NS	NS
NM_008377	*Lrig1*	BXD48a	NS	NS	4.85E-02	-1.5
NM_001146048	*Lrrc1*	BXD48a	1.33E-02	1.9	NS	NS
NM_001143755	*Lrrc27*	BXD48a	2.99E-02	1.5	NS	NS
NM_133807	*Lrrc59*	BXD48a	3.41E-02	-1.4	3.35E-02	-1.4
NM_021500	*Maea*	BXD48a	NS	NS	3.29E-02	-1.4
NM_023788	*Mageh1*	BXD48a	NS	NS	2.47E-02	-1.4

NM_001145802	*Mak*	BXD48a	3.89E-02	-1.8	NS	NS
NM_011841	*Mapk7*	BXD48a	8.47E-03	-1.4	3.93E-02	-1.4
NM_028372	*Mblac2*	BXD48a	NS	NS	4.03E-02	-1.5
NM_001012335	*Mdk*	BXD48a	NS	NS	3.51E-02	1.8
NM_026896	*Med27*	BXD48a	NS	NS	7.83E-04	-1.6
NM_001001979	*Megf10*	BXD48a	NS	NS	4.01E-02	1.5
NM_008587	*Mertk*	BXD48a	NS	NS	4.52E-02	1.9
NM_023799	*Mgea5*	BXD48a	3.77E-02	1.3	3.56E-05	1.5
NM_001243584	*Mif4gd*	BXD48a	NS	NS	4.80E-02	1.6
NM_172457	*Mob3a*	BXD48a	NS	NS	4.07E-02	-1.5
NM_023773	*Mphosph8*	BXD48a	4.42E-02	-1.4	2.27E-02	-1.5
NM_001101482	*Mrap2*	BXD48a	5.31E-03	1.9	NS	NS
NM_025436	*Msmo1*	BXD48a	5.09E-05	-2.1	4.71E-05	-2.1
NM_013602	*Mt1*	BXD48a	1.24E-02	1.5	9.46E-04	1.7
NM_008630	*Mt2*	BXD48a	6.94E-05	2.4	3.99E-06	2.7
NM_008638	*Mthfd2*	BXD48a	NS	NS	2.63E-03	-1.9
NM_001005863	*Mtus1*	BXD48a	NS	NS	1.86E-03	1.7
NM_138656	*Mvd*	BXD48a	3.34E-02	-1.6	3.44E-04	-2.0
NM_001008542	*Mxi1*	BXD48a	2.49E-06	1.7	3.63E-07	1.8
NM_001040395	*Nadk2*	BXD48a	1.20E-02	1.4	NS	NS
NM_001114085	*Nde1*	BXD48a	NS	NS	2.17E-02	1.6
NM_022565	*Ndst4*	BXD48a	NS	NS	4.88E-02	1.7
NM_001111324	*Nedd9*	BXD48a	2.03E-03	1.7	NS	NS
NM_001048168	*Nfyc*	BXD48a	NS	NS	4.03E-02	-1.4
NM_145469	*Nipal2*	BXD48a	NS	NS	3.51E-02	1.5
NM_021303	*Noc2l*	BXD48a	NS	NS	1.86E-03	-1.6
NM_001008421	*Nol10*	BXD48a	NS	NS	3.71E-02	-1.6
NM_138747	*Nop2*	BXD48a	3.93E-03	-1.5	3.80E-03	-1.5
NM_024193	*Nop56*	BXD48a	1.30E-02	-1.6	NS	NS
NM_018868	*Nop58*	BXD48a	NS	NS	2.72E-02	-1.4
NM_010342	*Npbwr1*	BXD48a	3.86E-02	1.6	NS	NS
NM_008723	*Npm3*	BXD48a	NS	NS	3.72E-02	-1.6
NM_175678	*Npsr1*	BXD48a	3.77E-02	-2.4	NS	NS
NM_021552	*Nsa2*	BXD48a	4.42E-02	1.4	NS	NS
NM_010941	*Nsdhl*	BXD48a	1.20E-02	-1.4	NS	NS
NM_001164604	*Nsmce2*	BXD48a	4.84E-02	1.4	NS	NS
NM_008751	*Nxph1*	BXD48a	NS	NS	1.06E-02	-1.5
NM_013614	*Odc1*	BXD48a	2.08E-03	1.8	NS	NS

NM_146491	*Olfr1410*	BXD48a	1.61E-03	-2.1	NS	NS
NM_010970	*Olfr23*	BXD48a	3.65E-02	-1.9	NS	NS
NM_001011858	*Olfr504*	BXD48a	4.84E-02	-2.0	NS	NS
NM_147067	*Olfr829*	BXD48a	7.80E-03	-2.9	NS	NS
NM_146557	*Olfr869*	BXD48a	NS	NS	2.82E-02	-2.5
NM_001134791	*Osbpl9*	BXD48a	1.03E-03	1.4	1.63E-02	1.3
NM_001101479	*Pabpc4l*	BXD48a	NS	NS	3.37E-02	1.8
NM_146169	*Paip2b*	BXD48a	NS	NS	4.15E-02	1.5
NM_198410	*Paqr6*	BXD48a	NS	NS	4.69E-03	1.8
NM_183028	*Pcmtd1*	BXD48a	3.77E-02	1.3	NS	NS
NM_153594	*Pcmtd2*	BXD48a	1.15E-04	2.0	NS	NS
NM_008803	*Pde8a*	BXD48a	2.07E-02	1.9	2.40E-02	1.8
NM_008808	*Pdgfa*	BXD48a	NS	NS	1.63E-02	-1.5
NM_001159367	*Per1*	BXD48a	4.06E-02	1.5	2.40E-02	1.6
NM_001109690	*Phf21a*	BXD48a	3.94E-02	1.6	NS	NS
NM_030064	*Phf23*	BXD48a	NS	NS	1.32E-02	-1.6
NM_178149	*Pik3ip1*	BXD48a	1.15E-04	2.2	9.40E-05	2.2
NM_001024955	*Pik3r1*	BXD48a	1.52E-06	1.6	7.15E-07	1.7
NM_181585	*Pik3r3*	BXD48a	NS	NS	2.91E-02	1.4
NM_023371	*Pin1*	BXD48a	5.15E-03	-1.5	NS	NS
NM_152813	*Plcd3*	BXD48a	1.89E-03	1.9	4.50E-04	2.0
NM_001033253	*Plekhg1*	BXD48a	2.55E-06	2.2	6.54E-04	1.8
NM_013807	*Plk3*	BXD48a	NS	NS	1.39E-03	-1.8
NM_022995	*Pmepa1*	BXD48a	2.39E-02	1.4	NS	NS
NM_001033225	*Pnrc1*	BXD48a	NS	NS	4.79E-02	1.3
NM_177782	*Prex1*	BXD48a	1.24E-02	-1.5	NS	NS
NM_011192	*Psme3*	BXD48a	2.42E-02	-1.3	NS	NS
NM_212446	*Psmf1*	BXD48a	1.20E-02	1.3	NS	NS
NM_022415	*Ptges*	BXD48a	NS	NS	2.14E-02	1.7
NM_001163565	*Ptpn5*	BXD48a	NS	NS	2.49E-02	-1.3
NM_025831	*Pxdc1*	BXD48a	2.92E-03	1.7	NS	NS
NM_029494	*Rab30*	BXD48a	7.32E-07	2.0	3.55E-05	1.8
NM_001038621	*Rabgap1l*	BXD48a	1.22E-03	1.4	3.32E-02	1.3
NM_001099624	*Rapgef2*	BXD48a	3.70E-06	1.6	9.55E-05	1.5
NM_001252494	*Rapgef6*	BXD48a	5.09E-05	1.9	1.52E-05	2.0
NM_001081549	*Rcan1*	BXD48a	4.53E-02	1.4	NS	NS
NM_009062	*Rgs4*	BXD48a	4.47E-02	1.5	3.38E-03	1.7
NM_153514	*Rhobtb2*	BXD48a	NS	NS	2.58E-03	-1.6

NM_172612	*Rnd1*	BXD48a	2.81E-02	-1.6	NS	NS
NM_001166553	*Rnf145*	BXD48a	1.43E-03	-1.5	6.78E-07	-1.8
NM_146244	*Rps6kl1*	BXD48a	2.14E-03	1.8	NS	NS
NM_145620	*Rrp9*	BXD48a	2.57E-03	-1.9	NS	NS
NM_013650	*S100a8*	BXD48a	NS	NS	7.85E-03	2.5
NM_001244916	*Sall2*	BXD48a	NS	NS	2.52E-04	-1.8
NM_172604	*Scara3*	BXD48a	3.10E-02	1.5	5.85E-03	1.6
NM_009129	*Scg2*	BXD48a	1.24E-02	1.3	8.84E-03	1.4
NM_011521	*Sdc4*	BXD48a	1.57E-04	2.5	2.25E-04	2.5
NM_001013370	*Sesn1*	BXD48a	3.83E-02	1.5	6.53E-04	1.7
NM_030261	*Sesn3*	BXD48a	NS	NS	4.30E-03	1.5
NM_016687	*Sfrp4*	BXD48a	NS	NS	9.46E-04	2.5
NM_029339	*Sgf29*	BXD48a	NS	NS	4.12E-02	-1.4
NM_001162965	*Sgsm1*	BXD48a	1.82E-02	-1.5	NS	NS
NM_009167	*Shc3*	BXD48a	1.95E-02	-1.3	1.10E-02	-1.4
NM_001286538	*Sigmar1*	BXD48a	2.39E-02	-1.4	NS	NS
NM_027921	*Slc16a14*	BXD48a	3.08E-03	1.6	NS	NS
NM_001029842	*Slc16a6*	BXD48a	NS	NS	1.12E-02	2.1
NM_025807	*Slc16a9*	BXD48a	NS	NS	2.27E-02	2.9
NM_001159593	*Slc20a1*	BXD48a	6.94E-05	-1.6	NS	NS
NM_198650	*Slc22a20*	BXD48a	4.55E-02	-1.6	NS	NS
ENSMUST00000114764	*Slc26a8*	BXD48a	NS	NS	4.25E-02	1.8
NM_007854	*Slc29a2*	BXD48a	2.46E-02	-1.7	NS	NS
NM_011400	*Slc2a1*	BXD48a	7.50E-05	2.1	5.10E-03	1.8
NM_175121	*Slc38a2*	BXD48a	NS	NS	1.88E-02	1.4
NM_001012305	*Slc39a12*	BXD48a	NS	NS	7.80E-03	1.7
NM_001199283	*Slc43a2*	BXD48a	2.81E-02	1.5	NS	NS
NM_001081263	*Slc44a5*	BXD48a	2.55E-06	1.9	1.43E-04	1.7
NM_029643	*Slc52a2*	BXD48a	NS	NS	2.98E-03	-1.7
NM_001038643	*Slco3a1*	BXD48a	NS	NS	6.17E-03	-1.5
NM_008539	*Smad1*	BXD48a	NS	NS	4.25E-02	1.3
NM_001177833	*Smox*	BXD48a	NS	NS	4.15E-02	1.5
NM_146126	*Sord*	BXD48a	1.62E-02	1.7	NS	NS
NM_001025428	*Spag9*	BXD48a	3.41E-02	1.3	3.35E-02	1.3
NM_182927	*Spred3*	BXD48a	2.75E-02	-1.8	1.08E-03	-2.2
NM_011898	*Spry4*	BXD48a	NS	NS	6.42E-03	-1.8
NM_009270	*Sqle*	BXD48a	1.61E-03	-1.6	1.08E-02	-1.5

NM_001081037	*Srgap1*	BXD48a	3.48E-02	-1.5	NS	NS
NM_009272	*Srm*	BXD48a	4.17E-02	-1.4	4.80E-02	-1.4
NM_016795	*Srpk1*	BXD48a	1.20E-02	-1.5	2.59E-02	-1.5
NM_011358	*Srsf2*	BXD48a	2.46E-02	-1.4	2.52E-04	-1.6
NM_001195485	*Srsf7*	BXD48a	NS	NS	3.29E-04	-1.9
NM_009218	*Sstr3*	BXD48a	NS	NS	8.03E-03	-1.9
NM_011374	*St8sia1*	BXD48a	2.64E-02	1.4	7.21E-03	1.5
NM_133774	*Stard4*	BXD48a	3.00E-02	-1.6	1.44E-02	-1.7
NM_153563	*Strip1*	BXD48a	4.74E-03	-1.4	1.33E-03	-1.4
NM_019995	*Supt20*	BXD48a	NS	NS	4.69E-03	-1.7
NM_013678	*Surf2*	BXD48a	NS	NS	4.69E-03	-1.4
NM_001040085	*Sytl2*	BXD48a	NS	NS	1.33E-02	1.8
NM_001289603	*Tfcp2*	BXD48a	NS	NS	4.69E-03	-1.6
NM_009368	*Tgfb3*	BXD48a	NS	NS	2.53E-02	2.1
NM_011580	*Thbs1*	BXD48a	4.17E-02	2.0	NS	NS
NM_174989	*Ticam1*	BXD48a	1.24E-02	2.0	NS	NS
NM_001081145	*Tigd2*	BXD48a	4.92E-02	1.4	NS	NS
NM_026708	*Tlcd1*	BXD48a	NS	NS	1.63E-02	-1.5
NM_133211	*Tlr7*	BXD48a	1.30E-02	2.0	4.64E-02	1.9
NM_031178	*Tlr9*	BXD48a	1.29E-02	2.1	NS	NS
NM_177344	*Tmem203*	BXD48a	2.74E-02	-1.7	NS	NS
NM_001048267	*Tnpo1*	BXD48a	1.52E-03	1.5	9.13E-03	1.4
NM_001109748	*Tomm40*	BXD48a	NS	NS	3.35E-02	-1.4
NM_001164792	*Tpbg*	BXD48a	1.20E-02	2.0	NS	NS
NM_001099792	*Trmt61a*	BXD48a	NS	NS	2.63E-03	-1.8
NM_032000	*Trps1*	BXD48a	NS	NS	3.51E-02	1.5
NM_001177751	*Tsc22d1*	BXD48a	4.19E-02	1.2	NS	NS
NM_197996	*Tspan15*	BXD48a	NS	NS	2.65E-02	1.6
NM_146116	*Tubb4b*	BXD48a	NS	NS	2.80E-02	-1.4
NM_001009935	*Txnip*	BXD48a	2.55E-06	3.8	1.99E-07	4.6
NM_001169576	*Ube2h*	BXD48a	1.29E-02	1.4	6.17E-03	1.5
NM_001145162	*Ube2ql1*	BXD48a	NS	NS	3.51E-02	-1.3
NM_030724	*Uck2*	BXD48a	NS	NS	1.63E-02	-1.4
ENSMUST00000053686	*Uck2*	BXD48a	NS	NS	2.99E-02	-1.7
NM_009462	*Usp10*	BXD48a	NS	NS	7.78E-03	-1.4
NM_183225	*Usp24*	BXD48a	4.84E-02	-1.4	NS	NS
NM_001081249	*Vcan*	BXD48a	NS	NS	2.05E-02	-1.5

NM_001166737	*Vmn1r103*	BXD48a	2.77E-02	-1.8	NS	NS
NM_001105179	*Vmn2r51*	BXD48a	4.17E-02	-2.1	NS	NS
NM_022997	*Vps35*	BXD48a	NS	NS	2.67E-02	-1.3
NM_145967	*Vstm2a*	BXD48a	2.12E-03	-1.4	9.46E-04	-1.4
NM_054068	*Vsx1*	BXD48a	NS	NS	3.65E-02	-1.6
NM_172372	*Wdr45*	BXD48a	2.74E-02	1.7	NS	NS
NM_134139	*Wdr74*	BXD48a	3.00E-02	-1.4	NS	NS
NM_145940	*Wipi1*	BXD48a	NS	NS	4.80E-02	1.4
NM_001005342	*Ypel4*	BXD48a	3.41E-02	1.9	4.25E-02	1.9
NM_027166	*Ypel5*	BXD48a	1.28E-04	1.6	1.49E-04	1.6
NM_028106	*Zbed3*	BXD48a	2.65E-03	1.6	1.44E-02	1.5
NM_001115130	*Zbtb44*	BXD48a	NS	NS	4.85E-02	1.4
NM_001082485	*Zfp266*	BXD48a	3.00E-02	1.4	NS	NS
NM_001110309	*Zfp426*	BXD48a	2.01E-02	1.5	NS	NS
NM_145622	*Zfp65*	BXD48a	NS	NS	3.58E-02	1.5
NM_001113486	*-44448*	BXD100	NS	NS	6.87E-03	-1.3
NM_001033262	*-44264*	BXD100	NS	NS	2.48E-03	-1.8
NM_001166372	*-44256*	BXD100	4.02E-02	1.4	3.02E-02	1.4
NM_021446	*0610007P14Rik*	BXD100	3.82E-03	-1.4	3.27E-03	-1.4
NM_001145895	*1700019G17Rik*	BXD100	3.40E-02	1.6	NS	NS
NR_045177	*2900055J20Rik*	BXD100	4.69E-03	1.7	7.87E-07	2.2
NM_001081963	*9430020K01Rik*	BXD100	NS	NS	1.43E-02	1.7
NM_001167925	*A730017C20Rik*	BXD100	2.62E-02	1.2	7.66E-03	1.2
NR_015488	*A930003A15Rik*	BXD100	NS	NS	3.62E-03	2.1
NR_033609	*A930017M01Rik*	BXD100	2.61E-03	1.8	NS	NS
NM_030210	*Aacs*	BXD100	7.38E-03	-1.5	9.63E-03	-1.5
NM_001166556	*Abca6*	BXD100	4.74E-02	1.9	NS	NS
NM_013851	*Abca8b*	BXD100	2.36E-04	2.3	2.01E-02	1.8
NM_011075	*Abcb1b*	BXD100	NS	NS	1.79E-04	2.3
NM_001190443	*Abcf2*	BXD100	NS	NS	4.08E-02	-1.3
NM_025826	*Acadsb*	BXD100	1.54E-02	1.6	NS	NS
NM_009338	*Acat2*	BXD100	NS	NS	1.58E-02	-1.4
NM_153151	*Acat3*	BXD100	NS	NS	2.66E-02	-1.4
NM_001199296	*Acly*	BXD100	NS	NS	4.11E-03	-1.4
NM_001205385	*Actr3*	BXD100	1.20E-02	-1.2	NS	NS
NM_001270996	*Adam9*	BXD100	1.67E-02	1.4	NS	NS
NM_001038587	*Adar*	BXD100	NS	NS	2.66E-02	1.4
NM_001024837	*Adarb1*	BXD100	4.80E-02	-1.4	NS	NS

NM_001163029	*Adgre5*	BXD100	2.21E-02	1.7	NS	NS
NM_133222	*Adgrl4*	BXD100	2.44E-02	1.4	5.32E-03	1.5
NM_001080798	*Aff1*	BXD100	6.91E-03	1.8	2.01E-02	1.7
NM_178162	*Agfg2*	BXD100	2.07E-04	-2.1	1.08E-03	-2.0
NM_026644	*Agpat4*	BXD100	9.46E-04	1.5	NS	NS
NM_007428	*Agt*	BXD100	1.34E-02	3.1	NS	NS
NM_009642	*Agtrap*	BXD100	1.34E-02	1.7	NS	NS
NM_001172146	*Aimp2*	BXD100	1.59E-02	-1.7	3.09E-02	-1.6
NM_198111	*Akap6*	BXD100	NS	NS	4.95E-02	1.2
NM_017476	*Akap8l*	BXD100	7.23E-03	1.4	7.40E-03	1.4
NM_009658	*Akr1b3*	BXD100	3.29E-02	-1.4	NS	NS
NM_134066	*Akr1c18*	BXD100	9.90E-03	-1.9	NS	NS
ENSMUST0000 0080898	*Amd2*	BXD100	NS	NS	4.84E-02	-1.5
NM_019764	*Amotl2*	BXD100	NS	NS	4.68E-02	1.6
NM_007426	*Angpt2*	BXD100	NS	NS	3.09E-02	1.8
NM_020581	*Angptl4*	BXD100	7.08E-04	2.2	1.08E-03	2.2
NM_001024851	*Ankrd34a*	BXD100	2.82E-02	-1.8	1.63E-02	-1.8
NM_001081433	*Ankrd44*	BXD100	4.99E-02	1.5	NS	NS
NM_028390	*Anln*	BXD100	2.53E-02	2.0	NS	NS
NM_001277188	*Ano4*	BXD100	NS	NS	2.70E-02	1.5
NM_001253813	*Ano6*	BXD100	4.10E-03	1.5	1.82E-02	1.4
NM_026674	*Aph1c*	BXD100	1.17E-02	1.5	NS	NS
NM_001109914	*Apold1*	BXD100	1.79E-02	1.8	NS	NS
NM_007481	*Arf6*	BXD100	NS	NS	1.99E-02	-1.3
NM_009705	*Arg2*	BXD100	4.66E-02	1.7	NS	NS
NM_001172205	*Arid5a*	BXD100	8.07E-06	2.8	7.23E-05	2.6
NM_023598	*Arid5b*	BXD100	1.15E-07	2.1	3.01E-04	1.7
NM_001042591	*Arrdc3*	BXD100	1.34E-10	3.3	4.09E-06	2.4
NM_001042592	*Arrdc4*	BXD100	7.08E-04	1.9	NS	NS
NM_007492	*Arx*	BXD100	NS	NS	3.33E-03	-1.9
NM_019652	*Asna1*	BXD100	NS	NS	3.50E-02	-1.3
NM_179203	*Atad3a*	BXD100	NS	NS	5.20E-03	-1.5
NM_001287180	*Atf4*	BXD100	3.51E-02	-1.4	NS	NS
NM_019426	*Atf7ip*	BXD100	NS	NS	6.60E-03	1.5
NM_026217	*Atg12*	BXD100	2.56E-04	1.4	1.22E-02	1.3
NM_001199304	*Atxn1*	BXD100	NS	NS	2.04E-02	1.4
NM_030235	*Avl9*	BXD100	3.41E-02	1.3	NS	NS

NM_016847	*Avpr1a*	BXD100	3.96E-05	-2.3	1.60E-06	-2.6
NM_020025	*B3galt2*	BXD100	2.21E-02	1.5	3.47E-02	1.5
NM_001122993	*B3galt5*	BXD100	1.30E-02	1.6	4.07E-02	1.5
NM_001159407	*B3gnt5*	BXD100	3.47E-04	1.9	3.04E-03	1.7
NM_019835	*B4galt5*	BXD100	1.01E-02	-1.5	1.67E-03	-1.6
NM_001001182	*Baz2b*	BXD100	3.32E-02	1.4	1.44E-02	1.5
NM_175325	*Bbs4*	BXD100	9.62E-04	1.4	6.19E-04	1.4
NM_178415	*Bbs9*	BXD100	3.95E-02	1.5	NS	NS
NM_027444	*Bbx*	BXD100	4.02E-02	1.6	NS	NS
NM_001284410	*Bcl2l11*	BXD100	6.04E-07	2.9	2.11E-07	3.1
NM_001122683	*Bdh1*	BXD100	3.55E-03	-1.4	3.61E-04	-1.5
NM_173404	*Bmp3*	BXD100	2.50E-02	-1.7	6.60E-03	-1.8
NM_001277216	*Bmpr1b*	BXD100	3.88E-02	1.6	NS	NS
NM_001136064	*Bscl2*	BXD100	2.49E-02	1.4	NS	NS
NM_133889	*Bsdc1*	BXD100	2.27E-05	1.5	3.33E-03	1.4
NM_007570	*Btg2*	BXD100	6.36E-03	1.7	8.59E-03	1.7
NM_009770	*Btg3*	BXD100	2.32E-06	2.4	6.73E-03	1.8
NM_016859	*Bysl*	BXD100	6.02E-03	-1.7	1.04E-02	-1.7
NM_007573	*C1qbp*	BXD100	3.07E-02	-1.3	NS	NS
NM_001135172	*C1qtnf7*	BXD100	1.28E-02	1.8	NS	NS
NM_001146287	*Cables1*	BXD100	NS	NS	3.08E-03	2.0
NM_001159533	*Cacna1c*	BXD100	NS	NS	3.44E-02	1.4
NM_026192	*Calcoco1*	BXD100	NS	NS	3.60E-02	1.4
NM_144817	*Camk1g*	BXD100	NS	NS	8.57E-03	-1.5
NM_025821	*Carhsp1*	BXD100	7.23E-03	1.8	NS	NS
NM_007625	*Cbx4*	BXD100	NS	NS	3.09E-02	-1.4
NM_001081429	*Ccdc15*	BXD100	NS	NS	4.69E-02	1.6
NM_177716	*Ccdc184*	BXD100	3.55E-03	-1.6	2.22E-02	-1.5
NM_144820	*Ccdc28a*	BXD100	NS	NS	3.13E-03	2.0
NM_011337	*Ccl3*	BXD100	1.52E-03	2.7	NS	NS
NM_007631	*Ccnd1*	BXD100	2.47E-02	-1.5	NS	NS
NM_009831	*Ccng1*	BXD100	NS	NS	3.49E-02	1.3
NM_007635	*Ccng2*	BXD100	NS	NS	3.32E-02	1.4
NM_001114328	*Ccpg1*	BXD100	NS	NS	2.95E-02	1.4
NM_009837	*Cct4*	BXD100	NS	NS	3.02E-02	-1.3
NM_001170395	*Cd163*	BXD100	2.45E-02	2.3	NS	NS
NM_007646	*Cd38*	BXD100	NS	NS	2.37E-02	2.0
NM_009866	*Cdh11*	BXD100	1.84E-02	-1.5	NS	NS

NM_009875	*Cdkn1b*	BXD100	2.29E-02	1.4	NS	NS
NM_172447	*Cfap69*	BXD100	1.01E-02	2.1	NS	NS
NM_001081345	*Chd2*	BXD100	3.55E-03	1.5	1.99E-02	1.4
NM_001163640	*Chn2*	BXD100	3.75E-02	1.4	NS	NS
NM_025854	*Cir1*	BXD100	2.23E-02	1.4	NS	NS
NM_001177770	*Clcc1*	BXD100	NS	NS	2.01E-02	1.7
NM_001243762	*Clcn5*	BXD100	1.01E-02	1.6	1.67E-03	1.8
NM_153384	*Clrn1*	BXD100	3.32E-03	1.9	8.21E-03	1.8
NM_024217	*Cmtm3*	BXD100	2.29E-02	1.4	NS	NS
NM_009932	*Col4a2*	BXD100	NS	NS	2.04E-02	1.6
NM_009898	*Coro1a*	BXD100	9.74E-03	-1.7	1.16E-02	-1.7
NM_001033310	*Cox18*	BXD100	2.87E-03	-1.5	1.89E-03	-1.5
NM_001252525	*Cpeb1*	BXD100	9.02E-03	1.9	NS	NS
NM_013499	*Cr1l*	BXD100	2.54E-02	1.5	NS	NS
NM_172728	*Creb5*	BXD100	2.70E-06	2.7	4.47E-03	2.0
NM_001110850	*Crem*	BXD100	1.83E-04	1.9	4.09E-06	2.2
NM_007762	*Crhr1*	BXD100	1.81E-04	-2.1	9.10E-06	-2.4
NM_030209	*Crispld2*	BXD100	NS	NS	1.95E-02	2.0
NM_153409	*Csrnp3*	BXD100	4.64E-02	1.3	NS	NS
NM_007791	*Csrp1*	BXD100	1.49E-02	1.6	NS	NS
NM_001145799	*Ctla2a*	BXD100	NS	NS	1.87E-02	1.8
NM_031251	*Ctns*	BXD100	1.25E-02	1.5	1.94E-02	1.4
NM_016748	*Ctps*	BXD100	1.96E-02	-1.5	1.04E-02	-1.6
NM_007801	*Ctsh*	BXD100	1.59E-02	2.1	NS	NS
NM_009984	*Ctsl*	BXD100	1.25E-02	1.5	NS	NS
NM_153775	*Ctu2*	BXD100	4.13E-02	-1.6	NS	NS
NM_025567	*Cyc1*	BXD100	1.05E-02	-1.5	5.67E-03	-1.6
NM_009994	*Cyp1b1*	BXD100	1.13E-04	2.9	1.14E-05	3.3
NM_001177713	*Cyp26b1*	BXD100	7.67E-03	-2.1	2.70E-02	-1.9
NM_028979	*Cyp2j9*	BXD100	5.22E-05	2.2	2.66E-02	1.7
NM_130882	*Cyp4f13*	BXD100	3.46E-02	1.6	NS	NS
NM_020010	*Cyp51*	BXD100	2.01E-03	-1.4	5.77E-05	-1.6
NM_010012	*Cyp8b1*	BXD100	NS	NS	2.66E-02	1.5
NM_010516	*Cyr61*	BXD100	3.25E-03	1.8	NS	NS
NM_001081365	*Cystm1*	BXD100	1.63E-02	1.5	NS	NS
NM_172911	*D8Ertd82e*	BXD100	6.77E-03	1.7	NS	NS
NM_001146308	*Dbnl*	BXD100	NS	NS	5.54E-03	-1.6
NM_001165980	*Dcaf17*	BXD100	1.17E-02	1.4	NS	NS

NM_153555	*Dcaf8*	BXD100	4.63E-02	1.2	NS	NS
NM_001080995	*Ddias*	BXD100	4.22E-02	1.7	NS	NS
NM_007837	*Ddit3*	BXD100	2.33E-02	1.7	NS	NS
NM_029083	*Ddit4*	BXD100	1.34E-02	1.7	NS	NS
NM_019553	*Ddx21*	BXD100	NS	NS	2.62E-04	-1.8
NM_001252457	*Ddx39b*	BXD100	1.11E-02	-1.3	2.74E-03	-1.4
NM_001282055	*Ddx46*	BXD100	3.29E-02	-1.4	NS	NS
NM_001037937	*Deptor*	BXD100	NS	NS	1.81E-02	1.8
NM_053272	*Dhcr24*	BXD100	5.85E-11	-1.9	7.27E-15	-2.2
NM_025522	*Dhrs7*	BXD100	6.11E-03	1.7	NS	NS
NM_007842	*Dhx9*	BXD100	3.57E-02	-1.3	NS	NS
NM_028315	*Dis3*	BXD100	NS	NS	3.23E-02	-1.5
NM_001042487	*Dlgap4*	BXD100	2.21E-02	-1.4	NS	NS
NM_001286013	*Dlk2*	BXD100	NS	NS	3.60E-02	-1.7
NR_002854	*Dlx1as*	BXD100	NS	NS	2.35E-02	-1.9
NM_009584	*Dnajc2*	BXD100	NS	NS	2.92E-02	-1.4
NM_001163536	*Dnajc5b*	BXD100	2.79E-02	-2.0	NS	NS
NM_001285807	*Dtna*	BXD100	1.56E-03	1.4	2.30E-04	1.5
NM_172442	*Dtx4*	BXD100	5.55E-03	1.4	NS	NS
NM_013642	*Dusp1*	BXD100	1.34E-10	2.9	3.33E-10	2.8
NM_019819	*Dusp14*	BXD100	6.53E-03	1.8	NS	NS
NM_001048054	*Dusp16*	BXD100	1.63E-07	2.7	2.42E-03	1.9
NM_026268	*Dusp6*	BXD100	1.50E-03	-1.7	6.22E-03	-1.6
NM_008748	*Dusp8*	BXD100	3.96E-07	1.6	6.81E-04	1.4
NM_001037957	*Dyrk1b*	BXD100	NS	NS	1.89E-04	1.6
NM_001289920	*E2f3*	BXD100	NS	NS	2.37E-02	-1.6
NM_026932	*Ebna1bp2*	BXD100	1.97E-02	-1.4	NS	NS
NM_001252653	*Ecm1*	BXD100	NS	NS	3.66E-02	1.6
NM_010104	*Edn1*	BXD100	3.32E-03	2.7	3.53E-03	2.7
NM_025380	*Eef1e1*	BXD100	NS	NS	3.12E-03	-1.5
NM_010108	*Efna3*	BXD100	NS	NS	6.22E-03	-1.5
NM_001289925	*Egr3*	BXD100	3.02E-09	-3.2	1.67E-04	-2.2
NM_018781	*Egr3*	BXD100	2.88E-02	-1.7	NS	NS
NM_153068	*Ehd2*	BXD100	NS	NS	2.21E-02	2.0
NM_010121	*Eif2ak3*	BXD100	3.39E-02	1.5	2.01E-02	1.5
NM_018749	*Eif3d*	BXD100	NS	NS	3.66E-02	-1.4
NM_016876	*Eif3g*	BXD100	NS	NS	3.71E-03	-1.5
NM_138669	*Eif4a3*	BXD100	3.51E-02	-1.4	NS	NS

NM_007917	*Eif4e*	BXD100	2.30E-02	-1.3	NS	NS
NM_001177883	*Elavl2*	BXD100	NS	NS	3.12E-03	-1.4
NM_001253692	*Elmod3*	BXD100	9.04E-03	1.5	NS	NS
NM_130450	*Elovl6*	BXD100	1.14E-02	-1.4	NS	NS
NM_175101	*Emc3*	BXD100	1.54E-02	1.3	NS	NS
NM_001146348	*Eng*	BXD100	3.23E-02	1.7	NS	NS
NM_134005	*Enpp3*	BXD100	3.41E-02	1.7	NS	NS
NM_001168620	*Enpp5*	BXD100	3.66E-04	1.5	NS	NS
NM_175313	*Eogt*	BXD100	NS	NS	3.57E-02	1.5
NM_010145	*Ephx1*	BXD100	4.95E-02	2.0	NS	NS
NM_153592	*Erlin2*	BXD100	4.67E-02	1.4	NS	NS
NM_023612	*Esm1*	BXD100	3.95E-02	1.8	NS	NS
NM_029250	*Etnk1*	BXD100	2.96E-02	1.4	NS	NS
NM_008815	*Etv4*	BXD100	2.79E-03	-1.7	NS	NS
NM_023794	*Etv5*	BXD100	2.07E-02	-1.6	2.66E-04	-1.9
NM_001252192	*Eya1*	BXD100	NS	NS	3.95E-02	1.4
NM_007970	*Ezh1*	BXD100	8.85E-03	1.4	1.05E-02	1.4
NM_010171	*F3*	BXD100	3.27E-02	1.5	NS	NS
NM_007983	*Faf1*	BXD100	5.55E-03	-1.4	NS	NS
NM_183187	*Fam107a*	BXD100	6.60E-07	3.6	1.11E-05	3.2
NM_028672	*Fam161a*	BXD100	1.31E-02	2.3	NS	NS
NM_182808	*Fam19a1*	BXD100	NS	NS	2.81E-02	1.5
NM_001113283	*Fam214a*	BXD100	NS	NS	4.02E-02	1.6
NM_001172216	*Fam221a*	BXD100	4.09E-02	1.5	NS	NS
NM_178908	*Fam26e*	BXD100	6.61E-06	2.2	1.06E-03	1.9
NM_175104	*Fam53c*	BXD100	4.47E-04	2.0	NS	NS
NM_178797	*Far2*	BXD100	3.95E-02	-1.4	1.06E-03	-1.5
NM_007988	*Fasn*	BXD100	NS	NS	3.53E-02	-1.4
NM_001081034	*Fbxo11*	BXD100	1.80E-02	-1.2	NS	NS
NM_172591	*Fcho2*	BXD100	2.68E-02	1.4	NS	NS
NM_010191	*Fdft1*	BXD100	2.48E-08	-1.7	6.89E-10	-1.8
NM_001253751	*Fdps*	BXD100	4.88E-02	-1.5	2.20E-03	-1.7
NM_028263	*Fgfbp3*	BXD100	NS	NS	2.17E-03	-1.6
NM_001163215	*Fgfr3*	BXD100	5.48E-05	2.0	1.15E-03	1.8
NM_001164259	*Fgfrl1*	BXD100	1.42E-02	-1.6	1.15E-02	-1.6
NM_001081243	*Filip1*	BXD100	NS	NS	3.71E-02	1.7
NM_010219	*Fkbp4*	BXD100	3.62E-02	-1.3	5.32E-03	-1.4
NM_134080	*Flnb*	BXD100	NS	NS	7.01E-03	1.7

NM_010228	*Flt1*	BXD100	NS	NS	2.57E-02	1.4
NM_001159706	*Folh1*	BXD100	1.08E-03	2.3	3.89E-03	2.1
NM_028428	*Fut11*	BXD100	3.49E-02	1.4	NS	NS
NM_011817	*Gadd45g*	BXD100	1.11E-02	2.0	NS	NS
NM_001081421	*Galnt16*	BXD100	4.07E-02	1.4	NS	NS
NM_173739	*Galnt18*	BXD100	2.21E-02	-1.7	NS	NS
NM_001190406	*Gas2l1*	BXD100	2.21E-02	1.5	NS	NS
NM_028022	*Gatsl3*	BXD100	NS	NS	1.47E-02	2.1
NM_018771	*Gipc1*	BXD100	NS	NS	4.19E-02	-1.5
NM_010288	*Gja1*	BXD100	4.19E-02	1.4	NS	NS
NM_001271628	*Gja5*	BXD100	NS	NS	8.85E-05	2.7
NM_027450	*Glipr2*	BXD100	4.24E-02	1.6	NS	NS
NM_029102	*Glt8d2*	BXD100	4.76E-03	2.2	NS	NS
NM_001081418	*Gltscr1*	BXD100	2.96E-02	-1.7	NS	NS
NM_001100452	*Gltscr1l*	BXD100	1.31E-02	1.4	NS	NS
ENSMUST00000123179	*Gm12666*	BXD100	3.70E-02	-1.4	NS	NS
uc008vqj.1	*Gm13089*	BXD100	4.64E-02	-1.8	NS	NS
ENSMUST00000115553	*Gm14862*	BXD100	3.79E-02	1.6	NS	NS
NM_001195529	*Gm4980*	BXD100	3.32E-02	1.6	NS	NS
NM_010313	*Gnb5*	BXD100	2.38E-02	-1.4	NS	NS
NM_001190414	*Gne*	BXD100	NS	NS	7.06E-03	-1.5
NM_001177669	*Golph3l*	BXD100	1.42E-02	1.5	NS	NS
NM_008149	*Gpam*	BXD100	3.54E-03	1.4	NS	NS
NM_001110809	*Gpatch4*	BXD100	1.50E-03	-1.8	7.37E-03	-1.7
NM_145965	*Gphn*	BXD100	2.21E-02	-1.3	NS	NS
NM_027518	*Gpr137c*	BXD100	4.39E-09	2.2	9.36E-07	2.0
NM_001167694	*Gpr19*	BXD100	9.04E-03	1.5	NS	NS
NM_173410	*Gpr26*	BXD100	3.29E-02	-1.5	NS	NS
NM_010338	*Gpr37*	BXD100	7.08E-03	1.7	4.60E-03	1.7
NM_145066	*Gpr85*	BXD100	6.34E-03	1.5	1.57E-02	1.5
NM_001195774	*Gprc5b*	BXD100	1.03E-02	1.5	NS	NS
NM_012014	*Gprin1*	BXD100	3.15E-02	-1.4	NS	NS
NM_001177874	*Gps1*	BXD100	NS	NS	3.61E-02	-1.3
NM_173866	*Gpt2*	BXD100	NS	NS	4.02E-02	1.5
NM_001159507	*Grip2*	BXD100	NS	NS	4.52E-02	-1.6
NM_001114333	*Grm1*	BXD100	1.96E-02	1.5	NS	NS
NM_153419	*Grwd1*	BXD100	NS	NS	5.16E-03	-1.9

NM_010358	*Gstm1*	BXD100	1.09E-02	1.4	NS	NS
NM_001160411	*Gstm4*	BXD100	2.21E-02	1.5	NS	NS
NM_001033322	*Gucy1a2*	BXD100	3.95E-02	1.5	NS	NS
NM_010389	*H2-Ob*	BXD100	2.70E-02	-1.6	NS	NS
NM_008214	*Hars*	BXD100	NS	NS	4.58E-02	-1.3
NM_008216	*Has2*	BXD100	3.25E-03	-2.5	NS	NS
NM_001081192	*Hcn4*	BXD100	NS	NS	2.01E-02	-1.6
NM_198962	*Hcrtr2*	BXD100	8.09E-04	-2.0	1.95E-02	-1.7
NM_144835	*Heatr1*	BXD100	1.14E-02	-1.6	3.04E-03	-1.7
NM_175256	*Heg1*	BXD100	3.95E-02	1.5	3.50E-03	1.7
NM_001162950	*Hif3a*	BXD100	4.95E-02	1.9	NS	NS
NM_010432	*Hipk1*	BXD100	4.64E-02	1.3	NS	NS
NM_025812	*Hmg20a*	BXD100	4.09E-03	1.5	4.98E-03	1.5
NM_008255	*Hmgcr*	BXD100	3.45E-06	-1.9	1.24E-04	-1.7
NM_008256	*Hmgcs2*	BXD100	3.47E-04	2.6	7.32E-03	2.2
NM_007545	*Hrk*	BXD100	NS	NS	1.74E-03	1.8
NM_010476	*Hsd17b7*	BXD100	6.24E-05	-1.7	6.28E-05	-1.8
NM_024255	*Hsdl2*	BXD100	4.95E-03	1.6	NS	NS
NM_010481	*Hspa9*	BXD100	1.03E-02	-1.4	NS	NS
NM_019564	*Htra1*	BXD100	2.87E-03	1.8	3.33E-03	1.8
NM_015755	*Hunk*	BXD100	2.29E-03	-1.9	NS	NS
NM_015790	*Icosl*	BXD100	1.43E-04	2.1	1.48E-02	1.7
NM_010495	*Id1*	BXD100	3.66E-04	2.1	NS	NS
NM_031166	*Id4*	BXD100	7.06E-03	2.2	NS	NS
NM_145360	*Idi1*	BXD100	1.41E-13	-2.9	3.06E-10	-2.4
NM_001101605	*Ifit1bl1*	BXD100	1.30E-03	2.3	NS	NS
NM_199157	*Ifnk*	BXD100	2.21E-02	1.9	NS	NS
NM_013562	*Ifrd1*	BXD100	3.23E-02	-1.6	3.65E-02	-1.5
NM_010515	*Igf2r*	BXD100	3.41E-02	1.5	NS	NS
NM_010518	*Igfbp5*	BXD100	2.83E-02	1.5	NS	NS
NM_001162884	*Igsf10*	BXD100	1.39E-02	1.7	NS	NS
NM_001123382	*Il1r1*	BXD100	1.78E-02	1.9	1.98E-02	1.9
NM_023626	*Ing3*	BXD100	NS	NS	3.95E-02	1.4
NM_172439	*Inpp5j*	BXD100	1.31E-02	1.7	1.04E-02	1.8
NM_153526	*Insig1*	BXD100	2.20E-07	-2.4	7.87E-07	-2.3
NM_010568	*Insr*	BXD100	1.97E-02	1.3	NS	NS
NM_029634	*Ip6k2*	BXD100	9.93E-03	1.4	NS	NS
NM_001164598	*Irf2bp2*	BXD100	NS	NS	6.18E-03	1.4

NM_016851	*Irf6*	BXD100	NS	NS	6.20E-03	1.8
NM_001081212	*Irs2*	BXD100	2.69E-02	1.5	NS	NS
NM_001276489	*Ism1*	BXD100	3.23E-02	1.8	NS	NS
NM_177290	*Itgb8*	BXD100	2.70E-02	1.6	NS	NS
NM_001168277	*Jazf1*	BXD100	3.41E-02	-1.5	NS	NS
NM_033398	*Jmjd6*	BXD100	NS	NS	2.66E-02	-1.4
NM_010591	*Jun*	BXD100	5.70E-17	4.0	1.05E-13	3.3
NM_001286944	*Jund*	BXD100	2.41E-05	1.8	2.63E-02	1.5
NM_001081134	*Kcng1*	BXD100	2.61E-05	-2.2	2.03E-03	-1.9
NM_008428	*Kcnj8*	BXD100	NS	NS	3.66E-02	-1.8
NM_001033525	*Kcnk6*	BXD100	NS	NS	2.20E-02	1.6
NM_032397	*Kcnn1*	BXD100	1.14E-02	-1.7	NS	NS
NM_026200	*Kcnv1*	BXD100	NS	NS	1.10E-02	-1.7
NM_026135	*Kctd16*	BXD100	NS	NS	2.66E-02	1.6
NM_026214	*Kctd4*	BXD100	NS	NS	6.39E-03	1.4
NM_011317	*Khdrbs1*	BXD100	NS	NS	2.41E-03	-1.5
NM_001145779	*Kif2a*	BXD100	NS	NS	3.44E-02	-1.3
NM_008451	*Klc2*	BXD100	4.40E-02	-1.3	NS	NS
NM_078477	*Klf16*	BXD100	8.43E-03	-1.6	1.75E-04	-1.9
NM_011803	*Klf6*	BXD100	NS	NS	1.82E-02	-1.5
NM_153128	*Klhl12*	BXD100	4.87E-02	1.5	NS	NS
NM_029436	*Klhl24*	BXD100	8.43E-03	1.4	1.22E-02	1.4
NM_178741	*Klhl8*	BXD100	NS	NS	4.66E-02	-1.5
NM_010655	*Kpna2*	BXD100	5.35E-03	-1.5	3.50E-03	-1.5
NM_008481	*Lama2*	BXD100	NS	NS	2.66E-02	1.8
NM_001077398	*Ldb2*	BXD100	NS	NS	3.71E-02	-1.5
NM_001252658	*Ldlr*	BXD100	6.94E-05	-2.4	2.00E-05	-2.5
NM_173012	*Letm2*	BXD100	2.21E-02	1.6	NS	NS
NM_134093	*Letmd1*	BXD100	4.07E-02	1.3	NS	NS
NM_008494	*Lfng*	BXD100	3.44E-06	1.9	NS	NS
NM_001199043	*Lgals8*	BXD100	NS	NS	1.69E-02	1.5
NM_001113386	*Lifr*	BXD100	4.95E-02	1.4	NS	NS
NM_013860	*Limd1*	BXD100	3.15E-02	1.9	NS	NS
NM_181074	*Lingo1*	BXD100	1.13E-04	-1.4	2.21E-02	-1.3
NM_001013758	*Lingo3*	BXD100	2.81E-02	-1.5	NS	NS
NM_201529	*Lmo7*	BXD100	NS	NS	4.59E-03	1.5
NM_001159577	*Lnx1*	BXD100	1.93E-02	1.7	NS	NS
NM_145376	*Lpcat1*	BXD100	NS	NS	2.00E-02	-1.3

NM_001145952	*Lpp*	BXD100	1.91E-02	1.7	2.27E-02	1.6
NM_001146048	*Lrrc1*	BXD100	3.46E-03	2.0	NS	NS
NM_145152	*Lrrc3*	BXD100	4.24E-02	-1.6	NS	NS
NM_133807	*Lrrc59*	BXD100	4.21E-02	-1.3	3.53E-02	-1.3
NM_010732	*Lrrn2*	BXD100	2.03E-03	-1.7	NS	NS
NM_001271416	*Ly6a*	BXD100	NS	NS	1.89E-03	2.3
NM_001252055	*Ly6c1*	BXD100	6.49E-04	2.6	2.30E-04	2.7
NM_053201	*Magee1*	BXD100	3.23E-02	-1.3	NS	NS
NM_001029850	*Magi1*	BXD100	4.48E-02	1.3	NS	NS
NM_001171187	*Mal*	BXD100	3.93E-02	1.7	1.09E-03	2.1
NM_010764	*Man2b1*	BXD100	NS	NS	4.00E-02	1.6
NM_011948	*Map3k4*	BXD100	NS	NS	1.04E-02	1.6
NM_011161	*Mapk11*	BXD100	NS	NS	4.09E-02	-1.6
NM_001045483	*Mapk1ip1*	BXD100	NS	NS	9.02E-03	1.4
NM_011841	*Mapk7*	BXD100	4.48E-03	-1.5	3.04E-03	-1.5
NM_175171	*Mast4*	BXD100	NS	NS	6.39E-03	1.4
NM_010770	*Matn3*	BXD100	NS	NS	4.95E-02	-1.6
NM_010773	*Mbd2*	BXD100	NS	NS	1.14E-02	1.4
NM_175341	*Mbnl2*	BXD100	NS	NS	6.89E-04	1.6
NM_008563	*Mcm3*	BXD100	1.03E-03	-2.0	NS	NS
NM_008566	*Mcm5*	BXD100	1.76E-02	-1.8	NS	NS
NM_008587	*Mertk*	BXD100	8.69E-03	2.0	2.08E-03	2.2
NM_008591	*Met*	BXD100	NS	NS	3.67E-02	-1.7
NM_027334	*Mettl7a1*	BXD100	7.89E-04	1.9	NS	NS
NM_029662	*Mfsd2a*	BXD100	1.20E-02	1.6	NS	NS
NM_023799	*Mgea5*	BXD100	1.95E-04	1.4	8.80E-04	1.4
NM_021565	*Midn*	BXD100	1.74E-02	-1.4	NS	NS
NM_001243584	*Mif4gd*	BXD100	2.00E-02	1.6	NS	NS
NM_001122667	*Mkl2*	BXD100	NS	NS	4.06E-02	1.3
NM_001285487	*Mknk1*	BXD100	3.57E-02	1.6	NS	NS
NM_008609	*Mmp15*	BXD100	9.04E-03	-1.6	6.60E-03	-1.6
NM_023773	*Mphosph8*	BXD100	NS	NS	1.15E-02	-1.5
NM_001101482	*Mrap2*	BXD100	8.30E-03	1.8	NS	NS
NM_027204	*Mrpl12*	BXD100	NS	NS	3.11E-02	-1.4
NM_026490	*Mrpl19*	BXD100	NS	NS	3.23E-02	-1.3
NM_053164	*Mrpl43*	BXD100	NS	NS	8.35E-04	-1.7
NM_026035	*Mrpl55*	BXD100	NS	NS	1.81E-02	-1.5
NM_025436	*Msmo1*	BXD100	2.39E-08	-2.6	1.27E-06	-2.4

NM_010834	*Mstn*	BXD100	3.41E-02	2.0	NS	NS
NM_013602	*Mt1*	BXD100	3.96E-07	2.0	7.56E-04	1.7
NM_008630	*Mt2*	BXD100	4.43E-10	3.6	4.00E-06	2.7
NM_023431	*Mum1*	BXD100	NS	NS	3.09E-02	-1.4
NM_001164630	*Mum1l1*	BXD100	4.19E-02	1.7	NS	NS
NM_138656	*Mvd*	BXD100	6.60E-07	-2.4	3.11E-07	-2.5
NM_023556	*Mvk*	BXD100	NS	NS	4.82E-02	-1.6
NM_010753	*Mxd4*	BXD100	6.64E-03	1.7	NS	NS
NM_001008542	*Mxi1*	BXD100	3.01E-11	2.1	2.55E-06	1.7
NM_001171615	*Myt1*	BXD100	2.00E-02	1.6	6.99E-03	1.6
NM_001024917	*N4bp2*	BXD100	6.11E-03	1.7	NS	NS
NM_030153	*Naa35*	BXD100	NS	NS	3.60E-02	-1.4
NM_001040395	*Nadk2*	BXD100	2.28E-03	1.5	NS	NS
NM_001081475	*Nasp*	BXD100	NS	NS	1.91E-02	-1.6
NM_001114085	*Nde1*	BXD100	4.19E-02	1.5	8.10E-03	1.7
NM_023239	*Ndnl2*	BXD100	8.07E-03	1.7	NS	NS
NM_001145959	*Ndrg2*	BXD100	NS	NS	2.91E-02	1.3
NM_027244	*Ndufa11*	BXD100	NS	NS	3.89E-02	-1.4
NM_026742	*Ndufaf4*	BXD100	1.80E-06	-1.8	1.44E-02	-1.4
NM_028757	*Nebl*	BXD100	NS	NS	4.44E-02	1.6
NM_001111324	*Nedd9*	BXD100	1.93E-02	1.5	NS	NS
NM_010904	*Nefh*	BXD100	3.40E-02	-1.8	NS	NS
NM_010914	*Nfyb*	BXD100	NS	NS	4.97E-02	-1.3
NM_001048168	*Nfyc*	BXD100	NS	NS	2.37E-02	-1.4
NM_026472	*Nifk*	BXD100	NS	NS	2.66E-02	-1.3
NM_207204	*Ninl*	BXD100	2.93E-02	-1.7	NS	NS
NM_145469	*Nipal2*	BXD100	1.44E-02	1.5	NS	NS
NM_028995	*Nipal3*	BXD100	1.30E-03	1.8	NS	NS
NM_001163387	*Nlgn1*	BXD100	NS	NS	1.94E-02	1.3
NM_029447	*Nln*	BXD100	2.17E-02	-1.5	NS	NS
NM_080637	*Nme5*	BXD100	4.99E-02	1.9	NS	NS
NM_021303	*Noc2l*	BXD100	NS	NS	8.35E-04	-1.6
NM_009834	*Noct*	BXD100	NS	NS	4.92E-04	-1.7
NM_001008421	*Nol10*	BXD100	1.30E-03	-1.8	5.32E-03	-1.7
NM_001001986	*Nol4l*	BXD100	1.74E-03	-1.6	3.62E-03	-1.6
NM_024193	*Nop56*	BXD100	2.87E-03	-1.7	NS	NS
NM_018868	*Nop58*	BXD100	NS	NS	1.45E-02	-1.4
NM_010342	*Npbwr1*	BXD100	3.93E-02	1.6	NS	NS

NM_028749	*Npl*	BXD100	4.19E-02	1.6	NS	NS
NM_008723	*Npm3*	BXD100	NS	NS	4.98E-03	-1.7
NM_015743	*Nr4a3*	BXD100	2.21E-02	-1.4	NS	NS
NM_010941	*Nsdhl*	BXD100	NS	NS	4.50E-03	-1.4
NM_001164363	*Nt5c2*	BXD100	3.49E-04	1.5	3.81E-02	1.3
NM_001190179	*Nup35*	BXD100	3.72E-02	-1.4	NS	NS
NM_008751	*Nxph1*	BXD100	NS	NS	2.57E-02	-1.4
NM_001161430	*Nxt2*	BXD100	2.51E-02	1.4	NS	NS
NM_146796	*Olfr1499*	BXD100	4.76E-02	-1.7	NS	NS
NM_145517	*Ormdl1*	BXD100	2.76E-02	1.6	NS	NS
NM_001134791	*Osbpl9*	BXD100	9.62E-06	1.5	3.53E-05	1.5
NM_001008497	*P2ry14*	BXD100	4.41E-02	1.9	NS	NS
NM_026420	*Paip2*	BXD100	2.61E-04	1.4	2.46E-02	1.3
NM_146169	*Paip2b*	BXD100	NS	NS	2.63E-02	1.5
NM_028829	*Paqr8*	BXD100	1.01E-03	1.6	2.12E-04	1.6
NM_177460	*Parp16*	BXD100	5.37E-04	1.9	NS	NS
NM_145619	*Parp3*	BXD100	NS	NS	4.95E-02	1.7
NM_011865	*Pcbp1*	BXD100	1.17E-02	-1.4	4.17E-02	-1.3
NM_183028	*Pcmtd1*	BXD100	NS	NS	3.79E-02	1.3
NM_153594	*Pcmtd2*	BXD100	2.98E-03	1.7	1.30E-02	1.6
NM_024229	*Pcyt2*	BXD100	9.15E-03	-1.6	NS	NS
NM_001177980	*Pde4b*	BXD100	8.97E-04	1.5	NS	NS
NM_008808	*Pdgfa*	BXD100	NS	NS	6.60E-03	-1.5
NM_011057	*Pdgfb*	BXD100	3.29E-02	-1.6	NS	NS
NM_001163794	*Pdik1l*	BXD100	NS	NS	4.95E-02	-1.4
NM_013743	*Pdk4*	BXD100	1.63E-02	1.7	NS	NS
NM_016861	*Pdlim1*	BXD100	4.07E-02	2.0	1.63E-02	2.1
NM_001159367	*Per1*	BXD100	7.59E-05	1.9	1.74E-03	1.7
NM_022889	*Pes1*	BXD100	2.21E-02	-1.3	NS	NS
NM_134025	*Pex12*	BXD100	4.76E-02	1.5	NS	NS
NM_025954	*Pgp*	BXD100	NS	NS	2.40E-02	-1.4
NM_008831	*Phb*	BXD100	NS	NS	5.04E-03	-1.4
NM_024250	*Phf10*	BXD100	2.07E-02	-1.4	NS	NS
NM_001109690	*Phf21a*	BXD100	4.68E-02	1.5	NS	NS
NM_053191	*Pi15*	BXD100	3.27E-02	1.8	NS	NS
NM_001145926	*Pianp*	BXD100	NS	NS	4.06E-02	-1.5
NM_178149	*Pik3ip1*	BXD100	1.55E-07	2.7	3.84E-04	2.0
NM_001024955	*Pik3r1*	BXD100	1.41E-13	2.1	1.87E-15	2.3

NM_145478	*Pim3*	BXD100	2.44E-02	-1.8	NS	NS
NM_028228	*Pinx1*	BXD100	NS	NS	1.04E-02	-1.6
NM_139269	*Pla2g16*	BXD100	2.00E-02	2.0	NS	NS
NM_001004762	*Pla2g4c*	BXD100	3.81E-02	-1.8	NS	NS
NM_008872	*Plat*	BXD100	5.23E-05	2.0	2.27E-02	1.6
NM_152813	*Plcd3*	BXD100	8.47E-03	1.7	1.53E-02	1.7
NM_001081456	*Plcd4*	BXD100	3.85E-03	1.8	2.78E-02	1.6
NM_001033253	*Plekhg1*	BXD100	1.90E-04	1.9	9.10E-06	2.1
NM_013807	*Plk3*	BXD100	1.48E-04	-1.9	1.08E-04	-1.9
NM_008884	*Pml*	BXD100	NS	NS	3.79E-02	1.5
NM_025443	*Pno1*	BXD100	NS	NS	4.90E-02	-1.5
NM_001033225	*Pnrc1*	BXD100	3.07E-02	1.3	NS	NS
NM_026383	*Pnrc2*	BXD100	6.79E-04	1.6	8.92E-03	1.4
NM_009086	*Polr1b*	BXD100	NS	NS	5.46E-03	-1.8
NM_029763	*Polr3f*	BXD100	NS	NS	3.35E-02	-1.4
NM_133249	*Ppargc1b*	BXD100	NS	NS	1.97E-02	-1.7
NM_008014	*Ppm1g*	BXD100	NS	NS	1.32E-03	-1.4
NM_008889	*Ppp1r14b*	BXD100	NS	NS	2.61E-02	-1.5
NM_027242	*Ppp1r35*	BXD100	NS	NS	2.92E-02	-1.6
NM_001161362	*Ppp2r3a*	BXD100	2.85E-02	1.4	NS	NS
NM_001164198	*Prkacb*	BXD100	1.06E-02	1.4	NS	NS
NM_001252476	*Prmt1*	BXD100	NS	NS	1.35E-03	-1.7
NM_145404	*Prmt7*	BXD100	4.54E-04	-1.6	2.59E-03	-1.5
NM_201371	*Prmt8*	BXD100	1.20E-04	-1.4	2.08E-03	-1.4
NM_027626	*Psd3*	BXD100	NS	NS	1.35E-02	1.4
NM_177698	*Psd3*	BXD100	NS	NS	3.32E-02	1.3
NM_011186	*Psmb5*	BXD100	NS	NS	3.09E-02	-1.4
NM_008950	*Psmc5*	BXD100	NS	NS	2.70E-02	-1.4
NM_025550	*Psmd6*	BXD100	NS	NS	2.21E-02	-1.4
NM_025682	*Pspc1*	BXD100	3.49E-02	-1.5	NS	NS
NM_133783	*Ptges2*	BXD100	NS	NS	2.04E-02	-1.6
NM_001109992	*Ptpn11*	BXD100	1.00E-02	1.3	NS	NS
NM_001163565	*Ptpn5*	BXD100	NS	NS	2.69E-02	-1.3
NM_025831	*Pxdc1*	BXD100	3.32E-02	1.5	NS	NS
NM_145458	*Pxk*	BXD100	1.15E-02	1.3	NS	NS
NM_029494	*Rab30*	BXD100	1.75E-06	1.9	1.59E-07	2.0
NM_029781	*Rab36*	BXD100	6.91E-03	1.5	NS	NS
NM_175122	*Rab39b*	BXD100	4.37E-02	1.3	NS	NS

NM_001038621	*Rabgap1l*	BXD100	2.86E-03	1.4	1.79E-04	1.5
NM_001099624	*Rapgef2*	BXD100	1.01E-08	1.8	1.30E-08	1.8
NM_001252494	*Rapgef6*	BXD100	9.78E-09	2.3	7.65E-07	2.1
NM_001176528	*Rara*	BXD100	1.34E-04	-1.8	NS	NS
NM_009025	*Rasa3*	BXD100	2.47E-02	-1.5	1.06E-03	-1.7
NM_011246	*Rasgrp1*	BXD100	1.54E-02	-1.5	NS	NS
NM_009029	*Rb1*	BXD100	2.21E-02	-1.3	NS	NS
NM_028234	*Rbm33*	BXD100	2.46E-03	1.5	3.81E-02	1.4
NM_001039080	*Rbms2*	BXD100	3.15E-02	1.7	NS	NS
NM_001081549	*Rcan1*	BXD100	3.43E-03	1.5	3.71E-02	1.4
NM_001286653	*Rcan2*	BXD100	1.76E-02	1.4	NS	NS
NM_009037	*Rcn1*	BXD100	2.56E-02	1.4	NS	NS
NM_026446	*Rgs19*	BXD100	5.69E-03	-1.7	NS	NS
NM_009062	*Rgs4*	BXD100	3.06E-03	1.6	9.73E-03	1.6
NM_007483	*Rhob*	BXD100	1.09E-02	1.4	NS	NS
NM_153514	*Rhobtb2*	BXD100	1.78E-02	-1.5	2.12E-02	-1.5
NM_024288	*Rmnd5a*	BXD100	NS	NS	3.67E-02	1.3
NM_011882	*Rnasel*	BXD100	NS	NS	6.99E-03	1.7
NM_026301	*Rnf125*	BXD100	2.00E-02	1.8	NS	NS
NM_025999	*Rnf141*	BXD100	NS	NS	4.82E-02	1.3
NM_001166553	*Rnf145*	BXD100	6.94E-05	-1.6	8.30E-05	-1.6
NM_001160368	*Rnf152*	BXD100	1.32E-02	1.6	NS	NS
NM_175388	*Rnf169*	BXD100	1.39E-02	1.5	NS	NS
NM_028783	*Robo4*	BXD100	1.96E-02	1.6	NS	NS
NM_001042556	*Rpf2*	BXD100	NS	NS	2.11E-02	-1.4
NM_133982	*Rpp25*	BXD100	NS	NS	2.08E-03	-1.8
NM_146244	*Rps6kl1*	BXD100	2.81E-02	1.6	3.81E-04	1.9
NM_199447	*Rrp12*	BXD100	1.63E-02	-1.7	4.25E-02	-1.7
NM_001081174	*Rsg1*	BXD100	9.04E-03	2.0	NS	NS
NM_001136227	*Rtkn*	BXD100	NS	NS	4.07E-02	1.5
NM_130892	*Rtn4ip1*	BXD100	NS	NS	4.40E-02	-1.5
NM_007901	*S1pr1*	BXD100	8.29E-03	1.7	NS	NS
NM_016926	*Sart3*	BXD100	3.30E-02	-1.5	NS	NS
NM_175155	*Sash1*	BXD100	4.46E-02	1.4	NS	NS
NM_172769	*Sc5d*	BXD100	NS	NS	1.99E-02	-1.3
NM_009127	*Scd1*	BXD100	9.02E-03	-1.5	NS	NS
NM_011324	*Scnn1a*	BXD100	4.87E-02	1.8	NS	NS
NM_027268	*Scrn1*	BXD100	3.83E-02	-1.2	NS	NS

NM_011521	*Sdc4*	BXD100	2.15E-06	2.9	1.80E-05	2.7
NM_024206	*Sec13*	BXD100	NS	NS	3.09E-02	-1.5
NM_011342	*Sec22b*	BXD100	4.50E-02	1.3	NS	NS
NM_172588	*Serinc5*	BXD100	NS	NS	2.65E-02	1.5
NM_001013370	*Sesn1*	BXD100	5.24E-03	1.6	NS	NS
NM_030261	*Sesn3*	BXD100	1.80E-06	1.7	1.15E-02	1.4
NM_001040398	*Setd1b*	BXD100	1.28E-02	-1.6	NS	NS
NM_001168525	*Sgms1*	BXD100	7.08E-04	2.2	4.40E-02	1.8
NM_009167	*Shc3*	BXD100	2.21E-02	-1.3	1.47E-02	-1.3
NM_001168505	*Shoc2*	BXD100	NS	NS	3.81E-02	-1.2
NM_001286538	*Sigmar1*	BXD100	8.85E-03	-1.5	1.15E-03	-1.6
NM_013612	*Slc11a1*	BXD100	NS	NS	2.03E-02	1.8
NM_027921	*Slc16a14*	BXD100	4.96E-03	1.6	NS	NS
NM_001029842	*Slc16a6*	BXD100	2.75E-02	1.9	NS	NS
NM_001159593	*Slc20a1*	BXD100	5.96E-03	-1.4	1.24E-02	-1.4
NM_138756	*Slc25a36*	BXD100	NS	NS	2.21E-02	1.4
NM_001009949	*Slc25a51*	BXD100	2.83E-02	1.4	NS	NS
NM_011977	*Slc27a1*	BXD100	1.42E-02	1.4	NS	NS
NM_007854	*Slc29a2*	BXD100	NS	NS	2.21E-02	-1.7
NM_023596	*Slc29a3*	BXD100	6.27E-03	1.5	8.91E-03	1.5
NM_011400	*Slc2a1*	BXD100	1.10E-06	2.4	4.63E-03	1.8
NM_175121	*Slc38a2*	BXD100	3.70E-02	1.3	NS	NS
NM_001012305	*Slc39a12*	BXD100	9.04E-03	1.7	4.07E-02	1.5
NM_026244	*Slc39a9*	BXD100	2.29E-02	1.3	NS	NS
NM_001199283	*Slc43a2*	BXD100	1.53E-04	1.8	2.94E-03	1.6
NM_001081263	*Slc44a5*	BXD100	2.87E-06	1.9	1.65E-08	2.2
NM_029643	*Slc52a2*	BXD100	4.66E-02	-1.5	3.50E-02	-1.5
NM_001164819	*Slc52a3*	BXD100	NS	NS	1.95E-02	1.7
NM_007515	*Slc7a3*	BXD100	2.22E-02	1.6	NS	NS
NM_012030	*Slc9a3r1*	BXD100	3.69E-02	-1.4	NS	NS
NM_177909	*Slc9a9*	BXD100	2.99E-02	1.5	NS	NS
NM_001038643	*Slco3a1*	BXD100	2.59E-03	-1.5	2.38E-03	-1.5
NM_008539	*Smad1*	BXD100	9.10E-05	1.6	NS	NS
NM_001252481	*Smad2*	BXD100	4.87E-03	-1.5	1.85E-02	-1.5
NM_031842	*Smarcd1*	BXD100	NS	NS	4.07E-02	-1.3
NM_134133	*Smim3*	BXD100	NS	NS	3.20E-02	1.9
NM_026796	*Smyd2*	BXD100	NS	NS	3.37E-03	-1.5
NM_009226	*Snrpd1*	BXD100	NS	NS	1.30E-02	-1.5

NM_026095	*Snrpd3*	BXD100	3.72E-02	-1.3	NS	NS
NM_001190156	*Snx7*	BXD100	3.41E-02	-1.4	NS	NS
NM_001034962	*Sorbs1*	BXD100	4.84E-03	1.4	1.82E-03	1.4
NM_146126	*Sord*	BXD100	1.38E-03	1.8	NS	NS
NM_001289464	*Sox17*	BXD100	NS	NS	2.35E-02	1.7
NM_009236	*Sox18*	BXD100	NS	NS	2.87E-02	1.9
NM_001025428	*Spag9*	BXD100	6.92E-04	1.5	1.80E-03	1.4
NM_182927	*Spred3*	BXD100	4.54E-04	-2.2	2.86E-04	-2.3
NM_011898	*Spry4*	BXD100	1.47E-04	-2.1	2.08E-03	-1.9
NM_009270	*Sqle*	BXD100	1.14E-03	-1.6	4.09E-06	-1.8
NM_033218	*Srebf2*	BXD100	NS	NS	1.84E-02	-1.3
NM_009272	*Srm*	BXD100	1.80E-03	-1.5	1.03E-03	-1.5
NM_016795	*Srpk1*	BXD100	7.59E-05	-1.7	2.35E-02	-1.4
NM_001080387	*Srsf10*	BXD100	3.41E-02	-1.4	NS	NS
NM_011358	*Srsf2*	BXD100	4.72E-03	-1.5	2.87E-02	-1.4
NM_001195485	*Srsf7*	BXD100	2.21E-02	-1.6	1.99E-02	-1.6
NM_001252505	*St6gal1*	BXD100	3.75E-02	1.5	NS	NS
NM_013666	*St8sia5*	BXD100	NS	NS	4.07E-02	-1.6
NM_133774	*Stard4*	BXD100	1.08E-04	-2.0	6.48E-05	-2.1
NM_019675	*Stmn4*	BXD100	NS	NS	3.32E-02	1.5
NM_013515	*Stom*	BXD100	2.85E-02	1.9	3.71E-02	1.9
NM_153563	*Strip1*	BXD100	1.53E-04	-1.5	1.06E-03	-1.4
NM_011505	*Stxbp4*	BXD100	7.67E-03	1.6	4.88E-02	1.5
NM_133670	*Sult1a1*	BXD100	3.53E-03	2.8	2.04E-02	2.4
NM_001164495	*Syn3*	BXD100	1.48E-02	1.4	3.19E-02	1.4
NM_153399	*Syne1*	BXD100	NS	NS	2.45E-02	1.5
NM_001005510	*Syne2*	BXD100	1.41E-02	1.5	NS	NS
NM_001040085	*Sytl2*	BXD100	6.02E-03	1.8	2.22E-04	2.2
NM_020024	*Taf10*	BXD100	NS	NS	2.39E-02	-1.3
NM_001177798	*Taf6l*	BXD100	NS	NS	2.39E-02	-1.7
NM_181275	*Tas2r139*	BXD100	3.41E-02	1.7	NS	NS
NM_146008	*Tcp11l2*	BXD100	3.29E-02	1.6	NS	NS
NM_009368	*Tgfb3*	BXD100	3.41E-02	2.0	NS	NS
NM_009381	*Thrsp*	BXD100	3.09E-02	1.6	NS	NS
NM_144543	*Thyn1*	BXD100	NS	NS	4.50E-02	-1.4
NM_026708	*Tlcd1*	BXD100	NS	NS	3.44E-02	-1.4
NM_133211	*Tlr7*	BXD100	NS	NS	1.95E-02	1.9
NM_177412	*Tmcc1*	BXD100	NS	NS	2.43E-02	1.4

NM_172541	*Tmem120a*	BXD100	4.46E-03	-1.7	NS	NS
NM_177344	*Tmem203*	BXD100	NS	NS	1.99E-02	-1.7
NM_183160	*Tmem252*	BXD100	4.46E-02	1.7	NS	NS
NM_001289727	*Tmem255a*	BXD100	2.99E-02	2.1	NS	NS
NM_001081283	*Tmem28*	BXD100	NS	NS	4.17E-02	-1.6
NM_177861	*Tmem67*	BXD100	6.90E-03	1.7	NS	NS
NM_021793	*Tmem8*	BXD100	4.36E-02	1.7	NS	NS
NM_133706	*Tmem97*	BXD100	NS	NS	2.01E-02	-1.7
NM_011607	*Tnc*	BXD100	9.63E-03	-1.6	NS	NS
NM_001048267	*Tnpo1*	BXD100	1.17E-04	1.6	1.79E-04	1.6
NM_144812	*Tnrc6b*	BXD100	2.76E-02	1.5	2.47E-03	1.6
NM_001109748	*Tomm40*	BXD100	5.91E-05	-1.6	NS	NS
NM_001170488	*Tprkb*	BXD100	3.29E-02	1.5	NS	NS
NM_029979	*Trim35*	BXD100	NS	NS	4.17E-02	-1.4
NM_001170855	*Trim36*	BXD100	NS	NS	1.07E-02	-2.1
NM_178110	*Trim62*	BXD100	1.45E-02	-1.5	NS	NS
NM_001167994	*Trmt2b*	BXD100	2.81E-02	1.7	NS	NS
NM_001099792	*Trmt61a*	BXD100	3.06E-03	-1.7	NS	NS
NM_013735	*Trp53bp1*	BXD100	NS	NS	2.76E-02	-1.4
NM_173378	*Trp53bp2*	BXD100	1.84E-02	1.6	NS	NS
NM_001282086	*Trpc6*	BXD100	2.85E-02	-1.7	NS	NS
NM_032000	*Trps1*	BXD100	NS	NS	4.13E-02	1.4
NM_001177751	*Tsc22d1*	BXD100	2.21E-02	1.2	NS	NS
NM_001077364	*Tsc22d3*	BXD100	7.23E-03	1.8	NS	NS
NM_183099	*Tssk5*	BXD100	3.83E-02	1.4	NS	NS
NM_146116	*Tubb4b*	BXD100	NS	NS	4.34E-03	-1.4
NM_023053	*Twsg1*	BXD100	3.06E-02	1.4	NS	NS
NM_001009935	*Txnip*	BXD100	8.40E-10	5.5	1.30E-08	4.9
NM_176860	*Ubash3b*	BXD100	3.90E-03	-1.7	NS	NS
NM_001169576	*Ube2h*	BXD100	1.81E-04	1.6	1.95E-05	1.7
NM_016723	*Uchl3*	BXD100	4.36E-02	-1.4	NS	NS
NM_030724	*Uck2*	BXD100	1.59E-02	-1.3	4.49E-02	-1.3
NM_001081132	*Upf2*	BXD100	3.55E-03	1.7	9.96E-03	1.6
NM_009462	*Usp10*	BXD100	1.45E-02	-1.3	2.38E-02	-1.3
NM_030180	*Usp54*	BXD100	3.96E-02	1.6	3.03E-03	1.8
NM_001013375	*Utp18*	BXD100	2.21E-02	-1.4	NS	NS
NM_175158	*Utp20*	BXD100	NS	NS	1.22E-02	-1.7
NM_001039385	*Vgf*	BXD100	2.10E-03	-2.2	2.17E-03	-2.2

NM_145967	*Vstm2a*	BXD100	NS	NS	2.20E-03	-1.4
NM_001172152	*Wdr17*	BXD100	NS	NS	3.60E-02	-1.4
NM_175450	*Wdr18*	BXD100	NS	NS	4.01E-02	-1.5
NM_020603	*Wdr46*	BXD100	1.94E-02	-1.7	1.97E-02	-1.7
NM_134139	*Wdr74*	BXD100	NS	NS	4.06E-02	-1.4
NM_009516	*Wee1*	BXD100	NS	NS	3.61E-02	-1.4
NM_023395	*Wfdc1*	BXD100	NS	NS	2.11E-02	1.8
NM_011273	*Xpr1*	BXD100	2.45E-02	1.3	NS	NS
NM_001005341	*Ypel2*	BXD100	3.13E-02	1.8	NS	NS
NM_025347	*Ypel3*	BXD100	4.42E-02	1.3	NS	NS
NM_001005342	*Ypel4*	BXD100	2.77E-06	3.1	1.11E-05	2.9
NM_027166	*Ypel5*	BXD100	1.75E-06	1.7	8.85E-05	1.6
NM_001163013	*Ythdc2*	BXD100	5.12E-03	-1.7	NS	NS
NM_028106	*Zbed3*	BXD100	3.55E-03	1.6	NS	NS
NM_173181	*Zc2hc1a*	BXD100	NS	NS	2.57E-02	-1.4
NM_028325	*Zcchc12*	BXD100	2.67E-02	1.9	NS	NS
NM_001113399	*Zfp385b*	BXD100	4.00E-02	1.4	NS	NS
NM_001110309	*Zfp426*	BXD100	2.12E-03	1.6	6.60E-03	1.5
NM_175513	*Zfp804a*	BXD100	6.53E-03	-1.4	NS	NS
NM_001267583	*Zfp811*	BXD100	NS	NS	4.32E-03	1.6
NM_001105557	*Zfp938*	BXD100	4.55E-02	1.3	4.92E-02	1.3
NM_172738	*Zfp954*	BXD100	1.25E-02	1.6	NS	NS
NM_183154	*Zfyve1*	BXD100	3.84E-02	1.4	NS	NS
NM_001252584	*Zmynd8*	BXD100	NS	NS	2.03E-03	1.7
NM_001205367	*-44446*	BXD60	8.80E-03	-1.3	NS	NS
NM_021446	*0610007P14Rik*	BXD60	NS	NS	3.71E-02	-1.3
NM_001083880	*1600012H06Rik*	BXD60	1.54E-02	1.5	NS	NS
NM_028820	*1700017B05Rik*	BXD60	1.92E-02	1.4	NS	NS
NM_144953	*1700019D03Rik*	BXD60	NS	NS	4.53E-02	1.6
NR_045177	*2900055J20Rik*	BXD60	2.14E-03	1.7	NS	NS
NM_027017	*3300002I08Rik*	BXD60	1.09E-02	1.6	NS	NS
NM_001033273	*5031439G07Rik*	BXD60	4.54E-04	1.4	3.14E-02	1.3
NM_001033304	*5330417C22Rik*	BXD60	8.26E-03	1.6	NS	NS
NM_027472	*5730455P16Rik*	BXD60	6.84E-04	1.6	NS	NS
NM_029964	*6030468B19Rik*	BXD60	9.80E-03	-1.7	NS	NS
NM_001123370	*9030025P20Rik*	BXD60	1.16E-02	1.5	NS	NS
NM_001142969	*A630073D07Rik*	BXD60	1.77E-02	2.6	NS	NS
NM_001167925	*A730017C20Rik*	BXD60	3.93E-05	1.4	1.67E-02	1.2

NR_015488	*A930003A15Rik*	BXD60	4.58E-02	1.7	1.52E-07	3.2
NR_033609	*A930017M01Rik*	BXD60	3.98E-05	2.1	1.20E-03	1.9
NM_001033336	*Abcc4*	BXD60	3.00E-02	1.7	NS	NS
NM_008991	*Abcd3*	BXD60	3.92E-02	-1.3	NS	NS
NM_009593	*Abcg1*	BXD60	1.35E-02	1.8	NS	NS
NM_001110271	*Abhd14a*	BXD60	1.62E-02	1.6	NS	NS
NM_134130	*Abhd3*	BXD60	1.41E-02	1.7	NS	NS
NM_025341	*Abhd6*	BXD60	2.73E-02	1.4	NS	NS
NM_025826	*Acadsb*	BXD60	NS	NS	4.06E-03	1.7
NM_009338	*Acat2*	BXD60	2.21E-02	-1.4	NS	NS
NM_153151	*Acat3*	BXD60	4.05E-02	-1.4	NS	NS
NM_139306	*Acer2*	BXD60	2.08E-02	2.0	NS	NS
NM_001199296	*Acly*	BXD60	4.85E-02	-1.3	NS	NS
NM_001270996	*Adam9*	BXD60	1.47E-03	1.4	NS	NS
NM_001163290	*Adck3*	BXD60	1.66E-02	1.7	NS	NS
NM_001025372	*Adcyap1r1*	BXD60	1.50E-03	1.5	NS	NS
NM_054053	*Adgrv1*	BXD60	1.43E-02	2.0	NS	NS
NM_001289656	*Agbl3*	BXD60	3.07E-02	1.5	NS	NS
NM_178162	*Agfg2*	BXD60	5.75E-03	-1.8	7.21E-03	-1.8
NM_153177	*Ago4*	BXD60	4.25E-02	1.6	NS	NS
NM_026644	*Agpat4*	BXD60	1.97E-02	1.3	1.27E-02	1.4
uc008hme.1	*AI606181*	BXD60	2.68E-03	1.9	NS	NS
NM_001172146	*Aimp2*	BXD60	1.06E-03	-1.9	NS	NS
NM_001035532	*Akap2*	BXD60	1.04E-03	1.6	NS	NS
NM_017476	*Akap8l*	BXD60	7.57E-04	1.5	NS	NS
NM_019698	*Aldh18a1*	BXD60	3.25E-02	1.7	NS	NS
NM_009657	*Aldoc*	BXD60	2.28E-03	1.4	NS	NS
NM_025538	*Alkbh7*	BXD60	NS	NS	2.38E-02	-1.5
NM_001159948	*Als2*	BXD60	4.93E-02	1.4	NS	NS
NM_001276301	*Ampd3*	BXD60	8.24E-04	1.9	NS	NS
NM_001190371	*Ankrd29*	BXD60	3.95E-03	1.6	NS	NS
NM_001024851	*Ankrd34a*	BXD60	1.46E-02	-1.8	NS	NS
ENSMUST00000105593	*Ankrd65*	BXD60	4.39E-02	-1.6	NS	NS
NM_001128103	*Ano3*	BXD60	5.19E-03	2.0	NS	NS
NM_001253813	*Ano6*	BXD60	3.69E-02	1.4	NS	NS
NM_007460	*Ap3d1*	BXD60	1.74E-02	-1.2	NS	NS
NM_011789	*Apc2*	BXD60	3.87E-02	1.5	NS	NS

NM_026674	*Aph1c*	BXD60	4.91E-02	1.4	NS	NS
NM_001109914	*Apold1*	BXD60	5.95E-03	1.9	9.96E-03	1.9
NM_175105	*Aqp11*	BXD60	2.70E-04	1.7	NS	NS
NM_009705	*Arg2*	BXD60	2.78E-02	1.7	NS	NS
NM_001172205	*Arid5a*	BXD60	7.11E-07	3.1	2.86E-03	2.2
NM_023598	*Arid5b*	BXD60	5.94E-04	1.7	1.29E-03	1.7
NM_172595	*Arl15*	BXD60	4.79E-02	1.5	NS	NS
NM_027870	*Armcx3*	BXD60	6.62E-03	1.4	NS	NS
NM_007488	*Arnt2*	BXD60	1.26E-02	-1.4	NS	NS
NM_001289679	*Arntl2*	BXD60	1.55E-03	2.4	NS	NS
NM_001042591	*Arrdc3*	BXD60	4.01E-13	4.1	7.09E-07	2.6
NM_001042592	*Arrdc4*	BXD60	5.90E-04	1.9	2.51E-02	1.7
NM_007492	*Arx*	BXD60	NS	NS	8.49E-03	-1.8
NM_001276461	*Asap1*	BXD60	3.08E-02	-1.3	NS	NS
NM_001029895	*Ate1*	BXD60	NS	NS	3.18E-02	-1.3
NM_001287180	*Atf4*	BXD60	1.64E-03	-1.6	2.04E-02	-1.5
NM_001081304	*Atf6*	BXD60	5.08E-03	1.4	NS	NS
NM_019426	*Atf7ip*	BXD60	NS	NS	3.68E-02	1.5
NM_026217	*Atg12*	BXD60	6.84E-04	1.4	2.02E-02	1.3
NM_026402	*Atg3*	BXD60	3.49E-02	-1.3	NS	NS
NM_001145967	*Atg4c*	BXD60	2.28E-02	1.5	NS	NS
NM_007881	*Atn1*	BXD60	4.17E-02	-1.3	NS	NS
NM_001243049	*Atp6v0a1*	BXD60	1.56E-02	1.3	NS	NS
NM_025272	*Atp6v0e*	BXD60	5.54E-04	2.1	NS	NS
NM_027855	*Atraid*	BXD60	8.20E-04	1.5	4.67E-02	1.4
NM_009125	*Atxn2*	BXD60	7.56E-04	-1.4	1.63E-02	-1.3
NM_016847	*Avpr1a*	BXD60	3.68E-02	-1.6	NS	NS
NM_001104646	*AW209491*	BXD60	1.19E-02	1.5	NS	NS
NM_001122993	*B3galt5*	BXD60	5.12E-06	2.2	NS	NS
NM_001159407	*B3gnt5*	BXD60	6.87E-03	1.6	1.69E-02	1.6
NM_145222	*B3gnt7*	BXD60	3.49E-02	-1.5	NS	NS
NM_007519	*Baat*	BXD60	4.36E-02	1.6	NS	NS
NM_013863	*Bag3*	BXD60	3.75E-02	1.6	NS	NS
NM_027404	*Bag5*	BXD60	1.05E-02	-1.5	NS	NS
NM_175325	*Bbs4*	BXD60	5.95E-03	1.3	1.30E-02	1.4
NM_027444	*Bbx*	BXD60	3.10E-03	1.8	NS	NS
NM_001001332	*BC117090*	BXD60	4.96E-02	-1.8	NS	NS
NM_001024468	*Bcat1*	BXD60	2.22E-06	1.7	NS	NS

NM_001284410	*Bcl2l11*	BXD60	5.60E-06	2.7	6.07E-07	3.0
NM_001277216	*Bmpr1b*	BXD60	5.93E-03	1.8	2.77E-02	1.7
NM_026396	*Brix1*	BXD60	3.36E-02	-1.4	NS	NS
NM_001037756	*Brms1l*	BXD60	2.40E-02	-1.4	NS	NS
NM_133889	*Bsdc1*	BXD60	5.12E-06	1.6	4.24E-03	1.4
NM_027060	*Btbd9*	BXD60	9.03E-05	1.6	NS	NS
NM_007570	*Btg2*	BXD60	4.49E-05	2.1	1.39E-03	1.9
NM_009770	*Btg3*	BXD60	6.02E-03	1.8	4.22E-03	1.9
NM_016859	*Bysl*	BXD60	NS	NS	4.12E-02	-1.6
NM_001252533	*Cacnb2*	BXD60	5.62E-03	1.4	NS	NS
NM_001199301	*Cacng5*	BXD60	2.29E-03	1.8	NS	NS
NM_026192	*Calcoco1*	BXD60	1.09E-03	1.6	NS	NS
NM_133926	*Camk1*	BXD60	1.74E-02	-1.6	NS	NS
NM_144817	*Camk1g*	BXD60	2.41E-05	-1.7	9.32E-04	-1.6
NM_009797	*Capza1*	BXD60	3.50E-02	-1.4	NS	NS
NM_001271353	*Cbs*	BXD60	2.81E-02	1.4	NS	NS
NM_007625	*Cbx4*	BXD60	NS	NS	1.29E-02	-1.4
NM_026201	*Ccar1*	BXD60	NS	NS	4.84E-02	-1.4
NM_029779	*Ccdc116*	BXD60	8.25E-03	-1.7	NS	NS
NM_177716	*Ccdc184*	BXD60	3.10E-03	-1.6	NS	NS
NM_144820	*Ccdc28a*	BXD60	1.81E-02	1.8	4.97E-02	1.7
NM_026009	*Ccdc47*	BXD60	2.99E-03	1.4	NS	NS
NM_025602	*Ccdc59*	BXD60	NS	NS	3.14E-02	-1.6
NM_007631	*Ccnd1*	BXD60	1.40E-03	-1.6	4.13E-04	-1.7
NM_009831	*Ccng1*	BXD60	1.30E-02	1.3	NS	NS
NM_007635	*Ccng2*	BXD60	5.26E-04	1.7	NS	NS
NM_025422	*Cd302*	BXD60	8.51E-04	1.9	NS	NS
NM_001111060	*Cd59a*	BXD60	1.40E-02	2.0	NS	NS
NM_001042605	*Cd74*	BXD60	7.93E-03	2.6	NS	NS
NM_001289915	*Cd83*	BXD60	3.49E-02	1.6	NS	NS
NM_177613	*Cdc34*	BXD60	2.79E-02	-1.6	NS	NS
NM_001161623	*Cdc45*	BXD60	4.66E-02	1.5	NS	NS
NM_007661	*Cdk11b*	BXD60	1.13E-02	-1.5	NS	NS
NM_001109626	*Cdk12*	BXD60	9.09E-03	1.6	NS	NS
NM_001168304	*Cdk19*	BXD60	3.61E-02	1.3	3.96E-03	1.5
NM_016756	*Cdk2*	BXD60	NS	NS	4.29E-02	-1.6
NM_009874	*Cdk7*	BXD60	6.75E-04	-1.7	NS	NS
NM_021339	*Cdon*	BXD60	4.59E-02	1.6	NS	NS

NM_026014	*Cdt1*	BXD60	NS	NS	1.23E-02	-1.8
NM_001134426	*Cdv3*	BXD60	8.20E-04	-1.4	NS	NS
NM_001159364	*Cep97*	BXD60	4.66E-02	1.5	NS	NS
NM_001081345	*Chd2*	BXD60	1.68E-03	1.5	2.62E-02	1.4
NM_138585	*Cherp*	BXD60	3.00E-02	-1.3	NS	NS
NM_009767	*Chic1*	BXD60	5.65E-03	1.8	NS	NS
NM_026885	*Chmp2a*	BXD60	4.89E-02	1.4	NS	NS
NM_023215	*Chtop*	BXD60	NS	NS	1.51E-02	-1.3
NM_025296	*Ciao1*	BXD60	3.83E-02	1.5	NS	NS
NM_001033302	*Ciart*	BXD60	6.96E-03	-2.3	1.24E-02	-2.3
NM_025854	*Cir1*	BXD60	NS	NS	2.51E-02	1.4
NM_001177770	*Clcc1*	BXD60	6.84E-04	1.9	NS	NS
NM_007711	*Clcn3*	BXD60	2.35E-02	1.3	NS	NS
NM_001243762	*Clcn5*	BXD60	9.20E-09	2.6	2.03E-03	1.8
NM_009902	*Cldn3*	BXD60	6.76E-03	2.1	NS	NS
NM_009191	*Clpb*	BXD60	4.59E-02	1.4	NS	NS
NM_024217	*Cmtm3*	BXD60	1.90E-02	1.4	NS	NS
NM_026066	*Cmtm5*	BXD60	2.40E-03	1.5	NS	NS
NM_007725	*Cnn2*	BXD60	3.00E-02	-1.6	NS	NS
NM_033570	*Cnnm4*	BXD60	1.35E-03	2.0	NS	NS
NM_026977	*Cnppd1*	BXD60	1.17E-03	1.6	NS	NS
NM_178612	*Cnpy4*	BXD60	8.91E-03	1.5	NS	NS
NM_001081653	*Cntnap5c*	BXD60	4.97E-02	1.4	NS	NS
NM_178599	*Commd8*	BXD60	4.91E-03	1.4	NS	NS
NM_001039710	*Coq10b*	BXD60	NS	NS	3.52E-02	-1.5
NM_009898	*Coro1a*	BXD60	NS	NS	2.38E-02	-1.7
NM_001252525	*Cpeb1*	BXD60	3.14E-02	1.7	NS	NS
NM_011957	*Creb3l1*	BXD60	4.90E-02	-1.7	NS	NS
NM_172728	*Creb5*	BXD60	NS	NS	3.84E-02	1.8
NM_170597	*Creg2*	BXD60	4.82E-02	1.5	NS	NS
NM_001110850	*Crem*	BXD60	2.14E-03	1.7	3.46E-05	2.1
NM_007762	*Crhr1*	BXD60	3.92E-03	-1.8	NS	NS
NM_009963	*Cry2*	BXD60	2.13E-03	1.4	NS	NS
NM_023565	*Cse1l*	BXD60	1.74E-02	-1.3	NS	NS
NM_026493	*Cspp1*	BXD60	2.40E-02	-1.5	NS	NS
NM_153409	*Csrnp3*	BXD60	3.98E-03	1.4	NS	NS
NM_007791	*Csrp1*	BXD60	2.73E-02	1.6	NS	NS
NM_016748	*Ctps*	BXD60	1.95E-02	-1.5	NS	NS

NM_009984	*Ctsl*	BXD60	3.50E-02	1.4	NS	NS
NM_009994	*Cyp1b1*	BXD60	2.76E-02	2.0	NS	NS
NM_028979	*Cyp2j9*	BXD60	7.31E-05	2.2	4.50E-02	1.6
NM_134127	*Cyp4f15*	BXD60	1.95E-02	1.8	NS	NS
NM_020010	*Cyp51*	BXD60	4.84E-03	-1.4	2.70E-02	-1.3
NM_001033279	*D17Wsu92e*	BXD60	6.61E-03	1.4	NS	NS
NM_001008231	*Daam2*	BXD60	2.14E-02	1.5	NS	NS
NM_172826	*Dact2*	BXD60	7.92E-04	1.9	NS	NS
NM_198114	*Dagla*	BXD60	2.79E-02	1.5	NS	NS
NM_001285917	*Dapk1*	BXD60	2.28E-02	1.3	NS	NS
NM_011932	*Dapp1*	BXD60	2.20E-02	1.7	NS	NS
NM_001195496	*Dclk2*	BXD60	1.29E-02	1.3	NS	NS
NM_001080995	*Ddias*	BXD60	3.52E-02	1.6	4.26E-02	1.7
NM_019553	*Ddx21*	BXD60	8.63E-03	-1.5	1.36E-04	-1.8
NM_001252457	*Ddx39b*	BXD60	NS	NS	2.29E-03	-1.4
NM_001037752	*Defb45*	BXD60	4.68E-02	-1.9	NS	NS
NM_172477	*Dennd2a*	BXD60	4.87E-02	1.6	NS	NS
NM_033562	*Derl2*	BXD60	4.82E-02	1.4	NS	NS
NM_053272	*Dhcr24*	BXD60	2.64E-06	-1.6	2.13E-06	-1.6
NM_025522	*Dhrs7*	BXD60	2.08E-02	1.6	NS	NS
NM_172594	*Dhx29*	BXD60	3.85E-03	-1.5	NS	NS
NM_025447	*Dimt1*	BXD60	1.33E-02	-1.4	NS	NS
NM_025926	*Dnajb4*	BXD60	2.14E-03	1.5	5.93E-04	1.5
NM_001163026	*Dnajc13*	BXD60	5.95E-03	-1.5	NS	NS
NM_029669	*Dnajc18*	BXD60	4.33E-02	1.3	NS	NS
NM_009584	*Dnajc2*	BXD60	5.98E-03	-1.5	NS	NS
NM_001099738	*Dnajc28*	BXD60	3.31E-02	1.6	NS	NS
NM_001199431	*Dnmt1*	BXD60	NS	NS	2.67E-03	-1.8
NM_172803	*Dock4*	BXD60	2.71E-02	1.4	NS	NS
NM_007875	*Dpagt1*	BXD60	4.33E-02	1.5	NS	NS
NM_172624	*Dpp9*	BXD60	3.88E-02	-1.5	NS	NS
NM_013503	*Drd5*	BXD60	2.79E-02	1.6	NS	NS
NM_023842	*Dsp*	BXD60	1.40E-02	1.9	NS	NS
NM_001285807	*Dtna*	BXD60	7.58E-07	1.6	5.79E-03	1.4
NM_172442	*Dtx4*	BXD60	8.86E-05	1.6	NS	NS
NM_013642	*Dusp1*	BXD60	2.84E-10	2.8	9.07E-09	2.6
NM_019819	*Dusp14*	BXD60	1.80E-02	1.7	NS	NS
NM_001048054	*Dusp16*	BXD60	2.26E-06	2.5	2.28E-04	2.1

NM_176933	*Dusp4*	BXD60	NS	NS	4.84E-02	-1.6
NM_001085390	*Dusp5*	BXD60	2.13E-02	-1.6	NS	NS
NM_026268	*Dusp6*	BXD60	1.51E-03	-1.7	NS	NS
NM_008748	*Dusp8*	BXD60	2.21E-10	1.8	4.97E-07	1.6
NM_172859	*Dzank1*	BXD60	3.18E-02	1.6	NS	NS
NM_001110331	*Eci2*	BXD60	2.29E-03	1.5	NS	NS
NM_145537	*Edem2*	BXD60	NS	NS	3.14E-02	1.4
NM_010104	*Edn1*	BXD60	7.18E-03	2.4	4.38E-03	2.7
NM_025380	*Eef1e1*	BXD60	6.82E-05	-1.6	NS	NS
NM_025994	*Efhd2*	BXD60	NS	NS	3.86E-02	-1.6
NM_010111	*Efnb2*	BXD60	1.95E-02	-1.5	NS	NS
NM_019397	*Egfl6*	BXD60	6.75E-04	2.2	NS	NS
NM_001289925	*Egr3*	BXD60	4.61E-06	-2.5	1.61E-04	-2.2
NM_018781	*Egr3*	BXD60	NS	NS	3.46E-02	-1.7
NM_010121	*Eif2ak3*	BXD60	4.58E-02	1.4	1.54E-02	1.6
NM_016876	*Eif3g*	BXD60	5.19E-03	-1.5	NS	NS
NM_145625	*Eif4b*	BXD60	2.16E-02	1.3	NS	NS
NM_175522	*Elfn1*	BXD60	8.90E-04	-2.0	NS	NS
NM_001170691	*Elmod2*	BXD60	8.48E-03	1.5	NS	NS
NM_001253692	*Elmod3*	BXD60	1.64E-04	1.7	NS	NS
NM_175101	*Emc3*	BXD60	1.72E-02	1.3	NS	NS
NM_198299	*Enkd1*	BXD60	3.43E-02	-1.5	NS	NS
NM_001168620	*Enpp5*	BXD60	2.51E-02	1.3	NS	NS
NM_001026214	*Entpd5*	BXD60	7.15E-04	1.8	NS	NS
NM_175313	*Eogt*	BXD60	7.88E-03	1.6	NS	NS
NM_013512	*Epb41l4a*	BXD60	2.47E-03	-1.5	NS	NS
NM_013513	*Epb42*	BXD60	1.76E-02	-1.7	NS	NS
NM_007938	*Epha6*	BXD60	3.80E-02	1.6	NS	NS
NM_015769	*Ercc4*	BXD60	1.19E-02	1.5	NS	NS
NM_010155	*Erf*	BXD60	3.09E-02	-1.6	NS	NS
NM_001034891	*Ermard*	BXD60	1.40E-02	1.5	NS	NS
NM_023913	*Ern1*	BXD60	2.28E-02	1.4	NS	NS
NM_029250	*Etnk1*	BXD60	4.25E-02	1.3	NS	NS
NM_001163154	*Etv1*	BXD60	9.03E-05	-1.8	NS	NS
NM_001083318	*Etv3*	BXD60	2.72E-02	1.6	NS	NS
NM_008815	*Etv4*	BXD60	9.18E-04	-1.7	2.04E-03	-1.7
NM_023794	*Etv5*	BXD60	5.25E-06	-2.2	3.12E-04	-1.9
NM_001283061	*Ewsr1*	BXD60	1.23E-02	-1.6	NS	NS

NM_001163514	*Extl2*	BXD60	1.76E-03	1.5	NS	NS
NM_007970	*Ezh1*	BXD60	2.53E-02	1.3	NS	NS
NM_010171	*F3*	BXD60	4.79E-02	1.5	3.92E-03	1.7
NM_023480	*Fahd1*	BXD60	4.25E-02	-1.6	NS	NS
NM_001163567	*Fam102b*	BXD60	3.95E-03	1.3	NS	NS
NM_001242423	*Fam105a*	BXD60	1.30E-02	1.7	NS	NS
NM_183187	*Fam107a*	BXD60	8.67E-07	3.6	1.03E-03	2.6
NM_001034851	*Fam134b*	BXD60	NS	NS	9.96E-03	1.4
NM_026501	*Fam134c*	BXD60	6.52E-03	1.8	NS	NS
NM_153574	*Fam13a*	BXD60	5.13E-04	2.0	NS	NS
NM_001033231	*Fam195b*	BXD60	4.72E-02	-1.5	NS	NS
NM_182808	*Fam19a1*	BXD60	1.64E-03	1.6	3.14E-02	1.5
NM_153782	*Fam20a*	BXD60	6.68E-03	1.8	NS	NS
NM_178908	*Fam26e*	BXD60	4.61E-06	2.3	1.32E-06	2.4
NM_001160378	*Fam46a*	BXD60	3.49E-02	1.6	NS	NS
NM_175104	*Fam53c*	BXD60	3.81E-03	1.8	6.77E-03	1.8
NM_177893	*Fan1*	BXD60	5.26E-04	2.0	NS	NS
NM_178797	*Far2*	BXD60	4.28E-04	-1.6	1.11E-02	-1.4
NM_007991	*Fbl*	BXD60	2.35E-02	-1.3	3.68E-02	-1.4
NM_001024142	*Fbxo10*	BXD60	2.49E-03	1.8	NS	NS
NM_001163702	*Fbxo27*	BXD60	1.17E-02	1.6	NS	NS
NM_134136	*Fbxo38*	BXD60	2.76E-02	1.4	NS	NS
NM_015791	*Fbxo8*	BXD60	2.02E-02	1.4	NS	NS
NM_010191	*Fdft1*	BXD60	2.64E-06	-1.5	2.04E-05	-1.5
NM_001253751	*Fdps*	BXD60	3.34E-02	-1.5	NS	NS
NM_015759	*Fgd3*	BXD60	1.52E-02	-1.7	NS	NS
NM_010197	*Fgf1*	BXD60	8.63E-03	1.9	NS	NS
NM_010201	*Fgf14*	BXD60	2.49E-02	1.5	NS	NS
NM_028263	*Fgfbp3*	BXD60	2.41E-02	-1.5	NS	NS
NM_001163215	*Fgfr3*	BXD60	1.44E-03	1.8	NS	NS
NM_001164259	*Fgfrl1*	BXD60	1.03E-02	-1.6	1.35E-02	-1.6
NM_008013	*Fgl2*	BXD60	6.83E-05	1.9	NS	NS
NM_153573	*Fkbp14*	BXD60	7.40E-03	1.7	NS	NS
NM_010219	*Fkbp4*	BXD60	4.10E-03	-1.4	NS	NS
NM_022009	*Flii*	BXD60	1.74E-02	-1.5	NS	NS
NM_001077698	*Fmnl1*	BXD60	NS	NS	4.26E-02	-1.7
NM_001038699	*Fn3k*	BXD60	NS	NS	3.14E-02	1.7
NM_181420	*Fn3krp*	BXD60	4.91E-03	1.6	NS	NS

NM_001159706	*Folh1*	BXD60	1.25E-02	1.9	NS	NS
NM_018789	*Foxo4*	BXD60	8.80E-03	1.9	NS	NS
NM_001081172	*Frmpd1*	BXD60	4.31E-02	1.5	NS	NS
NM_025799	*Fuca2*	BXD60	1.59E-02	1.4	NS	NS
NM_028058	*Fundc1*	BXD60	8.70E-03	1.5	NS	NS
NM_001012517	*Fut10*	BXD60	NS	NS	3.12E-02	1.9
NM_021458	*Fzd3*	BXD60	3.52E-02	1.3	NS	NS
NM_001162494	*Fzd6*	BXD60	3.82E-02	1.5	NS	NS
NM_008065	*Gabpa*	BXD60	1.30E-02	1.4	NS	NS
NM_010252	*Gabrg1*	BXD60	3.75E-02	1.5	NS	NS
NM_001160404	*Galnt1*	BXD60	2.41E-02	1.4	NS	NS
NM_001081421	*Galnt16*	BXD60	1.73E-04	1.7	NS	NS
NM_010256	*Gart*	BXD60	NS	NS	1.68E-02	-1.6
NM_030719	*Gatsl2*	BXD60	4.92E-03	1.6	NS	NS
NM_001161712	*Gcat*	BXD60	3.25E-02	-1.6	NS	NS
NM_182694	*Ggn*	BXD60	1.03E-02	-1.7	NS	NS
NM_010288	*Gja1*	BXD60	2.69E-02	1.4	NS	NS
ENSMUST00000108014	*Gm10985*	BXD60	2.43E-02	-2.0	NS	NS
NR_040366	*Gm15446*	BXD60	1.51E-02	1.4	NS	NS
ENSMUST00000180340	*Gm20926*	BXD60	3.87E-02	-1.6	NS	NS
ENSMUST00000177775	*Gm21095*	BXD60	NS	NS	1.12E-02	-2.6
ENSMUST00000179811	*Gm21760*	BXD60	NS	NS	1.40E-02	-2.9
ENSMUST00000178374	*Gm21874*	BXD60	2.57E-02	1.9	NS	NS
NM_001177715	*Gm2897*	BXD60	3.68E-02	-1.5	NS	NS
ENSMUST00000164069	*Gm3047*	BXD60	2.81E-02	-1.9	NS	NS
ENSMUST00000167882	*Gm3460*	BXD60	3.68E-02	-1.8	NS	NS
ENSMUST00000097935	*Gm6471*	BXD60	1.70E-02	-1.7	NS	NS
ENSMUST00000163738	*Gm8159*	BXD60	1.90E-02	-2.0	NS	NS
ENSMUST00000171636	*Gm8297*	BXD60	3.74E-02	-1.9	NS	NS
NM_177992	*Gmpr2*	BXD60	NS	NS	3.15E-02	1.5
NM_001190414	*Gne*	BXD60	NS	NS	2.02E-02	-1.5
NM_010317	*Gng4*	BXD60	4.19E-02	-1.4	NS	NS

NM_153547	*Gnl3*	BXD60	5.54E-03	-1.7	NS	NS
NM_001035122	*Golm1*	BXD60	6.84E-04	1.7	NS	NS
NM_008149	*Gpam*	BXD60	2.48E-04	1.5	2.72E-02	1.3
NM_001110809	*Gpatch4*	BXD60	4.50E-02	-1.5	NS	NS
NM_027518	*Gpr137c*	BXD60	8.32E-10	2.3	6.06E-09	2.3
NM_001190297	*Gpr155*	BXD60	2.76E-02	1.5	NS	NS
NM_177366	*Gpr157*	BXD60	NS	NS	4.39E-02	1.7
NM_001004761	*Gpr158*	BXD60	4.79E-02	1.6	NS	NS
NM_001167694	*Gpr19*	BXD60	2.36E-03	1.6	NS	NS
NM_010338	*Gpr37*	BXD60	5.12E-06	2.2	NS	NS
NM_175490	*Gpr75*	BXD60	4.99E-02	1.3	NS	NS
NM_145066	*Gpr85*	BXD60	5.60E-06	1.9	1.54E-03	1.6
NM_001195774	*Gprc5b*	BXD60	2.43E-02	1.5	NS	NS
NM_029522	*Gpsm2*	BXD60	5.54E-04	1.9	NS	NS
NM_173866	*Gpt2*	BXD60	1.77E-02	1.6	NS	NS
NM_172768	*Gramd1b*	BXD60	1.52E-02	1.4	NS	NS
NM_080289	*Grhpr*	BXD60	8.48E-03	1.7	NS	NS
NM_018869	*Grk5*	BXD60	1.55E-02	1.5	NS	NS
NM_001114333	*Grm1*	BXD60	1.34E-04	1.8	NS	NS
NM_008175	*Grn*	BXD60	2.23E-04	1.5	NS	NS
NM_026231	*Gstcd*	BXD60	NS	NS	3.12E-02	-1.9
NM_029555	*Gstk1*	BXD60	1.52E-04	2.0	NS	NS
NM_001160411	*Gstm4*	BXD60	1.67E-02	1.5	NS	NS
NM_026672	*Gstm7*	BXD60	1.98E-04	2.0	NS	NS
NM_016750	*H2afz*	BXD60	1.33E-02	1.3	NS	NS
NM_019975	*Hacl1*	BXD60	1.57E-02	1.6	NS	NS
NM_008216	*Has2*	BXD60	3.55E-02	-2.0	NS	NS
NM_008217	*Has3*	BXD60	2.49E-02	-1.9	NS	NS
NM_001081192	*Hcn4*	BXD60	1.95E-02	-1.5	NS	NS
NM_027382	*Hdac8*	BXD60	1.46E-02	1.5	4.93E-02	1.4
NM_001080549	*Hdx*	BXD60	1.11E-02	2.0	NS	NS
NM_144835	*Heatr1*	BXD60	3.73E-02	-1.5	1.37E-02	-1.7
NM_175256	*Heg1*	BXD60	9.01E-04	1.8	NS	NS
NM_022331	*Herpud1*	BXD60	1.83E-02	1.4	NS	NS
NM_145567	*Hibadh*	BXD60	2.48E-03	1.6	NS	NS
NM_001162950	*Hif3a*	BXD60	3.49E-03	2.2	1.40E-02	2.1
NM_010377	*Hist1h1t*	BXD60	2.35E-02	-1.7	NS	NS
NM_178213	*Hist2h2ab*	BXD60	6.32E-04	-1.9	NS	NS

NM_010657	*Hivep3*	BXD60	2.13E-03	1.7	NS	NS
NM_177338	*Hmbox1*	BXD60	4.53E-02	1.4	NS	NS
NM_025812	*Hmg20a*	BXD60	2.74E-02	1.4	NS	NS
NM_008255	*Hmgcr*	BXD60	4.89E-05	-1.8	3.34E-03	-1.6
NM_008256	*Hmgcs2*	BXD60	NS	NS	4.88E-02	1.9
NM_198937	*Hn1l*	BXD60	3.95E-03	-1.7	NS	NS
NM_013751	*Hrasls*	BXD60	3.30E-02	1.7	NS	NS
NM_053262	*Hsd17b11*	BXD60	3.52E-02	1.4	NS	NS
NM_010476	*Hsd17b7*	BXD60	1.17E-03	-1.6	3.15E-02	-1.4
NM_024255	*Hsdl2*	BXD60	3.14E-05	1.8	NS	NS
NM_001163434	*Hspa5*	BXD60	4.90E-02	1.3	NS	NS
NM_019564	*Htra1*	BXD60	2.17E-04	1.9	9.14E-03	1.7
NM_015790	*Icosl*	BXD60	3.63E-03	1.8	NS	NS
NM_010495	*Id1*	BXD60	3.26E-02	1.7	NS	NS
NM_031166	*Id4*	BXD60	3.08E-02	1.9	NS	NS
NM_145360	*Idi1*	BXD60	1.60E-07	-2.1	6.84E-08	-2.2
BC008167	*Ifi203*	BXD60	2.49E-02	1.8	NS	NS
NM_013562	*Ifrd1*	BXD60	NS	NS	5.68E-03	-1.7
NM_018854	*Ift20*	BXD60	1.54E-02	1.3	NS	NS
NM_010515	*Igf2r*	BXD60	1.02E-02	1.6	NS	NS
NM_001033323	*Igsf9b*	BXD60	5.08E-03	1.8	NS	NS
NM_010551	*Il16*	BXD60	3.75E-02	1.5	NS	NS
NM_026574	*Ino80*	BXD60	2.77E-03	-1.5	NS	NS
NM_001127363	*Inpp5a*	BXD60	2.04E-03	1.5	NS	NS
NM_008385	*Inpp5b*	BXD60	2.68E-02	1.6	NS	NS
NM_172439	*Inpp5j*	BXD60	7.36E-04	2.0	2.72E-03	1.9
NM_008916	*Inpp5k*	BXD60	1.53E-02	1.4	2.57E-02	1.4
NM_153526	*Insig1*	BXD60	1.46E-03	-1.8	9.98E-06	-2.2
NM_010568	*Insr*	BXD60	1.43E-02	1.3	NS	NS
NM_023579	*Ipo5*	BXD60	NS	NS	1.29E-02	-1.3
NM_001164598	*Irf2bp2*	BXD60	4.54E-02	1.3	NS	NS
NM_016851	*Irf6*	BXD60	1.92E-02	1.6	NS	NS
NM_177290	*Itgb8*	BXD60	5.07E-03	1.7	NS	NS
NM_010586	*Itpr2*	BXD60	1.13E-02	1.5	NS	NS
NM_001110275	*Itsn1*	BXD60	1.26E-02	1.3	NS	NS
NM_001205043	*Jarid2*	BXD60	4.69E-02	1.4	NS	NS
NM_010591	*Jun*	BXD60	3.46E-13	3.3	5.68E-14	3.4
NM_001286944	*Jund*	BXD60	6.95E-04	1.6	3.45E-02	1.5

NM_001038010	*Kat2a*	BXD60	2.77E-03	-1.4	NS	NS
NM_026370	*Kat8*	BXD60	2.67E-03	-1.7	NS	NS
NM_029116	*Kbtbd11*	BXD60	2.13E-02	1.5	NS	NS
NM_001039347	*Kcnd3*	BXD60	2.23E-04	-1.7	4.24E-03	-1.6
NM_001081134	*Kcng1*	BXD60	5.95E-03	-1.8	1.02E-02	-1.8
NM_029911	*Kcnk10*	BXD60	3.49E-04	1.8	2.71E-02	1.5
NM_001159850	*Kcnk2*	BXD60	3.68E-02	1.4	NS	NS
NM_026135	*Kctd16*	BXD60	4.28E-04	2.0	NS	NS
NM_172650	*Kctd3*	BXD60	3.49E-02	1.5	NS	NS
NM_026214	*Kctd4*	BXD60	1.59E-02	1.4	2.07E-02	1.4
NM_027008	*Kctd5*	BXD60	NS	NS	4.23E-02	1.4
NM_001033430	*Kdm7a*	BXD60	4.14E-02	1.6	NS	NS
NM_011317	*Khdrbs1*	BXD60	NS	NS	3.15E-02	-1.4
NM_010616	*Kif12*	BXD60	1.54E-02	-1.5	NS	NS
NM_001145779	*Kif2a*	BXD60	3.25E-02	-1.3	NS	NS
NM_001122733	*Kit*	BXD60	1.75E-02	-1.3	NS	NS
NM_001033298	*Kiz*	BXD60	6.06E-03	1.6	NS	NS
NM_078477	*Klf16*	BXD60	9.50E-05	-1.9	2.43E-06	-2.1
NM_011803	*Klf6*	BXD60	3.25E-02	-1.4	NS	NS
NM_010638	*Klf9*	BXD60	3.30E-03	1.5	NS	NS
NM_001033039	*Klhdc9*	BXD60	NS	NS	3.14E-02	2.0
NM_153128	*Klhl12*	BXD60	1.36E-03	1.8	NS	NS
NM_029436	*Klhl24*	BXD60	NS	NS	1.61E-03	1.5
NM_001195075	*Klhl3*	BXD60	3.49E-02	1.5	NS	NS
NM_001161800	*Klhl7*	BXD60	NS	NS	2.43E-02	1.5
NM_001177373	*Klk11*	BXD60	3.80E-02	1.6	NS	NS
NM_010655	*Kpna2*	BXD60	5.55E-04	-1.6	1.40E-02	-1.5
NM_008466	*Kpna3*	BXD60	1.62E-03	-1.3	NS	NS
NM_021284	*Kras*	BXD60	NS	NS	4.53E-02	-1.3
NM_178610	*Krr1*	BXD60	3.73E-03	-1.7	NS	NS
NM_010660	*Krt10*	BXD60	4.92E-02	-1.5	NS	NS
NM_010662	*Krt13*	BXD60	3.52E-02	-1.8	NS	NS
NM_026774	*Lamtor5*	BXD60	2.05E-02	1.3	NS	NS
NM_008640	*Laptm4a*	BXD60	4.33E-02	1.3	NS	NS
NM_138593	*Larp7*	BXD60	3.49E-02	-1.4	NS	NS
NM_010694	*Lcn3*	BXD60	2.43E-02	-1.7	NS	NS
NM_001247984	*Lcp1*	BXD60	NS	NS	3.96E-02	-1.5
NM_001077398	*Ldb2*	BXD60	1.43E-03	-1.7	NS	NS

NM_001252658	*Ldlr*	BXD60	3.98E-05	-2.4	1.36E-04	-2.4
NM_134093	*Letmd1*	BXD60	2.89E-02	1.3	NS	NS
NM_008494	*Lfng*	BXD60	5.13E-04	1.7	1.21E-02	1.5
NM_001199043	*Lgals8*	BXD60	6.75E-04	1.6	4.01E-02	1.4
NM_020278	*Lgi1*	BXD60	2.43E-02	1.4	NS	NS
NM_145219	*Lgi3*	BXD60	4.79E-02	1.6	NS	NS
NM_011175	*Lgmn*	BXD60	2.06E-02	1.3	NS	NS
NM_001083125	*Lhx6*	BXD60	3.70E-02	-1.7	NS	NS
NM_001001980	*Limch1*	BXD60	6.10E-04	1.8	NS	NS
NM_013860	*Limd1*	BXD60	9.30E-03	2.0	NS	NS
NM_144862	*Lims2*	BXD60	1.05E-02	2.0	NS	NS
NM_181074	*Lingo1*	BXD60	2.40E-03	-1.3	NS	NS
NM_201529	*Lmo7*	BXD60	7.31E-05	1.6	NS	NS
NM_028894	*Lonrf3*	BXD60	1.32E-02	1.8	NS	NS
NM_008377	*Lrig1*	BXD60	NS	NS	2.99E-02	-1.5
NM_008512	*Lrp1*	BXD60	2.79E-02	1.4	NS	NS
NM_001146048	*Lrrc1*	BXD60	1.63E-03	2.0	NS	NS
NM_001172064	*Lrrc56*	BXD60	8.34E-03	1.8	NS	NS
NM_133807	*Lrrc59*	BXD60	NS	NS	2.43E-02	-1.4
NM_178005	*Lrrtm2*	BXD60	NS	NS	3.46E-02	1.4
NM_181470	*Ltv1*	BXD60	3.49E-03	-1.8	NS	NS
NM_001252055	*Ly6c1*	BXD60	1.68E-04	2.7	NS	NS
NM_010748	*Lyst*	BXD60	2.18E-02	1.7	NS	NS
NM_172865	*Manea*	BXD60	1.40E-02	1.5	NS	NS
NM_001081230	*Map9*	BXD60	3.78E-02	-1.3	NS	NS
NM_001045483	*Mapk1ip1*	BXD60	2.36E-03	1.4	NS	NS
NM_011841	*Mapk7*	BXD60	3.44E-03	-1.4	3.68E-02	-1.4
NM_175171	*Mast4*	BXD60	7.28E-03	1.4	NS	NS
NM_175341	*Mbnl2*	BXD60	2.68E-02	1.4	NS	NS
NM_001167910	*Mbtps1*	BXD60	3.00E-02	1.4	NS	NS
NM_008563	*Mcm3*	BXD60	NS	NS	6.84E-03	-1.9
NM_008566	*Mcm5*	BXD60	2.27E-02	-1.8	NS	NS
NM_001288586	*Mdm2*	BXD60	1.65E-03	1.6	NS	NS
NM_027213	*Med6*	BXD60	1.74E-02	1.4	NS	NS
NM_001001979	*Megf10*	BXD60	7.63E-03	1.6	NS	NS
NM_001168488	*Men1*	BXD60	NS	NS	4.84E-02	-1.3
NM_008587	*Mertk*	BXD60	4.69E-04	2.4	3.00E-03	2.2
NM_008591	*Met*	BXD60	4.90E-02	-1.6	NS	NS

NM_175224	*Metap1*	BXD60	2.14E-02	-1.4	NS	NS
NM_027279	*Mettl18*	BXD60	1.43E-02	1.9	NS	NS
NM_024200	*Mfn1*	BXD60	4.67E-03	1.5	NS	NS
NM_029662	*Mfsd2a*	BXD60	3.98E-03	1.7	NS	NS
NM_173870	*Mgat4a*	BXD60	1.06E-03	1.7	NS	NS
NM_023799	*Mgea5*	BXD60	1.46E-03	1.4	1.20E-03	1.4
NM_001166249	*Mgll*	BXD60	1.32E-03	1.9	NS	NS
NM_030110	*Micu3*	BXD60	1.76E-03	1.4	NS	NS
NM_021565	*Midn*	BXD60	2.13E-03	-1.5	9.10E-03	-1.5
NM_001243584	*Mif4gd*	BXD60	8.48E-03	1.7	NS	NS
NM_021462	*Mknk2*	BXD60	8.02E-04	1.6	NS	NS
NM_175337	*Mlh3*	BXD60	2.28E-02	1.7	NS	NS
NM_175217	*Mmd2*	BXD60	8.20E-04	1.4	NS	NS
NM_008609	*Mmp15*	BXD60	1.74E-02	-1.5	2.82E-02	-1.5
NM_001081235	*Mn1*	BXD60	4.20E-02	-1.4	NS	NS
NM_001081287	*Mpp7*	BXD60	1.74E-02	1.8	NS	NS
NM_172610	*Mpped1*	BXD60	8.38E-03	-1.5	NS	NS
NM_025878	*Mrps18b*	BXD60	3.21E-02	-1.4	NS	NS
NM_025434	*Mrps28*	BXD60	2.00E-02	-1.6	NS	NS
NM_146222	*Msantd2*	BXD60	3.57E-04	-1.8	NS	NS
NM_010830	*Msh6*	BXD60	NS	NS	3.26E-02	-1.4
NM_025436	*Msmo1*	BXD60	8.86E-05	-2.0	5.11E-05	-2.1
NM_029619	*Msrb2*	BXD60	8.48E-03	1.8	NS	NS
NM_013602	*Mt1*	BXD60	2.75E-03	1.6	1.90E-02	1.5
NM_008630	*Mt2*	BXD60	2.61E-07	2.9	3.84E-05	2.4
NM_026788	*Mthfd2l*	BXD60	NS	NS	4.13E-02	-1.4
NM_198625	*Mtss1l*	BXD60	2.31E-02	-1.3	NS	NS
NM_001005863	*Mtus1*	BXD60	2.04E-03	1.7	NS	NS
NM_023431	*Mum1*	BXD60	NS	NS	1.84E-02	-1.5
NM_027877	*Mus81*	BXD60	4.13E-02	-1.6	NS	NS
NM_138656	*Mvd*	BXD60	8.15E-05	-2.1	1.20E-02	-1.7
NM_010753	*Mxd4*	BXD60	8.67E-04	1.8	NS	NS
NM_001008542	*Mxi1*	BXD60	1.51E-08	1.9	1.35E-04	1.6
NM_008651	*Mybl1*	BXD60	2.72E-02	1.7	NS	NS
NM_001177352	*Myc*	BXD60	2.29E-02	-1.5	NS	NS
NM_019472	*Myo10*	BXD60	1.63E-03	1.8	NS	NS
NM_001171615	*Myt1*	BXD60	3.94E-03	1.6	1.78E-02	1.6
NM_001040395	*Nadk2*	BXD60	6.82E-05	1.6	NS	NS

NM_001163564	*Naf1*	BXD60	1.64E-02	-1.6	NS	NS
NM_178728	*Napepld*	BXD60	8.81E-03	2.1	NS	NS
NM_026272	*Narf*	BXD60	1.30E-02	1.6	NS	NS
NM_001145959	*Ndrg2*	BXD60	7.56E-03	1.3	NS	NS
NM_022565	*Ndst4*	BXD60	NS	NS	5.18E-03	1.9
NM_010886	*Ndufa4*	BXD60	4.86E-02	1.2	NS	NS
NM_026742	*Ndufaf4*	BXD60	5.33E-04	-1.6	NS	NS
NM_001111324	*Nedd9*	BXD60	8.61E-03	1.6	NS	NS
NM_001113209	*Nfib*	BXD60	1.82E-02	1.3	NS	NS
NM_010911	*Nfs1*	BXD60	2.25E-02	1.6	NS	NS
NM_001048168	*Nfyc*	BXD60	NS	NS	4.23E-02	-1.4
NM_025449	*Nicn1*	BXD60	1.50E-03	1.3	NS	NS
NM_026472	*Nifk*	BXD60	2.15E-02	-1.3	NS	NS
NM_145469	*Nipal2*	BXD60	6.32E-04	1.7	NS	NS
NM_028995	*Nipal3*	BXD60	6.38E-03	1.7	NS	NS
NM_025937	*Nkap*	BXD60	4.79E-02	-1.7	NS	NS
NM_028186	*Nkd2*	BXD60	4.43E-02	1.6	NS	NS
NM_138314	*Nme7*	BXD60	1.02E-02	1.5	NS	NS
NM_145497	*Nmrk1*	BXD60	3.08E-04	2.6	NS	NS
NM_021303	*Noc2l*	BXD60	NS	NS	3.57E-02	-1.4
NM_009834	*Noct*	BXD60	3.56E-03	-1.6	NS	NS
NM_001001986	*Nol4l*	BXD60	4.79E-02	-1.4	9.14E-03	-1.5
NM_138747	*Nop2*	BXD60	NS	NS	4.24E-03	-1.5
NM_024193	*Nop56*	BXD60	5.38E-03	-1.6	1.63E-02	-1.6
NM_018868	*Nop58*	BXD60	4.54E-02	-1.3	NS	NS
NM_021361	*Nova1*	BXD60	2.21E-02	1.5	NS	NS
NM_008719	*Npas2*	BXD60	4.79E-02	-1.8	NS	NS
NM_013780	*Npas3*	BXD60	NS	NS	2.78E-02	-1.5
NM_010342	*Npbwr1*	BXD60	1.02E-02	1.7	NS	NS
NM_018787	*Npff*	BXD60	4.78E-02	1.3	NS	NS
NM_008723	*Npm3*	BXD60	NS	NS	1.18E-02	-1.7
NM_010934	*Npy1r*	BXD60	4.31E-02	-1.4	NS	NS
NM_001139509	*Nr4a2*	BXD60	4.25E-02	-2.2	NS	NS
NM_001164363	*Nt5c2*	BXD60	1.63E-03	1.4	NS	NS
NM_153136	*Nudt18*	BXD60	1.17E-02	1.6	NS	NS
NM_008751	*Nxph1*	BXD60	NS	NS	3.82E-02	-1.4
NM_001161430	*Nxt2*	BXD60	8.21E-04	1.5	NS	NS
NM_178644	*Oaf*	BXD60	2.40E-03	2.2	4.84E-02	1.9

NM_013614	*Odc1*	BXD60	4.16E-03	1.7	NS	NS
NM_198673	*Odf3l1*	BXD60	3.57E-04	-1.7	NS	NS
NM_146571	*Olfr1015*	BXD60	1.35E-02	-2.0	NS	NS
NM_146323	*Olfr1232*	BXD60	2.91E-03	-1.9	NS	NS
NM_001011853	*Olfr1425*	BXD60	1.54E-02	-2.1	NS	NS
NM_147036	*Olfr283*	BXD60	3.45E-02	-1.5	NS	NS
NM_146306	*Olfr518*	BXD60	4.25E-02	-1.4	NS	NS
NM_146361	*Olfr557*	BXD60	NS	NS	3.15E-02	1.7
NM_146822	*Olfr640*	BXD60	3.30E-02	-1.9	NS	NS
NM_013621	*Olfr69*	BXD60	2.64E-02	-1.9	NS	NS
NM_146864	*Olfr768*	BXD60	4.92E-02	-1.7	NS	NS
NM_152818	*Osbp2*	BXD60	2.73E-02	1.3	NS	NS
NM_001134791	*Osbpl9*	BXD60	7.74E-06	1.5	8.74E-04	1.4
NM_172416	*Ostm1*	BXD60	4.81E-03	1.6	NS	NS
NM_130880	*Otud7a*	BXD60	NS	NS	3.08E-02	1.4
NM_145460	*Oxnad1*	BXD60	4.36E-02	1.3	NS	NS
NM_001008497	*P2ry14*	BXD60	2.81E-02	1.9	NS	NS
NM_011119	*Pa2g4*	BXD60	3.65E-02	-1.3	NS	NS
NM_026420	*Paip2*	BXD60	1.73E-04	1.4	3.71E-02	1.3
NM_146169	*Paip2b*	BXD60	3.67E-02	1.4	NS	NS
NM_021362	*Pappa*	BXD60	1.66E-02	1.9	NS	NS
NM_028829	*Paqr8*	BXD60	9.82E-03	1.4	1.27E-02	1.5
NM_001253690	*Patz1*	BXD60	8.11E-03	1.6	NS	NS
NM_011865	*Pcbp1*	BXD60	8.14E-06	-1.7	NS	NS
NM_053133	*Pcdhb8*	BXD60	4.31E-02	-1.6	NS	NS
NM_146129	*Pcif1*	BXD60	NS	NS	1.61E-02	-1.6
NM_183028	*Pcmtd1*	BXD60	NS	NS	3.38E-02	1.3
NM_153594	*Pcmtd2*	BXD60	1.14E-04	2.0	NS	NS
NM_025823	*Pcyox1*	BXD60	3.07E-02	1.4	NS	NS
NM_001177980	*Pde4b*	BXD60	2.16E-05	1.6	1.14E-02	1.4
NM_001170669	*Pde8b*	BXD60	1.95E-02	1.7	NS	NS
NM_008808	*Pdgfa*	BXD60	9.02E-03	-1.5	3.92E-03	-1.5
NM_027924	*Pdgfd*	BXD60	3.99E-02	1.9	NS	NS
NM_008810	*Pdha1*	BXD60	1.67E-02	1.5	NS	NS
NM_001033222	*Pdzd8*	BXD60	3.54E-02	-1.3	NS	NS
NM_023523	*Pecr*	BXD60	1.49E-02	1.6	NS	NS
NM_001159367	*Per1*	BXD60	1.68E-03	1.7	NS	NS
NM_172417	*Perm1*	BXD60	3.08E-02	-1.8	NS	NS

NM_011072	*Pfn1*	BXD60	2.01E-02	-1.4	NS	NS
NM_025700	*Pgm1*	BXD60	4.76E-02	-1.5	NS	NS
NM_009343	*Phf1*	BXD60	NS	NS	1.40E-02	-1.6
NM_024250	*Phf10*	BXD60	3.12E-03	-1.4	NS	NS
NM_174852	*Phf12*	BXD60	2.52E-03	-1.6	NS	NS
NM_172705	*Phf13*	BXD60	NS	NS	3.68E-02	-1.7
NM_001109690	*Phf21a*	BXD60	4.45E-02	1.5	NS	NS
NM_030064	*Phf23*	BXD60	NS	NS	2.34E-02	-1.5
NM_016966	*Phgdh*	BXD60	9.03E-05	1.8	NS	NS
NM_053191	*Pi15*	BXD60	NS	NS	2.33E-02	1.9
NM_001145926	*Pianp*	BXD60	3.48E-02	-1.5	NS	NS
NM_001003948	*Pid1*	BXD60	1.73E-02	1.5	NS	NS
NM_001145955	*Pigv*	BXD60	3.00E-02	1.5	NS	NS
NM_178149	*Pik3ip1*	BXD60	2.48E-03	1.8	3.03E-03	1.9
NM_001024955	*Pik3r1*	BXD60	1.11E-12	2.0	1.74E-09	1.8
NM_028228	*Pinx1*	BXD60	4.67E-03	-1.7	NS	NS
NM_177298	*Pisd*	BXD60	4.12E-02	1.6	NS	NS
NM_145823	*Pitpnc1*	BXD60	4.11E-02	1.2	NS	NS
NM_026163	*Pkp2*	BXD60	9.35E-03	1.9	NS	NS
NM_172695	*Plaa*	BXD60	4.36E-02	-1.2	NS	NS
NM_008872	*Plat*	BXD60	1.59E-02	1.6	4.53E-02	1.6
NM_152813	*Plcd3*	BXD60	1.80E-03	1.8	NS	NS
NM_001081456	*Plcd4*	BXD60	1.97E-02	1.6	NS	NS
NM_008876	*Pld2*	BXD60	2.75E-02	1.4	NS	NS
NM_133942	*Plekha1*	BXD60	1.81E-02	1.4	NS	NS
NM_001033253	*Plekhg1*	BXD60	1.93E-08	2.5	2.53E-03	1.8
NM_181073	*Plekhh1*	BXD60	3.55E-02	1.7	NS	NS
NM_013807	*Plk3*	BXD60	1.33E-06	-2.2	1.79E-03	-1.8
NM_026925	*Pnlip*	BXD60	2.18E-02	-1.5	NS	NS
NM_175498	*Pnma2*	BXD60	NS	NS	4.39E-02	-1.5
NM_008891	*Pnn*	BXD60	4.57E-02	-1.4	NS	NS
NM_025443	*Pno1*	BXD60	1.95E-02	-1.5	NS	NS
NM_026383	*Pnrc2*	BXD60	3.41E-04	1.6	NS	NS
NM_029037	*Pomk*	BXD60	1.95E-02	1.5	NS	NS
NM_133931	*Pot1a*	BXD60	3.73E-02	1.4	NS	NS
NM_028370	*Pot1b*	BXD60	2.99E-02	1.7	NS	NS
NM_008900	*Pou3f3*	BXD60	4.67E-03	-1.5	NS	NS
NM_133249	*Ppargc1b*	BXD60	NS	NS	2.30E-02	-1.7

NM_008014	*Ppm1g*	BXD60	2.51E-02	-1.3	NS	NS
NM_027242	*Ppp1r35*	BXD60	NS	NS	1.28E-02	-1.7
NM_001171004	*Prkd3*	BXD60	2.93E-03	1.6	NS	NS
NM_001252476	*Prmt1*	BXD60	NS	NS	1.72E-02	-1.5
NM_001081240	*Prmt10*	BXD60	3.75E-02	1.4	NS	NS
NM_144944	*Prokr2*	BXD60	1.72E-02	-1.7	NS	NS
NM_018786	*Prpf40b*	BXD60	2.99E-02	-1.6	NS	NS
NM_133701	*Prpf6*	BXD60	1.63E-02	1.4	NS	NS
NM_175022	*Prr12*	BXD60	3.08E-02	-1.5	NS	NS
NM_146061	*Prr5*	BXD60	1.44E-02	1.5	NS	NS
NM_177698	*Psd3*	BXD60	1.53E-03	1.4	NS	NS
NM_008945	*Psmb4*	BXD60	4.99E-02	1.3	NS	NS
NM_001282017	*Psmd4*	BXD60	3.43E-02	-1.4	NS	NS
NM_134013	*Psme4*	BXD60	2.14E-02	1.3	NS	NS
NM_025682	*Pspc1*	BXD60	2.15E-04	-1.8	NS	NS
NM_207232	*Ptpdc1*	BXD60	5.54E-03	1.8	NS	NS
NM_011201	*Ptpn1*	BXD60	2.43E-02	-1.3	NS	NS
NM_019933	*Ptpn4*	BXD60	3.63E-03	1.7	NS	NS
NM_025831	*Pxdc1*	BXD60	1.80E-05	2.0	NS	NS
NM_153781	*Pygb*	BXD60	4.99E-02	1.3	NS	NS
NM_001168270	*Qars*	BXD60	3.30E-02	1.4	NS	NS
NM_001024945	*Qsox1*	BXD60	9.77E-05	1.8	NS	NS
NM_001146012	*R3hcc1*	BXD60	NS	NS	4.13E-02	-1.5
NM_017382	*Rab11a*	BXD60	4.70E-02	-1.3	NS	NS
NM_001033172	*Rab11fip2*	BXD60	1.74E-02	1.3	NS	NS
NM_023635	*Rab27a*	BXD60	3.82E-02	1.4	NS	NS
NM_029494	*Rab30*	BXD60	2.56E-12	2.5	6.06E-09	2.2
NM_175122	*Rab39b*	BXD60	3.31E-03	1.3	NS	NS
NM_001038621	*Rabgap1l*	BXD60	9.59E-06	1.5	7.46E-04	1.4
NM_001277938	*Rad51d*	BXD60	1.33E-02	1.8	NS	NS
NM_019491	*Rala*	BXD60	3.32E-03	-1.6	NS	NS
NM_011239	*Ranbp1*	BXD60	2.51E-02	-1.5	NS	NS
NM_001099624	*Rapgef2*	BXD60	1.26E-10	1.9	1.75E-09	1.9
NM_001252494	*Rapgef6*	BXD60	1.09E-08	2.3	6.66E-08	2.3
NM_001176528	*Rara*	BXD60	1.53E-03	-1.6	5.68E-03	-1.6
NM_009025	*Rasa3*	BXD60	1.33E-02	-1.5	1.29E-02	-1.6
NM_177644	*Rasal2*	BXD60	1.33E-02	1.5	NS	NS
NM_178045	*Rassf4*	BXD60	NS	NS	2.04E-02	1.7

NM_015754	*Rbbp9*	BXD60	3.75E-02	1.4	NS	NS
NM_021477	*Rbfox1*	BXD60	2.05E-02	1.3	NS	NS
NM_001110827	*Rbfox2*	BXD60	1.40E-02	1.3	2.94E-02	1.3
NM_028234	*Rbm33*	BXD60	6.95E-04	1.5	1.12E-03	1.6
NM_001290123	*Rbm4*	BXD60	2.80E-02	1.5	NS	NS
NM_153405	*Rbm45*	BXD60	4.79E-02	-1.4	3.75E-02	-1.5
NM_001024952	*Rc3h1*	BXD60	4.31E-02	1.3	NS	NS
NM_001100591	*Rc3h2*	BXD60	2.10E-02	1.3	NS	NS
NM_001081549	*Rcan1*	BXD60	5.72E-06	1.7	NS	NS
NM_027764	*Rcbtb1*	BXD60	3.82E-02	1.5	NS	NS
NM_030017	*Rdh12*	BXD60	2.07E-02	1.7	NS	NS
NM_001204931	*Reep6*	BXD60	NS	NS	1.39E-02	-1.7
NM_011259	*Reg3a*	BXD60	3.14E-02	-1.6	NS	NS
NM_028713	*Rftn2*	BXD60	2.58E-02	1.5	NS	NS
NM_016846	*Rgl1*	BXD60	1.52E-02	1.4	NS	NS
NM_009062	*Rgs4*	BXD60	NS	NS	2.34E-02	1.5
NM_001282061	*Rgs6*	BXD60	6.76E-03	1.6	NS	NS
NM_001199003	*Rgs7*	BXD60	4.40E-02	-1.3	NS	NS
NM_007483	*Rhob*	BXD60	1.78E-02	1.4	NS	NS
NM_153514	*Rhobtb2*	BXD60	NS	NS	5.00E-02	-1.4
NM_011882	*Rnasel*	BXD60	4.50E-02	1.5	NS	NS
NM_172612	*Rnd1*	BXD60	4.79E-02	-1.5	3.60E-02	-1.5
NM_009543	*Rnf103*	BXD60	3.30E-02	1.3	NS	NS
NM_144528	*Rnf126*	BXD60	1.67E-02	-1.3	NS	NS
NM_001170643	*Rnf144b*	BXD60	3.42E-02	1.7	NS	NS
NM_001166553	*Rnf145*	BXD60	1.89E-04	-1.5	1.05E-05	-1.7
NM_001033135	*Rnf149*	BXD60	1.51E-02	1.9	NS	NS
NM_194346	*Rnf31*	BXD60	4.79E-02	-1.5	NS	NS
NM_001164767	*Robo3*	BXD60	2.01E-02	-1.6	NS	NS
NM_146246	*Rp1l1*	BXD60	1.95E-02	-1.6	NS	NS
NM_133982	*Rpp25*	BXD60	1.97E-02	-1.6	NS	NS
NM_001033212	*Rprml*	BXD60	9.82E-03	-1.7	NS	NS
NM_146244	*Rps6kl1*	BXD60	8.94E-06	2.1	2.43E-02	1.6
NM_027491	*Rragd*	BXD60	8.48E-03	1.4	NS	NS
NM_001005525	*Rsrc2*	BXD60	7.44E-03	-1.4	NS	NS
NM_145422	*Rtcb*	BXD60	2.12E-02	1.5	NS	NS
NM_001111026	*Runx1t1*	BXD60	4.76E-02	-1.4	NS	NS
NM_001042607	*Ryk*	BXD60	1.39E-02	1.3	NS	NS

NM_007901	*S1pr1*	BXD60	2.72E-02	1.6	NS	NS
NM_026283	*Samd8*	BXD60	1.20E-04	1.5	NS	NS
NM_175155	*Sash1*	BXD60	2.13E-02	1.5	NS	NS
NM_009129	*Scg2*	BXD60	3.73E-03	1.4	NS	NS
NM_011324	*Scnn1a*	BXD60	6.30E-03	2.1	NS	NS
NM_001271472	*Scube1*	BXD60	3.33E-02	-1.6	NS	NS
NM_011521	*Sdc4*	BXD60	2.14E-04	2.4	3.39E-02	1.8
NM_019951	*Sec11a*	BXD60	9.91E-03	1.4	NS	NS
NM_146013	*Sec14l4*	BXD60	6.26E-03	2.0	NS	NS
NM_009154	*Sema5a*	BXD60	2.40E-02	1.5	NS	NS
NM_175400	*Sephs1*	BXD60	8.38E-03	1.7	NS	NS
NM_001253386	*Serinc2*	BXD60	2.68E-02	1.6	NS	NS
NM_172588	*Serinc5*	BXD60	5.31E-05	1.8	NS	NS
NM_001013370	*Sesn1*	BXD60	2.05E-03	1.6	NS	NS
NM_030261	*Sesn3*	BXD60	1.18E-04	1.6	3.00E-03	1.5
NM_001204875	*Set*	BXD60	2.43E-02	-1.3	NS	NS
NM_001040398	*Setd1b*	BXD60	NS	NS	3.19E-03	-1.7
NM_080793	*Setd7*	BXD60	3.70E-02	1.6	NS	NS
NM_031179	*Sf3b1*	BXD60	1.96E-02	-1.3	NS	NS
NM_026377	*Sfr1*	BXD60	1.29E-02	-1.3	NS	NS
NM_016687	*Sfrp4*	BXD60	1.74E-02	2.0	NS	NS
NM_145512	*Sft2d2*	BXD60	2.81E-02	1.6	NS	NS
NM_001168525	*Sgms1*	BXD60	4.99E-03	2.0	NS	NS
NM_001282037	*Sh3glb1*	BXD60	3.48E-02	-1.2	NS	NS
NM_001168505	*Shoc2*	BXD60	4.87E-02	-1.2	NS	NS
NM_001289668	*Sidt2*	BXD60	8.34E-03	1.6	NS	NS
NM_001286538	*Sigmar1*	BXD60	NS	NS	3.92E-03	-1.5
NM_010831	*Sik1*	BXD60	4.96E-02	-1.4	NS	NS
NM_001110350	*Sin3a*	BXD60	NS	NS	4.24E-03	-1.5
NM_001167983	*Sipa1l1*	BXD60	NS	NS	2.18E-02	1.4
NM_029612	*Slamf9*	BXD60	NS	NS	7.80E-03	2.2
NM_001146161	*Slc11a2*	BXD60	3.23E-02	-1.5	NS	NS
NM_023044	*Slc15a3*	BXD60	1.64E-02	1.8	NS	NS
NM_001114332	*Slc16a10*	BXD60	4.31E-02	1.6	NS	NS
NM_027921	*Slc16a14*	BXD60	2.33E-03	1.6	1.30E-02	1.5
NM_001029842	*Slc16a6*	BXD60	4.96E-02	1.7	2.04E-02	2.0
NM_001199271	*Slc19a1*	BXD60	1.23E-02	1.5	NS	NS
NM_001159593	*Slc20a1*	BXD60	6.85E-03	-1.4	NS	NS

NM_001110240	*Slc24a2*	BXD60	1.67E-02	1.6	NS	NS
NM_172152	*Slc24a4*	BXD60	2.76E-02	1.6	NS	NS
NM_138756	*Slc25a36*	BXD60	6.68E-03	1.4	NS	NS
NM_011977	*Slc27a1*	BXD60	2.05E-03	1.5	NS	NS
NM_007854	*Slc29a2*	BXD60	NS	NS	4.03E-02	-1.7
NM_011400	*Slc2a1*	BXD60	9.59E-04	1.8	1.67E-02	1.7
NM_011401	*Slc2a3*	BXD60	3.00E-02	1.4	NS	NS
NM_019741	*Slc2a5*	BXD60	NS	NS	5.68E-03	1.9
NM_021435	*Slc35b4*	BXD60	2.05E-04	1.6	NS	NS
NM_028787	*Slc35f5*	BXD60	2.05E-02	1.3	NS	NS
NM_001164798	*Slc38a10*	BXD60	4.76E-02	1.3	NS	NS
NM_175121	*Slc38a2*	BXD60	3.79E-02	1.3	NS	NS
NM_134135	*Slc39a3*	BXD60	3.86E-02	1.5	NS	NS
NM_177388	*Slc41a2*	BXD60	3.25E-02	1.4	NS	NS
NM_001199283	*Slc43a2*	BXD60	7.40E-04	1.7	NS	NS
NM_021398	*Slc43a3*	BXD60	7.84E-03	1.7	NS	NS
NM_001199186	*Slc44a2*	BXD60	1.07E-02	1.5	NS	NS
NM_001081263	*Slc44a5*	BXD60	3.20E-07	2.0	2.70E-05	1.8
NM_172890	*Slc6a11*	BXD60	1.10E-02	1.3	NS	NS
NM_012030	*Slc9a3r1*	BXD60	4.33E-02	-1.4	NS	NS
NM_001038643	*Slco3a1*	BXD60	4.90E-02	-1.3	1.03E-02	-1.5
NM_172841	*Slco5a1*	BXD60	2.06E-02	1.6	NS	NS
NM_001252481	*Smad2*	BXD60	2.82E-03	-1.6	1.68E-02	-1.5
NM_008540	*Smad4*	BXD60	8.05E-03	1.3	NS	NS
NM_031842	*Smarcd1*	BXD60	1.64E-03	-1.4	NS	NS
NM_001031814	*Smg1*	BXD60	1.59E-02	1.6	NS	NS
NR_028086	*Smim10l2a*	BXD60	3.68E-02	1.7	NS	NS
NM_133697	*Smim14*	BXD60	3.78E-02	1.2	3.15E-02	1.3
NM_134133	*Smim3*	BXD60	NS	NS	1.68E-05	3.0
NM_001252629	*Smn1*	BXD60	2.68E-02	-1.5	NS	NS
NM_001177833	*Smox*	BXD60	1.36E-02	1.5	NS	NS
NM_183316	*Snapc5*	BXD60	4.99E-02	1.4	NS	NS
NM_001199151	*Sncaip*	BXD60	3.14E-02	1.4	NS	NS
NM_027671	*Sntg1*	BXD60	2.70E-02	1.5	NS	NS
NM_175483	*Snx33*	BXD60	3.62E-02	1.7	NS	NS
NM_183173	*Sowaha*	BXD60	NS	NS	1.63E-02	1.7
NM_001025428	*Spag9*	BXD60	2.64E-02	1.3	9.96E-03	1.4
NM_170756	*Spata2*	BXD60	4.36E-02	1.5	NS	NS

NM_001164566	*Spats2l*	BXD60	9.78E-03	-1.5	NS	NS
NM_172430	*Sphkap*	BXD60	3.49E-03	1.5	NS	NS
NM_023220	*Sppl2a*	BXD60	1.95E-02	1.4	NS	NS
NM_182927	*Spred3*	BXD60	1.70E-03	-2.0	5.93E-03	-2.0
NM_011898	*Spry4*	BXD60	1.74E-04	-2.1	3.91E-06	-2.5
NM_009270	*Sqle*	BXD60	9.36E-04	-1.6	3.84E-02	-1.4
NM_020493	*Srf*	BXD60	NS	NS	4.01E-02	-1.6
NM_080448	*Srgap3*	BXD60	3.09E-02	1.3	NS	NS
NM_009272	*Srm*	BXD60	2.79E-03	-1.4	1.46E-02	-1.4
NM_001109909	*Srrt*	BXD60	NS	NS	1.54E-02	-1.5
NM_011358	*Srsf2*	BXD60	9.03E-05	-1.6	1.54E-02	-1.4
NM_013663	*Srsf3*	BXD60	4.09E-02	-1.4	NS	NS
NM_001195485	*Srsf7*	BXD60	7.18E-04	-1.8	4.64E-04	-1.9
NM_178750	*Ss18l1*	BXD60	8.63E-03	-1.4	NS	NS
NM_001110145	*Ssb*	BXD60	4.67E-02	-1.3	NS	NS
NM_026155	*Ssr3*	BXD60	1.64E-02	1.3	NS	NS
NM_012028	*St6galnac5*	BXD60	1.50E-03	-1.4	NS	NS
NM_011374	*St8sia1*	BXD60	8.05E-03	1.4	6.59E-04	1.6
NM_019990	*Stard10*	BXD60	3.80E-02	1.5	NS	NS
NM_133774	*Stard4*	BXD60	4.61E-06	-2.2	1.12E-02	-1.7
NM_001113563	*Stat5b*	BXD60	1.81E-02	1.5	NS	NS
NM_009287	*Stim1*	BXD60	2.43E-02	1.6	NS	NS
NM_013515	*Stom*	BXD60	1.95E-02	1.9	NS	NS
NM_175367	*Ston2*	BXD60	8.80E-03	1.5	NS	NS
NM_153563	*Strip1*	BXD60	NS	NS	2.34E-02	-1.3
NM_001102423	*Stx16*	BXD60	3.98E-03	1.5	NS	NS
NM_011505	*Stxbp4*	BXD60	1.32E-02	1.6	NS	NS
NM_001114611	*Stxbp5l*	BXD60	2.84E-03	1.5	NS	NS
NM_019995	*Supt20*	BXD60	1.74E-02	-1.5	NS	NS
NM_009296	*Supt4a*	BXD60	2.81E-02	1.4	NS	NS
NM_144796	*Susd4*	BXD60	2.95E-02	1.4	NS	NS
NM_001242419	*Susd6*	BXD60	2.72E-02	1.5	NS	NS
NM_027832	*Sval1*	BXD60	1.51E-02	-2.5	NS	NS
NM_013679	*Svs6*	BXD60	NS	NS	4.12E-02	2.0
NM_001164495	*Syn3*	BXD60	NS	NS	1.84E-02	1.4
NM_153399	*Syne1*	BXD60	3.49E-02	1.4	1.22E-03	1.7
NM_001005510	*Syne2*	BXD60	1.62E-02	1.5	NS	NS
NM_194341	*Synrg*	BXD60	2.81E-02	1.5	NS	NS

NM_001040085	*Sytl2*	BXD60	9.95E-04	2.0	2.09E-04	2.2
NM_001161730	*Tap1*	BXD60	4.76E-02	1.8	NS	NS
NM_029745	*Tbc1d9b*	BXD60	4.96E-02	1.4	NS	NS
NM_011553	*Tcp10a*	BXD60	3.30E-02	-1.7	NS	NS
NM_146008	*Tcp11l2*	BXD60	1.87E-02	1.6	NS	NS
NM_028354	*Tdp1*	BXD60	2.96E-02	1.3	NS	NS
NM_026099	*Tex43*	BXD60	4.58E-02	-1.5	NS	NS
NM_001184706	*Tfdp2*	BXD60	9.43E-03	1.6	NS	NS
NM_001289550	*Tgfb1i1*	BXD60	2.62E-02	-1.6	NS	NS
NM_009368	*Tgfb3*	BXD60	3.78E-02	1.9	NS	NS
NM_009381	*Thrsp*	BXD60	9.75E-05	2.1	3.92E-03	1.9
NM_174989	*Ticam1*	BXD60	7.35E-04	2.2	NS	NS
NM_001081145	*Tigd2*	BXD60	5.08E-03	1.5	1.23E-02	1.5
NM_001136082	*Timeless*	BXD60	4.87E-02	-1.6	NS	NS
NM_172664	*Tlk1*	BXD60	4.25E-02	-1.3	NS	NS
NM_133352	*Tm9sf3*	BXD60	2.35E-02	1.3	NS	NS
NM_172476	*Tmc7*	BXD60	2.38E-02	1.4	NS	NS
NM_177412	*Tmcc1*	BXD60	8.94E-03	1.4	9.87E-03	1.4
NM_025360	*Tmed3*	BXD60	4.25E-02	1.7	NS	NS
NM_134142	*Tmem109*	BXD60	4.89E-02	-1.8	NS	NS
NM_027495	*Tmem144*	BXD60	1.59E-02	1.6	NS	NS
NM_175106	*Tmem177*	BXD60	NS	NS	4.76E-02	1.7
NM_001253817	*Tmem184b*	BXD60	2.93E-02	1.4	NS	NS
NM_001113211	*Tmem194*	BXD60	3.30E-02	1.6	NS	NS
NM_001160385	*Tmem196*	BXD60	3.44E-04	2.0	NS	NS
NM_177056	*Tmem198*	BXD60	9.61E-03	1.6	NS	NS
NM_144525	*Tmem214*	BXD60	2.35E-02	-1.4	NS	NS
NM_025667	*Tmem222*	BXD60	5.95E-03	1.5	NS	NS
NM_001033321	*Tmem231*	BXD60	7.79E-03	1.6	NS	NS
NM_027935	*Tmem50a*	BXD60	3.68E-02	1.3	NS	NS
NM_001081186	*Tmem52b*	BXD60	4.82E-02	-1.5	NS	NS
NM_021793	*Tmem8*	BXD60	3.83E-03	1.9	NS	NS
NM_133706	*Tmem97*	BXD60	NS	NS	5.79E-03	-1.8
NM_011607	*Tnc*	BXD60	1.47E-02	-1.5	NS	NS
NM_001161746	*Tnfrsf12a*	BXD60	NS	NS	5.68E-03	1.9
NM_001048267	*Tnpo1*	BXD60	5.60E-06	1.7	3.45E-02	1.4
NM_144812	*Tnrc6b*	BXD60	6.06E-03	1.5	4.03E-02	1.5
NM_001109748	*Tomm40*	BXD60	NS	NS	2.22E-02	-1.4

NM_001160180	*Tor1aip2*	BXD60	3.79E-02	1.3	NS	NS
NM_001170488	*Tprkb*	BXD60	5.03E-03	1.6	4.19E-02	1.5
NM_198102	*Tra2a*	BXD60	4.20E-02	-1.4	NS	NS
NM_009186	*Tra2b*	BXD60	4.25E-02	-1.3	NS	NS
NM_001163470	*Trafd1*	BXD60	NS	NS	4.81E-02	1.3
NM_001014398	*Trcg1*	BXD60	6.95E-04	-1.8	NS	NS
NM_009054	*Trim27*	BXD60	4.89E-02	-1.4	NS	NS
NM_178110	*Trim62*	BXD60	2.77E-03	-1.6	4.26E-02	-1.5
NM_001127233	*Trp53*	BXD60	NS	NS	1.65E-02	-1.5
NM_173378	*Trp53bp2*	BXD60	3.84E-02	1.5	NS	NS
NM_001199105	*Trp53inp1*	BXD60	2.48E-04	2.2	NS	NS
NM_011643	*Trpc1*	BXD60	1.89E-02	1.4	NS	NS
NM_032000	*Trps1*	BXD60	5.88E-03	1.5	NS	NS
NM_001289575	*Tsc1*	BXD60	9.50E-03	1.6	NS	NS
NM_001077364	*Tsc22d3*	BXD60	2.35E-02	1.6	NS	NS
NM_016909	*Tsnax*	BXD60	3.88E-02	-1.3	3.18E-02	-1.3
NM_173007	*Tspan12*	BXD60	3.52E-02	1.3	NS	NS
NM_197996	*Tspan15*	BXD60	2.99E-03	1.7	NS	NS
NM_019793	*Tspan3*	BXD60	2.79E-02	1.3	NS	NS
NM_025982	*Tspan31*	BXD60	4.82E-03	1.3	NS	NS
NM_146173	*Tspan33*	BXD60	2.76E-02	1.8	NS	NS
NM_019634	*Tspan7*	BXD60	2.32E-02	1.2	NS	NS
NM_145607	*Ttc13*	BXD60	3.91E-02	-1.3	NS	NS
NM_028341	*Ttc39c*	BXD60	4.58E-02	1.6	NS	NS
NM_021885	*Tub*	BXD60	3.29E-02	1.3	NS	NS
NM_146116	*Tubb4b*	BXD60	NS	NS	3.96E-03	-1.5
NM_001009935	*Txnip*	BXD60	2.11E-05	3.3	1.88E-04	3.1
NM_001015876	*Tyw1*	BXD60	6.26E-03	1.7	NS	NS
NM_133806	*Uap1*	BXD60	2.08E-02	-1.3	NS	NS
NM_176860	*Ubash3b*	BXD60	5.03E-03	-1.7	NS	NS
NM_145420	*Ube2d1*	BXD60	4.34E-02	-1.2	NS	NS
NM_001169576	*Ube2h*	BXD60	1.59E-02	1.4	1.68E-05	1.7
NM_011908	*Ubl3*	BXD60	3.08E-02	1.3	NS	NS
NM_001083319	*Ubp1*	BXD60	NS	NS	4.83E-02	-1.3
NM_001177374	*Ubr2*	BXD60	3.65E-02	1.4	NS	NS
NM_030724	*Uck2*	BXD60	2.49E-03	-1.4	3.46E-02	-1.3
NM_026194	*Ufl1*	BXD60	4.66E-02	1.3	NS	NS
NM_013881	*Ulk2*	BXD60	1.25E-02	1.3	NS	NS

NM_175309	*Upk3b*	BXD60	4.65E-02	-1.5	NS	NS
NM_009462	*Usp10*	BXD60	NS	NS	2.59E-02	-1.3
NM_144937	*Usp3*	BXD60	3.25E-02	1.4	NS	NS
NM_152825	*Usp45*	BXD60	4.92E-02	1.5	NS	NS
NM_030180	*Usp54*	BXD60	4.18E-04	1.9	NS	NS
NM_001033236	*Vcpkmt*	BXD60	1.57E-02	1.5	NS	NS
NM_001025250	*Vegfa*	BXD60	1.76E-03	-1.8	NS	NS
NM_001039385	*Vgf*	BXD60	1.11E-03	-2.2	7.46E-04	-2.4
NM_177151	*Vps13b*	BXD60	8.35E-03	1.5	NS	NS
NM_029929	*Vps33a*	BXD60	3.82E-02	1.3	NS	NS
NM_145967	*Vstm2a*	BXD60	1.59E-02	-1.3	3.28E-02	-1.3
NM_016800	*Vti1b*	BXD60	3.22E-02	-1.3	NS	NS
NM_001177812	*Wbp1l*	BXD60	1.11E-02	1.3	2.29E-02	1.3
NM_153391	*Wdr19*	BXD60	1.54E-02	1.5	NS	NS
NM_173741	*Wdr24*	BXD60	4.31E-02	1.4	NS	NS
NM_172372	*Wdr45*	BXD60	8.02E-04	1.9	NS	NS
NM_134139	*Wdr74*	BXD60	NS	NS	1.40E-02	-1.5
NM_001159636	*Wdsub1*	BXD60	6.85E-03	1.7	NS	NS
NM_178398	*Wipi2*	BXD60	3.11E-02	1.3	NS	NS
NM_011728	*Xpa*	BXD60	1.96E-02	1.6	NS	NS
NM_011916	*Xrn1*	BXD60	1.33E-02	1.4	NS	NS
NM_198626	*Xxylt1*	BXD60	NS	NS	4.36E-02	-1.6
NM_207633	*Yipf6*	BXD60	6.75E-04	1.5	NS	NS
NM_001005342	*Ypel4*	BXD60	7.58E-07	3.3	4.19E-03	2.2
NM_027166	*Ypel5*	BXD60	2.26E-06	1.7	3.17E-05	1.6
NM_028106	*Zbed3*	BXD60	7.48E-04	1.6	NS	NS
NM_001033324	*Zbtb16*	BXD60	4.84E-02	1.6	3.71E-02	1.8
NM_175537	*Zbtb38*	BXD60	3.11E-02	1.3	NS	NS
NM_001115130	*Zbtb44*	BXD60	4.96E-02	1.3	4.60E-02	1.3
NM_173181	*Zc2hc1a*	BXD60	1.47E-03	-1.5	NS	NS
NM_178404	*Zc3h6*	BXD60	4.54E-02	1.4	NS	NS
NM_145931	*Zc3h7a*	BXD60	2.13E-02	1.5	NS	NS
NM_001003916	*Zc4h2*	BXD60	4.67E-02	1.4	NS	NS
NM_001033795	*Zcchc16*	BXD60	3.95E-02	1.6	NS	NS
NM_001101433	*Zcchc24*	BXD60	2.81E-02	1.5	NS	NS
NM_199309	*Zdhhc19*	BXD60	1.18E-02	-1.7	NS	NS
NM_001159905	*Zfand2b*	BXD60	2.69E-02	2.0	NS	NS
NM_011747	*Zfp13*	BXD60	6.56E-03	1.5	NS	NS

NM_001081217	*Zfp174*	BXD60	3.62E-02	1.5	NS	NS
NM_001113399	*Zfp385b*	BXD60	3.82E-02	1.4	NS	NS
NM_001110309	*Zfp426*	BXD60	1.54E-02	1.4	4.02E-02	1.4
NM_009557	*Zfp46*	BXD60	3.30E-02	1.4	NS	NS
NM_207255	*Zfp532*	BXD60	1.27E-03	1.5	NS	NS
NM_183149	*Zfp598*	BXD60	9.82E-03	-1.6	NS	NS
NM_001005425	*Zfp663*	BXD60	1.54E-02	2.3	NS	NS
NM_001289690	*Zfp740*	BXD60	3.68E-02	1.5	NS	NS
NM_001267583	*Zfp811*	BXD60	2.77E-03	1.6	1.93E-03	1.7
NM_178417	*Zfp867*	BXD60	3.41E-03	1.7	NS	NS
NM_177712	*Zfp874a*	BXD60	NS	NS	3.08E-02	1.7
NM_001076791	*Zfp874b*	BXD60	4.73E-02	1.5	NS	NS
NM_011763	*Zfp9*	BXD60	1.24E-02	1.5	NS	NS
NM_145563	*Zfp932*	BXD60	2.29E-04	1.5	NS	NS
NM_001042438	*Zhx1*	BXD60	1.53E-03	1.4	NS	NS
NM_177263	*Zhx3*	BXD60	1.17E-03	1.8	NS	NS
NM_026015	*Zmat5*	BXD60	3.08E-02	1.6	NS	NS
NM_001252584	*Zmynd8*	BXD60	NS	NS	1.37E-02	1.6
NM_023162	*Znrd1*	BXD60	9.91E-03	1.4	NS	NS
NM_009553	*Zscan2*	BXD60	NS	NS	3.14E-02	1.6
ENSMUST00000079024	*Zscan29*	BXD60	6.95E-04	1.9	NS	NS
NM_028028	*Zswim1*	BXD60	NS	NS	4.13E-02	-1.5
NM_027964	*Zswim2*	BXD60	3.49E-02	-1.5	NS	NS
NM_001045536	*Zzef1*	BXD60	4.79E-02	1.5	NS	NS
NM_001166372	*-44256*	BXD71	3.11E-03	1.6	NS	NS
NM_030210	*Aacs*	BXD71	NS	NS	3.33E-03	-1.6
NM_013851	*Abca8b*	BXD71	4.85E-03	2.1	4.33E-02	1.8
NM_025538	*Alkbh7*	BXD71	NS	NS	1.37E-02	-1.6
NM_028390	*Anln*	BXD71	3.15E-02	2.1	NS	NS
NM_007481	*Arf6*	BXD71	NS	NS	2.77E-02	-1.3
NM_001172205	*Arid5a*	BXD71	NS	NS	2.47E-02	1.9
NM_023598	*Arid5b*	BXD71	1.45E-02	1.6	3.28E-04	1.8
NM_001162485	*Arrdc1*	BXD71	3.22E-02	1.4	NS	NS
NM_001042591	*Arrdc3*	BXD71	1.29E-05	2.4	1.45E-04	2.2
NM_001042592	*Arrdc4*	BXD71	1.16E-02	1.8	1.47E-02	1.7
NM_001287180	*Atf4*	BXD71	NS	NS	1.12E-02	-1.5
NM_026217	*Atg12*	BXD71	4.54E-03	1.4	4.49E-02	1.3

NM_001081182	*Atp8b2*	BXD71	NS	NS	2.45E-02	1.7
NM_001199304	*Atxn1*	BXD71	3.08E-02	1.4	2.09E-02	1.4
NM_001080930	*Atxn1l*	BXD71	NS	NS	4.79E-02	1.5
NM_016847	*Avpr1a*	BXD71	4.38E-03	-2.0	6.99E-03	-1.9
NM_020025	*B3galt2*	BXD71	9.02E-03	1.6	1.45E-02	1.5
NM_175325	*Bbs4*	BXD71	4.31E-03	1.4	3.27E-03	1.4
NM_001284410	*Bcl2l11*	BXD71	5.01E-04	2.3	2.11E-03	2.1
NM_133889	*Bsdc1*	BXD71	NS	NS	3.28E-04	1.5
NM_007570	*Btg2*	BXD71	NS	NS	8.98E-03	1.8
NM_009770	*Btg3*	BXD71	NS	NS	8.32E-04	2.0
NM_144817	*Camk1g*	BXD71	4.42E-02	-1.4	NS	NS
NM_007625	*Cbx4*	BXD71	NS	NS	2.33E-02	-1.4
NM_172404	*Ccbl1*	BXD71	3.22E-02	1.6	NS	NS
NM_144820	*Ccdc28a*	BXD71	2.93E-02	1.9	NS	NS
NM_026014	*Cdt1*	BXD71	NS	NS	1.99E-02	-1.7
NM_001081345	*Chd2*	BXD71	NS	NS	3.83E-03	1.6
NM_001289429	*Cipc*	BXD71	NS	NS	1.73E-02	-1.4
NM_025854	*Cir1*	BXD71	NS	NS	4.25E-03	1.5
NM_001243762	*Clcn5*	BXD71	NS	NS	2.69E-03	1.8
NM_001252525	*Cpeb1*	BXD71	4.37E-02	1.9	1.16E-02	2.0
NM_172728	*Creb5*	BXD71	NS	NS	2.06E-02	1.9
NM_001110850	*Crem*	BXD71	NS	NS	1.80E-02	1.7
NM_007762	*Crhr1*	BXD71	3.24E-02	-1.7	2.02E-02	-1.7
NM_028979	*Cyp2j9*	BXD71	NS	NS	2.11E-03	2.0
NM_020010	*Cyp51*	BXD71	NS	NS	1.28E-02	-1.4
NM_019553	*Ddx21*	BXD71	3.15E-02	-1.5	NS	NS
NM_021494	*Dennd5a*	BXD71	NS	NS	3.76E-02	1.3
NM_053272	*Dhcr24*	BXD71	1.49E-02	-1.4	8.45E-08	-1.7
NM_007842	*Dhx9*	BXD71	NS	NS	2.31E-02	-1.3
NM_019454	*Dll4*	BXD71	1.45E-02	-1.8	NS	NS
NM_025926	*Dnajb4*	BXD71	1.63E-03	1.5	NS	NS
NM_029669	*Dnajc18*	BXD71	3.98E-03	1.5	NS	NS
NM_001285807	*Dtna*	BXD71	NS	NS	2.46E-03	1.4
NM_013642	*Dusp1*	BXD71	5.22E-08	2.5	4.11E-08	2.5
NM_001048054	*Dusp16*	BXD71	2.18E-02	1.8	9.73E-06	2.4
NM_176933	*Dusp4*	BXD71	NS	NS	3.28E-04	-2.1
NM_026268	*Dusp6*	BXD71	NS	NS	3.33E-03	-1.7
NM_008748	*Dusp8*	BXD71	3.62E-04	1.4	2.02E-05	1.5

NM_026932	*Ebna1bp2*	BXD71	NS	NS	3.80E-02	-1.4
NM_001289925	*Egr3*	BXD71	2.11E-02	-1.8	1.45E-04	-2.3
NM_023794	*Etv5*	BXD71	6.16E-03	-1.8	2.03E-02	-1.6
NM_001163514	*Extl2*	BXD71	NS	NS	1.10E-02	1.5
NM_001163567	*Fam102b*	BXD71	3.22E-02	1.3	NS	NS
NM_183187	*Fam107a*	BXD71	4.08E-03	2.5	9.57E-04	2.6
NM_178908	*Fam26e*	BXD71	2.09E-03	1.9	2.48E-03	1.9
NM_175104	*Fam53c*	BXD71	6.27E-03	1.8	3.12E-02	1.7
NM_010191	*Fdft1*	BXD71	NS	NS	8.38E-04	-1.4
NM_001164259	*Fgfrl1*	BXD71	NS	NS	1.76E-02	-1.6
NM_008013	*Fgl2*	BXD71	NS	NS	2.56E-03	1.8
NM_022009	*Flii*	BXD71	NS	NS	1.09E-02	-1.5
NM_001159706	*Folh1*	BXD71	NS	NS	4.50E-04	2.5
NM_008046	*Fst*	BXD71	4.25E-02	-1.7	1.88E-03	-1.9
NM_027518	*Gpr137c*	BXD71	9.55E-06	1.9	7.50E-06	1.9
NM_010338	*Gpr37*	BXD71	4.09E-04	2.0	3.83E-03	1.8
NM_145066	*Gpr85*	BXD71	3.15E-02	1.5	1.66E-02	1.5
NM_172768	*Gramd1b*	BXD71	2.78E-02	1.5	NS	NS
NM_001114333	*Grm1*	BXD71	4.42E-02	1.5	NS	NS
NM_198962	*Hcrtr2*	BXD71	NS	NS	2.46E-03	-1.9
NM_144835	*Heatr1*	BXD71	NS	NS	2.28E-02	-1.6
NM_010422	*Hexb*	BXD71	NS	NS	2.31E-02	1.4
NM_001098203	*Hic1*	BXD71	3.11E-02	-1.6	NS	NS
NM_010431	*Hif1a*	BXD71	2.53E-02	-1.3	NS	NS
NM_008255	*Hmgcr*	BXD71	NS	NS	8.41E-03	-1.5
NM_008256	*Hmgcs2*	BXD71	NS	NS	2.46E-03	2.4
NM_010476	*Hsd17b7*	BXD71	2.24E-03	-1.6	1.65E-02	-1.5
NM_024255	*Hsdl2*	BXD71	NS	NS	2.02E-02	1.5
NM_019564	*Htra1*	BXD71	2.30E-02	1.7	4.64E-03	1.8
NM_015755	*Hunk*	BXD71	NS	NS	3.00E-02	-1.7
NM_145360	*Idi1*	BXD71	7.98E-05	-1.8	8.45E-08	-2.2
NM_001101605	*Ifit1bl1*	BXD71	1.45E-02	2.1	NS	NS
NM_153526	*Insig1*	BXD71	3.69E-04	-2.0	2.46E-03	-1.8
NM_016851	*Irf6*	BXD71	NS	NS	1.56E-02	1.7
NM_010591	*Jun*	BXD71	2.72E-19	4.5	5.89E-15	3.6
NM_001286944	*Jund*	BXD71	3.77E-04	1.8	3.42E-04	1.7
NM_001081134	*Kcng1*	BXD71	NS	NS	6.79E-03	-1.8
NM_026214	*Kctd4*	BXD71	1.59E-02	1.5	NS	NS

NM_001122733	*Kit*	BXD71	NS	NS	3.28E-02	-1.3
NM_010636	*Klf12*	BXD71	NS	NS	2.66E-02	1.4
NM_078477	*Klf16*	BXD71	NS	NS	8.75E-03	-1.6
NM_001252658	*Ldlr*	BXD71	NS	NS	3.94E-03	-2.0
NM_001199043	*Lgals8*	BXD71	4.08E-03	1.6	NS	NS
NM_178005	*Lrrtm2*	BXD71	1.63E-02	1.4	NS	NS
NM_001045483	*Mapk1ip1*	BXD71	4.95E-03	1.5	3.33E-03	1.5
NM_011841	*Mapk7*	BXD71	1.45E-02	-1.4	NS	NS
NM_008563	*Mcm3*	BXD71	NS	NS	3.05E-02	-1.8
NM_008567	*Mcm6*	BXD71	3.15E-02	-1.5	NS	NS
NM_023799	*Mgea5*	BXD71	NS	NS	6.28E-03	1.4
NM_030110	*Micu3*	BXD71	NS	NS	2.63E-02	1.4
NM_021565	*Midn*	BXD71	NS	NS	1.65E-02	-1.5
NM_025436	*Msmo1*	BXD71	3.98E-03	-1.8	2.02E-05	-2.2
NM_013602	*Mt1*	BXD71	2.09E-03	1.6	2.77E-02	1.5
NM_008630	*Mt2*	BXD71	1.20E-04	2.4	1.39E-03	2.1
NM_008638	*Mthfd2*	BXD71	NS	NS	9.64E-03	-1.8
NM_138656	*Mvd*	BXD71	2.50E-02	-1.7	1.88E-03	-1.9
NM_001008542	*Mxi1*	BXD71	3.88E-03	1.5	9.79E-09	2.0
NM_028995	*Nipal3*	BXD71	NS	NS	1.70E-03	1.9
NM_021303	*Noc2l*	BXD71	NS	NS	1.65E-02	-1.5
NM_138747	*Nop2*	BXD71	NS	NS	1.45E-04	-1.7
NM_010342	*Npbwr1*	BXD71	1.63E-02	1.7	1.39E-03	1.9
NM_019409	*Omg*	BXD71	NS	NS	3.33E-03	1.5
NM_152818	*Osbp2*	BXD71	1.79E-02	1.4	4.17E-02	1.4
NM_001134791	*Osbpl9*	BXD71	NS	NS	2.34E-04	1.5
NM_001285839	*Osgepl1*	BXD71	NS	NS	1.18E-02	1.7
NM_008812	*Padi2*	BXD71	NS	NS	2.71E-02	-1.8
NM_146169	*Paip2b*	BXD71	NS	NS	2.14E-02	1.5
NM_153594	*Pcmtd2*	BXD71	NS	NS	4.35E-02	1.6
NM_024229	*Pcyt2*	BXD71	NS	NS	1.27E-02	-1.6
NM_001177980	*Pde4b*	BXD71	4.71E-03	1.5	3.92E-02	1.4
NM_008808	*Pdgfa*	BXD71	NS	NS	2.89E-02	-1.4
NM_001159367	*Per1*	BXD71	1.20E-02	1.6	9.68E-03	1.6
NM_053191	*Pi15*	BXD71	1.94E-02	2.0	NS	NS
NM_178149	*Pik3ip1*	BXD71	2.21E-03	2.0	1.39E-03	2.0
NM_001024955	*Pik3r1*	BXD71	5.00E-09	1.8	3.38E-10	1.9
NM_008872	*Plat*	BXD71	NS	NS	2.14E-02	1.6

NM_152813	*Plcd3*	BXD71	NS	NS	1.27E-02	1.7
NM_001033253	*Plekhg1*	BXD71	1.94E-02	1.6	7.14E-05	2.0
NM_013807	*Plk3*	BXD71	NS	NS	1.65E-02	-1.6
NM_001033225	*Pnrc1*	BXD71	NS	NS	1.96E-02	1.4
NM_175523	*Ppm1k*	BXD71	3.91E-03	1.7	NS	NS
NM_028980	*Ppp4r4*	BXD71	7.19E-03	1.6	NS	NS
NM_182997	*Prkab2*	BXD71	3.03E-02	1.4	1.05E-02	1.5
NM_177698	*Psd3*	BXD71	3.10E-02	1.3	NS	NS
NM_139270	*Pth2r*	BXD71	4.70E-02	-2.0	NS	NS
NM_025831	*Pxdc1*	BXD71	2.29E-03	1.8	NS	NS
NM_029494	*Rab30*	BXD71	1.55E-03	1.6	2.02E-05	1.8
NM_001038621	*Rabgap1l*	BXD71	2.09E-03	1.4	1.06E-02	1.4
NM_001099624	*Rapgef2*	BXD71	1.29E-09	1.9	1.41E-07	1.7
NM_001252494	*Rapgef6*	BXD71	1.37E-06	2.1	1.95E-07	2.2
NM_028234	*Rbm33*	BXD71	NS	NS	1.80E-02	1.4
NM_001141981	*Rbm43*	BXD71	NS	NS	2.24E-02	1.5
NM_009062	*Rgs4*	BXD71	NS	NS	5.41E-03	1.6
NM_153514	*Rhobtb2*	BXD71	NS	NS	1.76E-02	-1.5
NM_021540	*Rnf130*	BXD71	5.14E-03	1.4	NS	NS
NM_001166553	*Rnf145*	BXD71	NS	NS	1.95E-03	-1.5
NM_146244	*Rps6kl1*	BXD71	6.92E-04	1.9	3.28E-04	2.0
NM_172604	*Scara3*	BXD71	NS	NS	4.55E-03	1.6
NM_009129	*Scg2*	BXD71	NS	NS	2.46E-03	1.4
NM_011521	*Sdc4*	BXD71	3.22E-02	1.9	1.10E-02	2.0
NM_030261	*Sesn3*	BXD71	NS	NS	1.20E-03	1.5
NM_009167	*Shc3*	BXD71	NS	NS	1.76E-02	-1.3
NM_001286538	*Sigmar1*	BXD71	NS	NS	4.25E-03	-1.5
NM_001167983	*Sipa1l1*	BXD71	NS	NS	2.37E-02	1.4
NM_027921	*Slc16a14*	BXD71	1.63E-02	1.6	3.42E-04	1.8
NM_001029842	*Slc16a6*	BXD71	NS	NS	4.70E-02	1.9
NM_001159593	*Slc20a1*	BXD71	3.68E-02	-1.4	1.51E-03	-1.5
NM_172479	*Slc38a5*	BXD71	NS	NS	2.59E-02	-1.5
NM_001081263	*Slc44a5*	BXD71	6.49E-05	1.8	3.51E-04	1.7
NM_025690	*Sltm*	BXD71	NS	NS	1.47E-02	-1.4
NM_008539	*Smad1*	BXD71	NS	NS	1.81E-02	1.4
NM_027671	*Sntg1*	BXD71	NS	NS	1.99E-02	1.6
NM_182927	*Spred3*	BXD71	3.15E-02	-1.9	NS	NS
NM_011898	*Spry4*	BXD71	3.22E-02	-1.7	1.69E-02	-1.8

NM_009270	*Sqle*	BXD71	NS	NS	4.96E-04	-1.7
NM_011358	*Srsf2*	BXD71	NS	NS	8.49E-03	-1.5
NM_001195485	*Srsf7*	BXD71	NS	NS	8.64E-03	-1.7
NM_133774	*Stard4*	BXD71	NS	NS	2.02E-02	-1.7
NM_019675	*Stmn4*	BXD71	3.15E-02	1.6	2.93E-02	1.5
NM_153563	*Strip1*	BXD71	NS	NS	1.65E-02	-1.4
NM_153399	*Syne1*	BXD71	3.22E-02	1.5	NS	NS
NM_001040085	*Sytl2*	BXD71	NS	NS	2.92E-03	2.0
NM_029431	*Them4*	BXD71	NS	NS	3.45E-02	1.5
NM_177412	*Tmcc1*	BXD71	NS	NS	1.96E-02	1.4
NM_001160385	*Tmem196*	BXD71	NS	NS	1.51E-03	1.9
NM_177344	*Tmem203*	BXD71	NS	NS	9.32E-03	-1.8
NM_011607	*Tnc*	BXD71	NS	NS	2.31E-02	-1.5
NM_001122843	*Tnpo2*	BXD71	3.91E-03	1.4	NS	NS
NM_198102	*Tra2a*	BXD71	NS	NS	7.41E-03	-1.5
NM_009186	*Tra2b*	BXD71	NS	NS	7.57E-03	-1.4
NM_001170855	*Trim36*	BXD71	NS	NS	4.03E-02	-2.0
NM_027182	*Trip13*	BXD71	4.37E-02	-1.7	NS	NS
NM_001009935	*Txnip*	BXD71	NS	NS	6.22E-05	3.3
NM_001169576	*Ube2h*	BXD71	6.92E-04	1.6	6.96E-04	1.6
NM_001083319	*Ubp1*	BXD71	NS	NS	1.32E-02	-1.4
NM_030724	*Uck2*	BXD71	NS	NS	2.46E-03	-1.4
NM_001039385	*Vgf*	BXD71	NS	NS	4.25E-03	-2.2
NM_001142580	*Vipas39*	BXD71	1.63E-02	1.4	NS	NS
NM_029478	*Vmp1*	BXD71	NS	NS	3.24E-02	1.4
NM_001110015	*Wdr36*	BXD71	NS	NS	5.12E-03	-1.5
NM_001035226	*Xpo1*	BXD71	3.86E-02	1.5	NS	NS
NM_001005342	*Ypel4*	BXD71	2.09E-03	2.4	1.50E-02	2.1
NM_027166	*Ypel5*	BXD71	1.29E-05	1.7	3.04E-03	1.5
NM_001115130	*Zbtb44*	BXD71	1.23E-02	1.4	1.76E-02	1.4
NM_001110309	*Zfp426*	BXD71	4.38E-03	1.6	8.75E-03	1.5
NM_001252584	*Zmynd8*	BXD71	7.48E-04	1.8	NS	NS
NR_045177	*2900055J20Rik*	BXD73	1.15E-02	1.7	NS	NS
NR_015488	*A930003A15Rik*	BXD73	2.81E-02	1.9	NS	NS
NM_030210	*Aacs*	BXD73	4.52E-02	-1.5	8.85E-03	-1.6
NM_001190443	*Abcf2*	BXD73	5.26E-03	-1.4	NS	NS
NM_009338	*Acat2*	BXD73	3.70E-02	-1.4	NS	NS
NM_025538	*Alkbh7*	BXD73	6.48E-03	-1.6	NS	NS

NM_020581	*Angptl4*	BXD73	2.29E-02	1.9	2.44E-02	2.0
NM_001277188	*Ano4*	BXD73	4.10E-02	1.5	NS	NS
NM_009705	*Arg2*	BXD73	3.94E-02	1.9	NS	NS
NM_023598	*Arid5b*	BXD73	6.46E-04	1.7	NS	NS
NM_001042591	*Arrdc3*	BXD73	2.05E-07	2.8	2.35E-06	2.6
NM_001042592	*Arrdc4*	BXD73	1.79E-02	1.7	NS	NS
NM_027444	*Bbx*	BXD73	NS	NS	1.25E-02	1.8
NM_001284410	*Bcl2l11*	BXD73	3.04E-06	2.8	3.19E-03	2.2
NM_009747	*Bdkrb2*	BXD73	6.66E-04	2.2	NS	NS
NM_007570	*Btg2*	BXD73	3.36E-02	1.7	NS	NS
NM_009770	*Btg3*	BXD73	1.41E-02	1.8	NS	NS
NM_144817	*Camk1g*	BXD73	8.90E-03	-1.5	NS	NS
NM_001285446	*Cbfa2t2*	BXD73	1.12E-02	-1.4	NS	NS
NM_007631	*Ccnd1*	BXD73	4.35E-04	-1.8	NS	NS
NM_007635	*Ccng2*	BXD73	NS	NS	3.24E-02	1.5
NM_031998	*Cep41*	BXD73	4.42E-02	-2.0	NS	NS
NM_001081345	*Chd2*	BXD73	4.31E-02	1.4	NS	NS
NM_199008	*Cox11*	BXD73	NS	NS	1.78E-02	-1.5
NM_001252525	*Cpeb1*	BXD73	3.08E-02	1.9	NS	NS
NM_172728	*Creb5*	BXD73	3.73E-02	1.8	9.76E-03	2.0
NM_001110850	*Crem*	BXD73	7.82E-03	1.7	2.92E-02	1.7
NM_028868	*Cxxc1*	BXD73	4.79E-02	-1.4	NS	NS
NM_028979	*Cyp2j9*	BXD73	1.41E-02	1.8	NS	NS
NM_020010	*Cyp51*	BXD73	2.77E-03	-1.5	4.33E-03	-1.5
NM_019553	*Ddx21*	BXD73	1.03E-02	-1.6	NS	NS
NM_053272	*Dhcr24*	BXD73	4.61E-06	-1.6	2.35E-06	-1.6
NM_177648	*Dolk*	BXD73	2.57E-02	-1.4	NS	NS
NM_001285807	*Dtna*	BXD73	2.63E-02	1.3	NS	NS
NM_013642	*Dusp1*	BXD73	3.06E-03	1.8	2.23E-03	1.8
NM_026268	*Dusp6*	BXD73	8.69E-04	-1.8	NS	NS
NM_008748	*Dusp8*	BXD73	1.68E-04	1.5	3.10E-02	1.3
NM_013512	*Epb41l4a*	BXD73	4.60E-02	-1.5	NS	NS
NM_008815	*Etv4*	BXD73	NS	NS	1.78E-02	-1.6
NM_010171	*F3*	BXD73	6.55E-03	1.7	NS	NS
NM_183187	*Fam107a*	BXD73	1.08E-04	3.0	1.90E-02	2.3
NM_178908	*Fam26e*	BXD73	2.23E-03	1.9	NS	NS
NM_175104	*Fam53c*	BXD73	3.73E-02	1.7	NS	NS
NM_010191	*Fdft1*	BXD73	2.21E-04	-1.5	2.50E-03	-1.4

NM_001253751	*Fdps*	BXD73	1.27E-03	-1.8	NS	NS
NM_010234	*Fos*	BXD73	3.06E-03	2.3	1.87E-02	2.1
NM_008036	*Fosb*	BXD73	1.37E-03	2.7	2.40E-02	2.3
NM_010244	*Fv1*	BXD73	1.37E-03	-2.2	NS	NS
NM_028022	*Gatsl3*	BXD73	1.64E-02	2.1	NS	NS
NM_001205101	*Gm13212*	BXD73	NS	NS	2.99E-02	2.3
NM_001204913	*Gm20172*	BXD73	NS	NS	1.87E-02	2.2
NM_153547	*Gnl3*	BXD73	4.62E-02	-1.6	NS	NS
NM_027518	*Gpr137c*	BXD73	5.27E-06	1.9	2.93E-02	1.5
NM_145066	*Gpr85*	BXD73	2.29E-02	1.5	NS	NS
NM_001195774	*Gprc5b*	BXD73	2.37E-02	1.5	NS	NS
NM_153419	*Grwd1*	BXD73	NS	NS	1.27E-02	-2.0
NM_010360	*Gstm5*	BXD73	3.70E-02	1.3	NS	NS
NM_198962	*Hcrtr2*	BXD73	1.63E-02	-1.8	NS	NS
NM_144835	*Heatr1*	BXD73	8.14E-03	-1.7	4.29E-02	-1.6
NM_026101	*Herc4*	BXD73	8.59E-03	-1.6	NS	NS
NM_010431	*Hif1a*	BXD73	1.68E-02	-1.3	NS	NS
NM_008256	*Hmgcs2*	BXD73	5.48E-04	2.7	1.13E-02	2.3
NM_198937	*Hn1l*	BXD73	2.24E-02	-1.6	NS	NS
NM_053262	*Hsd17b11*	BXD73	2.05E-02	1.5	NS	NS
NM_010476	*Hsd17b7*	BXD73	7.60E-04	-1.7	7.99E-04	-1.7
NM_019564	*Htra1*	BXD73	2.91E-02	1.6	NS	NS
NM_145360	*Idi1*	BXD73	2.05E-07	-2.1	8.33E-07	-2.1
NM_013562	*Ifrd1*	BXD73	NS	NS	8.35E-03	-1.8
NM_010518	*Igfbp5*	BXD73	NS	NS	3.94E-02	1.6
NM_001033323	*Igsf9b*	BXD73	1.53E-02	1.8	NS	NS
NM_153526	*Insig1*	BXD73	1.57E-06	-2.3	5.97E-06	-2.3
NM_010591	*Jun*	BXD73	2.62E-08	2.5	2.35E-06	2.2
NM_001286944	*Jund*	BXD73	9.56E-06	1.9	4.29E-02	1.5
NM_001081134	*Kcng1*	BXD73	NS	NS	2.38E-02	-1.8
NM_080465	*Kcnn2*	BXD73	4.70E-02	1.4	NS	NS
NM_026200	*Kcnv1*	BXD73	4.12E-02	-1.7	NS	NS
NM_010636	*Klf12*	BXD73	NS	NS	3.94E-02	1.4
NM_078477	*Klf16*	BXD73	2.71E-02	-1.6	NS	NS
NM_010638	*Klf9*	BXD73	2.34E-02	1.5	NS	NS
NM_001252658	*Ldlr*	BXD73	5.10E-04	-2.3	3.61E-04	-2.3
NM_001045483	*Mapk1ip1*	BXD73	4.71E-02	1.4	NS	NS
NM_011841	*Mapk7*	BXD73	2.71E-02	-1.4	NS	NS

NM_008567	*Mcm6*	BXD73	1.79E-02	-1.5	NS	NS
NM_026896	*Med27*	BXD73	1.45E-03	-1.5	NS	NS
NM_023799	*Mgea5*	BXD73	3.30E-03	1.4	1.97E-04	1.5
NM_025878	*Mrps18b*	BXD73	NS	NS	1.06E-03	-1.8
NM_025436	*Msmo1*	BXD73	2.71E-05	-2.2	1.83E-04	-2.1
NM_013602	*Mt1*	BXD73	1.93E-04	1.8	NS	NS
NM_008630	*Mt2*	BXD73	1.57E-06	2.8	5.18E-03	2.1
NM_138656	*Mvd*	BXD73	2.42E-04	-2.1	5.38E-03	-1.9
NM_001008542	*Mxi1*	BXD73	1.57E-06	1.8	2.40E-04	1.6
NM_028757	*Nebl*	BXD73	NS	NS	9.71E-04	2.0
NM_001048168	*Nfyc*	BXD73	1.45E-02	-1.4	NS	NS
NM_001161329	*Nol11*	BXD73	1.82E-02	-1.4	NS	NS
NM_010941	*Nsdhl*	BXD73	1.08E-02	-1.4	NS	NS
NM_178644	*Oaf*	BXD73	NS	NS	1.36E-02	2.2
NM_146293	*Olfr1143*	BXD73	NS	NS	2.92E-02	-2.4
NM_146462	*Olfr1202*	BXD73	4.31E-02	2.4	NS	NS
NM_001134791	*Osbpl9*	BXD73	1.54E-02	1.3	5.59E-04	1.5
NM_029078	*Pcf11*	BXD73	3.75E-02	-1.5	NS	NS
NM_178149	*Pik3ip1*	BXD73	5.18E-03	1.9	NS	NS
NM_001024955	*Pik3r1*	BXD73	5.78E-05	1.5	2.87E-04	1.5
NM_152813	*Plcd3*	BXD73	2.89E-04	2.1	NS	NS
NM_001033253	*Plekhg1*	BXD73	1.37E-03	1.8	1.25E-02	1.7
NM_013807	*Plk3*	BXD73	4.26E-02	-1.6	2.40E-02	-1.6
NM_001033225	*Pnrc1*	BXD73	3.39E-02	1.4	NS	NS
NM_008654	*Ppp1r15a*	BXD73	4.10E-02	1.5	NS	NS
NM_145404	*Prmt7*	BXD73	4.92E-03	-1.5	NS	NS
NM_001136079	*Ptger4*	BXD73	4.91E-02	-1.7	NS	NS
NM_029494	*Rab30*	BXD73	5.41E-06	1.9	1.99E-03	1.6
NM_001099624	*Rapgef2*	BXD73	3.40E-04	1.5	NS	NS
NM_001252494	*Rapgef6*	BXD73	2.08E-05	2.0	2.40E-05	2.0
NM_028234	*Rbm33*	BXD73	NS	NS	3.37E-02	1.4
NM_009062	*Rgs4*	BXD73	1.41E-03	1.7	NS	NS
NM_001166553	*Rnf145*	BXD73	5.26E-03	-1.4	NS	NS
NM_146244	*Rps6kl1*	BXD73	6.55E-03	1.8	3.85E-03	1.8
NM_172604	*Scara3*	BXD73	1.60E-02	1.6	NS	NS
NM_011521	*Sdc4*	BXD73	2.71E-02	1.9	NS	NS
NM_030261	*Sesn3*	BXD73	8.68E-03	1.4	1.41E-03	1.5
NM_027921	*Slc16a14*	BXD73	1.46E-02	1.6	NS	NS

NM_001159593	*Slc20a1*	BXD73	2.18E-04	-1.6	NS	NS
NM_001199283	*Slc43a2*	BXD73	5.28E-04	1.8	NS	NS
NM_008539	*Smad1*	BXD73	1.14E-03	1.5	NS	NS
NM_001252481	*Smad2*	BXD73	NS	NS	1.87E-03	-1.7
NM_009238	*Sox4*	BXD73	1.84E-02	1.6	NS	NS
NM_182927	*Spred3*	BXD73	2.31E-02	-1.9	NS	NS
NM_009270	*Sqle*	BXD73	4.31E-02	-1.4	1.87E-02	-1.5
NM_009272	*Srm*	BXD73	7.09E-04	-1.6	NS	NS
NM_016795	*Srpk1*	BXD73	3.70E-03	-1.6	NS	NS
NM_011358	*Srsf2*	BXD73	2.23E-03	-1.5	NS	NS
NM_001195485	*Srsf7*	BXD73	3.06E-03	-1.8	NS	NS
NM_133774	*Stard4*	BXD73	8.91E-05	-2.1	2.01E-02	-1.7
NM_001040085	*Sytl2*	BXD73	9.37E-04	2.1	NS	NS
NM_177003	*Tigar*	BXD73	NS	NS	3.54E-02	-1.7
NM_133706	*Tmem97*	BXD73	NS	NS	4.84E-03	-1.9
NM_001127233	*Trp53*	BXD73	3.73E-02	-1.5	NS	NS
NM_001009935	*Txnip*	BXD73	4.38E-07	4.4	3.98E-05	3.5
NM_001169576	*Ube2h*	BXD73	1.45E-02	1.4	NS	NS
NM_020603	*Wdr46*	BXD73	NS	NS	4.29E-02	-1.8
NM_001005342	*Ypel4*	BXD73	2.88E-02	2.0	1.78E-02	2.1
NM_001033466	*Zbtb2*	BXD73	1.08E-02	-1.7	NS	NS
NM_001267583	*Zfp811*	BXD73	1.05E-03	1.7	NS	NS
